# Constrained TACC3 peptidomimetics for a non-canonical protein–protein interface elucidate allosteric communication in Aurora-A kinase†

**DOI:** 10.1039/d4sc06100d

**Published:** 2024-11-28

**Authors:** Diana Gimenez, Martin Walko, Jennifer A. Miles, Richard Bayliss, Megan H. Wright, Andrew J. Wilson

**Affiliations:** a School of Chemistry, University of Birmingham Edgbaston Birmingham B15 2TT UK a.j.wilson.1@bham.ac.uk; b School of Chemistry, University of Leeds Woodhouse Lane Leeds LS2 9JT UK M.H.Wright@leeds.ac.uk; c School of Molecular and Cellular Biology, University of Leeds Woodhouse Lane Leeds LS2 9JT UK R.W.Bayliss@leeds.ac.uk; d Astbury Centre for Structural Molecular Biology, University of Leeds Woodhouse Lane Leeds LS2 9JT UK

## Abstract

Peptidomimetic design for non-canonical interfaces is less well established than for α-helix and β-strand mediated protein–protein interactions. Using the TACC3/Aurora-A kinase interaction as a model, we developed a series of constrained TACC3 peptide variants with 10-fold increased binding potencies (*K*_d_) towards Aurora-A in comparison to the parent peptide. High-affinity is achieved in part by restricting the accessible conformational ensemble of the peptide leading to a more favourable entropy of binding. In addition to acting as potent orthosteric TACC3/Aurora-A inhibitors, these peptidomimetics were shown to activate the kinase and inhibit the N-Myc/Aurora-A interaction at a distal site. Thus, the potency of these tools uniquely allowed us to unveil new insight into the role of allosteric communication in the kinase.

## Abstract 2

Rational design is used to optimize peptidomimetic inhibitors of the TACC3/Aurora-A kinase interaction.

## Graphical abstract

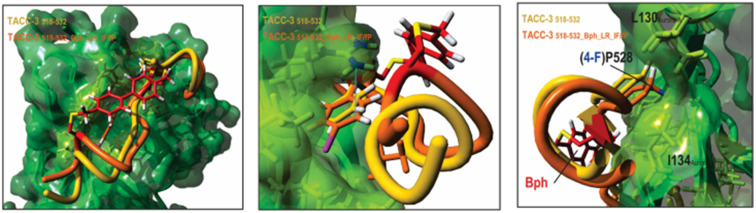

## 1 Introduction

Protein–protein interactions (PPIs) play a crucial mechanistic role in regulating health and disease biology.^1,2^ PPIs thus represent important targets in drug discovery.^3^ Competitive/orthosteric PPI inhibition has been historically challenging given that protein–protein interfaces are relatively large and lack the well-defined pockets that are characteristic of traditional drug targets.^4,5^ Although success has been achieved using fragment-based approaches, small-molecules developed from screening libraries and computational methods,^6^ there remains a need to develop new enabling methods and modalities for PPI modulation. Peptides are attractive given they offer functionally optimal molecular recognition properties.^7,8^ Significant efforts have been invested in developing peptidomimetic inhibitors of α-helix mediated PPIs, with cyclization employed as a tool to bias a given peptide toward its bioactive conformation, suppress proteolysis, and improve cell uptake.^9^ Emerging studies extend this approach to β-strand^10–12^ and loop^13^ mediated PPIs, whilst screening tools have yielded cyclic and bicyclic peptides for a range of other targets.^14,15^ However, strategies for the design of constrained peptides that mimic non-canonical secondary structures are less well developed. Whilst general principles to “pre-organise” irregular structures have to be elaborated, hydrophobic cross-links that replace crucial ExoS residues involved in 14-3-3 binding were rationally introduced and optimized to stabilize an irregular bound peptide structure.^16^ Herein, we used rational design to develop a peptidomimetic inhibitor of the TACC3/Aurora-A interaction. Judicious incorporation of non-natural amino acids and constraints led to a peptide with enhanced affinity for its target as a consequence of excluding accessible conformations thus raising the ground state energy of the conformational ensemble. The power of these peptidomimetic tools to understand the target protein is illustrated through experiments showing that they activate Aurora-A kinase and inhibit a further PPI (N-Myc/Aurora-A) at a remote site.

## 2 Results and discussion

Aurora-A is a Ser/Thr protein kinase that plays an essential role in mitosis.^17^ Aberrant Aurora-A function is associated with cancer development and progression making it an attractive drug discovery target. However, despite entry of numerous active site Aurora-A inhibitors into clinical trials,^18–21^ none have progressed to clinical use. This might arise due to on-target toxicity associated with the essential function of Aurora-A^22,23^ but also because of Aurora-A functions that are independent of its intrinsic kinase activity.^24,25^ This means that active site kinase inhibitors might not suffice to achieve therapeutic efficacy. As an incomplete kinase, Aurora-A function and localization is regulated through recognition of a plethora of clients. Among those it recruits and phosphorylates is Transforming Acidic Coiled-Coil Containing Protein 3 (TACC3). TACC3 is instrumental for spindle assembly and chromosome segregation in mammalian cells, playing a central role in achieving microtubule stabilization during mitosis through its interaction with chTOG/XMAP215.^26,27^ TACC3 is mutated and overexpressed in different cancer types, including glioblastoma, with its fusion products also exerting oncogenic activity.^28,29^ Moreover, depletion or pharmacological inhibition of Aurora-A kinase activity by small-molecule inhibitors has been shown to disrupt centrosomal localization of TACC3,^30^ and suppress tumor growth,^31^ whilst siRNA-mediated TACC3 depletion leads to a similar phenotype to that which occurs through Aurora-A active-site inhibition.^32,33^

**Fig. 1:**
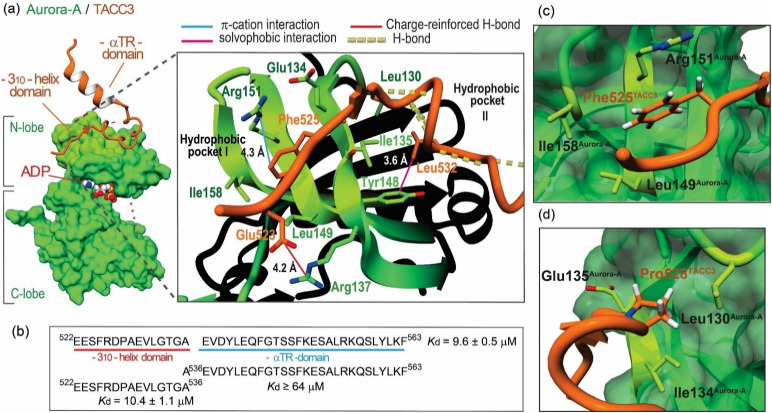
Key features of the TACC3/Aurora-A complex: (a) crystal structure of the Aurora-A_122-403-C290A/C393A/D274N_ catalytic domain (forest-green) in complex with TACC3_522-563_ (orange; PDB: 5ODT), magnified view of the TACC3/Aurora-A PPI interface with key residues shown as sticks, and TACC3 hot-spot residues highlighted; (b) sequence of TACC3 docking region to Aurora-A, TACC3_522-563_, and individual dissociation constants (*K*_d_) for each domain as measured by FA direct binding assays. *K*_d_ values are given as the mean value and corresponding standard deviation (SD) from triplicate titrations of Aurora-A _122-403-C290A/C393A_ in the presence of the corresponding FAM-labeled peptides (50 nM) (*n* = 3); (c) magnified view of the MD calculated energy minimum structure of WT TACC3_522-536_ (in orange) in the presence of Aurora-A, showing the arrangement around the key Phe525^TACC3^; (d) of the MD calculated energy minimum structure of WT TACC3_522-536_ (in orange) in the presence of Aurora-A, showing the *exo*-pucker conformation and arrangement of Pro528^TACC3^.

Burgess *et al.* reported an X-ray crystal structure for a fragment of TACC3 (residues 519–563) bound to Aurora-A_122-403 C290A/C393A/D274N_ (PDB: 5ODT; Fig. 1a).^26^ The TACC3 fragment was shown to adopt an extended conformation with two short regions adopting secondary structure: a 3_10_-helical turn (residues 527–531), and an α-helix (residues 546–555, the αTR domain, Fig. 1a). Fluorescence anisotropy (FA) titrations established that TACC3_522-536_ – encompassing the 3_10_-helical turn – makes the dominant contribution to Aurora-A affinity (∼10–20 μM).^27^ Microscale thermophoresis further identified Phe525^TACC3^ as a hot-spot residue for Aurora-A binding. Moreover, HeLa cells that express a F525A variant of TACC3 generated using CRISPR-Cas9 exhibited a phenotype consistent with critical roles of this hot-spot in early and late mitosis.^26,27^

Our first objective was to optimize the affinity of TACC3_522-536_ for Aurora-A. Our analyses of the TACC3/Aurora-A co-crystal structure identified three interactions that might play a role in influencing the affinity of TACC3 for Aurora-A: a charge-reinforced hydrogen-bond between Glu523^TACC3^ and Arg137^Aurora-A^; a hydrophobic contact between Leu532^TACC3^ and Tyr148^Aurora-A^; and, a cation–π interaction between Phe525^TACC3^ and Arg151^Aurora-A^. These analyses were supported through *in silico* alanine scanning (Table S1†).^34^

### 2.1 Sequence variation leads to optimization of the Phe525^TACC3^ interaction with Aurora-A

**Fig. 2:**
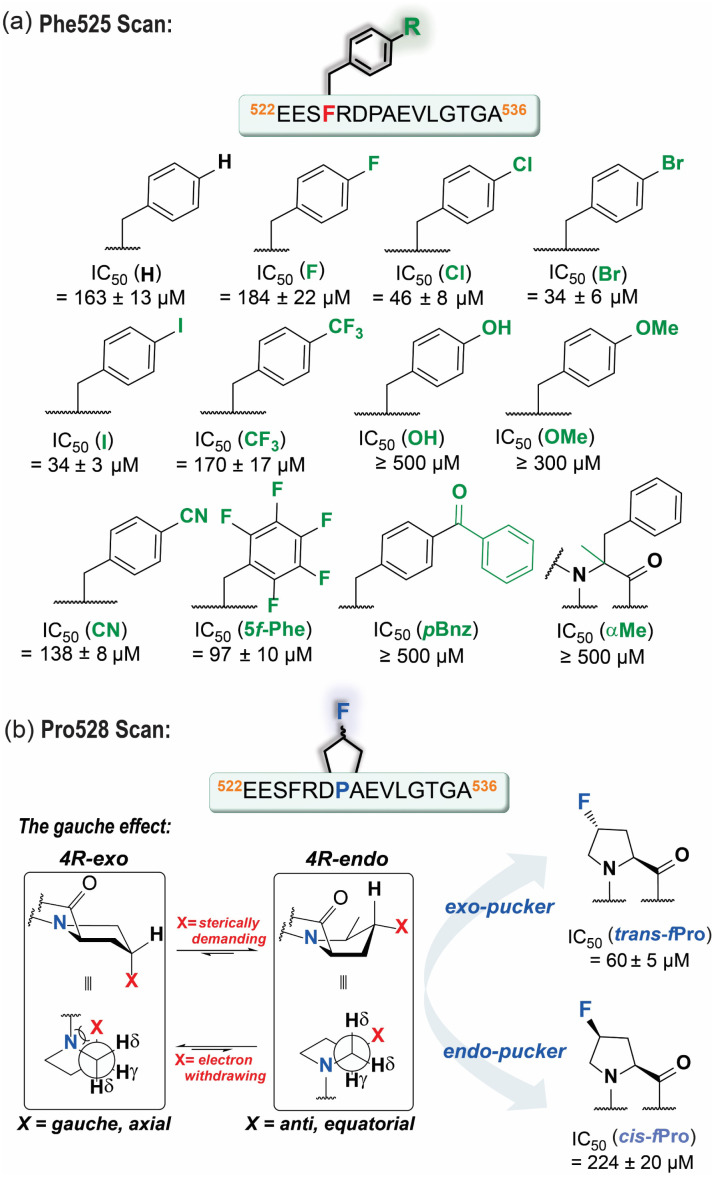
Single-point amino-acid modification: (a) sequence and chemical structure of the different Phe525 analogs tested and their corresponding competition FA IC_50_ values; (b) schematics illustrating the gauche effect in fluoro-prolines and the corresponding competition FA IC_50_ values assessed for the TACC3_522-536_ variants carrying these modifications. All IC_50_ values are reported as the average value ± SD of triplicate competition experiments (*n* = 3) against 200 nM FAM-Ahx-TACC3_522-536_ at 25 °C in 25 mM Tris, 150 mM NaCl, 5 mM MgCl_2_, pH 7.5, using 5 μM Aurora-A_122-403-C290A/C393A_.

We hypothesized that *para*-substituted phenylalanine analogs able to occupy an increased volume within the Phe525^TACC3^ binding pocket (Leu149^Aurora-A^, Ile158^Aurora-A^, Arg151^Aurora-A^) could enhance solvophobic packing and modulate the strength of the Phe525^TACC3^/Arg151^Aurora-A^ cation–π interaction (Fig. 1c). We prepared a series of TACC3_522-536_ variants where a variety of unnatural phenylalanine analogs were incorporated in place of the naturally occurring Phe525^TACC3^ (Fig. 2). Their IC_50_ values (Fig. 2, S1 and Table S2†) were then determined through competition FA experiments against the fluorescently labeled TACC3_522-536_ WT sequence bound to Aurora-A_122-403-C290A/C393A_. Compared to the WT peptide (IC_50_ = 163 ± 13 μM), up to ∼5-fold enhanced inhibitory potencies for three of the variants based on simple halogen *para*-substituted analogs were observed: TACC3_522-536-(4-Cl)525F_ (IC_50_ = 46 ± 8 μM), TACC3_522-536-(4-Br)525F_ (IC_50_ = 34 ± 6 μM) and TACC3_522-536-(4-I)525F_ (IC_50_ = 34 ± 3 μM). For the smaller (4-F)^525^Phe variant no improvement was observed, indicating that both the halogen-atom size and electronic properties play a role in enhancing affinity towards Aurora-A. To provide further insight on the improved affinities, molecular dynamics (MD) analyses were carried out by modelling the WT and the iodinated variant (TACC3_522-536-(4-I)525F_) in complex with Aurora-A (Fig. S2–S7†). The calculated minimum energy structures were consistent with deeper insertion of (4-I)Phe525^TACC3^ into its hydrophobic pocket on Aurora-A (see Fig. S5–S7†). Additionally, MD analyses of the number of contacts per-residue between TACC3_522-536(4-I)525F_ and the protein over the course of the trajectory indicated increased and more sustained contacts between the iodinated amino acid and Aurora-A (see Fig. S4†*vs.* Fig. S7†). In their minimum energy structures, a shorter distance between Arg151^Aurora-A^ and Phe525^TACC3^ (3.9 Å for (4-I)Phe525 *vs.* 4.2 Å for Phe525) is consistent with a stronger cation–π interaction.

### 2.2 A non-natural amino acid promotes conformational selection at the Pro528^TACC3^ residue

MD analyses alongside *in silico* alanine scanning^34^ were instrumental in examining additional residues for further optimization (Fig. 2, S2–S4 and Table S1†). Both analyses indicated that when bound to Aurora-A, residues: Val531^TACC3^ (ΔΔ*G*_pred_ = 3.6 kJ mol^−1^), Leu532^TACC3^ (ΔΔ*G*_pred_ = 8.2 kJ mol^−1^) and Pro531^TACC3^ (ΔΔ*G*_pred_ = 4.8 kJ mol^−1^) are important for binding and establish a significant number of contacts with the surface of Aurora-A (Fig. S4†). We also observed in the energy minimum structure of the complex, that Pro531^TACC3^ exhibits a C^γ^-*exo*-pucker conformation, which likely facilitates deeper insertion of the side-chain into a hydrophobic pocket on Aurora-A (Leu130^Aurora-A^, Ile134^Aurora-A^ and Glu135^Aurora-A^Fig. 1d and S2†). The *exo*-pucker conformation at the proline residue allows a peptidyl-prolyl *trans*-amide bond of the preceding residue, which favors the accommodation and propagation of the TACC3 backbone chain across the surface of Aurora-A.

We assessed the effects of sequence variation at Leu532^TACC3^ and Val531^TACC3^ by replacing them with isoleucine or norleucine (nLeu). However, poor tolerance to these modifications was observed (TACC3_522-536-L532nL_ IC_50_ = 213 ± 16 μM, TACC3_522-536-V531I_ IC_50_ ≥ 300 μM, TACC3_522-536-V531nL_ IC_50_ ≥ 300 μM *vs.* TACC3_522-536_ IC_50_ = 163 ± 13 μM; Fig. S8†).

Exploiting stereo-electronic effects (*i.e.* gauche effect; Fig. 2b) to favor the Pro528 *exo*-pucker conformation observed in the TACC3/Aurora-A structure was more profitable.^35^ Incorporation of fluorine has proven effective in controlling the prolyl-ring preference, with *trans*-(4-F)Pro favoring the C^γ^-*exo*-pucker conformation at a 6 : 1 ratio, and *cis*-(4-F)Pro biasing the equilibrium to the opposite *endo*-conformer in an estimated 20 : 1 ratio.^36^ When TACC3_522-536_ variants based on both possible (4-F)Pro isomers (*i.e. cis* or *trans*-fluoroproline) were tested using competition FA assays (Fig. S9†), we observed that the *exo*-pucker stabilizing *trans*-isomer had ∼3-fold increased TACC3/Aurora-A inhibitory potency (TACC3_522-536-*trans*-(4-F)528P_ IC_50_ = 60 ± 5 μM *vs.* TACC3_522-536_ IC_50_ = 163 ± 13 μM; Fig. 2b). In contrast, incorporation of the *endo*-pucker stabilizing *cis*-(4-F)Pro residue resulted in the opposing effect, reducing the inhibitory potency of the peptide to IC_50_ ≥ 200 μM. Overall, these data (reinforced with MD simulations on the *trans*-(4-F)Pro variant Fig. S10–S12† for further discussion) demonstrate that the TACC3/Aurora-A interaction is sensitive to the conformation of Pro528^TACC3^ and that inhibitory potency can be tuned using synthetic variation.

### 2.3 Constraining TACC3 leads to improved inhibitory potency

Although the TACC3/Aurora-A interaction lacks a defined secondary structure, TACC3 becomes more ordered on binding to Aurora-A.^26^ We, therefore, explored the use of a maleimide crosslinker to constrain the cysteine variants of the WT peptide between the *i* and *i*+3 positions to limit the conformational landscape of the peptide and perhaps favor formation of the 3_10_-helical turn observed in the crystal structure (Table S3 and Fig. S13†).^37–39^ These variants did not lead to enhanced inhibitory potency (Table S3 and† Fig. S13†).

**Fig. 3:**
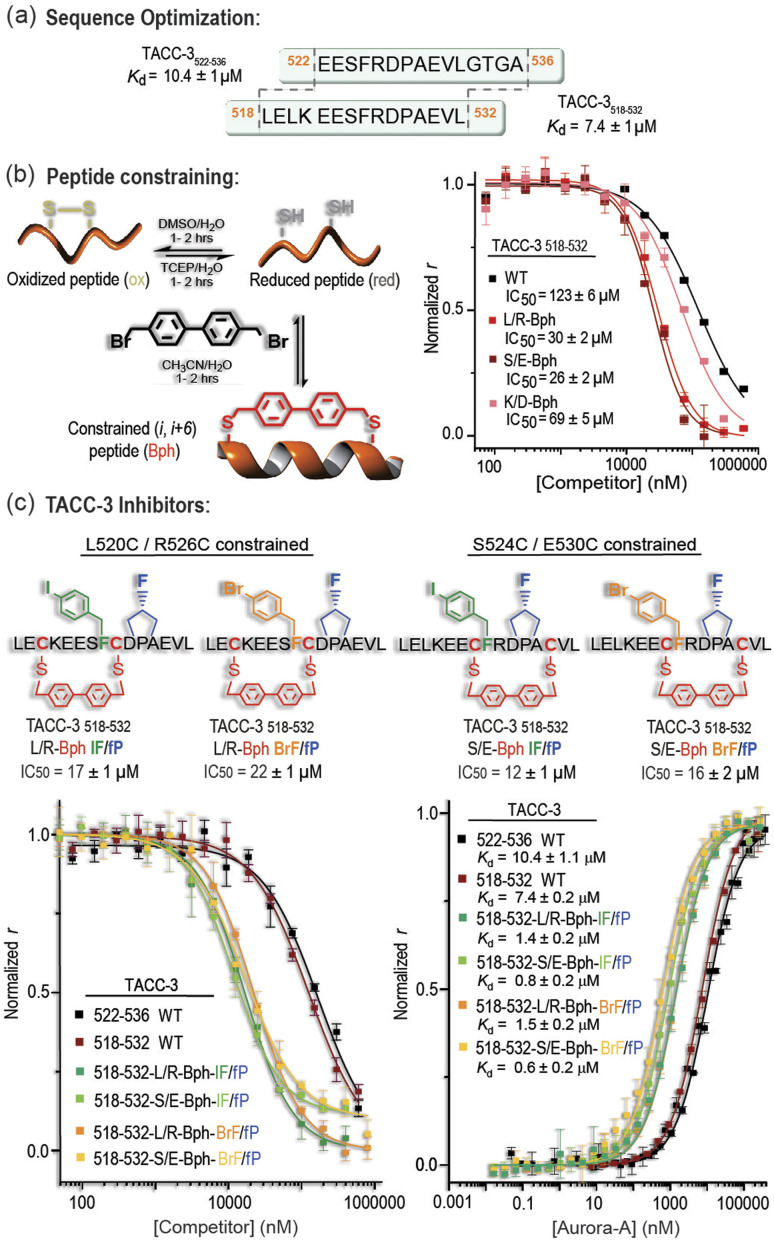
Novel constrained TACC3/Aurora-A PPI inhibitors: (a) sequence and *K*_d_ values measured for WT peptides TACC3_522-536_ and TACC3_518-532_; (b) schematics of peptide constraint; (c) structure of lead Bph-constrained TACC3/Aurora-A PPI inhibitors. (Left lower panel) Competition FA results for lead inhibitors against TACC3_522-536_ WT (5 μM Aurora-A_122-403-C290A/C393A_, 200 nM FAM-Ahx-TACC3_522-536_). (Right lower panel) FA direct titration of FAM-Ahx-inhibitors (50 nM) with Aurora-A_122-403-C290A/C393A_ (all values are given as the average ± SD of a triplicate assay in 25 mM Tris, 150 mM NaCl, 5 mM MgCl_2_, pH 7.5, 25 °C).

Better inhibitory potencies were obtained using longer constraints (*i.e.* 4,4′-bis(methyl)biphenyl, Bph) between cysteines introduced at *i* and *i*+6 positions (Table S3 and Fig. S13†). To better accommodate such a constraint into the peptide, we also re-explored the TACC3 sequence by truncating and elongating the TACC3_522-536_ peptide. We found TACC3_518-532_, with four additional residues at the N-terminus and four fewer at the C-terminus, to be a suitable template for additional development (Fig. 3a, S14 and Table S4†). This sequence had marginally improved binding potency to Aurora-A when compared to the original TACC3_522-536_ peptide (TACC3_518-532_*K*_d_ = 7.4 μM *vs.* TACC3_522-536_*K*_d_ = 10.4 μM; Fig. 3a), but more importantly opened up the tactical modification of additional residues not present in the original TACC3_522-536_ sequence.

With the aid of MD analysis on TACC3_518-532_ (Fig. S15–S17†), we identified accessible solvent-exposed L520/R526, K521/D528, and S524/E530 residue pairs as promising options for introduction of cysteines and then addition of a constraint with a variety of linkers. When these TACC3_518-532_-based variants were tested, up to 4-fold improved IC_50_ values were found for two of the biphenyl-constrained peptides, both showing inhibitory concentrations in the range of IC_50_ ∼ 30 μM (TACC3_518-532-L/R-Bph_ and TACC3_518-532-S/E-Bph_; Fig. 3b). In comparison, larger IC_50_ values were observed for the corresponding reduced and oxidized species, or when using alternative more flexible, and/or hydrophobic constraints such as octanoyl and polyethylene glycol-based linkers (Table S5, Fig. S18, S19–S22† for MD analyses and discussion).

### 2.4 Synergistic incorporation of constraints and non-natural amino-acids further improves TACC3 affinity for Aurora-A

With two amino-acid modifications that enhanced binding potency of TACC3 to Aurora-A and two constrained backbones identified, we assessed the extent to which these modifications could be productively combined in a single compound (Fig. 3c). Four modified peptides were assessed: TACC3_518-532-L/R-Bph-I_ and TACC3_518-532-L/R-Bph-Br_ based on the L520C/R526C biphenyl-constrained structure carrying either (4-I)Phe525 or (4-Br)Phe525 modifications, and the corresponding peptides: TACC3_518-532-S/E-Bph-I_ and TACC3_518-532-S/E-Bph-Br_, based on the alternative S524C/E530C constrained template (Fig. S23†). All variants exhibited IC_50_ values of ∼20–30 μM against the WT peptide (Fig. S23†). Next, we assessed the effect of incorporating the *trans*-(4-F)Pro528 residue by synthesizing and analyzing the corresponding peptides carrying this modification (TACC3_518-532-L/R-Bph-I/fP_; TACC3_518-532-L/R-Bph-Br/fP_; TACC3_518-532-S/E-Bph-I/fP and_ TACC3_518-532-S/E-Bph-Br/fP_; Fig. 3c, S24, S25 and Table S6†). These variants showed lower IC_50_ values in competition experiments than previous compounds, with all peptides exhibiting IC_50_ values in the range 10–22 ± 2 μM (the limit of sensitivity for the assay). To more accurately assess binding potencies to Aurora-A, we employed direct FA titrations of the protein in the presence of FAM-labelled peptides and assessed their corresponding dissociation constants, *K*_d_. Twelve-fold improved *K*_d_ values were measured for the constrained peptides when compared to the linear sequences (Fig. 3c), with all variants showing low micromolar/high nanomolar affinities. MD simulations indicate that these combined modifications act as designed *i.e.* the halogenated phenylalanine better occupies its pocket and the *trans*-fluoroproline favors the *exo*-pucker conformation (Fig. S26–S33†).

Peptide constraints and unnatural amino acids can suppress proteolysis.^40^ The L/R constrained peptide exhibited increased resistance to both trypsin (cleavage at positively charged residues) and α-chymotrypsin (cleavage at aromatic and hydrophobic aliphatic residues) whilst the S/E constrained variant was not protected in comparison to the WT sequence, but did exhibit a change in cleavage site (Fig. S34–S40†).

Overall, these results establish the successful complementary incorporation of peptide constraint, unnatural amino acid, and proline conformational control as a means to rationally develop TACC3/Aurora-A inhibitors.

### 2.5 The constraint restricts the conformational landscape of TACC3 and pre-disposes Phe525 towards Aurora-A binding

**Fig. 4:**
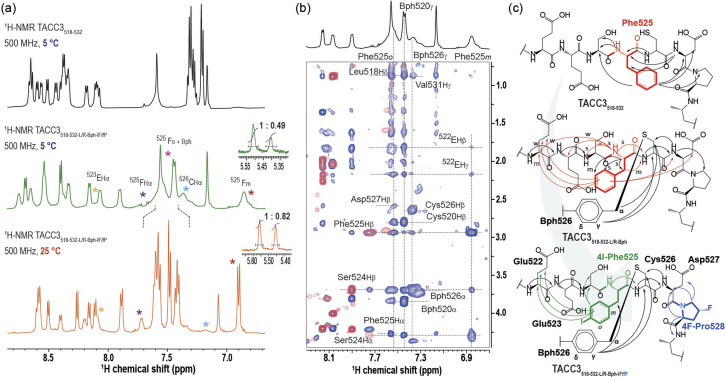
NMR secondary structure analysis of TACC3_518-532_ and constrained TACC3 _518-532_Bph_LR_IF/fP_: (a) ^1^H-NMR spectra of TACC3_518-532_ (in black) and TACC3_518-532_L/R_Bph _IF/fP_. (in green) showing the amide-bond region of the peptides as observed at 5 °C; shown in orange is the ^1^H-NMR spectra of TACC3_518-532_L/R_Bph_IF/fP_ as observed at 25 °C; (b) superposition of the ^1^H–^1^H TOCSY (in red) and ^1^H–^1^H NOESY spectra (in blue) at the NH-Hα region of constrained TACC3_518-532_L/R_Bph_IF/fP_; (c) schematics showing the comparative intramolecular spatial ^1^H–^1^H NOESY contacts and the phenylalanine aromatic ring orientation as observed within TACC3_518-532_ WT, control constrained TACC3_518-532_L/R_Bph_ and TACC3_518-532_Bph_LR_IF/fP_. All samples as measured in buffer/D_2_O 90/10% vol/vol. Buffer: 25 mM potassium phosphate, 50 mM NaCl, 5 mM MgCl_2_, pH = 7.5.

To better understand the role of the modifications in increasing the affinity of the TACC3 peptidomimetics towards Aurora-A, we studied their unbound solution structure by NMR spectroscopy (Fig. 4). At 5 °C, we found that TACC3_518-532_ showed well-resolved ^1^H resonances (Fig. 4a, S41–S49 and Table S7†), exhibiting amide-bond N–H vicinal coupling constants for each residue, ^3^*J*_NH–αH_, persistently in the range of 6–7 Hz, consistent with a general disordered/random coil secondary structure.^41^ Analysis of TACC3_518-532_ based on recently published random-coil chemical shifts for disordered proteins (Δ*δ*_RC_)^42^ supported this conclusion; both Cα (Δ*δ*_Cα_) and Hα (Δ*δ*_Hα_) secondary chemical shifts showed extended regions with small negative values (|Δ*δ*_Cα_|≤ 1 ppm; |Δ*δ*_Hα_|≤ 0.25 ppm), much lower and for Δ*δ*_Hα_ of opposing sign, to those of α-helical structures (Δ*δ*_Cα_ ≥ 2.0–3.5 ppm; Table S8,† Fig. S50–S52†).^43,44^ We found no evidence of a folded (helical) structure in solution when the corresponding TOCSY/NOESY spectra were analyzed; only correlations between consecutive (*i*, *i*−1) amide bonds were observed. Modified peptide TACC3_518-532-L/R-Bph-IF/fP_, exhibited broadened ^1^H resonances of lower intensity, in particular for the constrained Bph group, residues Cys526^TACC3^, Glu523^TACC3^, and (4-I)Phe525^TACC3^ (Fig. 4a, S53–S60 and Table S8† for full spectral data at 5 °C). This indicates slower interconversion between conformers, in contrast to the unconstrained peptide. We have observed such behavior previously for constrained peptides that target helical interfaces.^39^ To further probe the effect of the constraint in TACC3_518-532-L/R-Bph-IF/fP_, we carried out ^1^H variable temperature (VT) NMR experiments from 5–50 °C (Fig. S61 and S62†). Upon gradually increasing the temperature we measured a progressive shift to higher signal intensities (Fig. 4a), improved spatial resolution, and resonance narrowing, confirming that exchange between conformers occurs at low temperatures. TOCSY/NOESY spectra of TACC3_518-532-L/R-Bph-IF/fP_ revealed similar structural features to those found for the WT peptide, and were indicative of a disordered/random-coil structure based on ^1^H_NH_ and *δ*_RC_ chemical shifts (Table S10, Fig. S50–S52†); however, some key differences were observed. Firstly, we observed a clear conformational bias at the *trans*-(4-F)Pro528^TACC3^ residue in the constrained peptide towards the desired *exo*-pucker/*trans*-amide population that persisted even at room temperature (*K*_*exo*/*endo*_ (5 °C) = 2.04; *K*_*exo*/*endo*_ (25 °C) = 1.22; insets Fig. 4a and Table S11†). Secondly, we observed more extensive nOe correlations for Phe525^TACC3^. Spectral correlations for the WT unconstrained peptide indicate Phe525^TACC3^ contacts both the N-terminal residue (Ser524^TACC3^) and the C-terminal residues (Asp527^TACC3^, Pro528^TACC3^) (Fig. S38–S41†), whereas for TACC3_518-532-L/R-Bph-IF/fP_ the correlations were consistent with preferential orientation towards the N-terminus (Glu522^TACC3^ and Ser524^TACC3^, Fig. 4b, c, S58–S60†). These results indicate that whilst Phe525^TACC3^ freely rotates about the C_α_–C_β_ dihedral in the WT sequence, in the constrained peptide its orientation is biased to that observed when bound to Aurora-A. We also examined the constrained variant TACC3_518-532-L/R-Bph_, where the (4-I)Phe525 and *trans*-(4-F)Pro528 are not present (Tables S12, 13,† Fig. S63–S71†). We observed intermediate behavior for this peptide (Fig. 4c), with nOe correlations between residues on either side of Phe525, but with higher intensity for N-terminal residues. Finally, we analyzed our second constrained model peptide, TACC3_518-532-S/E-Bph_ (Tables S14, S15, Fig. S72–S80†) and TACC3_518-532-S/E-Bph-IF/fP_ (Tables S16, 17, Fig. S81–S89†). Results from these experiments were consistent with those observed for TACC3_518-532-L/R-Bph-IF/fP_, showing for the S/E Bph-constrained peptide a similar random-coil structure (Table S17, Fig. S50–52†), orientational bias of Phe525^TACC-^ towards the N-terminus (Fig. S86–S89†) and bias of the *trans*-(4-F)Pro528 residue towards the *exo*-pucker/*trans*-amide configuration (*K*_*exo*/*endo*_ = 2.70, Table S11, Fig. S90 and S91†).

Taken together, these NMR data indicate a combination of all three modifications restricts the conformational ensemble in the constrained peptidomimetics, presumably disfavouring some that are unproductive for binding and in effect predisposing the peptides towards higher affinity Aurora-A binding.

### 2.6 Enhanced binding of peptidomimetics to Aurora-A is entropically driven

**Fig. 5:**
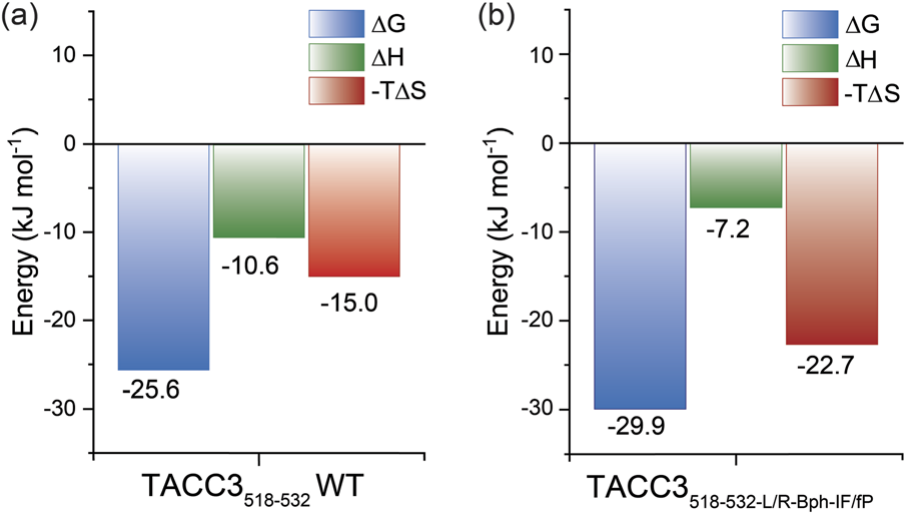
Thermodynamics of binding in the presence of Aurora-A: (a) ITC thermodynamic signatures of linear TACC3_518-532_; (b) constrained TACC3_518-532-L/R-Bph-I/fP_ binding to Aurora-A_122-403-C290A/C393A_. Data is shown as the average ± SD of two independent titrations of the protein (46 μM) in 25 mM Tris buffer, 150 mM NaCl, 5 mM MgCl_2_, 5% v/v glycerol at pH 7.5.

Whilst the NMR analyses indicated that the constrained peptidomimetics are conformationally more restricted in comparison to the linear variant, they also indicated the constrained peptide ensemble has a largely disordered/random-coil conformation. To explore how restricting the accessible conformations influences the thermodynamics of binding, we carried out isothermal titration calorimetry (ITC) experiments (Fig. 5 and Table S18†). By comparing the individual contributions of enthalpy and entropy for the interaction of constrained and unconstrained TACC3 variants to Aurora-A (Fig. 5), the constrained variants (*e.g.* TACC3_518-532-LR-Bph-IF/fP_ −*T*Δ*S* = −22.7 kJ mol^−1^) were observed to exhibit a more favorable entropy of binding in comparison to the linear variants (TACC3_522-536_ −*T*Δ*S* = −2.7 kJ mol^−1^, TACC3_518-532_*T*Δ*S* = −15.0 kJ mol^−1^; Fig. S92–S96†). This is compensated by a less favorable enthalpic contribution to binding (TACC3_518-532_ Δ*H* = −10.6 kJ mol^−1^ constrained variants Δ*H* ∼ −7.2–8.5 kJ mol^−1^).^9,45,46^

To further assess the thermodynamic determinants of binding, we performed variable-temperature fluorescence anisotropy experiments (Fig. S97–S98†) and Van't Hoff analyses under different conditions, specifically: buffer (Tris, HEPES), pH (pH = 6.0, 7.5 and 8.0) and salt content (NaCl = 0 mM, 150 mM). Although we observed subtle differences in absolute values of −*T*Δ*S* and Δ*H*, the observed trends were consistent with the ITC experiments (*i.e.* more favorable −*T*Δ*S* and less favorable Δ*H* for the constrained *versus* unconstrained peptide), indicating that the thermodynamic differences between unconstrained peptide and constrained peptidomimetic may be attributed to the constraint.

### 2.7 TACC3_518-532_ peptidomimetics stimulate Aurora-A activity

**Fig. 6:**
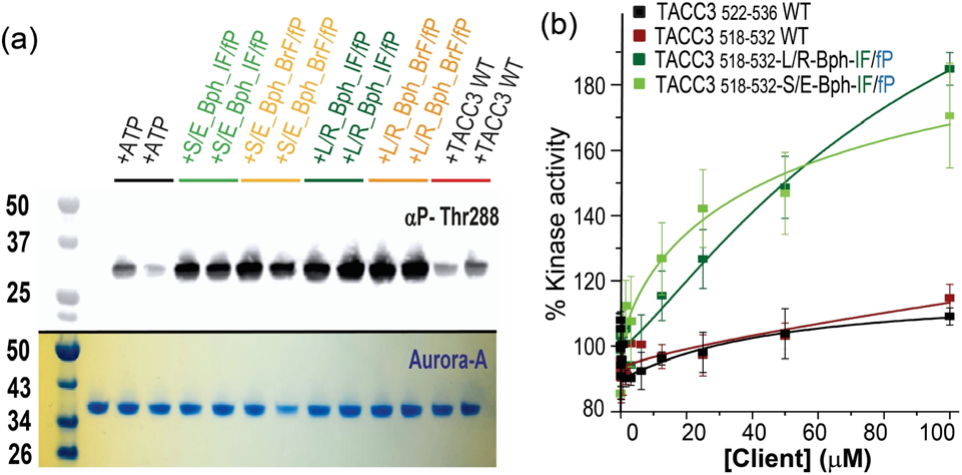
Kinase activation by peptidomimetics (a) qualitative kinase assay monitoring the autophosphorylation of unphosphorylated Aurora-A in the presence of TACC3_522-536_ and constrained peptides (all peptides 10 μM). Incubates were analyzed by SDS-PAGE (lower panel) and western-blotted with an α-phospho-T288 Aurora-A antibody (upper panel); (b) % kinase activation of Aurora-A (10 nM) in the presence of linear and constrained TACC3 peptides. ADP-Glo luminescence signal was normalized to the protein-only activity after subtracting the background signal. Shown are the means ± SD at each concentration of the peptides from duplicate experiments.

As an incomplete kinase,^26,27^ control of Aurora-A localization and activation is achieved through interaction with its intrinsically disordered clients. Indeed, TACC3 behaves as an allosteric activator of Aurora-A, whereby the protein binds to and activates the kinase, then in turn is phosphorylated, and recruited to microtubules.^27^ Such client/Aurora-A interactions cover a significant surface area on the kinase and so delineating the minimal structural determinants and changes needed to promote kinase activity is challenging. Similarly, orthosteric inhibitors of Aurora-A PPIs that simultaneously inactivate the kinase are unlikely to be differentiated from active site kinase inhibitors. We therefore assessed the effect of the peptidomimetics on Aurora-A kinase activity by performing Aurora-A autophosphorylation experiments *in vitro* (Fig. 6a). When unphosphorylated Aurora-A was incubated in the presence of the constrained peptides and ATP, they were found to stimulate Aurora-A kinase activity more effectively when compared to the WT unconstrained control peptide, with the effect being qualitatively more pronounced for the L/R constrained variants.

ADP-Glo assays provided further quantitative data on the phosphorylation activity of the protein (10 nM) in the presence of excess substrate (kemptide; 100 μM) at increasing concentrations of the linear and constrained peptides (Fig. 6b, note the acetylated peptides are used and full saturation of Aurora A may not occur at maximal peptide concentrations on the basis of the affinities determined by ITC). These experiments illustrate that both linear WT variants are relatively poor activators of the kinase, whereas the constrained peptidomimetics stimulate Aurora-A and promote substrate phosphorylation in a dose-dependent manner by up to 160–190%. A mass spectrometry kinetic assay for kemptide phosphorylation corroborated these results (Fig. S99†). Importantly, whilst TACC3_519-563_ was previously shown to activate Aurora-A,^26,27^ the effects with the shorter truncated TACC3_522-536_, and TACC3_518-532_ used here are small, implying that other features in the longer TACC3 sequence can play a role in activation of Aurora. In contrast the modified and truncated peptides (*e.g.*, TACC3_518-532-LR-Bph-IF/fP_) have a more pronounced effect in activating the kinase, unveiling a “minimal activation motif” that is enough for activation but only if binding is sufficiently potent.

### 2.8 Constrained TACC3_518-532-L/R-Bph-I/fP_ acts as an allosteric inhibitor of the N-Myc/Aurora-A interaction

**Fig. 7:**
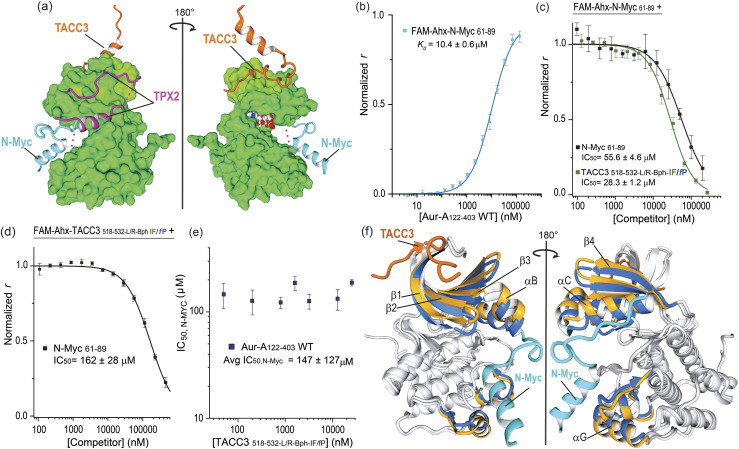
Constrained TACC3_518-532-LR-Bph-IF/fP_ as an allosteric inhibitor of N-Myc/Aurora-A PPI: (a) MUSTANG-aligned X-ray crystal structures of TACC3/Aurora-A complex (PDB: 5ODT), TPX2 (PDB: 1OL5), and N-Myc (PDB: 5G1X) showing their binding regions. For representation, only the Aurora-A molecule present in the TACC3/Aurora-A structure is shown; (b) direct FA titration of fluorescently-labeled FAM-Ahx-N-Myc_61-89_ (50 nM) with Aurora-A_122-403-C290A/C393A_; (c) FA competition assay of control N-Myc_61-89_ (black line) and constrained TACC3_518-532-LR-Bph-IF/fP_ (forest green) peptides against FAM-Ahx-N-Myc_61-89_ (200 nM) in the presence of Aurora-A_122-403-C290A/C393A_; (d) FA competition assay of N-Myc_61-89_ against fluorescently-labeled constrained FAM-Ahx-TACC3_518-532-LR-Bph-IF/fP_ (200 nM) in the presence of Aurora-A_122-403-C290A/C393A_; (e) completion FA N-Myc IC_50_'s values measured for N-Myc_61-89_ against fluorescently-labeled TACC3_518-532-LR-Bph-IF/fP_ at increased concentrations of the tracer and constant protein concentration; (f) MUSTANG-aligned X-ray crystal structures of TACC3/Aurora-A (PDB: 5ODT; TACC3 shown in orange; Aurora-A in grey) and N-Myc/Aurora-A complexes (PDB: 5G1X; N-Myc shown in blue; Aurora-A in grey). For comparison, highlighted in color are beta-sheets β1–β4 and helices αB, αC and αG in Aurora-A structures when bound to TACC3 (shown in gold) or to N-Myc (shown in cyan). All FA competition data and derived IC_50_ values are given as the means ± SD of a triplicate assay at a constant concentration of the protein (5 μM) in 25 mM Tris, 150 mM NaCl, 5 mM MgCl_2_, pH 7.5, 25 °C.

We finally assessed if the increase in potency was gained at the expense of binding pocket specificity. We therefore tested TACC3_518-532-L/R-Bph-IF/fP_ in FA competition experiments against TPX2 and N-Myc (Fig. 7). Similarly to TACC3, TPX2_1-43_ has been shown to bind Aurora-A at the N-lobe of the protein. However, it binds to the opposing face, *via* two separate segments linked by a disordered region that can interact and insert into the W, F and Y pockets on Aurora-A (Fig. 7a, TPX2_1-43_; PDB: 1OL5).^47^ On the other hand, N_Myc has been shown to bind between the N- and C-lobes to a groove adjacent to the activation loop of the kinase (N-Myc_61-89_; PDB: 5G1X).^48^ In FA competition assays, the constrained peptidomimetics exhibited limited evidence of TPX2 displacement (IC_50_ ≫ 200 μM *versus* TPX2_7-43_ IC_50,_ = 20 ± 1 μM; see Fig. S100†) indicating promising specificity for the TACC3 binding site. More surprisingly, the constrained variants were observed to out-compete N-Myc (Fig. 7c and S101†). The most potent constrained variant (TACC3_518-532-L/R-Bph-I/fP_ IC_50_ = 28 ± 1 μM) was more potent than the control N-Myc sequence (N-Myc_61-89_, IC_50_ = 56 ± 5 μM). To assess if these observations could be the consequence of orthosteric competition for the N-Myc binding site we performed the reverse experiment where acetylated N-Myc_61-89_ was employed to displace fluorescently-labeled TACC-3_518-532-L/R-Bph-I/fP_ (Fig. 7d). This N-Myc sequence was also observed to out-compete the TACC3 peptide.

An allosteric mode of inhibition is characterized by the competitor binding at a separate site from that of the “substrate–ligand”, and showing similar affinity for the target regardless of the presence of the substrate-ligand.^49–51^ Under this model the dose/response IC_50_ values observed for a competitor should remain constant regardless of the ligand concentration. To probe further the role of allostery, we performed FA competition assays at varying concentrations of FAM-labelled TACC3_518-532-L/R-Bph-IF/fP_ (0.05–25.6 μM, Fig. S102†); performing the opposing experiment *i.e.* displacing varying concentrations of FAM-N-Myc_61-89,_ was not practical due to the significantly weaker binding of N-Myc for Aurora-A. The N-Myc IC_50_ values at each tracer concentration did not vary markedly (IC_50_ ∼ 120–160) μM (Fig. 7e) confirming an allosteric mode of inhibition.

Analysis of the crystal structures reported for Aurora-A in the presence of TACC3 and N-Myc offers a rationale for the allosteric inhibition. Numerous, conformational changes in the protein upon binding each substrate are observed (Fig. 7f). The N-lobe sheets β1–β3 and helices α_B_–α_C_ show a significant deviation in their relative positions between both structures, and the deviation appears to be conformationally transmitted to αG helix in the C-lobe, which is directly involved in N-Myc binding. The displacement in β1-β3 and helices αB–αC in the TACC3/Aurora-A structure, when compared to that of N-Myc, was also observed in a recently reported CEP192/Aurora-A structure. Notably, binding of CEP192 only to the TACC3 site on Aurora-A stimulates kinase activity, consistent with a conserved function through a similar allosteric mechanism.^52^

## 3 Conclusions

**Fig. 8:**
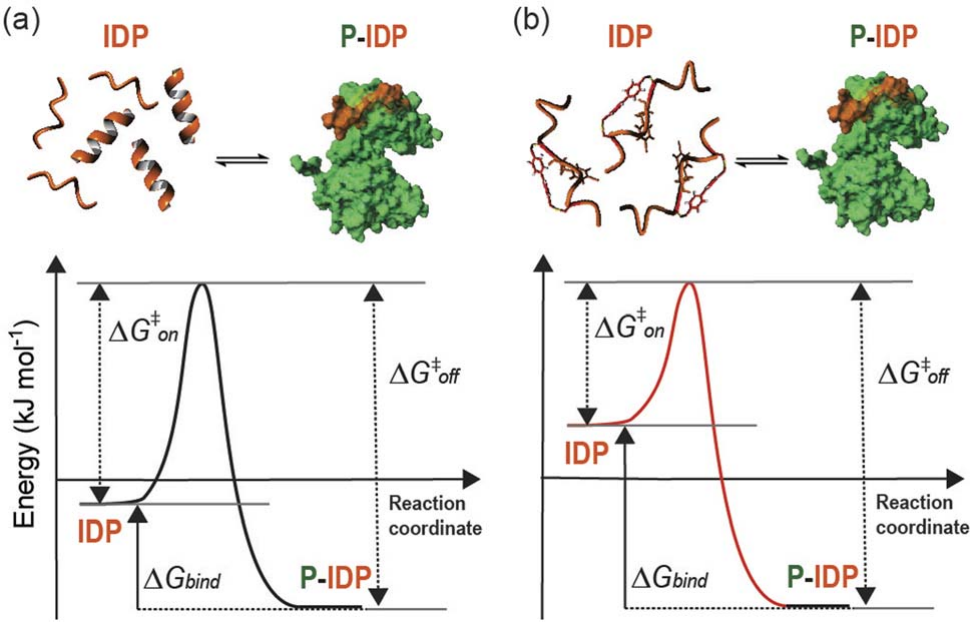
Thermodynamics of binding in the presence of Aurora-A. Diagram schematically illustrating the hypothetical free energy profile of a one-step/one-barrier peptide-protein binding event for a linear (a) and a constrained peptide (b).

Herein we have described a rational approach to the development of competitive peptidomimetic inhibitors, *e.g.* TACC3_518-532-L/R-Bph-IF/fP_, of the TACC3/Aurora-A interaction. Key to the success of this approach was the incorporation of a constraint within the peptide despite the absence of a well-defined secondary structure at the interface. Whilst a structurally well-defined interaction between TACC3/Aurora-A occurs and must be replicated in large part by the peptidomimetic, the unbound form of both the peptide and the constrained peptidomimetic were shown to be largely disordered. The enhanced affinity of the constrained peptidomimetic arises from a restricted conformational space, in effect raising the energy of the ensemble and resulting in a more favourable entropy of binding to the kinase (Fig. 8). Related effects have been observed whereby subtle changes to a 14-3-3 binding macrocycle can bias a conformational ensemble towards the bound conformation as demonstrated through free energy perturbation and molecular dynamics analyses.^53^ The behavior observed here is also similar to that recently described to occur upon methylation of backbone amides for a ligand that recognizes its target through β-strand complementation.^10^ Raising the energy of the unbound ensemble can offer a general route to enhancing target binding affinity as opposed to preorganizing a ligand in its bound conformation – a difficult prospect given PPIs occur as partially bound states.^46^ Promisingly, the high affinity of the most potent peptidomimetic TACC3_518-532-L/R-Bph-IF/fP_ opens up new opportunities to understand and allosterically regulate the function and interactions of Aurora-A. A number of Aurora-A interactors *e.g.* TACC3 (ref. 26) and TPX2 (ref. 54) activate the kinase, despite binding at different sites, whilst paradoxically CEP192,^55^ which binds at both TACC3 and TPX2 sites, suppresses kinase activity. All three proteins may participate in additional non-covalent interaction with the kinase beyond that revealed by their X-ray structures, complicating an interpretation of the minimal determinants for allosteric kinase modulation. In contrast our minimal peptidomimetics of only thirteen residues are sufficient to induce conformational changes needed to activate the kinase by binding only a single pocket on the N-lobe. This “minimal activation motif” relies on the more potent binding of the peptidomimetic in contrast to the native sequence.

The more potent peptidomimetic also allosterically displaces N-Myc from its binding site and does so more effectively that the native sequence; this allosteric displacement is likely linked by common structural changes that lead to kinase activation. Inhibition of the N-Myc/Aurora-A interaction^39^ may represent a target for anticancer drug-development – a number of active-site ligands have been shown to allosterically displace N-Myc,^48,56^ but may prove problematic as a consequence of downregulating the essential functions of Aurora-A.^57,58^ Being able to orthosterically inhibit the TACC3/Aurora-A interaction and allosterically inhibit N-Myc/Aurora-A without inhibiting kinase activity may thus represent a promising alternative to these active-site ligands. Further efforts towards this goal would need to establish the extent to which allosteric N-Myc displacement and Aurora-A activation can be balanced. Thus, these peptidomimetic tools inform on allosteric regulation in Aurora A and reveal new opportunities for chemical probe development.

## Data availability

All relevant data are included in the ESI.†

## Author contributions

A. J. W. conceived and designed the research program, with the input of M. H. W and R. B.; D. G. designed the studies and performed the research, with support from M. W. J. A. M. performed the kinase autophosphorylation assays *in vitro* and contributed to protein production. A. J. W and D. G. wrote the manuscript with contributions from all authors.

## Conflicts of interest

There are no conflicts to declare.

## Supplementary Material

SC-OLF-D4SC06100D-s001

SC-OLF-D4SC06100D-s002
