# Supplementary material for: Constrained TACC3 peptidomimetics for a non-canonical protein–protein interface elucidate allosteric communication in Aurora-A kinase
Source: Chem Sci. 2024 Nov 28;16(1):354–63. doi: 10.1039/d4sc06100d (PMC11604048; doi:10.1039/d4sc06100d)
Supplement: SC-016-D4SC06100D-s002 [file SC-016-D4SC06100D-s002.pdf]

Supplementary Information (SI) for Chemical Science.
This journal is © The Royal Society of Chemistry 2024

Constrained TACC3 peptidomimetics for a non-canonical protein-protein interface elucidate allosteric communication in Aurora-A Kinase

Diana Gimenez,[a] Martin Walko,[b] Jennifer A. Miles,[c, d] Richard Bayliss,[c, d] Megan H. Wright[b, d] and Andrew J. Wilson[a, b, d]

[a] *School of Chemistry, University of Birmingham, Edgbaston, Birmingham B15 2TT, UK.*

[b] *School of Chemistry, University of Leeds, Woodhouse Lane, Leeds LS2 9JT, UK.*

[c] *School of Molecular and Cellular Biology, University of Leeds, Woodhouse Lane, Leeds LS2 9JT, UK*

[d] *Astbury Centre for Structural Molecular Biology, University of Leeds, Woodhouse Lane, Leeds LS2 9JT, UK.*

Contents

Supplementary data and figures....4

"In silico" alanine scan analysis of TACC3 $_{522\text{-}536}$....4

TACC3 $_{522\text{-}536}$ Phe525 scan....5

MD analysis of WT TACC3 $_{522\text{-}536}$ interaction with Aurora-A....7

MD analysis of TACC3$_{522\text{-}536(4\text{-}I)Phe525}$ interaction with Aurora-A....10

TACC3 $_{522\text{-}536}$ Pro528 scan and Val531/Leu532 variants....13

MD analysis of TACC3$_{522\text{-}536\text{-}\textit{trans}\text{-}(4\text{-}F)Pro528}$ interaction with Aurora-A....14

TACC3 $_{522\text{-}536}$ systematic constraint scan....17

TACC3 $_{522\text{-}536}$ sequence optimization....19

MD analysis of WT TACC3 $_{518\text{-}532}$ interaction with Aurora-A....21

TACC3 $_{518\text{-}532}$ (*i*, *i+6*) constrained variants....23

MD analysis of biphenyl- constrained TACC3 $_{518\text{-}532\text{-}L/R\text{-}Bph}$ interaction with Aurora-A....25

MD analysis of biphenyl- constrained TACC3 $_{518\text{-}532\text{-}S/E\text{-}Bph}$ interaction with Aurora-A....26

TACC3$_{518\text{-}532}$ Bph- constrained/Phe525 halogen-substituted variants....27

TACC3$_{518\text{-}532}$ Bph- constrained/Phe525 halogen-substituted/ (4-F)Pro528 variants....28

MD analysis of constrained TACC3 $_{518\text{-}532\text{-}L/R\text{-}Bph\text{-}IF/fP}$ interaction with Aurora-A....30

MD trajectory analysis of constrained TACC3 $_{518\text{-}532\text{-}S/E\text{-}Bph\text{-}IF/fP}$ interaction with Aurora-A....32

Enzymatic stability of Peptidomimetics....36

NMR analysis: TACC3 $_{518\text{-}532}$....43

NMR Peptide secondary structure in solution: Secondary $\Delta\delta_{C\alpha}$, $\Delta\delta_{H\alpha}$ and $\Delta\delta_{NH}$ Chemical Shifts (SCS)...49

NMR analysis: TACC3$_{518\text{-}532}$ Leu520Cys/Arg526Cys Bph constrained (4-I)Phe525/*trans*-(4-F)Pro528 variant 52

^{1}H (VT)-NMR of TACC3$_{518-532}$ Leu520Cys/Arg526Cys Bph- constrained (4-I)Phe525/*trans*-(4-F)Pro528 variant 58

^{1}H-(VT)-NMR: Conformational preference at Pro528 62

NMR analysis: Control TACC3 $_{518-532}$ Leu520Cys/Arg526Cys Bph constrained variant 64

NMR analysis: Control TACC3 $_{518-532}$ Ser524Cys/Glu530Cys Bph constrained variant 71

NMR analysis: TACC3 $_{518-532}$ Ser524Cys/Glu530Cys Bph constrained (4-I)Phe525/*trans*-(4-F)Pro528 variant 78

^{1}H (VT)-NMR of TACC3$_{518-532}$ Ser524Cys/Glu520Cys Bph- constrained (4-I)Phe525/*trans*-(4-F) Pro528 variant 85

Isothermal titration calorimetry (ITC) 87

Variable temperature fluorescence anisotropy titrations (VT-FA) 91

Kinetic Analyses of Kinase Activity 93

FA competition assays in the presence of FAM-Ahx-TPX2 $_{7-47}$ 93

FA competition assays in the presence of FAM-Ahx-N-Myc$_{61-89}$ 94

Allosteric inhibition: N-Myc $_{61-89}$ FA competition assays in the presence of FAM-Ahx- TACC3 $_{518-532-L/R-Bph-IF/fP}$ 96

Materials and methods 98

Abbreviations 105

References 105

Summary of peptide characterization 106

Analytical HPLC traces and high-resolution mass spectra of synthesised peptides 108

Supplementary data and figures

"In silico" alanine scan analysis of TACC3 $_{522\text{-}536}$

-TACC3 $_{522\text{-}536}$ WT-

		522 Glu	523 Glu	524 Ser	**525 Phe**	526 Arg	527 Asp	**528 Pro**	529 Ala	530 Glu	**531 Val**	**532 Leu**	533 Ala	534 Thr	Gly 535	Ala 536	537 Glu
**PPCheck**	Total energy of complex (kJ/mol)	0	60.6	0.12	**35.7**	23.9	33.8	**10.8**	0	8.2	**8.02**	**17.7**	0	9.6	0	0	0
	Number of interface residues	0	0	0	**3**	0	0	**1**	0	1	**0**	**0**	0	0	0	0	0
	Normalized energy per residue (kJ/mol)	0	0.21	0.01	**-0.01**	0.09	0.12	**0**	0	0.01	**0.03**	**0.06**	0	0.04	0	0	0
**BUDE AS**	ΔΔG (kJ/mol)	0	3.4	0	**25.3**	0.8	2.5	**4.8**	0	0	**3.6**	**8.2**	0	0.1	0	0	0

Table S1. *In silico* TACC3 $_{522\text{-}536}$ alanine scan. Virtual alanine scan results for the Aurora-A/TACC3$_{522\text{-}536}$ interaction using PPCheck[1] (http://caps.ncbs.res.in/ppcheck/) and BUDE alanine Scan[2] (https://pragmaticproteindesign.bio.ed.ac.uk/balas/). In each case non-charged hot-spot residues are highlighted in red.

TACC3 $_{522\text{-}536}$ Phe525 scan

Peptide		Sequence[a]		IC_{50}[b] (µM)
**TACC3 $_{522\text{-}536}$**	**Ac-**	**EES [525]F RDPAEVLGTGA**	**$-NH_2$**	**163 ± 13**
TACC3 $_{522\text{-}536}$	Ac-	EES **[525](4-F)F** RDPAEVLGTGA	$-NH_2$	184 ± 22
TACC3 $_{522\text{-}536}$	Ac-	EES **[525](4-Cl)F** RDPAEVLGTGA	$-NH_2$	46 ± 8
TACC3 $_{522\text{-}536}$	Ac-	EES **[525](4-Br)** RDPAEVLGTGA	$-NH_2$	34 ± 6
TACC3 $_{522\text{-}536}$	Ac-	EES **[525](4-I)F** RDPAEVLGTGA	$-NH_2$	34 ± 3
TACC3 $_{522\text{-}536}$	Ac-	EES **[525](4-CF_3)F** RDPAEVLGTGA	$-NH_2$	170 ± 17
TACC3 $_{522\text{-}536}$	Ac-	EES **[525](4-CN)F** RDPAEVLGTGA	$-NH_2$	138 ± 8
TACC3 $_{522\text{-}536}$	Ac-	EES **[525]Tyr** RDPAEVLGTGA	$-NH_2$	≥ 500
TACC3 $_{522\text{-}536}$	Ac-	EES **[525]Tyr(OMe)** RDPAEVLGTGA	$-NH_2$	≥ 300
TACC3 $_{522\text{-}536}$	Ac-	EES **[525](*5f*)F** RDPAEVLGTGA	$-NH_2$	97 ± 10
TACC3 $_{522\text{-}536}$	Ac-	EES **[525](*p*-Bnz)F** RDPAEVLGTGA	$-NH_2$	≥ 500
TACC3 $_{522\text{-}536}$	Ac-	EES **[525](*α*-Me)F** RDPAEVLGTGA	$-NH_2$	≥ 500

Table S2. Systematic TACC3 $_{522\text{-}536}$ Phe525 scan. [a] One letter code for amino acids. [b] IC_{50} values given as the mean value and corresponding standard deviation (SD) determined from triplicate competition FA assays against fluorescein labeled Fam-Ahx-TACC3$_{522\text{-}536}$ (200 nM) in the presence of Aurora-A$_{122\text{-}403\text{-}C290A/C393A}$ (5 µM)(n= 3). All assays were performed in 25 mM Tris, 150 mM NaCl, 5 mM $MgCl_2$, pH= 7.5.

-

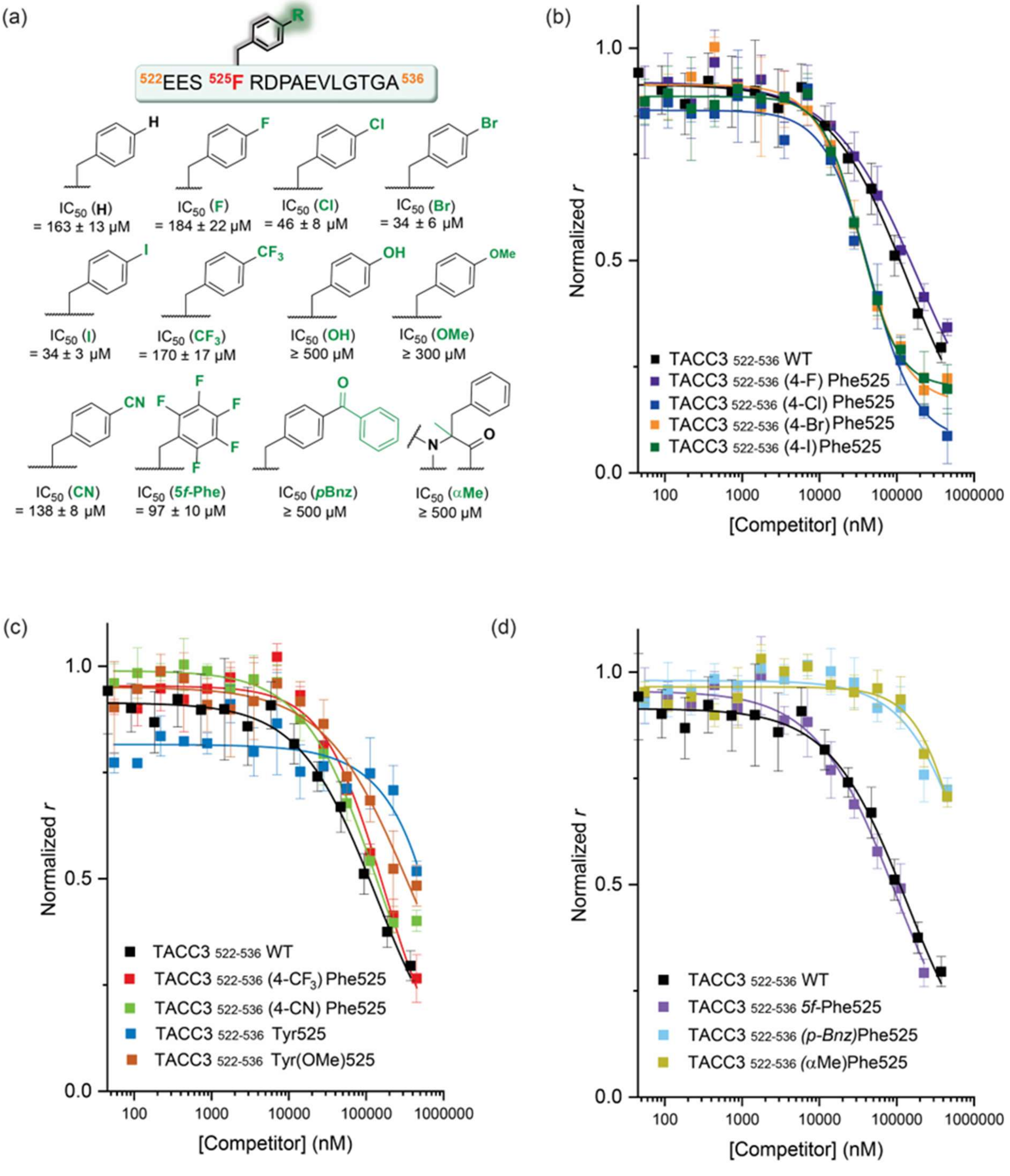

Figure S1. Competition FA results for peptides in Table 2. (25 mM Tris, 150 mM NaCl, 5 mM $MgCl_2$, pH= 7.5, 5 µM Aurora-A $_{122\text{-}403\text{-}C290A/C393A}$, 200 nM Fam-Ahx-TACC3$_{522\text{-}536}$, 25 °C).

MD analysis of WT TACC3 $_{522\text{-}536}$ interaction with Aurora-A

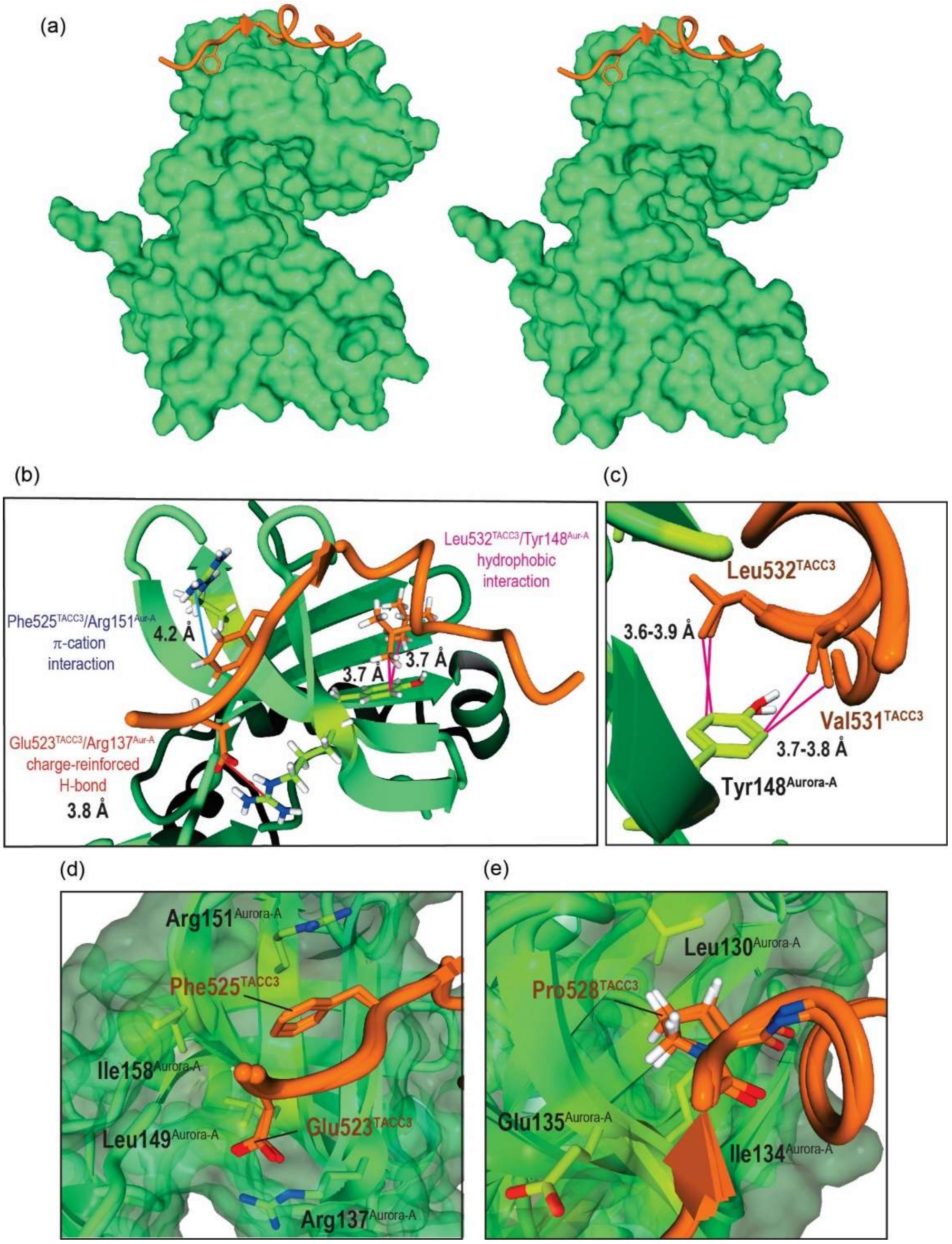

Figure S2. (a) Replicate energy minimized structure of Aurora-A$_{122\text{–}403\ C290A/C393A/D274N}$ /TACC3 $_{522\text{-}536}$ complex after 150 ns of MD simulation (600 frames); (b) showing key interactions; (c) Inset showing the two possible hydrophobic contacts measured in the minimum energy structures between Tyr148$^{Aurora\text{-}A}$ and residues Val531, and Leu532 in TACC3 (d) Inset showing the accommodation of Phe525^{TACC3} in its binding pocket, with the aromatic ring orientated towards the N-terminus. (e) Inset showing the accommodation and Cγ-*exo*-pucker conformation of the Pro528 ^{TACC3} residue in its binding pocket, which favors a *trans*-amide configuration of the peptide bond.

MD trajectory analyses were also employed to explore how the secondary structure of the peptide evolves with time at each specific residue. As shown in Fig. S3, the peptide is predicted to remain largely unstructured in the presence of the protein, with only residues 527-532 showing some propensity to organize into a transient α-helical secondary structure.

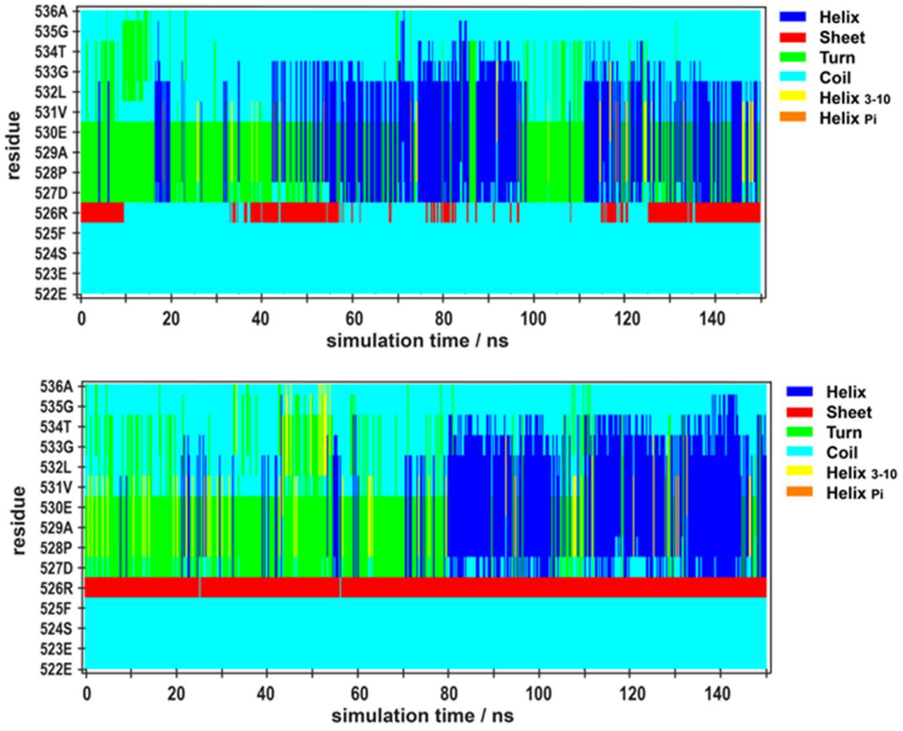

Figure S3. Replicate MD simulations of bound TACC3$_{522-536}$ WT in complex with Aurora-A$_{122–403-C290A/C393A/D274N}$, showing TACC3$_{522-536}$ per-residue secondary structure.

In addition, MD simulations were also employed to ascertain which residues within the peptide are estimated to show a higher number of sustained statistical contacts with Aurora-A, thus potentially acting as non-covalent contacts between TACC3 and Aurora-A (Fig S4, represents the number of contacts to the protein calculated for each of the peptide residues during consecutive time-intervals of 2.50 fs). Qualitatively, MD analysis agrees with results from "*in silico*" alanine scanning in that Phe525^{TACC3}, Pro528^{TACC3}, Val531^{TACC3}, and Leu532^{TACC3} are important for binding, showing a higher number of contacts to the protein during the simulation. Arg526^{TACC3} and Glu530^{TACC3} also seem to be transient contributors to binding, if modestly. Notably, Glu523^{TACC3} which was expected to show a significant number of contacts to the protein was predicted to be a relatively poor contributor, implying that the observed charge-reinforced hydrogen-bond between Glu523^{TACC3} and ^{151}Arg151$^{Aurora-A}$ observed in the crystal structure might be more transient in nature.

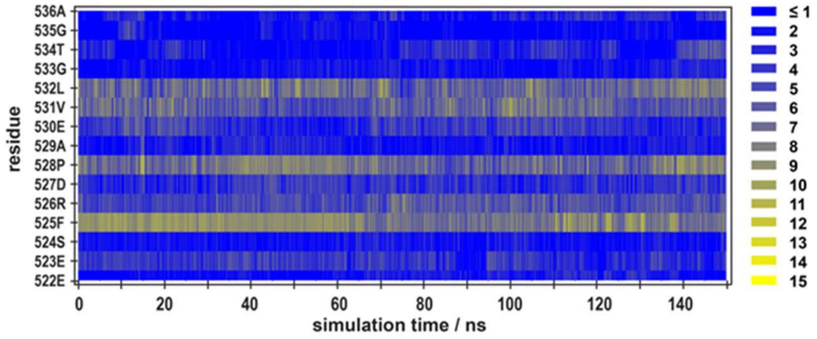

Per-residue average number of contacts per frame (2.5 fs; n= 600)															
Residue	522	523	524	525	526	527	528	529	530	531	532	533	534	535	536
Avg. number of contacts	1.9	3.7	1.9	8.2	5.2	3.6	7.1	2.2	3.8	6.2	6.8	1.7	2.4	1.2	2.2

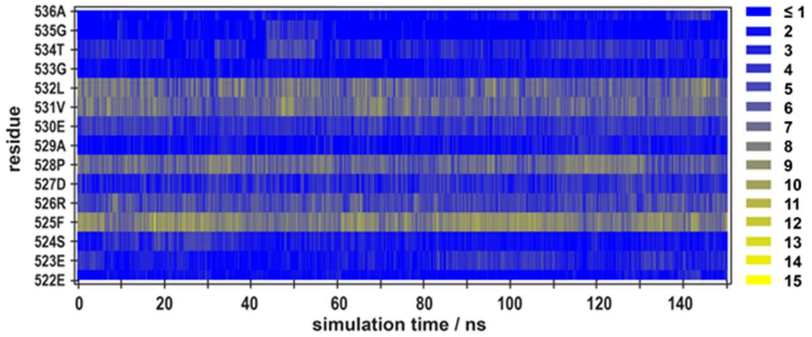

Per-residue average number of contacts per frame (2.5 fs; n= 600)															
Residue	522	523	524	525	526	527	528	529	530	531	532	533	534	535	536
Avg. number of contacts	1.4	2.6	2.4	8.2	5.1	3.3	6.7	1.9	3.8	6.7	6.7	1.5	3.0	1.1	1.4

Figure S4. Replicate MD simulations of bound TACC3$_{522\text{-}536}$ WT in complex with Aurora-A$_{122–403\text{-}C290A/C393A/D274N}$, showing TACC3$_{522\text{-}536}$ per-residue number of contacts to the protein.

MD analysis of TACC3$_{522\text{-}536(4\text{-}I)Phe525}$ interaction with Aurora-A

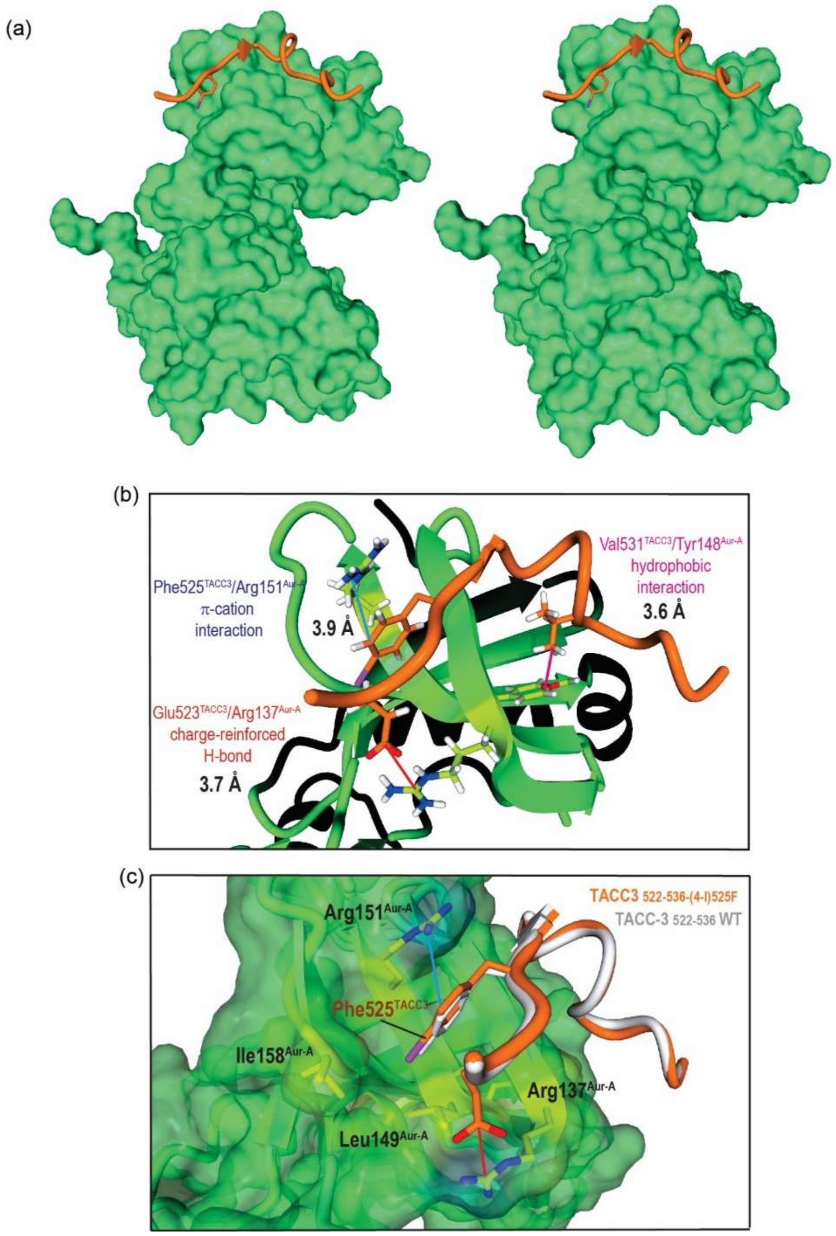

Figure S5. (a) Replicate energy minimized structure of Aurora-A$_{122\text{–}403\text{-}C290A/C393A/D274N}$/TACC3$_{522\text{-}536\text{-}(4\text{-}I)Phe525}$ complex after 150 ns of MD simulation (600 frames); (b) Inset showing the accommodation and key interactions of TACC3$_{522\text{-}536\text{-}(4\text{-}I)Phe525}$ in its binding pocket. Here a stronger hydrophobic contact is observed between Tyr148$^{Aurora\text{-}A}$ and Val531^{TACC3} (3.6 Å), rather than with Leu532^{TACC3} (3.8 Å), as observed in the WT sequence; (c) For comparison, superimposed MD minimum energy structures of control TACC3$_{522\text{-}536}$ WT (shown in grey) and TACC3$_{522\text{-}536\text{-}(4\text{-}I)Phe525}$ (shown in orange), showing an apparent better insertion of the iodinated (4-I)Phe525 residue in its hydrophobic pocket on Aurora-A.

Trajectory analyses of the peptide secondary structure per residue for this variant in the presence of Aurora-A suggest only transient organization, as seen for the control TACC3$_{522\text{-}536}$ peptide (Fig. S6). However, when compared, a small but significant increase in the calculated number of contacts per residue (Fig. S7) is predicted for the iodo-substituted (4-I)Phe525 residue, from an average of 8.2 contacts per segment of 2.5 fs to 8.6 - 8.8 contacts per frame, which is indicative of a deeper insertion into its hydrophobic pocket on Aurora-A .

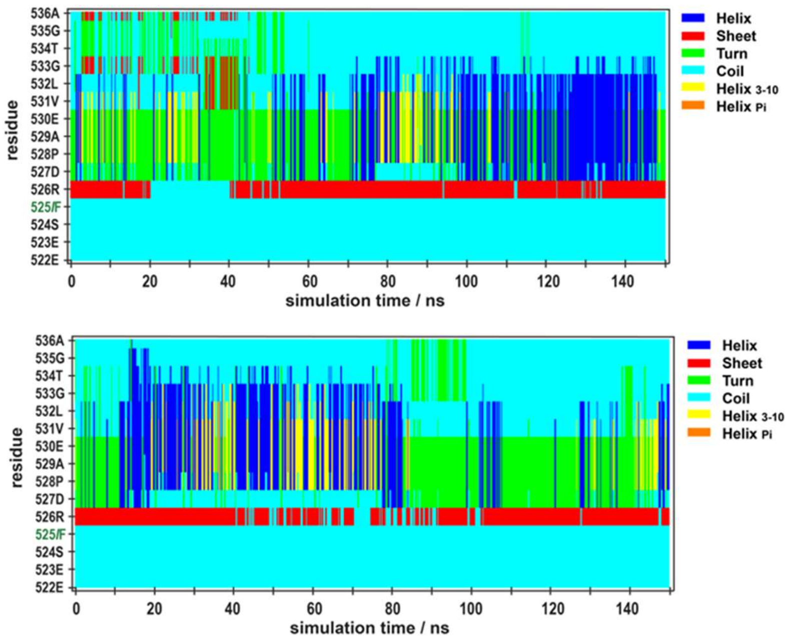

Figure S6. Replicate MD simulations of bound TACC3$_{522\text{-}536\text{-}(4\text{-}I)Phe525}$ in complex with Aurora-A$_{122–403\text{-}C290A/C393A/D274N}$, showing TACC3$_{522\text{-}536\text{-}(4\text{-}I)Phe525}$ per-residue secondary structure.

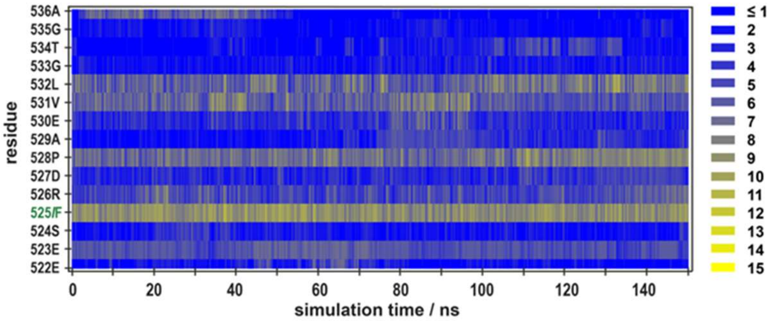

Per-residue average number of contacts per frame (2.5 fs; n= 600)															
Residue	522	523	524	525	526	527	528	529	530	531	532	533	534	535	536
Avg. number of contacts	1.9	5.5	2.2	8.8	5.0	3.4	7.3	2.7	3.6	6.3	6.7	2.0	2.0	1.4	2.4

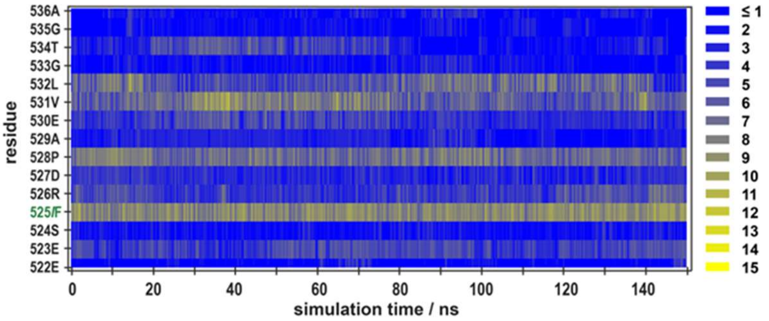

Per-residue average number of contacts per frame (2.5 fs; n= 600)															
Residue	522	523	524	525	526	527	528	529	530	531	532	533	534	535	536
Avg. number of contacts	1.0	4.5	1.8	8.6	4.5	3.2	7.1	2.3	4.1	6.8	6.0	1.6	3.0	1.3	1.6

Figure S7. Replicate MD simulations of bound TACC3$_{522\text{-}536\text{-}(4\text{-}I)Phe525}$ in complex with Aurora-A$_{122–403\text{-}C290A/C393A/D274N}$, showing TACC3$_{522\text{-}536\text{-}(4\text{-}I)Phe525}$ per-residue number of contacts to the protein.

TACC3 $_{522-536}$ Pro528 scan and Val531/Leu532 variants

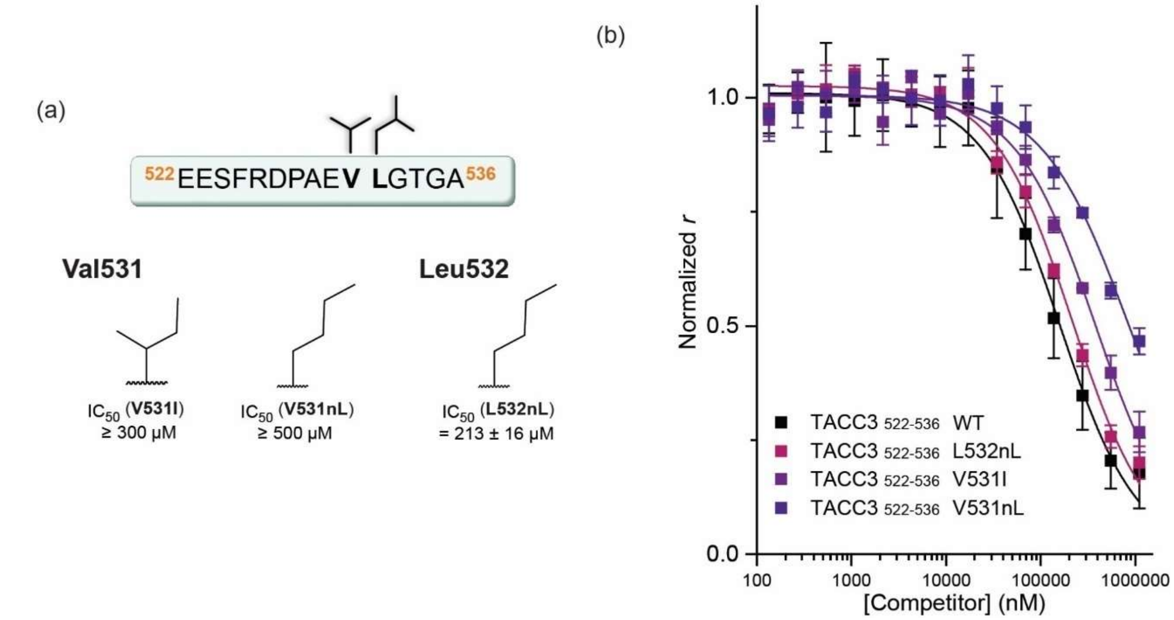

Figure S8. Competition FA results for Val531 and Leu532 variants. (25 mM Tris, 150 mM NaCl, 5 mM $MgCl_2$, pH= 7.5, 5 µM Aurora-A$_{122–403-C290A/C393A}$, 200 nM FAM-Ahx-TACC3$_{522-536}$, 25 °C).

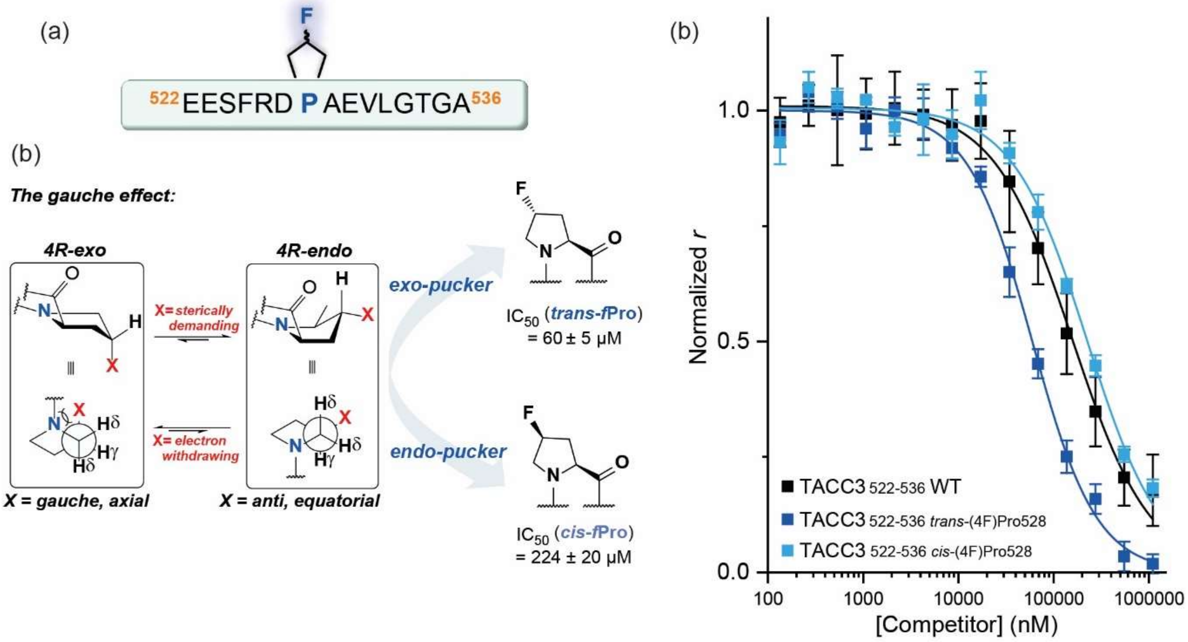

Figure S9. Competition FA results for (4-F)Pro528 substituted TACC3 peptides. (25 mM Tris, 150 mM NaCl, 5 mM $MgCl_2$, pH= 7.5, 5 µM Aurora-A$_{122–403-C290A/C393A}$, 200 nM FAM-Ahx-TACC3$_{522-536}$, 25 °C)

MD analysis of TACC3$_{522\text{-}536\text{-}trans\text{-}(4\text{-}F)Pro528}$ interaction with Aurora-A

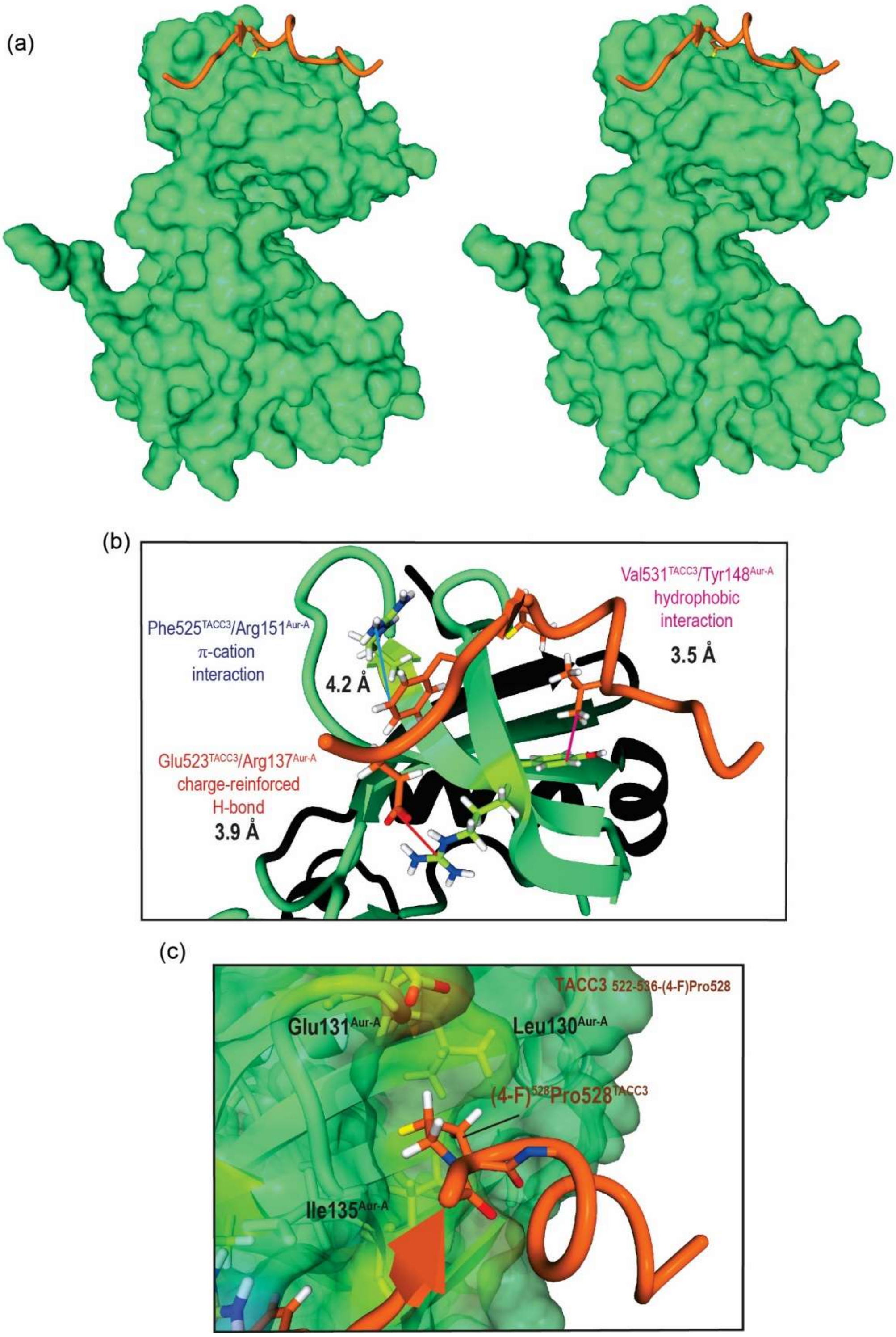

Figure S10. (a) Energy minimized structure of Aurora-A$_{122–403\text{-}C290A/C393A/D274N}$ /TACC3$_{522\text{-}536\text{-}trans\text{-}(4\text{-}F)Pro528}$ complex after 150 ns of MD simulation (600 frames). (b) Inset showing the accommodation and key interactions of TACC3$_{522\text{-}536\text{-}trans\text{-}(4\text{-}F)Pro528}$ in its binding pocket. (c) Inset showing the accommodation and *exo*-pucker conformation of (4-F)Pro528^{TACC3} in its binding pocket.

Consistent with previous trajectory analyses of the peptide secondary structure in the presence of Aurora-A, this variant exhibits only transient organization, as seen for the control TACC3$_{522-536}$ peptide (Fig. S11). However, and as observed previously for the iodo-substituted (4-I)Phe525 residue, replacement of the Pro528 residue by *trans*-(4f)Pro528 increased modestly the average number of contacts to Aurora-A at this position, from an average of 6.7 - 7.1 contacts per segment of 2.5 fs to 7.6 - 8.1 contacts, which is indicative of deeper insertion of this residue into its pocket on Aurora-A .

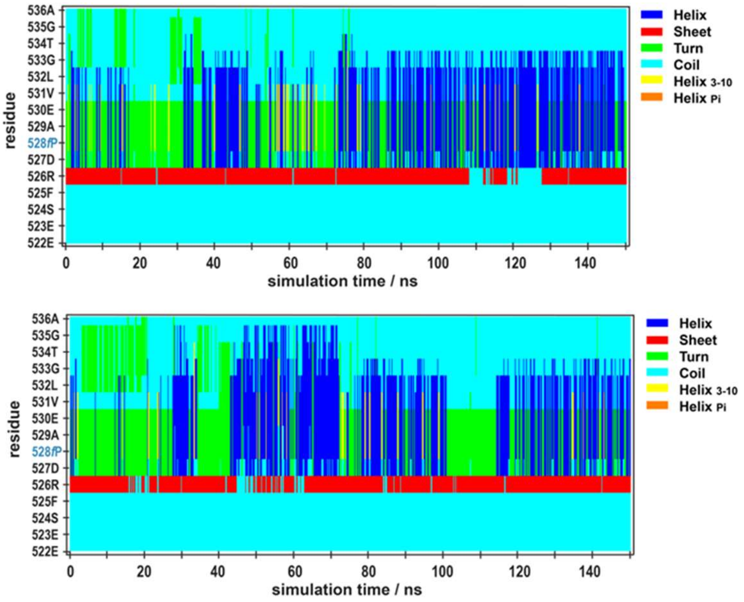

Figure S11. Replicate MD simulations of bound TACC3 $_{522-536-\textit{trans}-(4-F)Pro528}$ in complex with Aurora-A $_{122–403\ C290A/C393A/D274N}$, showing TACC3 $_{522-536-\textit{trans}-(4-F)Pro528}$ per-residue secondary structure.

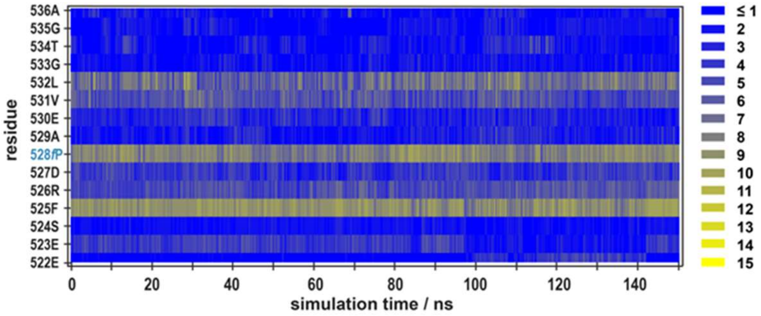

Per-residue average number of contacts per frame (2.5 fs; n= 600)															
Residue	522	523	524	525	526	527	528	529	530	531	532	533	534	535	536
Avg. number of contacts	1.6	3.5	1.7	8.9	5.2	3.8	8.1	2.3	2.9	5.5	6.9	1.7	1.8	1.5	1.6

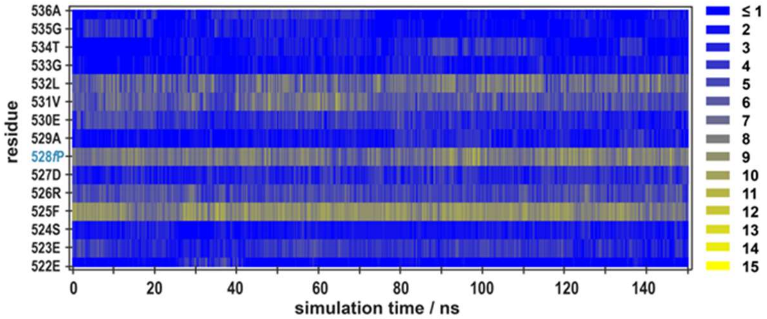

Per-residue average number of contacts per frame (2.5 fs; n= 600)															
Residue	522	523	524	525	526	527	528	529	530	531	532	533	534	535	536
Avg. number of contacts	1.1	3.6	2.0	8.7	5.0	3.1	7.6	2.1	3.5	6.1	6.8	1.8	2.1	1.9	1.9

Figure S12. Replicate MD simulations of bound TACC3$_{522\text{-}536\text{-}\textit{trans}\text{-(4-F)Pro528}}$ in complex with Aurora-A$_{122\text{–}403\text{-C290A/C393A/D274N}}$, showing TACC3$_{522\text{-}536\text{-}\textit{trans}\text{-(4-F)Pro528}}$ per-residue number of contacts to the protein.

TACC3 $_{522-536}$ systematic constraint scan

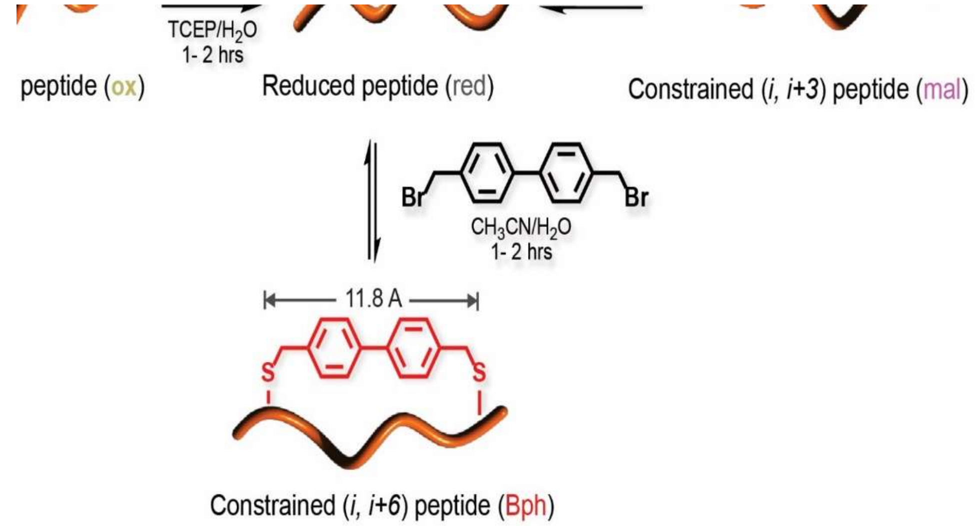

Peptide	Sequence[a]			(ox)	(red)	(mal)	(Bph)
				IC$_{50}$[b] (μM)			
**TACC3 $_{522-536}$[c]**	**Ac-**	**EESFRDPAEVLGTGA**	**-NH$_2$**	**114 ± 16**	**176± 27**	**183 ± 21**	**183 ± 21**
TACC3 $_{522-536\ E523C/R526C}$	Ac-	ECSFCDPAEVLGTGA	-NH$_2$	≥500	≥500	376 ± 28	
TACC3 $_{522-536\ S524C/D527C}$	Ac-	EECFRCPAEVLGTGA	-NH$_2$	≥500	309 ± 38	≥500	
TACC3 $_{522-536\ F525C/P528C}$	Ac-	EESCRDCAEVLGTGA	-NH$_2$	≥500	≥500	244 ± 28	
TACC3 $_{522-536\ R526C/A529C}$	Ac-	EESFCDPCEVLGTGA	-NH$_2$	≥500	352 ± 45	≥500	
TACC3 $_{522-536\ D527C/E530C}$	Ac-	EESFRCPACVLGTGA	-NH$_2$	≥500	≥500	186 ± 50	
TACC3 $_{522-536\ P528C/V531C}$	Ac-	EESFRDCAECLGTGA	-NH$_2$	≥500	≥500	≥500	
TACC3 $_{522-536\ A529C/L532C}$	Ac-	EESFRDPCEVCGTGA	-NH$_2$	≥500	≥500	483 ± 58	
TACC3 $_{522-536\ E530C/G533C}$	Ac-	EESFRDPACVLCTGA	-NH$_2$	≥500	≥500	223 ± 17	
TACC3 $_{522-536\ V531C/T534C}$	Ac-	EESFRDPAECLGCGA	-NH$_2$	≥500	≥500	412 ± 73	
TACC3 $_{522-536\ L532C/G535C}$	Ac-	EESFRDPAEVCGTCA	-NH$_2$	≥500	≥500	480 ± 60	
TACC3 $_{522-536\ G533C/A536C}$	Ac-	EESFRDPAEVLCTGC	-NH$_2$	≥500	80 ± 3	≥500	
TACC3 $_{522-536\ R526C/V531C}$	Ac-	EESFCDPAECLGTGA	-NH$_2$	≥500	≥500		249 ± 43
TACC3 $_{522-536\ E522C/P528C}$	Ac-	CESFRDCAEVLGTGA	-NH$_2$	≥500	≥500		280 ± 32
TACC3 $_{522-536\ A529C/G535C}$	Ac-	EESFRDPCEVLGTCA	-NH$_2$	77 ± 12	118 ± 26		151 ± 15

Table S3. TACC3$_{522-536}$ (*i*, *i* + 3) constraint. [a] One letter code for amino acids. [b] IC$_{50}$ values given as the mean value and corresponding standard deviation (SD) determined from triplicate competition FA assays against fluorescein labeled FAM-Ahx-TACC3$_{522-536}$ (200 nM) in the presence of Aurora-A$_{122-403-C290A/C393A}$ (5 μM)(n= 3). [c] Values given for individual replica control assays, with TACC3$_{522-536}$ subject to the same DMSO incubation procedure as used for other variants in the case of oxidized species (see supporting methods). All experiments were performed in 25 mM Tris, 150 mM NaCl, 5 mM $MgCl_2$, pH= 7.5.

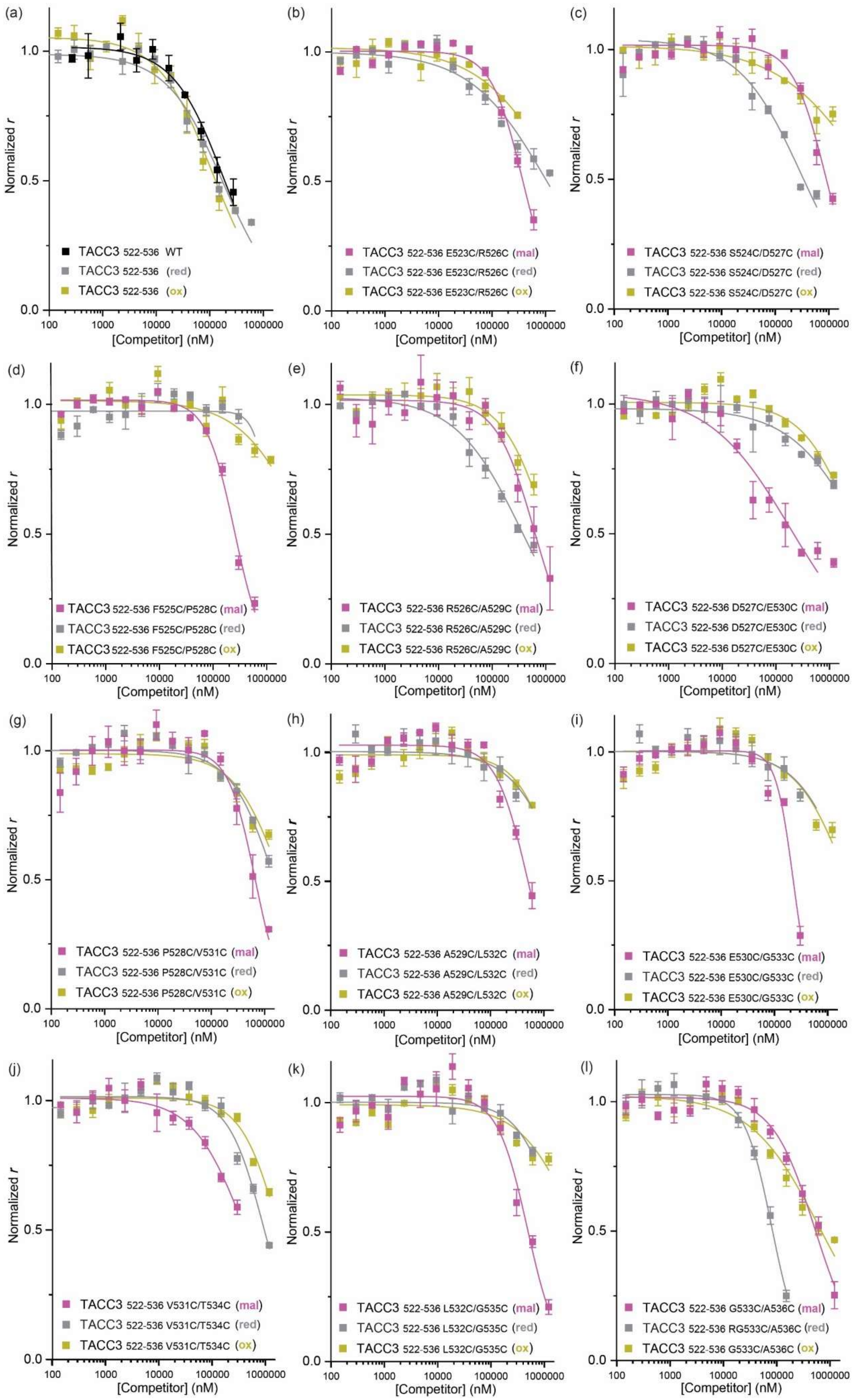
(a)
TACC3 522-536 WT
TACC3 522-536 (red)
TACC3 522-536 (ox)
(b)
TACC3 522-536 E523C/R526C (mal)
TACC3 522-536 E523C/R526C (red)
TACC3 522-536 E523C/R526C (ox)
(c)
TACC3 522-536 S524C/D527C (mal)
TACC3 522-536 S524C/D527C (red)
TACC3 522-536 S524C/D527C (ox)
(d)
TACC3 522-536 F525C/P528C (mal)
TACC3 522-536 F525C/P528C (red)
TACC3 522-536 F525C/P528C (ox)
(e)
TACC3 522-536 R526C/A529C (mal)
TACC3 522-536 R526C/A529C (red)
TACC3 522-536 R526C/A529C (ox)
(f)
TACC3 522-536 D527C/E530C (mal)
TACC3 522-536 D527C/E530C (red)
TACC3 522-536 D527C/E530C (ox)
(g)
TACC3 522-536 P528C/V531C (mal)
TACC3 522-536 P528C/V531C (red)
TACC3 522-536 P528C/V531C (ox)
(h)
TACC3 522-536 A529C/L532C (mal)
TACC3 522-536 A529C/L532C (red)
TACC3 522-536 A529C/L532C (ox)
(i)
TACC3 522-536 E530C/G533C (mal)
TACC3 522-536 E530C/G533C (red)
TACC3 522-536 E530C/G533C (ox)
(j)
TACC3 522-536 V531C/T534C (mal)
TACC3 522-536 V531C/T534C (red)
TACC3 522-536 V531C/T534C (ox)
(k)
TACC3 522-536 L532C/G535C (mal)
TACC3 522-536 L532C/G535C (red)
TACC3 522-536 L532C/G535C (ox)
(l)
TACC3 522-536 G533C/A536C (mal)
TACC3 522-536 RG533C/A536C (red)
TACC3 522-536 G533C/A536C (ox)
Normalized r
[Competitor] (nM)

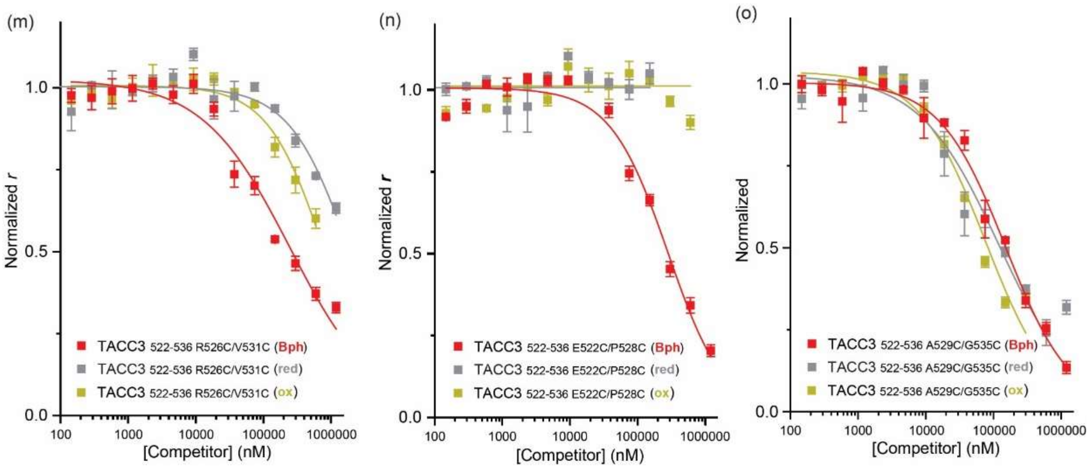

Figure S13. Competition FA results for peptides in Table 3. (25 mM Tris, 150 mM NaCl, 5 mM $MgCl_2$, pH= 7.5, 5 µM Aurora-$A_{122-403-C290A/C393}$, 200 nM FAM-Ahx-$TACC3_{522-536}$, 25 °C).

TACC3 $_{522-536}$ sequence optimization

Peptide	Sequence[a]	K_d [b] (µM)	IC_{50}[b] (µM)
TACC3 $_{522-563}$	Fam-Ahx-EESFRDPAEVLGTGA EVDYLEQFGTSSFKESALRKQSLYLKF -NH_2	9.6 ± 0.5	
	-3_{10}- domain; -αTR- domain		
TACC3 $_{522-536}$	Fam-Ahx-EESFRDPAEVLGTGA -NH_2	10.4 ± 1.1	
TACC3 $_{522-536}$	Ac- EESFRDPAEVLGTGA -NH_2		168 ± 15
TACC3 $_{522-532}$	Fam-Ahx-EESFRDPAEVL -NH_2	7.1 ± 0.8	
TACC3 $_{522-542}$	Fam-Ahx-EESFRDPAEVLGTGA EVDYLE -NH_2	4.5 ± 0.2	
TACC3 $_{522-552}$	Fam-Ahx-EESFRDPAEVLGTGA EVDYLEQFGTSSFKES -NH_2	5.2 ± 0.5	
TACC3 $_{536-563}$	Fam-Ahx- A EVDYLEQFGTSSFKESALRKQSLYLKF -NH_2	≥ 64 ± 8	
TACC3 $_{518-532}$	Fam-Ahx-LELK EESFRDPAEVL -NH_2	7.4 ± 0.2	
TACC3 $_{518-532}$	Ac- LELK EESFRDPAEVL -NH_2		123 ± 6

Table S4. TACC3 $_{522-536}$ sequence optimization. [a] One letter code for amino acids. [b] K_d values given as the mean value and corresponding standard deviation (SD) determined from triplicate titrations of Aurora-A $_{122-403-C290A/C393A}$ in the presence of the corresponding fluorescein labeled peptides (50 nM) (n= 3). All assays were performed in 25 mM Tris, 150 mM NaCl, 5 mM $MgCl_2$, pH= 7.5.

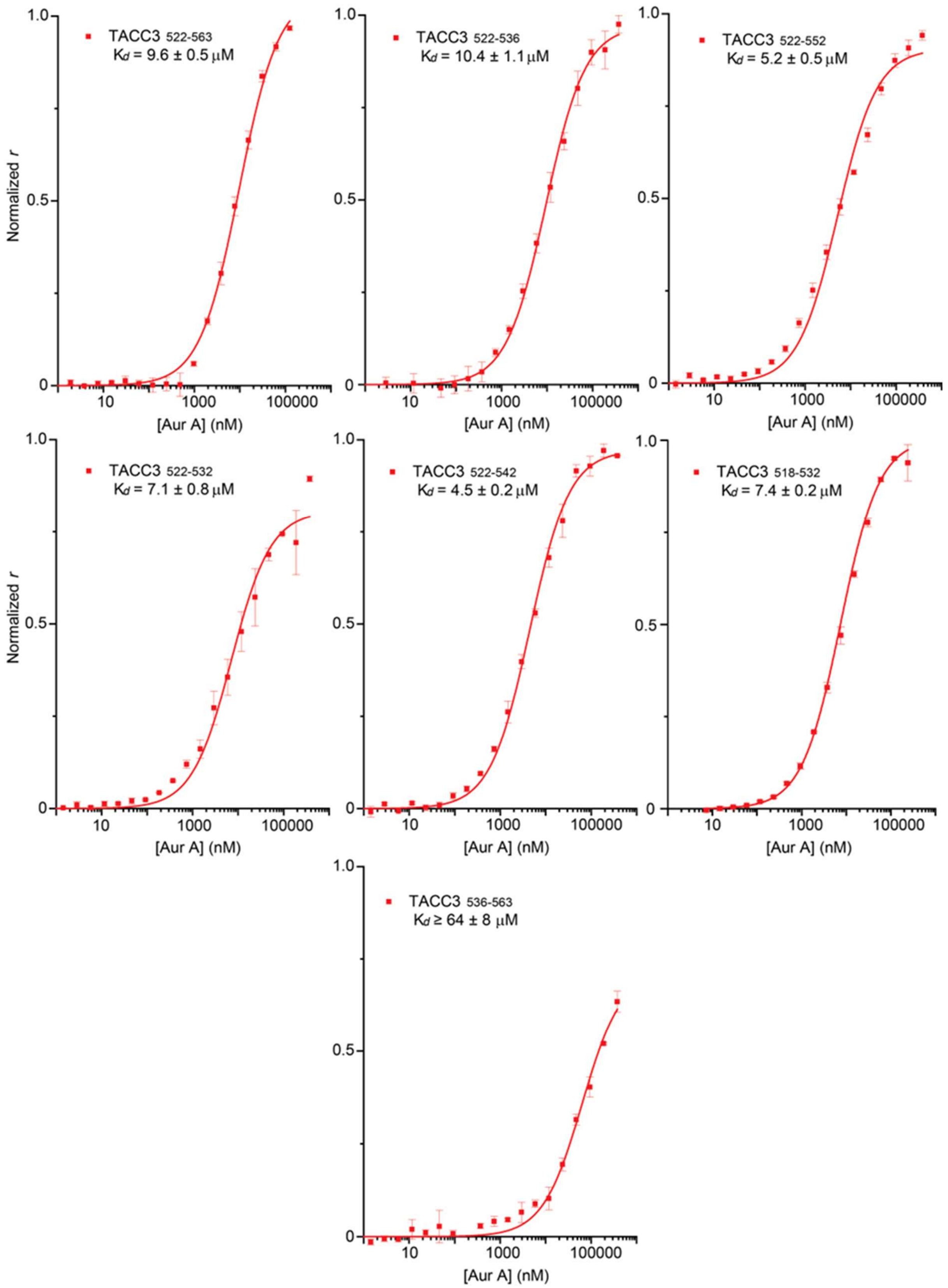

Figure S14. FA direct binding assays for peptides variants in Table S4. (25 mM Tris, 150 mM NaCl, 5 mM $MgCl_2$, pH= 7.5, 50 nM fluorescein-labeled FAM-Ahx-peptides, 25 °C).

MD analysis of WT TACC3 $_{518\text{-}532}$ interaction with Aurora-A

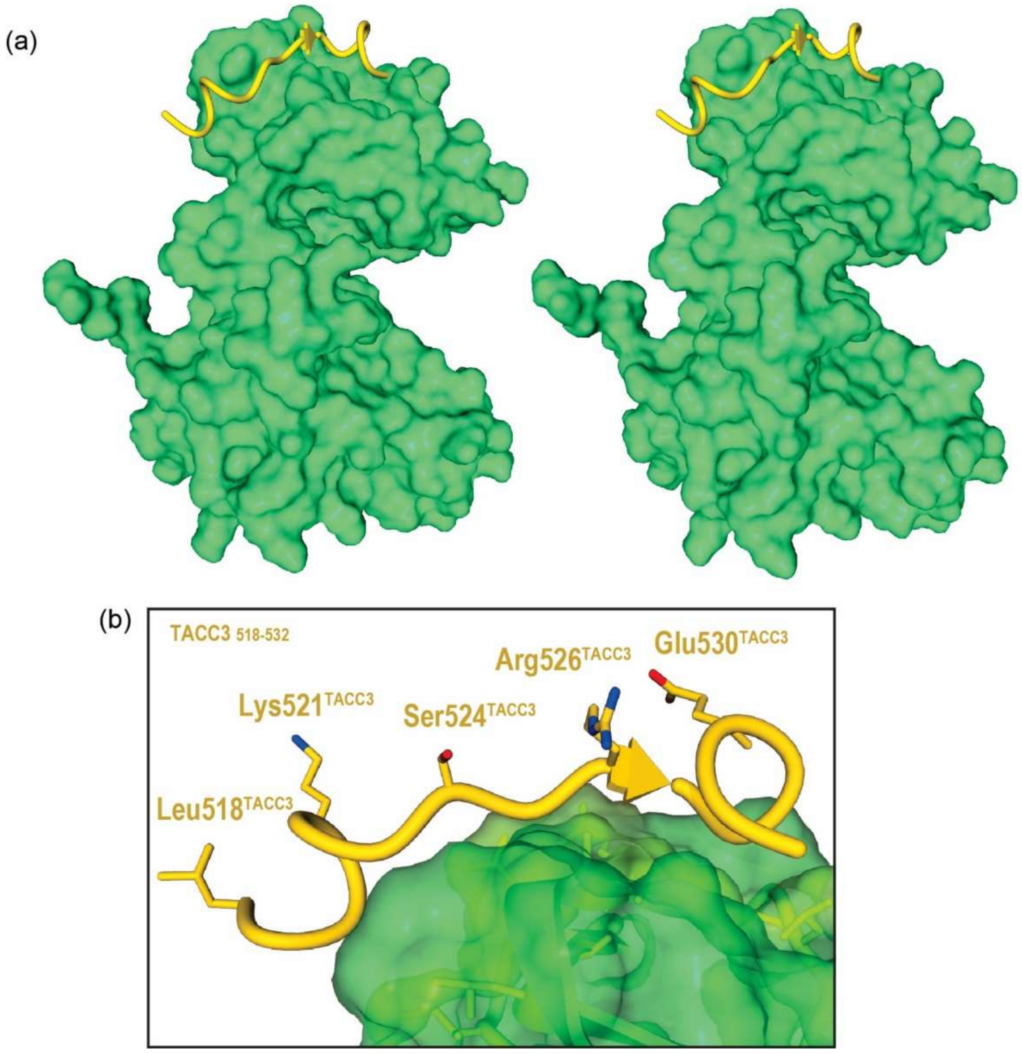

Figure S15. (a) Energy minimized structure of Aurora-A$_{122–403\text{-}C290A/C393A/D274N}$ /TACC3$_{518\text{-}532}$ complex after 150 ns of MD simulation (600 frames). (b) Highlighted in the inset are TACC3$_{518\text{-}532}$ predicted solvent-exposed residues at the interface of Aurora-A.

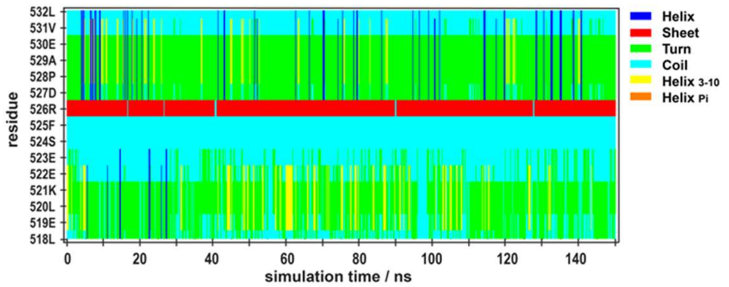

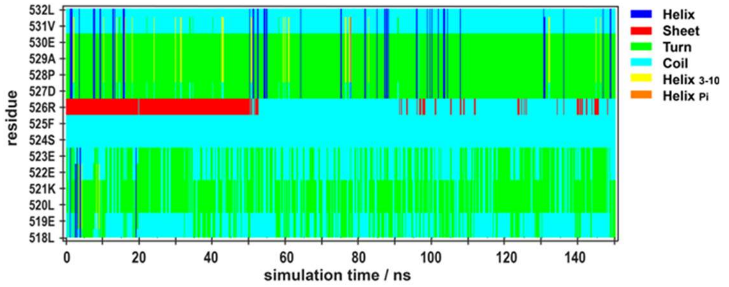

Figure S16. Replicate MD simulations of bound TACC3$_{518\text{-}532}$ in complex with Aurora-A$_{122\text{–}403\text{-}C290A/C393A/D274N}$, showing TACC3$_{518\text{-}532}$ per-residue secondary structure.

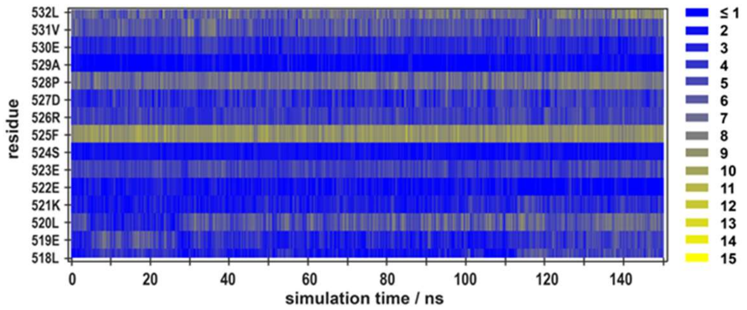

Per-residue average number of contacts per frame (2.5 fs; n= 600)															
Residue	518	519	520	521	522	523	524	525	526	527	528	529	530	531	532
Avg. number of contacts	3.1	3.6	5.5	2.8	1.8	4.6	1.8	8.5	4.4	3.8	7.3	1.2	3.3	5.5	6.7

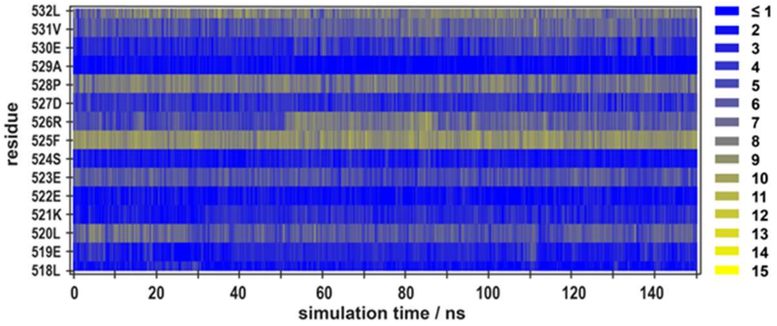

Per-residue average number of contacts per frame (2.5 fs; n= 600)															
Residue	518	519	520	521	522	523	524	525	526	527	528	529	530	531	532
Avg. number of contacts	2.0	2.8	5.9	3.0	1.8	5.5	2.2	8.4	6.2	3.6	7.4	1.3	3.7	6.1	7.7

Figure S17. Replicate MD simulations of bound TACC3 $_{518\text{-}532}$ in complex with Aurora-A$_{122\text{–}403\text{-}C290A/C393A/D274N}$, showing TACC3$_{518\text{-}532}$ per-residue number of contacts to the protein.

TACC3 $_{518-532}$ (*i*, *i+6*) constrained variants

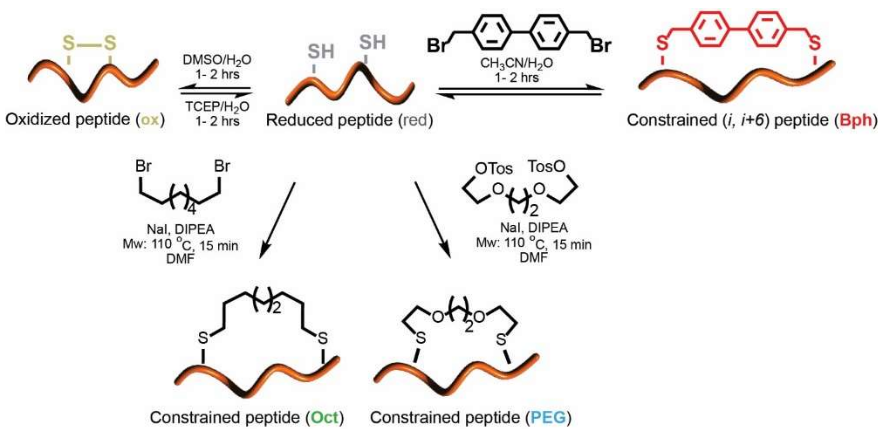

Constraint position	Peptide	Sequence[a]	IC_{50}[b] (μM)
**NO constrain**	**TACC3 $_{518-532}$ WT**	**Ac- LELKEESFRDPAEVL -NH2**	**123 ± 6**
	TACC3 $_{518-532}$ (+DTT)	Ac- LELKEESFRDPAEVL -NH2	172 ± 18
	TACC3 $_{518-532}$ (+DMSO)	Ac- LELKEESFRDPAEVL -NH2	140 ± 18
**K521C/D527C constrain**	TACC3 $_{518-532\ K521C/D527C}$ (red)	Ac- LELCEESFRCPAEVL -NH2	148 ± 12
	TACC3 $_{518-532\ K521C/D527C}$ (ox)	Ac- LELCEESFRCPAEVL -NH2	125 ± 12
	TACC3 $_{518-532\ K521C/D527C}$ (Bph)	Ac- LELCEESFRCPAEVL -NH2 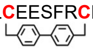	69 ± 5
**L520C/R526C constrain**	TACC3 $_{518-532\ L520C/R526C}$ (red)	Ac- LECKEESFCDPAEVL -NH2	153 ± 10
	TACC3 $_{518-532\ L520C/R526C}$ (ox)	Ac- LECKEESFCDPAEVL -NH2	≥ 200
	TACC3 $_{518-532\ L520C/R526C}$ (Bph)	Ac- LECKEESFCDPAEVL -NH2 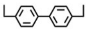	30 ± 2
	TACC3 $_{518-532\ L520C/R526C}$ (Oct)	Ac- LECKEESFCDPAEVL -NH2	114 ± 12
	TACC3 $_{518-532\ L520C/R526C}$ (PEG)	Ac- LECKEESFCDPAEVL -NH2	≥ 200
**S524C/E530C constrain**	TACC3 $_{518-532\ S524C/E530C}$ (red)	Ac- LELKEECFRDPACVL -NH2	76 ± 14
	TACC3 $_{518-532\ S524C/E530C}$ (ox)	Ac- LELKEECFRDPACVL -NH2	197 ± 27
	TACC3 $_{518-532\ S524C/E530C}$ (Bph)	Ac- LELKEECFRDPACVL -NH2 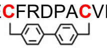	26 ± 2
	TACC3 $_{518-532\ S524C/E530C}$ (Oct)	Ac- LELKEECFRDPACVL -NH2	≥ 200
	TACC3 $_{518-532\ S524C/E530C}$ (PEG)	Ac- LELKEECFRDPACVL -NH2	≥ 200

Table S5. Additional TACC3 $_{518-532}$ (*i*, *i* + 6) constraint scan. [a] One letter code for amino acids. [b] IC_{50} values given as the mean value and corresponding standard deviation (SD) determined from triplicate competition FA assays against fluorescein labeled WT TACC3 $_{522-526}$ Ahx-FAM (200 nM) in the presence of Aurora-A $_{122-403-C290A/C393A}$ (5 μM)(n= 3). All assays were performed in 25 mM Tris, 150 mM NaCl, 5 mM $MgCl_2$, pH= 7.5.

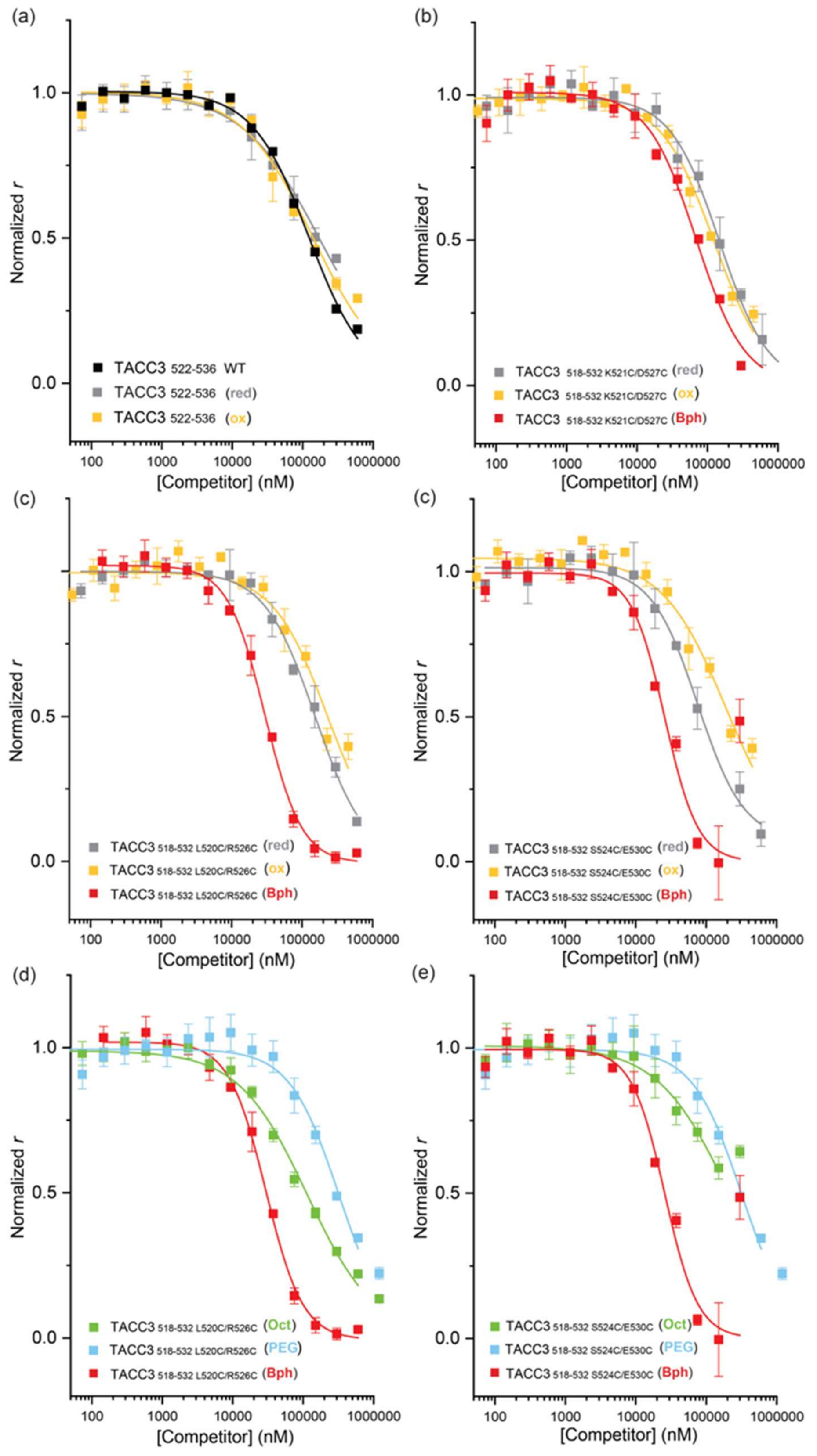

Figure S18. Competition FA results for peptides in Table 5. (25 mM Tris, 150 mM NaCl, 5 mM $MgCl_2$, pH= 7.5, 5 µM Aurora-$A_{122\text{-}403\text{-}C290A/C393A}$, 200 nM FAM-Ahx-$TACC3_{522\text{-}536}$, 25 °C)

MD analysis of biphenyl- constrained TACC3 $_{518\text{-}532\text{-}L/R\text{-}Bph}$ interaction with Aurora-A

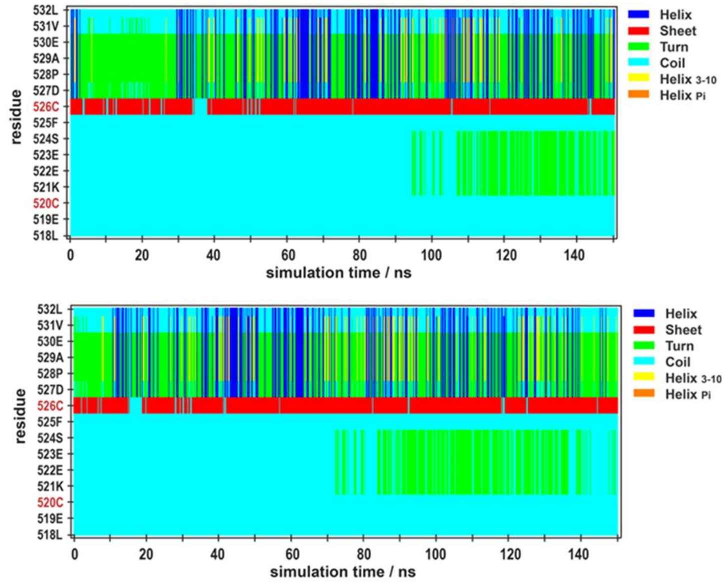

Figure S19. Replicate MD simulations of bound constrained TACC3$_{518\text{-}532\text{-}L/R\text{-}Bph}$ in complex with Aurora-A$_{122–403\text{-}C290A/C393A/D274N}$, showing TACC3$_{518\text{-}532\text{-}L/R\text{-}Bph}$ per-residue secondary structure.

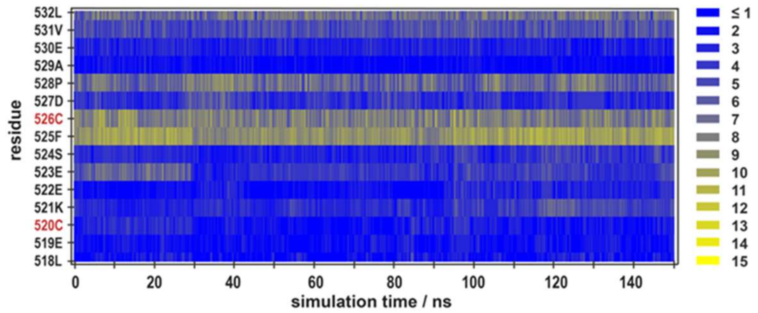

Per-residue average number of contacts per frame (2.5 fs; n= 600)															
Residue	518	519	520	521	522	523	524	525	526	527	528	529	530	531	532
Avg. number of contacts	1.8	1.8	2.3	3.8	2.1	4.4	3.3	9.4	7.9	3.7	6.9	1.3	2.7	5.3	7.1

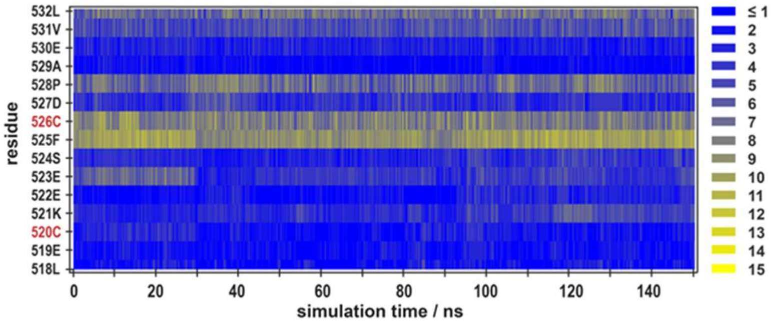

Per-residue average number of contacts per frame (2.5 fs; n= 600)															
Residue	518	519	520	521	522	523	524	525	526	527	528	529	530	531	532
Avg. number of contacts	1.8	1.9	2.3	3.8	2.3	4.5	3.5	9.5	7.9	3.7	6.9	1.4	2.7	5.3	7.1

Figure S20. Replicate MD simulations of bound TACC3$_{518\text{-}532\text{-}L/R\text{-}Bph}$ in complex with Aurora-A$_{122–403\text{-}C290A/C393A/D274N}$, showing TACC3$_{518\text{-}532\text{-}L/R\text{-}Bph}$ per-residue number of contacts to the protein.

MD analysis of biphenyl- constrained TACC3 $_{518\text{-}532\text{-}S/E\text{-}Bph}$ interaction with Aurora-A

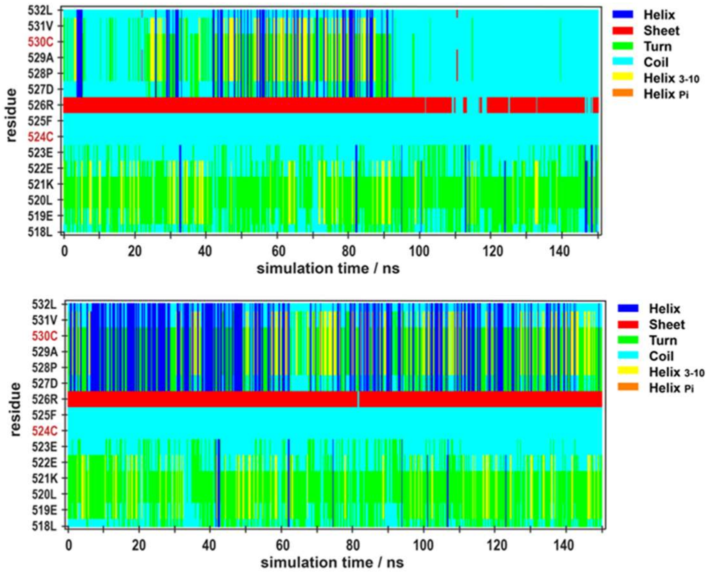

Figure S21. Replicate MD simulations of bound constrained TACC3$_{518\text{-}532\text{-}S/E\text{-}Bph}$ in complex with Aurora-A$_{122–403\text{-}C290A/C393A/D274N}$, showing TACC3$_{518\text{-}532\text{-}S/E\text{-}Bph}$ per-residue secondary structure.

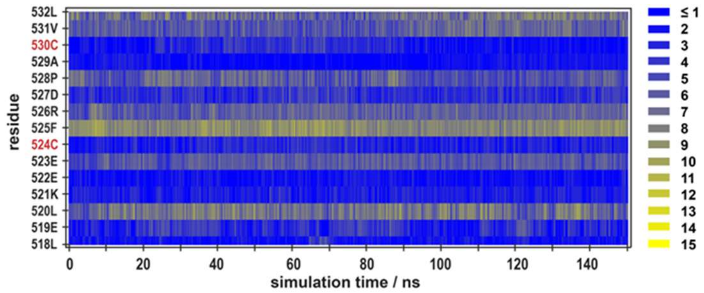

Per-residue average number of contacts per frame (2.5 fs; n= 600)															
Residue	518	519	520	521	522	523	524	525	526	527	528	529	530	531	532
Avg. number of contacts	2.0	3.3	7.3	2.6	1.8	5.8	2.8	8.6	6.1	3.6	5.9	1.6	2.5	6.0	7.0

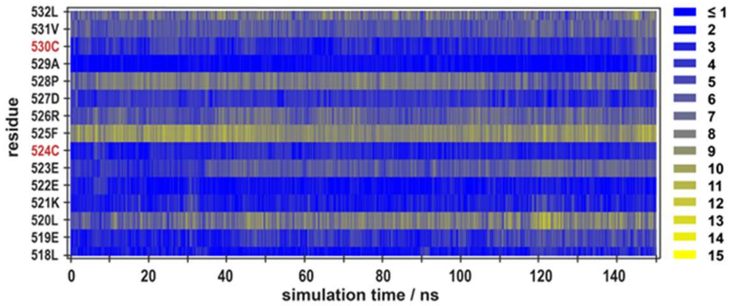

Per-residue average number of contacts per frame (2.5 fs; n= 600)															
Residue	518	519	520	521	522	523	524	525	526	527	528	529	530	531	532
Avg. number of contacts	2.3	3.8	7.6	2.8	2.0	5.6	2.9	8.9	6.2	3.8	7.4	1.5	3.5	5.9	6.9

Figure S22. Replicate MD simulations of bound TACC3$_{518\text{-}532\text{-}S/E\text{-}Bph}$ in complex with Aurora-A$_{122–403\text{-}C290A/C393A/D274N}$, showing TACC3$_{518\text{-}532\text{-}S/E\text{-}Bph}$ per-residue number of contacts to the protein.

TACC3$_{518-532}$ Bph- constrained/Phe525 halogen-substituted variants

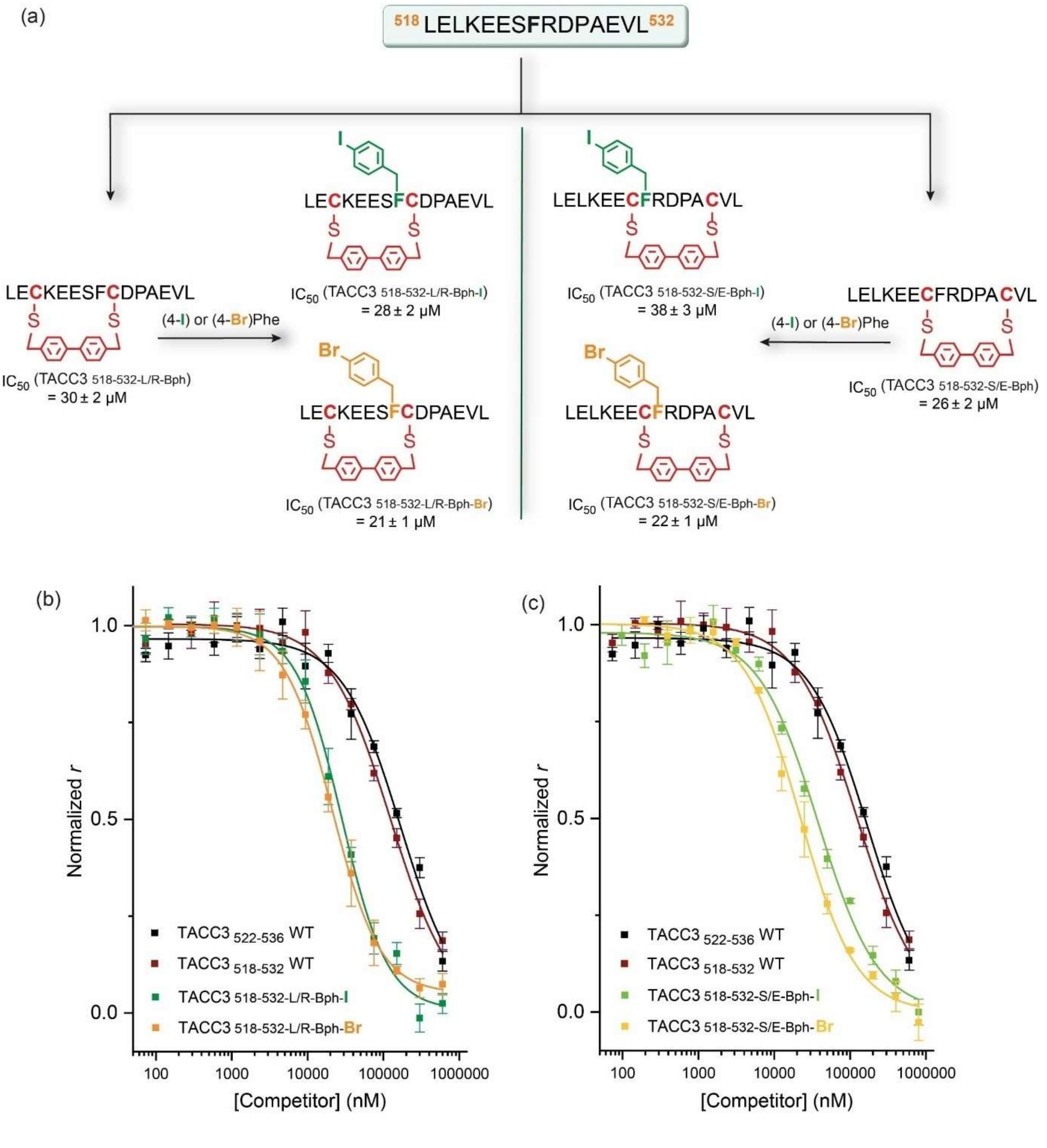

Figure S23. Competition FA results for Bph- constrained and Phe525 halogen-substituted TACC3$_{518-532}$ variants. (25 mM Tris, 150 mM NaCl, 5 mM $MgCl_2$, pH= 7.5, 5 µM Aurora-A$_{122-403-C290A/C393A}$, 200 nM Fam-Ahx-TACC3 $_{522-536}$, 25 °C).

TACC3$_{518-532}$ Bph- constrained/Phe525 halogen-substituted/ (4-F)Pro528 variants

Peptide	Sequence[a]			IC_{50}[b] (µM)	K_d [c] (µM)	ΔG[d] (kJ K^{-1} mol^{-1})
TACC3 $_{522-536}$	Ac-	EESFRDPAEVLGTGA	$-NH_2$	168 ± 15		
TACC3 $_{522-536}$	FAM-Ahx-	EESFRDPAEVLGTGA	$-NH_2$		10.47 ± 1.13	-28.5 ± 0.5
TACC3 $_{518-532\text{-L/R-Bph-IF/}f\text{P}}$	Ac-	LELCKEES^{I}FCD*f*PAEVL	$-NH_2$	17 ± 1		
TACC3 $_{518-532\text{-L/R-Bph-IF/}f\text{P}}$	FAM-Ahx-	LELCKEES^{I}FCD*f*PAEVL	$-NH_2$		1.42 ± 0.24	-33.4 ± 0.5
TACC3 $_{518-532\text{-L/R-Bph-BrF/}f\text{P}}$	Ac-	LELCKEES^{Br}FCD*f*PAEVL	$-NH_2$	22 ± 1		
TACC3 $_{518-532\text{-L/R-Bph-BrF/}f\text{P}}$	FAM-Ahx-	LELCKEES^{Br}FCD*f*PAEVL	$-NH_2$		1.48 ± 0.16	-33.3 ± 0.3
TACC3 $_{518-532\text{-S/E-Bph-IF/}f\text{P}}$	Ac-	LELKEEC^{I}FRD*f*PACVL	$-NH_2$	12 ± 1		
TACC3 $_{518-532\text{-S/E-Bph-IF/}f\text{P}}$	FAM-Ahx-	LELKEEC^{I}FRD*f*PACVL	$-NH_2$		0.90 ± 0.20	-34.6 ± 0.6
TACC3 $_{518-532\text{-S/E-Bph-BrF/}f\text{P}}$	Ac-	LELKEEC^{Br}FRD*f*PACVL	$-NH_2$	16 ± 1		
TACC3 $_{518-532\text{-S/E-Bph-BrF/}f\text{P}}$	FAM-Ahx-	LELKEEC^{Br}FRD*f*PACVL	$-NH_2$		0.62 ± 0.15	-35.5 ± 0.6

Table S6. Fully modified TACC3$_{518-532}$ Bph- constrained leads. [a] One letter code for amino acids. [b] IC_{50} values given as the mean value and corresponding standard deviation (SD) determined from competition FA assays against fluorescein labeled FAM-Ahx-TACC3$_{522-536}$ (200 nM) in the presence of Aurora-A$_{122-403-C290A/C393A}$ (5 µM)(n= 6). [c] K_d values and [d] ΔG given as the mean value and corresponding standard deviation (SD) determined from direct Aurora-A$_{122-403-C290A/C393A}$ titration FA assays in the presence of the corresponding fluorescein-labeled peptides (50 nM) (n= 6). All assays were performed in 25 mM Tris, 150 mM NaCl, 5 mM $MgCl_2$, pH= 7.5.

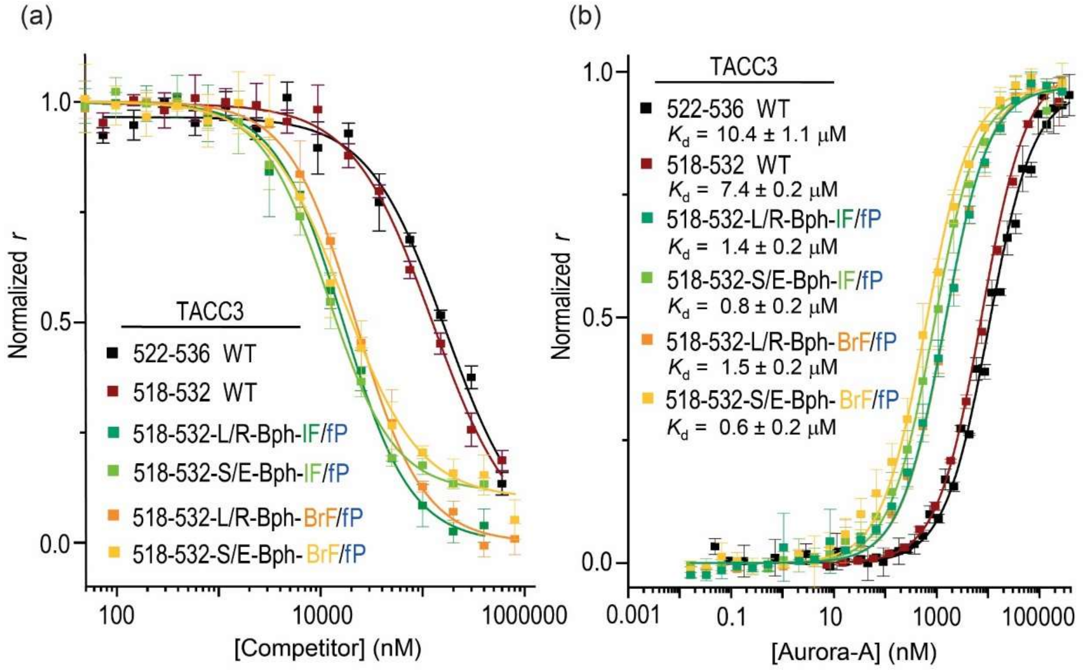

Figure S24. (a) Competition and (b) direct FA Aurora-A titration assays for peptides in Table S6. (25 mM Tris, 150 mM NaCl, 5 mM $MgCl_2$, pH= 7.5, 25 °C).

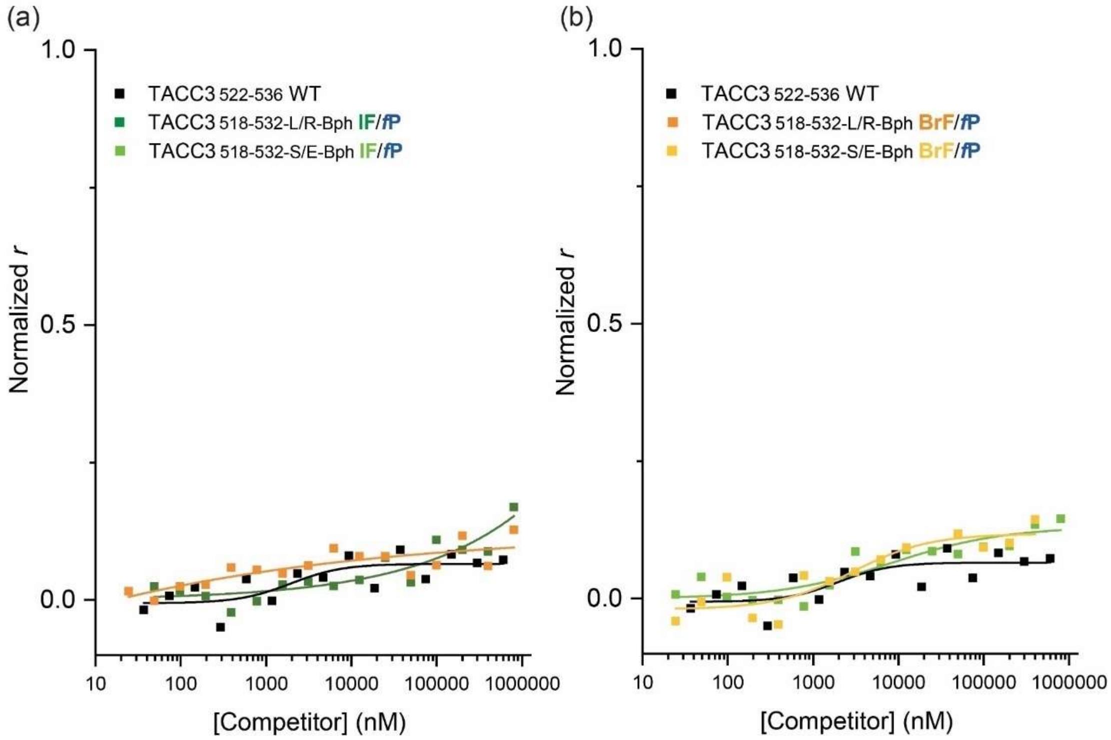

Figure S25. Control competition FA assays for peptides in Table S6 in the absence of protein. (25 mM Tris, 150 mM NaCl, 5 mM $MgCl_2$, pH= 7.5, 200 nM FAM-Ahx-TACC3 $_{522\text{-}536}$, 25 °C)

MD analysis of constrained TACC3 $_{518\text{-}532\text{-}L/R\text{-}Bph\text{-}IF/fP}$ interaction with Aurora-A

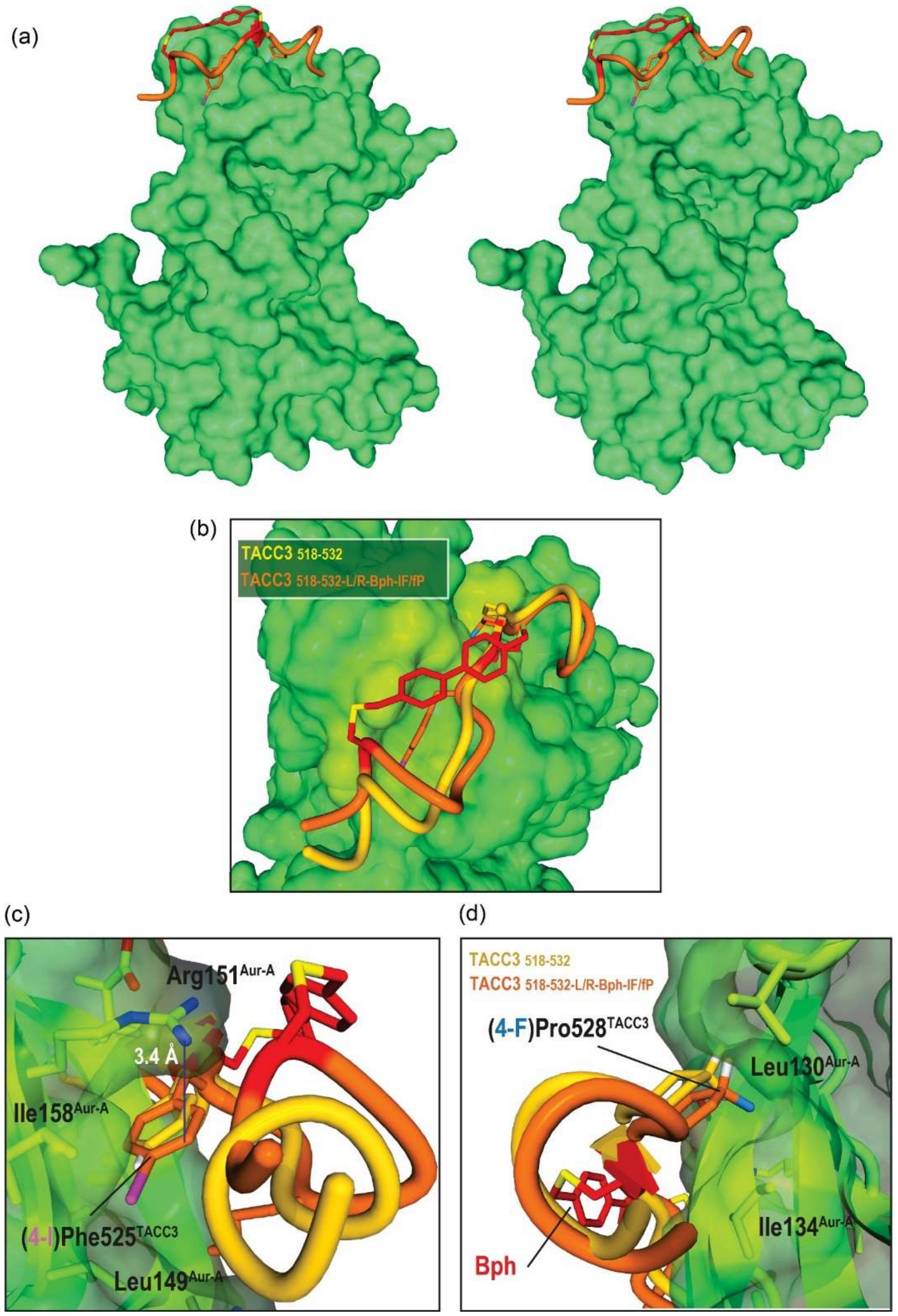

Figure S26. (a) Energy minimized structure of Aurora-A$_{122\text{–}403\ C290A/C393A/D274N}$ /TACC3$_{518\text{-}532\text{-}L/R\text{-}Bph\text{-}IF/fP}$ complex after 150 ns of MD simulation (600 frames). (b) Inset showing the solvent-exposed constraint at the interface of Aurora-A. (c) Inset showing the accommodation of iodinated (4-I)-Phe525^{TACC3} in its binding pocket. (d) Inset showing the accommodation and *exo*-pucker conformation of fluorinated *trans*-(4-F)Pro528^{TACC3} in its binding pocket.

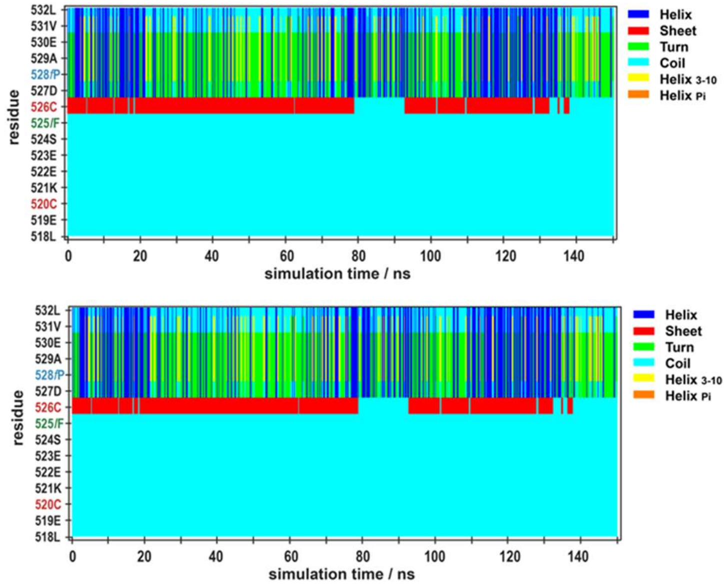

Figure S27. Replicate MD simulations of bound constrained TACC3$_{518\text{-}532\text{-}L/R\text{-}Bph\text{-}IF/fP}$ in complex with Aurora-A$_{122–403\text{-}C290A/C393A/D274N}$, showing TACC3$_{518\text{-}532\text{-}L/R\text{-}Bph\text{-}IF/fP}$ per-residue secondary structure.

As shown in Fig. S28, MD trajectory analyses for the final modified L/R-constrained peptide still indicated an increased number of contacts between (4-I)Phe525 (8.9-9.3 average number contacts per 2.5 fs segment) and *trans*-(4f)Pro528 residues (7.7-8.0) average number contacts per 2.5 fs segment) and Aurora-A, when compared two both WT linear sequences, TACC3$_{522\text{-}536}$ or TACC3$_{518\text{-}532}$. Results also indicate a significant contribution of constrained Cys526 residue to Aurora-A binding.

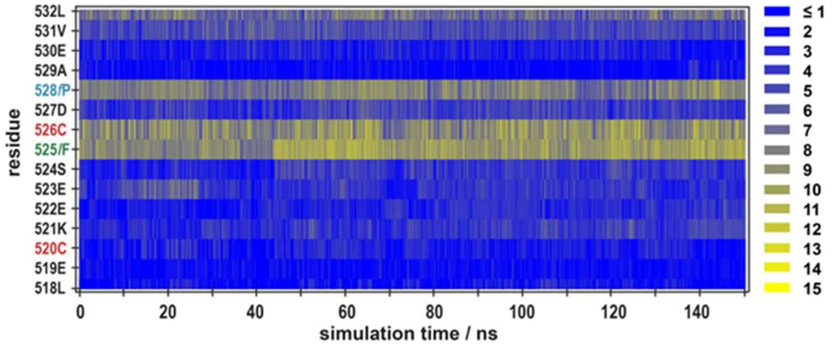

Per-residue average number of contacts per frame (2.5 fs; n= 600)															
Residue	518	519	520	521	522	523	524	525	526	527	528	529	530	531	532
Avg. number of contacts	1.8	1.6	2.5	3.9	3.0	3.9	3.7	9.3	8.7	4.3	8.0	1.4	2.6	5.1	7.0

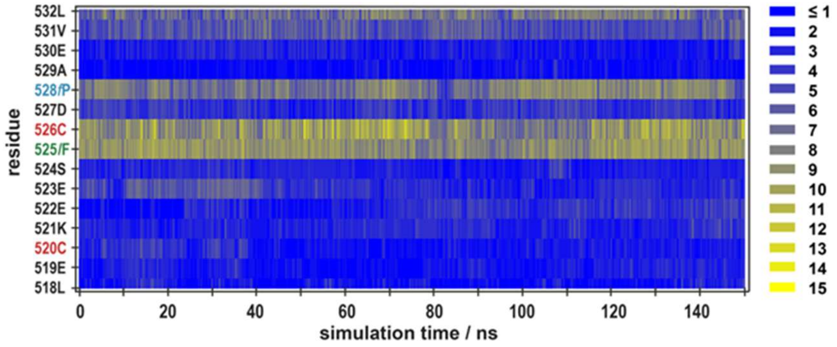

Per-residue average number of contacts per frame (2.5 fs; n= 600)															
Residue	518	519	520	521	522	523	524	525	526	527	528	529	530	531	532
Avg. number of contacts	1.9	2.2	2.4	3.0	3.0	3.9	2.7	8.9	8.9	3.7	7.7	1.4	2.5	5.6	7.1

Figure S28. Replicate MD simulations of bound TACC3$_{518\text{-}532_Bph_LR_IF/fP}$ in complex with Aurora-A$_{122–403\text{-}C290A/C393A/D274N}$, showing TACC3$_{518\text{-}532_Bph_LR_IF/fP}$ per-residue number of contacts to the protein.

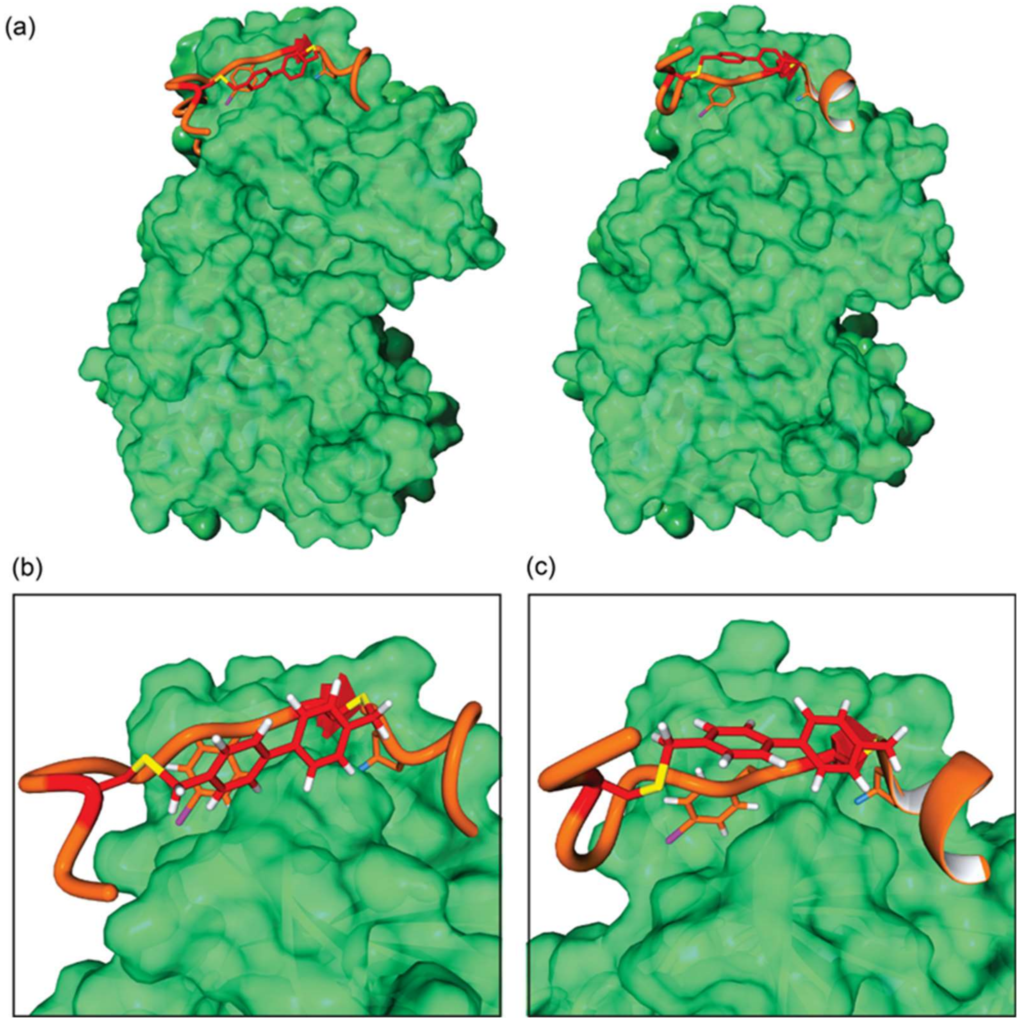

Figure S29. (a) Average structure of Aurora-A$_{122–403\text{-}C290A/C393A/D274N}$/TACC3 $_{518\text{-}532\text{-}L/R\text{-}Bph\text{-}IF/fP}$ complex after 150 ns of MD simulation (600 frames). (b) and (c) Insets showing the constraint at the interface of Aurora-A.

MD trajectory analysis of constrained TACC3 $_{518\text{-}532\text{-}S/E\text{-}Bph\text{-}IF/fP}$ interaction with Aurora-A

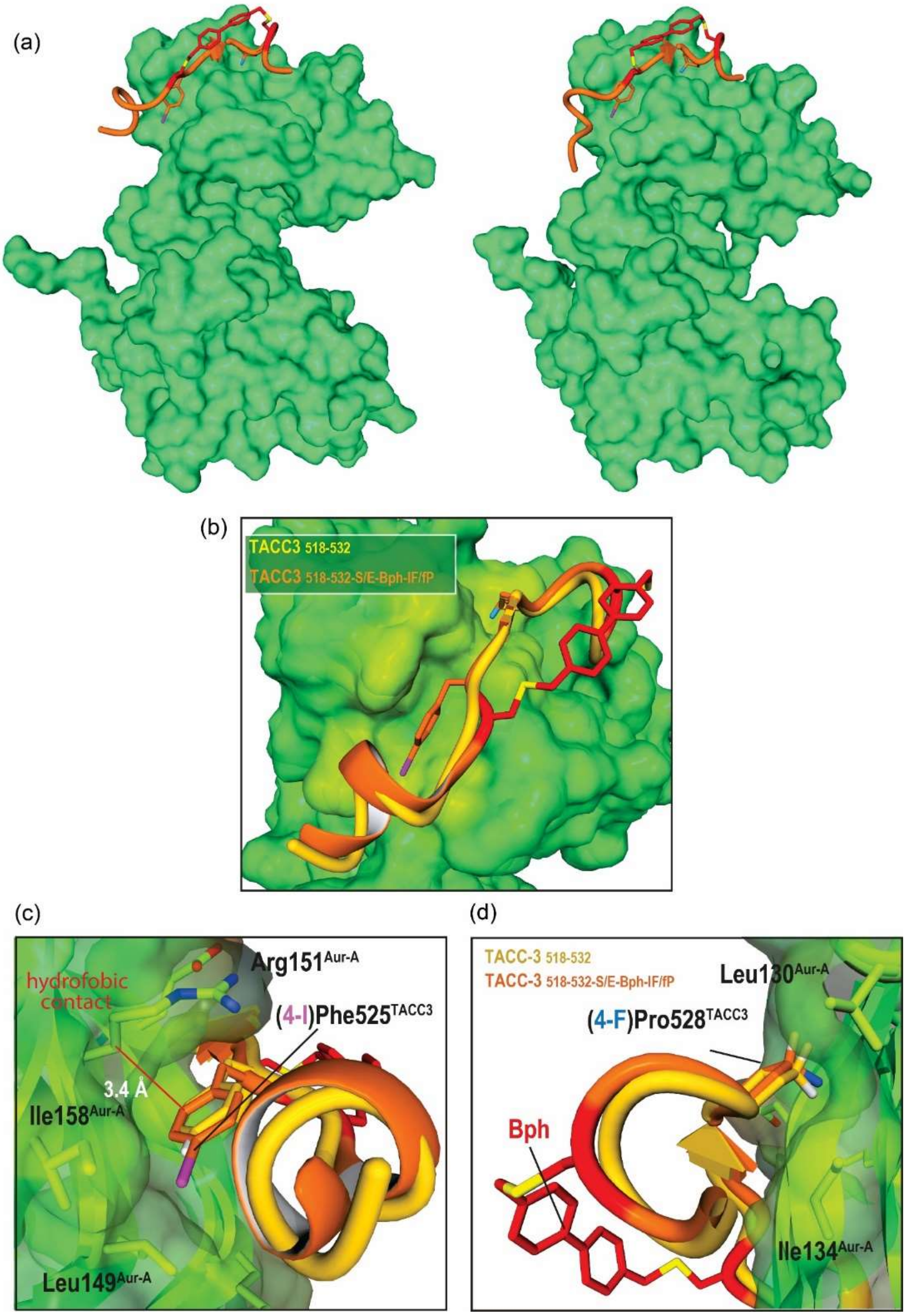

Figure S30. (a) Energy minimized structure of Aurora-A$_{122–403\text{-}C290A/C393A/D274N}$ /TACC3$_{518\text{-}532\text{-}S/E\text{-}Bph\text{-}IF/fP}$ complex after 150 ns of MD simulation (600 frames). (b) Inset showing the solvent exposed constraint at the interface of Aurora-A. (c) Inset showing the accommodation of iodinated (4-I)-Phe525^{TACC3} in its binding pocket. (d) Inset showing the accommodation and *exo*-pucker conformation of fluorinated *trans*-(4-F)Pro528^{TACC3} in its binding pocket.

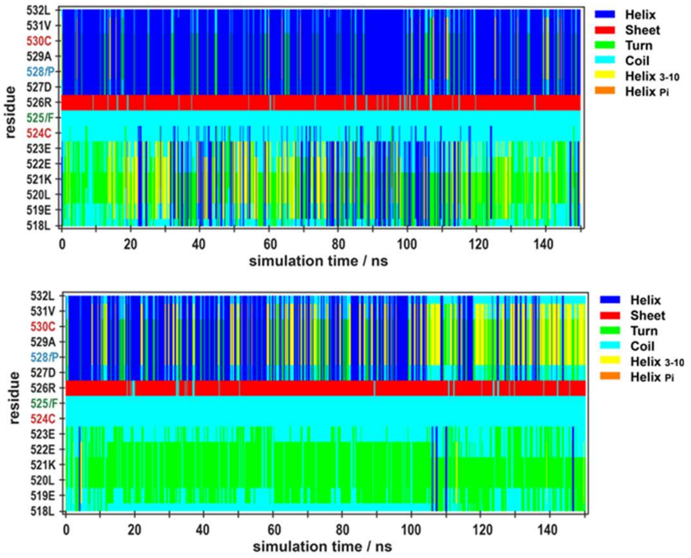

Figure S31. Replicate MD simulations of bound constrained TACC3$_{518\text{-}532\text{-}S/E\text{-}Bph\text{-}IF/fP}$ in complex with Aurora-A$_{122\text{–}403\text{-}C290A/C393A/D274N}$, showing TACC3$_{518\text{-}532\text{-}S/E\text{-}Bph\text{-}IF/fP}$ per-residue secondary structure.

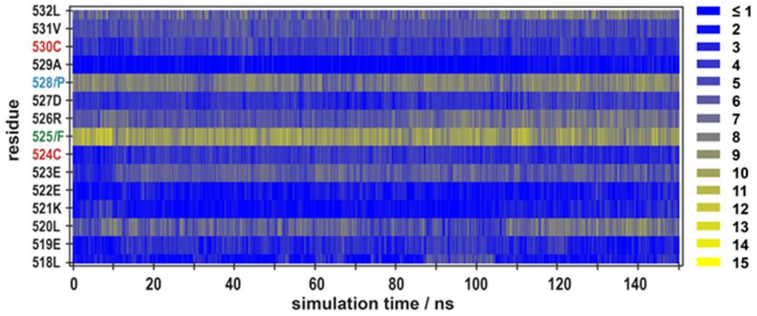

Per-residue average number of contacts per frame (2.5 fs; n= 600)															
Residue	518	519	520	521	522	523	524	525	526	527	528	529	530	531	532
Avg. number of contacts	2.6	3.2	6.4	2.1	2.1	5.9	3.7	9.5	6.4	3.8	8.0	1.4	4.0	5.4	6.8

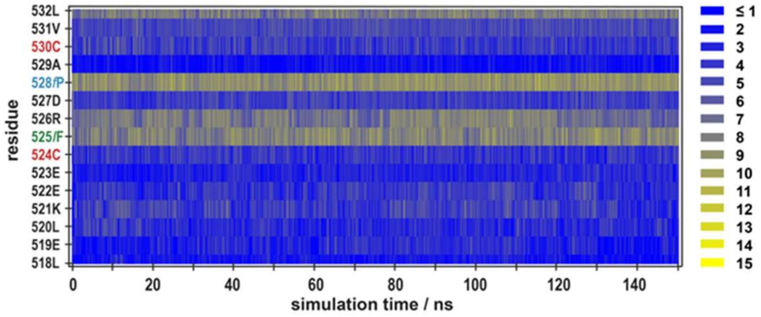

Per-residue average number of contacts per frame (2.5 fs; n= 600)															
Residue	518	519	520	521	522	523	524	525	526	527	528	529	530	531	532
Avg. number of contacts	2.2	3.0	3.4	4.4	4.0	2.8	3.9	8.1	7.6	4.3	8.9	1.8	4.3	5.0	7.8

Figure S32. Replicate MD simulations of bound TACC3$_{518\text{-}532\text{-}S/E\text{-}Bph\text{-}IF/fP}$ in complex with Aurora-A$_{122\text{–}403\text{-}C290A/C393A/D274N}$, showing TACC3$_{518\text{-}532\text{-}S/E\text{-}Bph\text{-}IF/fP}$ per-residue number of contacts to the protein.

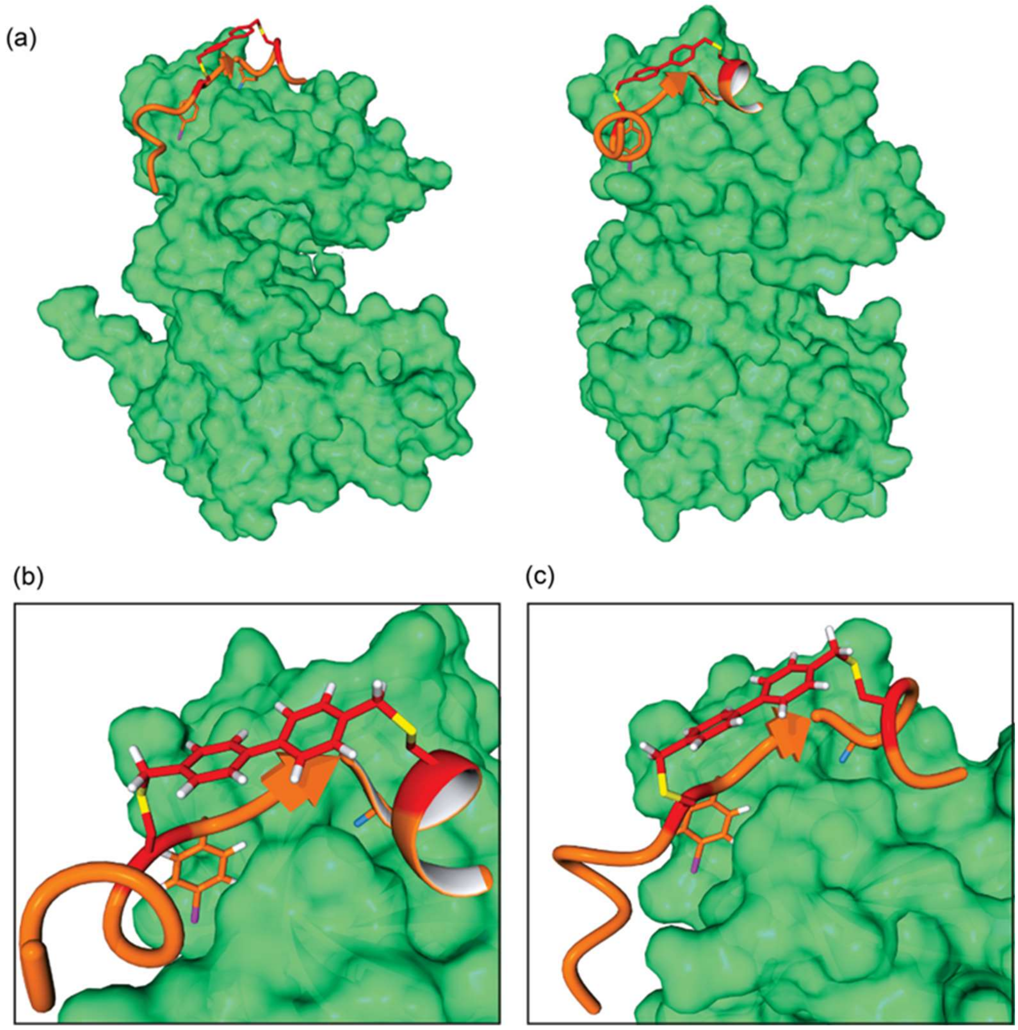

Figure S33. (a) Average structure of Aurora-A$_{122\text{–}403\text{-C290A/C393A/D274N}}$/TACC3$_{518\text{-}532\text{-S/E-Bph-IF/fP}}$ complex after 150 ns of MD simulation (600 frames). (b) and (c) Insets showing the constraint at the interface of Aurora-A.

Enzymatic stability of Peptidomimetics

In the presence of α-chymotrypsin, $TACC3_{518\text{-}532}$ was found to undergo rapid degradation at the aromatic Phe525 ($t_{1/2}$= 32 ± 4 min; Fig. S34a; Fig. S35). In contrast, when the L/R constrained variant was tested, it was resistant to chymotrypsin for ~ 6 hr with no cleavage by-products detected either by HPLC or LC/MS analysis (Fig. S34a; Fig S36). When the S/E constrained peptide was assessed (Fig S37) degradation was more rapid and in line with the unmodified sequence ($t_{1/2}$= 26 ± 2 min). However, LC/MS analysis of the digested products indicated that the constrained peptide was not cleaved at the central Phe525 residue, but at $Leu520^{TACC3}$ and $Val531^{TACC3}$. Similar behavior was observed in trypsin digests (Fig. 34b). Rapid degradation was observed for $TACC3_{518\text{-}532}$ at both positively charged residues, $Lys521^{TACC3}$ and $Arg523^{TACC3}$ ($t_{1/2}$= 4 ± 1 min, Fig. S38). In comparison, the L/R constrained was resistant to degradation by this enzyme with no digested products observed ($t_{1/2}$≥ 330 min, Fig. S39). The S/E variant, again, showed only partially improved stability, digesting quickly at the exposed $Lys521^{TACC3}$ residue but with suppressed cleavage at $Arg532^{TACC3}$, Fig. S40).

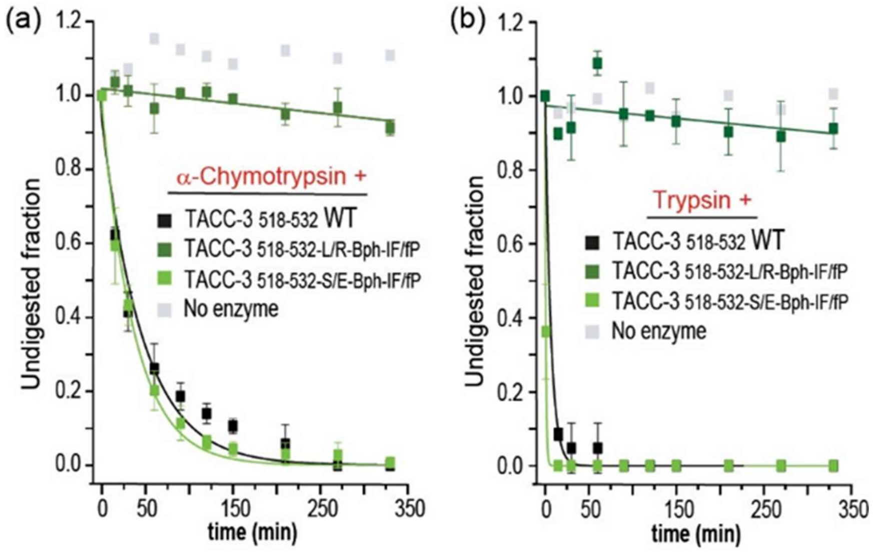

Figure S34. Proteolytic stability of constrained peptides. (a) Proteolytic stability of linear control $TACC3_{518\text{-}532}$ (black line), constrained $TACC3_{518\text{-}532\text{-}L/R\text{-}Bph\text{-}IF/fP}$ (forest green), and $TACC3_{518\text{-}532\text{-}S/E\text{-}Bph\text{-}IF/fP}$ (lemon green) in the presence of trypsin and **(b)** α-chymotrypsin. Analytical HPLC areas under the undegraded peptide peaks are normalized to the starting initial area of each peptide. Data is given as the average of three independent experiments and are shown as means ± SD.

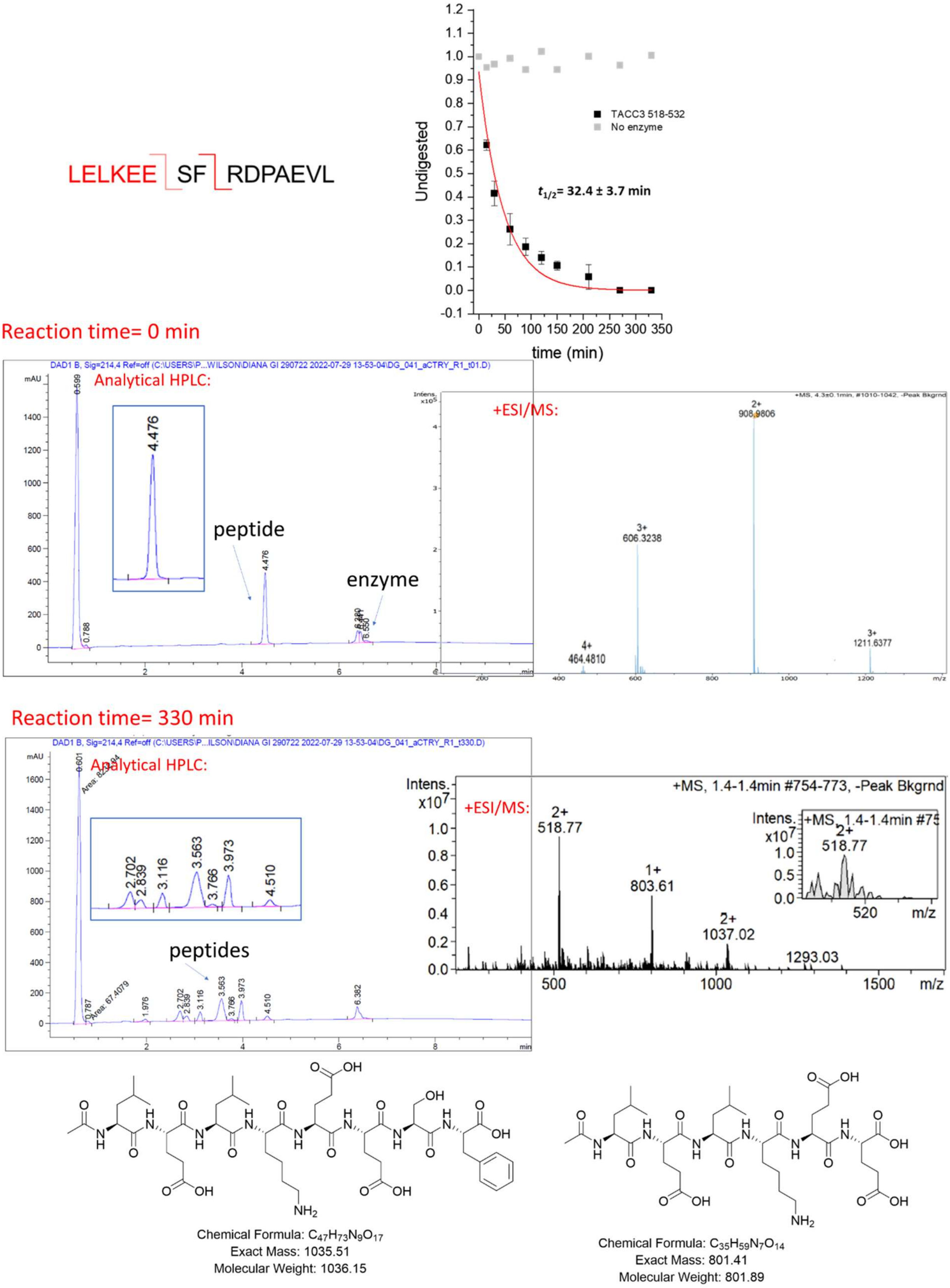

Figure S35. **Enzymatic stability of TACC3$_{518\text{-}532}$ in the presence of α-chymotrypsin**: peptide:enzyme ratio 1: 25 mol/mol in HEPES Buffer 25 mM, pH= 7.5, 25 °C.

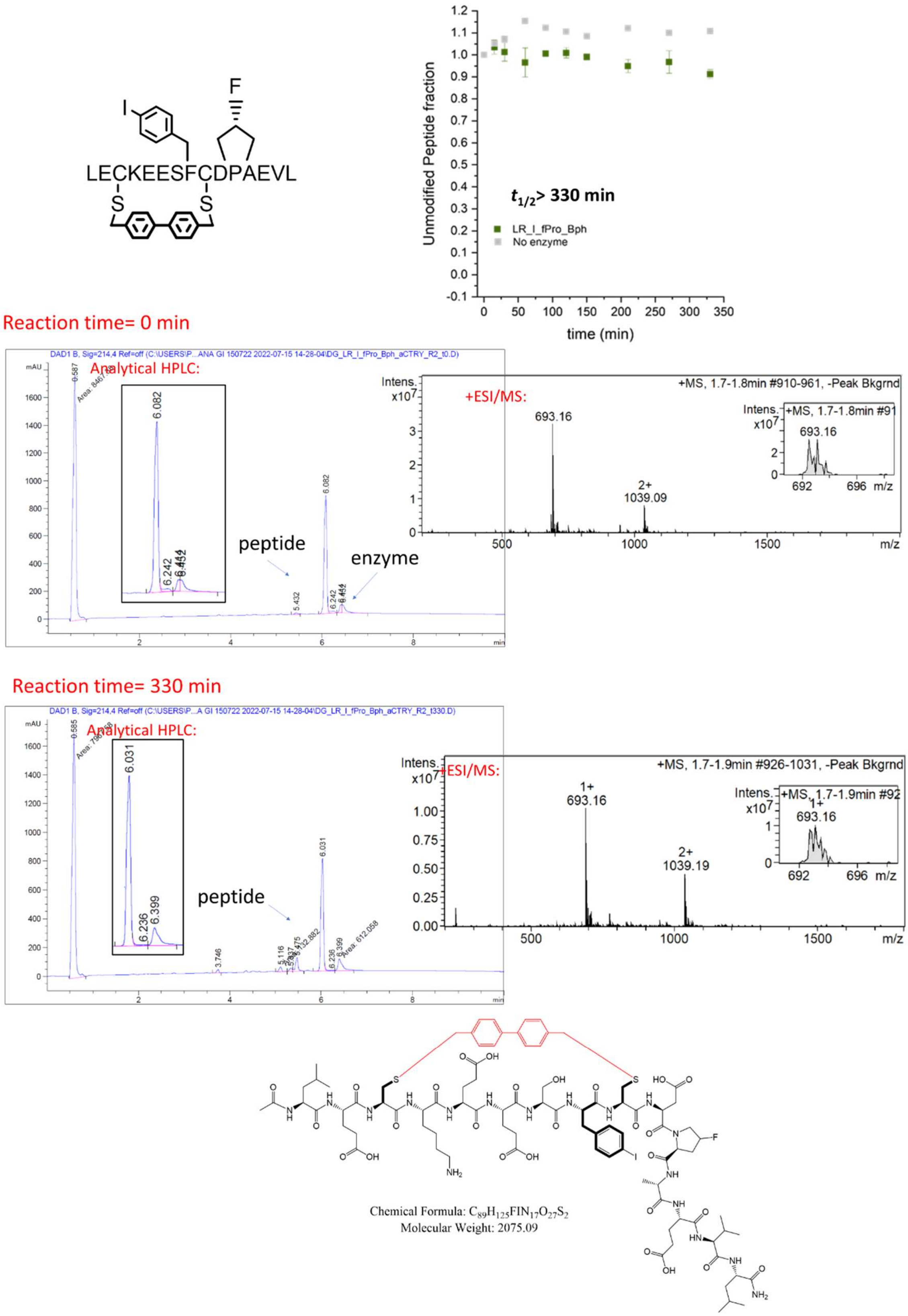

Figure S36. **Enzymatic stability of TACC3$_{518\text{-}532\text{-Bph-L/R-IF/fP}}$ the presence of α-chymotrypsin**: peptide:enzyme ratio 1: 25 mol/mol in HEPES Buffer 25 mM, pH= 7.5, 25 °C.

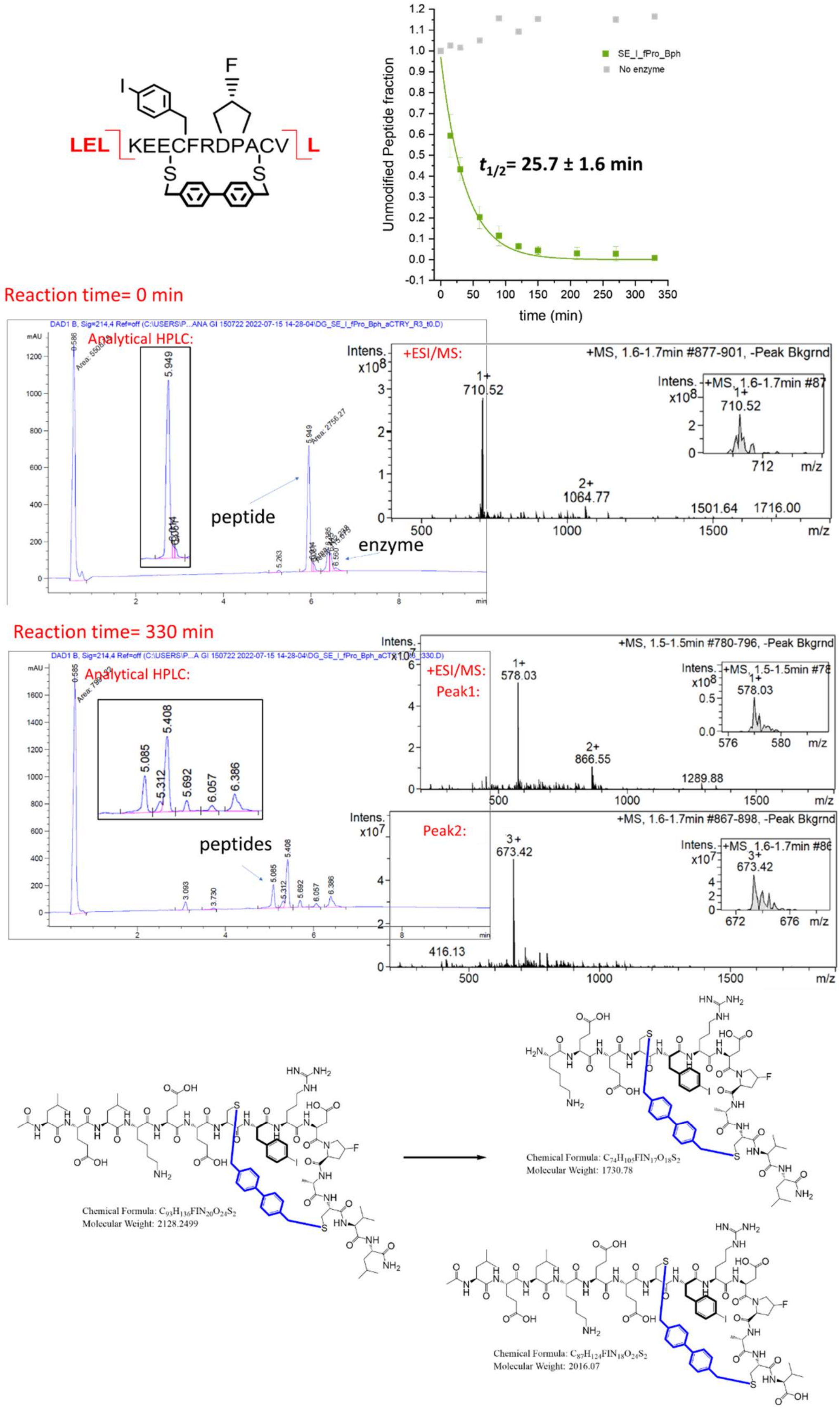

Figure S37. **Enzymatic stability of TACC3$_{518\text{-}532\text{-}Bph\text{-}S/E\text{-}IF/fP}$ in the presence of α-chymotrypsin**: peptide:enzyme ratio 1: 25 mol/mol in HEPES Buffer 25 mM, pH= 7.5, 25 °C.

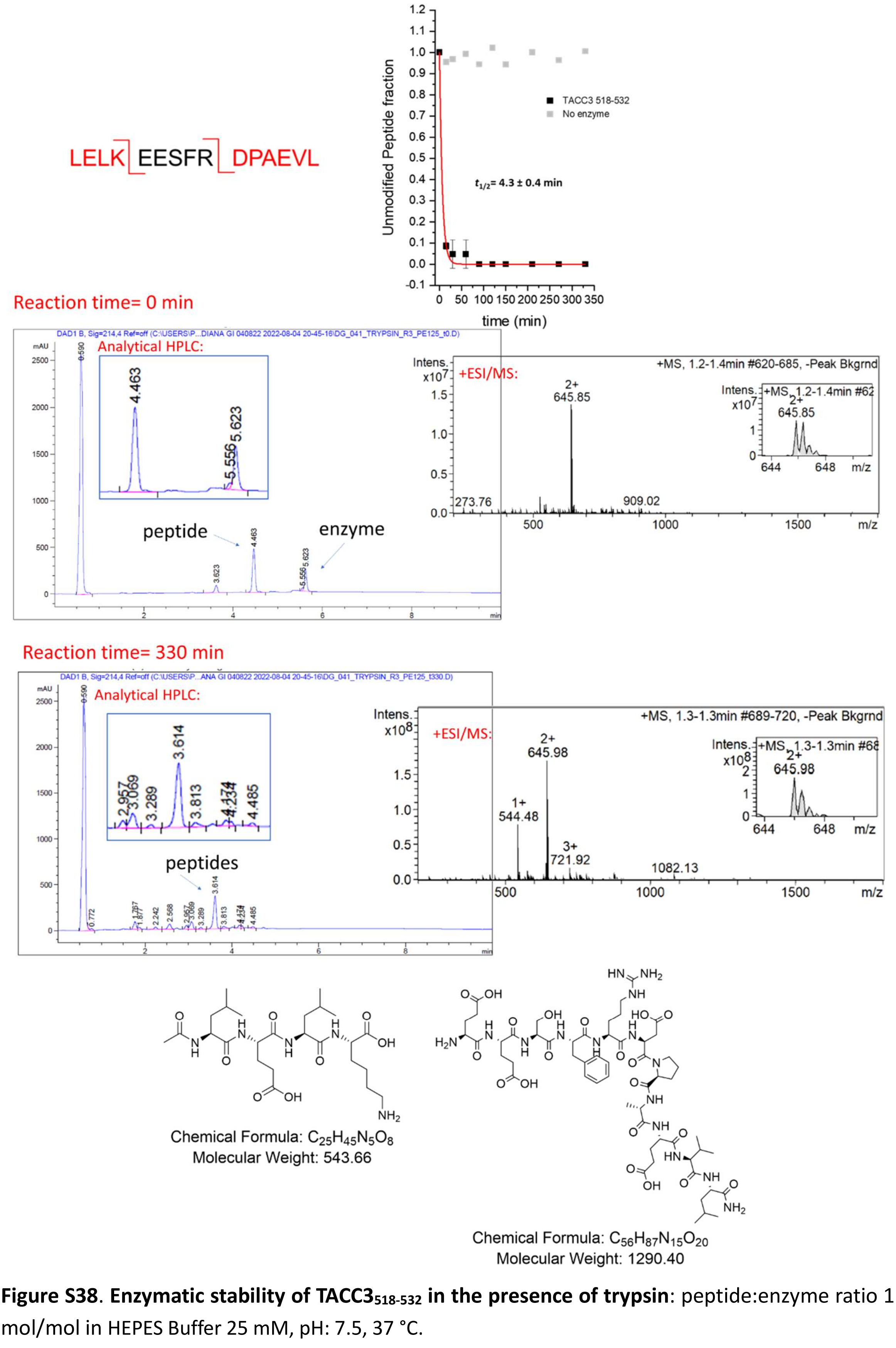

Figure S38. **Enzymatic stability of TACC3$_{518\text{-}532}$ in the presence of trypsin**: peptide:enzyme ratio 1: 25 mol/mol in HEPES Buffer 25 mM, pH: 7.5, 37 °C.

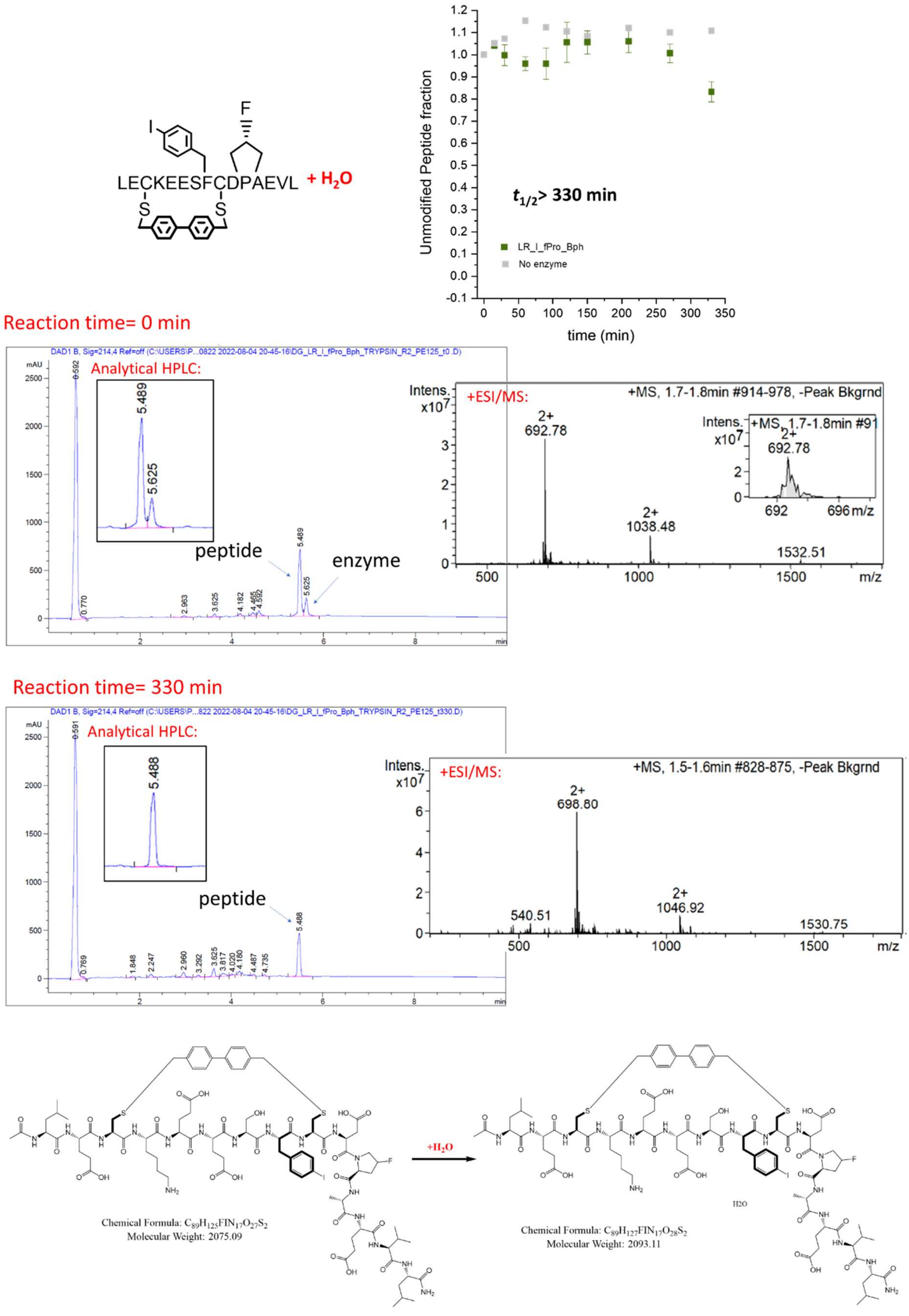

Figure S39. **Enzymatic stability of TACC3$_{518\text{-}532\text{-}Bph\text{-}L/R\text{-}IF/fP}$ in the presence of trypsin**: peptide:enzyme ratio 1: 25 mol/mol in HEPES Buffer 25 mM, pH= 7.5, 37 °C.

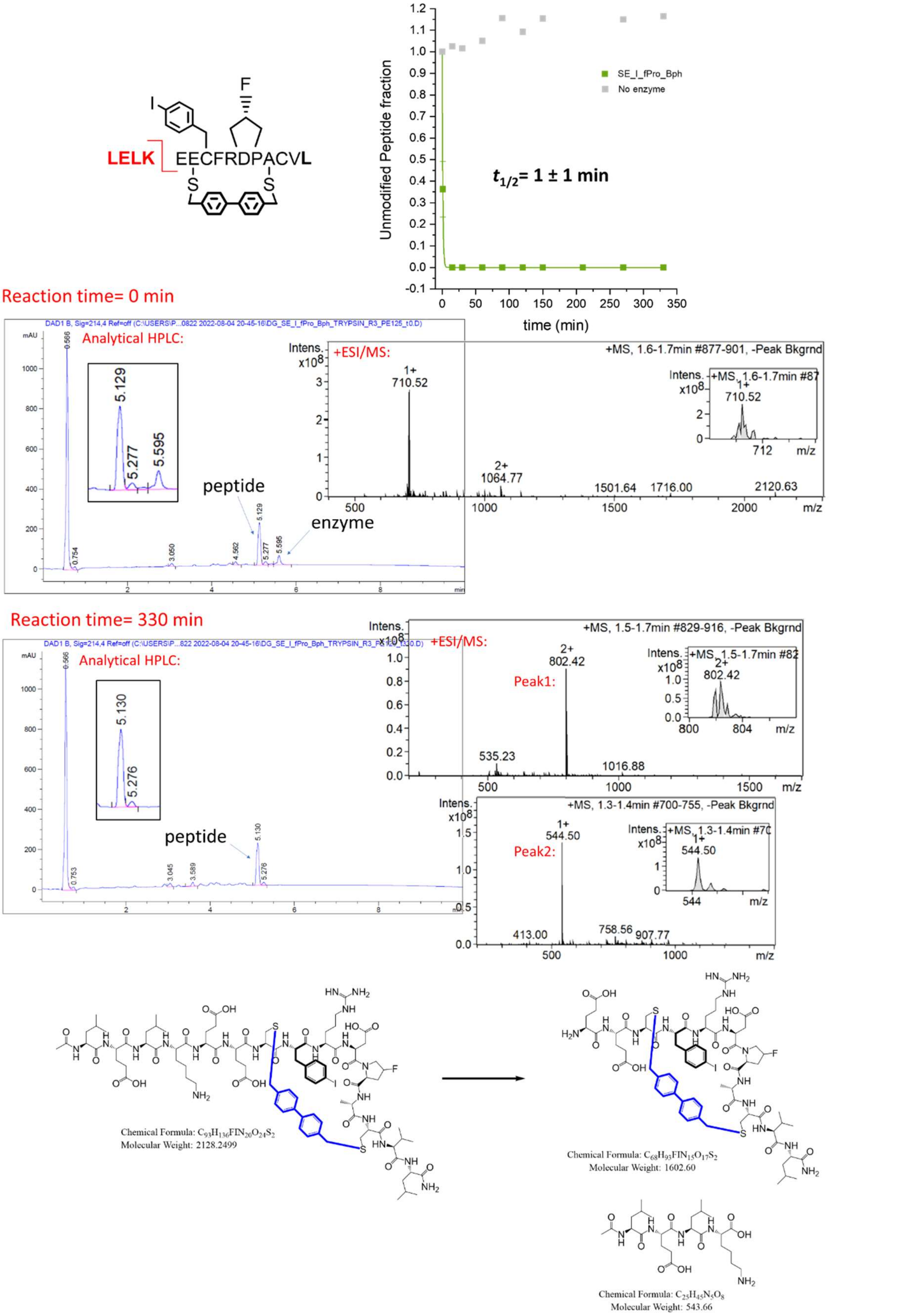

Figure S40. **Enzymatic stability of TACC3$_{518\text{-}532\text{-Bph-S/E-IF/fP}}$ in the presence of trypsin**: peptide:enzyme ratio 1: 25 mol/mol in HEPES Buffer 25 mM, pH= 7.5, 37 °C.

NMR analysis: TACC3 $_{518\text{-}532}$

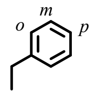

Ac-LELKEESFRDPAEVL

TACC3 $_{518\text{-}532}$ WT Residue[a]	$\delta_{NH,obs}$[b]	$\delta_{H\alpha,obs}$[b]	$\delta_{H\beta,obs}$[b]	$\delta_{H\gamma,obs}$[b]	$\delta_{H\delta,obs}$[b]	$\delta_{H\varepsilon,obs}$[b]	others
L518	8.374 (*d*, $^3J_{NH\text{-}\alpha H}$ = 6.102 Hz)	4.229	1.543/1.619 (*a*,*b*)	1.609	0.898/0.838		
E519	8.658 (*d*, $^3J_{NH\text{-}\alpha H}$ = 7.003 Hz)	4.235	1.964/1.872 (*a*,*b*)	2.202/2.172 (*a*,*b*)			
L520	8.342 (*d*, $^3J_{NH\text{-}\alpha H}$ = 5.952 Hz)	4.316	1.619/1.543 (*a*,*b*)	1.509	0.898/0.838		
K521	8.439 (*d*, $^3J_{NH\text{-}\alpha H}$ = 6.853 Hz)	4.292	1.784/1.738 (*a*,*b*)	1.394	1.628	2.935	NH_ε= 7.519
E522	8.597 (*d*, $^3J_{NH\text{-}\alpha H}$ = 6.102 Hz)	4.224	2.015/1.921 (*a*,*b*)	2.261			
E523	8.644 (*d*, $^3J_{NH\text{-}\alpha H}$ = 7.153 Hz)	4.235	1.964/1.872 (*a*,*b*)	2.202/2.172 (*a*,*b*)			
S524	8.396 (*d*, $^3J_{NH\text{-}\alpha H}$ = 7.303 Hz)	4.358	3.767				
F525	8.331 (*d*, $^3J_{NH\text{-}\alpha H}$ = 5.202 Hz)	4.562	3.052				Ar= 7.322, 7.311 7.289, 7.208, 7.208
R526	8.08 (*d*, $^3J_{NH\text{-}\alpha H}$ = 7.202 Hz)	4.227	1.658/1.597 (*a*,*b*)	1.503	3.114		NH_ε= 7.294 NH_η= 7.166
D527	8.563 (*d*, $^3J_{NH\text{-}\alpha H}$ = 5.002 Hz)	4.676	2.724/2.526 (*a*,*b*)				
P528		4.323	2.292	2.022/1.974	3.908/3.841		
A529	8.511 (*d*, $^3J_{NH\text{-}\alpha H}$ = 6.102 Hz)	4.22	1.363				
E530	8.103 (*d*, $^3J_{NH\text{-}\alpha H}$ = 6.252 Hz)	4.175	1.973/2.020 (*a*,*b*)	2.269/2.196 (*a*,*b*)			
V531	8.181 (*d*, $^3J_{NH\text{-}\alpha H}$ = 6.703 Hz)	3.991	2.048	0.987			
L532	8.374 (*d*, $^3J_{NH\text{-}\alpha H}$ = 6.102 Hz)	4.229	1.543/1.619 (*a*,*b*)	1.609	0.898/0.838		

Table S7. ^{1}H-NMR assignments in TACC3$_{518\text{-}532}$: [a]One letter code for amino acids. [b]Experimental chemical shifts (ppm) as observed for the peptide in buffer/D_2O 90/10 vol/vol at 5 °C. Buffer: 25 mM potassium phosphate, 50 mM NaCl, 5 mM $MgCl_2$, pH= 7.5.

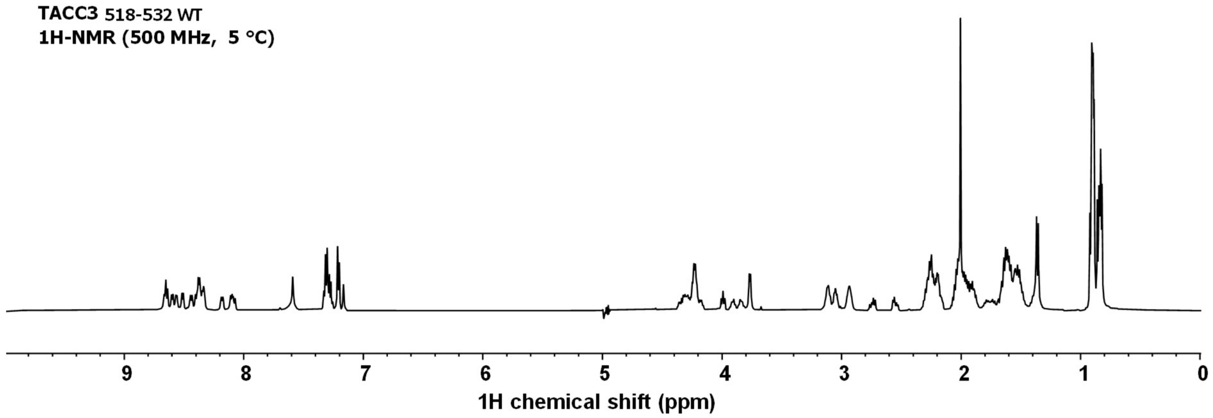

Figure S41. ^{1}H-NMR (500 MHz) trace of TACC3$_{518\text{-}532}$ in buffer/D_2O 90/10 vol/vol at 5 °C. Buffer: 25 mM potassium phosphate, 50 mM NaCl, 5 mM $MgCl_2$, pH= 7.5.

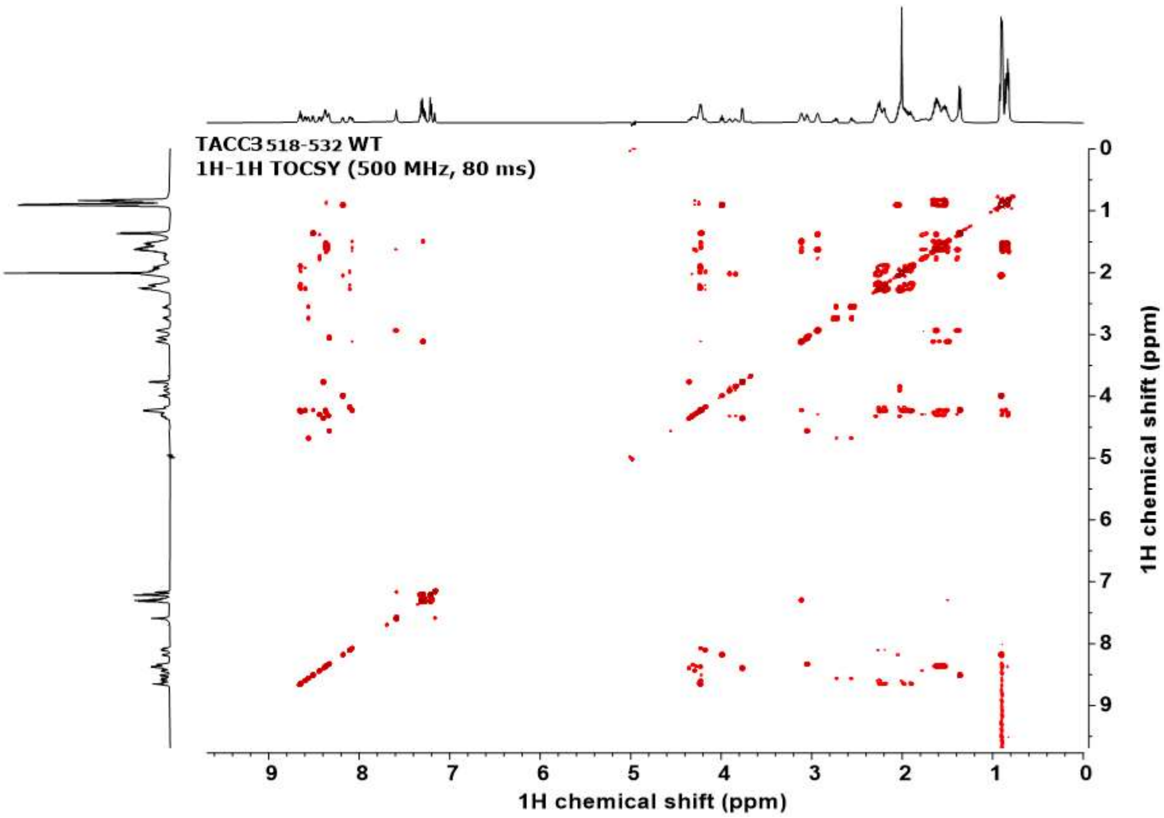

Figure S42. ^{1}H-^{1}H TOCSY NMR (500 MHz) trace of TACC3$_{518\text{-}532}$ in buffer/D_2O 90/10 vol/vol at 5 °C. Buffer: 25 mM potassium phosphate, 50 mM NaCl, 5 mM $MgCl_2$, pH= 7.5.

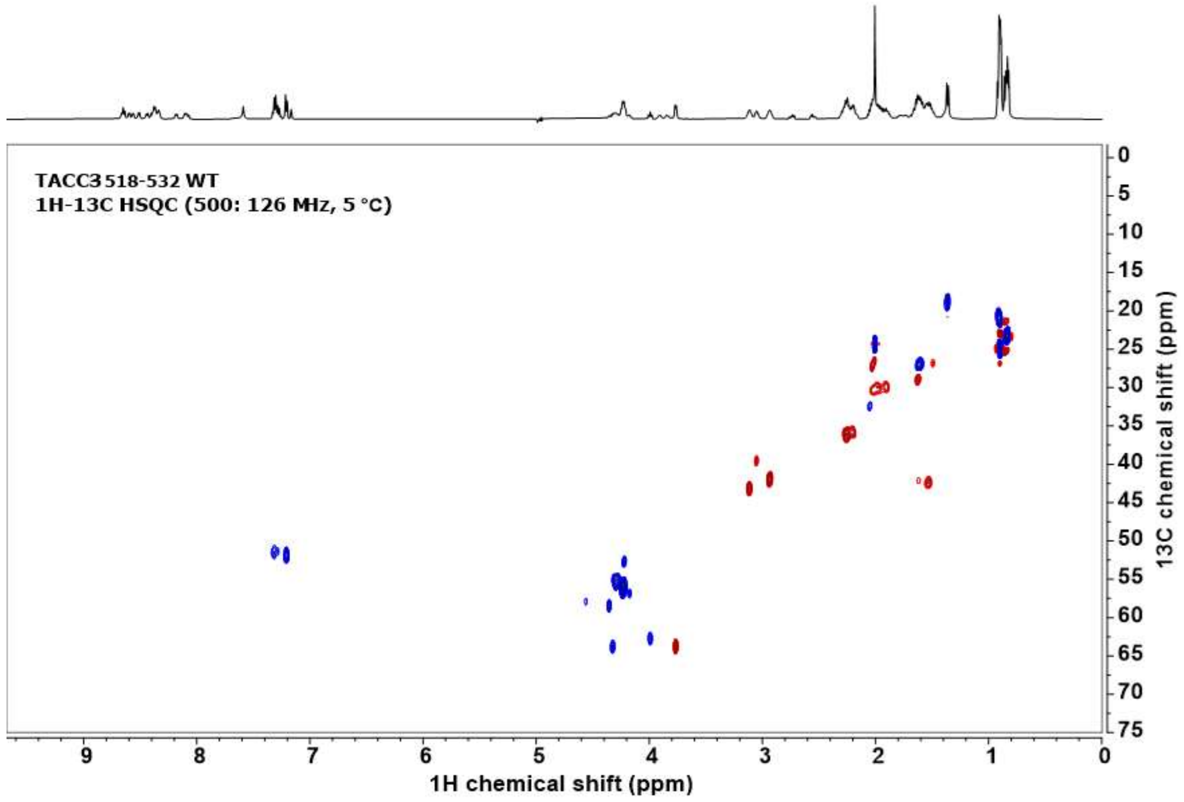

Figure S43. ^{1}H-^{13}C HSQC NMR (500:126 MHz) trace of TACC3$_{518\text{-}532}$ in buffer/D_2O 90/10 vol/vol at 5 °C. Buffer: 25 mM potassium phosphate, 50 mM NaCl, 5 mM $MgCl_2$, pH= 7.5.

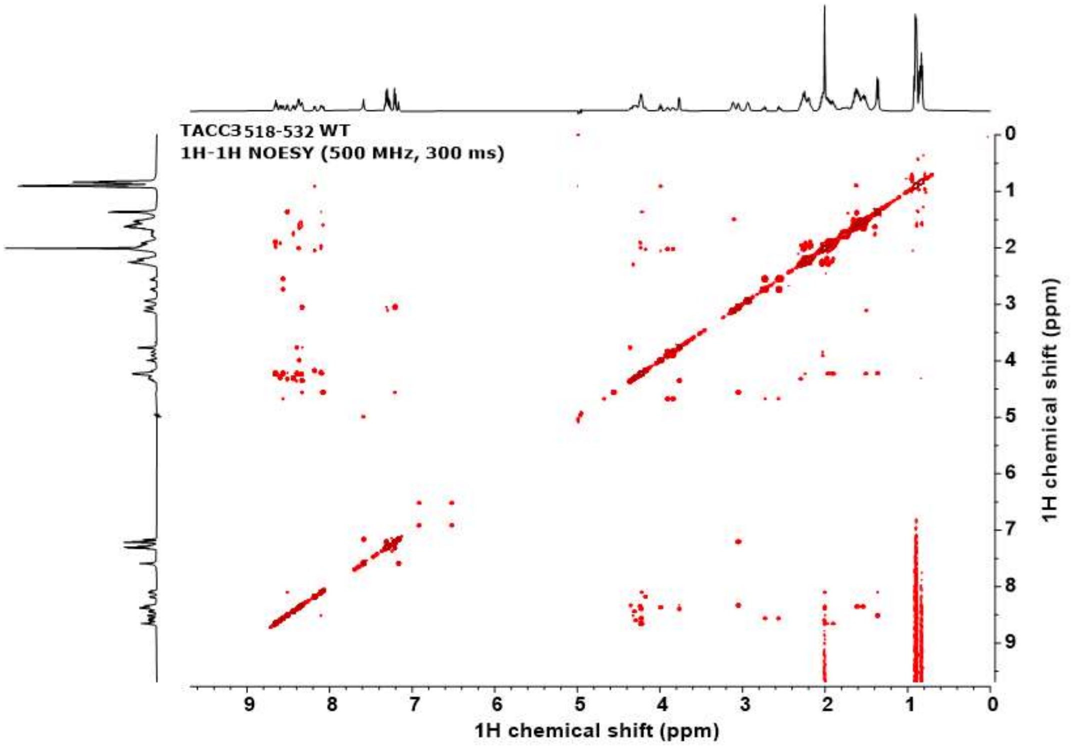

Figure S44. ^{1}H-^{1}H NOESY NMR (500 MHz) trace of TACC3$_{518\text{-}532}$ in buffer/D_2O 90/10 vol/vol at 5 °C. Buffer: 25 mM potassium phosphate, 50 mM NaCl, 5 mM $MgCl_2$, pH= 7.5.

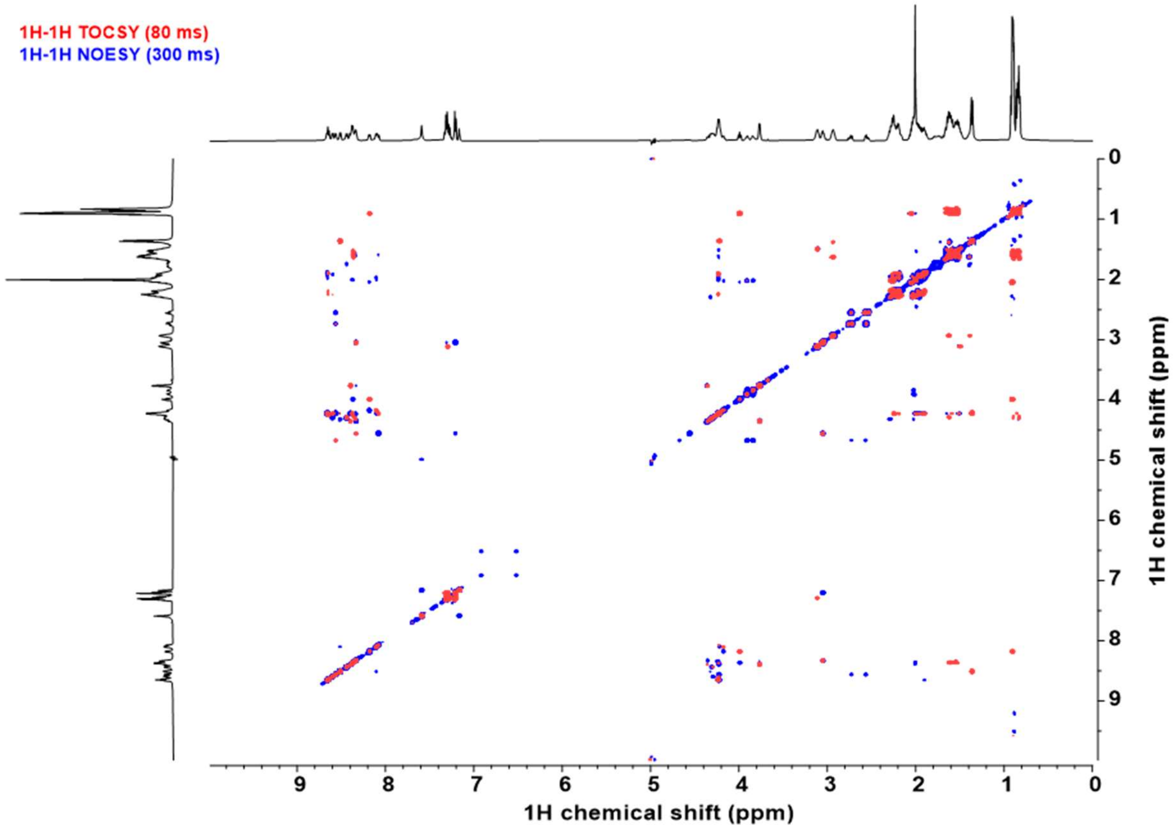

Figure S45. Overlaid ^1H-^1H TOCSY (in red) and ^1H-^1H NOESY spectra (in blue) of TACC3$_{518-532}$ in buffer/D_2O 90/10 vol/vol at 5 °C. Buffer: 25 mM potassium phosphate, 50 mM NaCl, 5 mM $MgCl_2$, pH= 7.5.

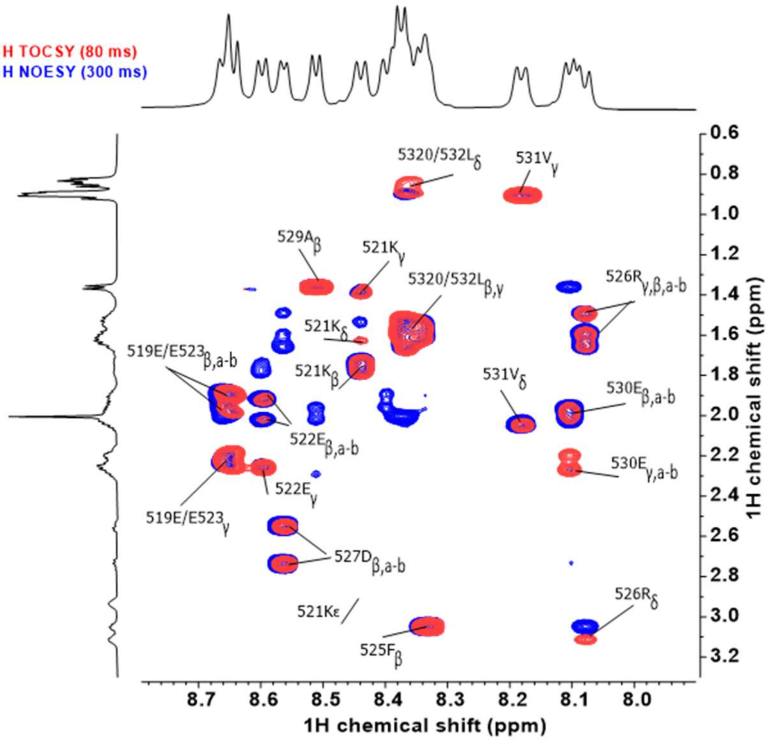

Figure S46. Magnified ^{1}H-^{1}H TOCSY spectra (red) and ^{1}H-^{1}H NOESY spectra (blue) of **TACC3$_{518-532}$** at the amide NH-side chain region showing sequence signal assignments.

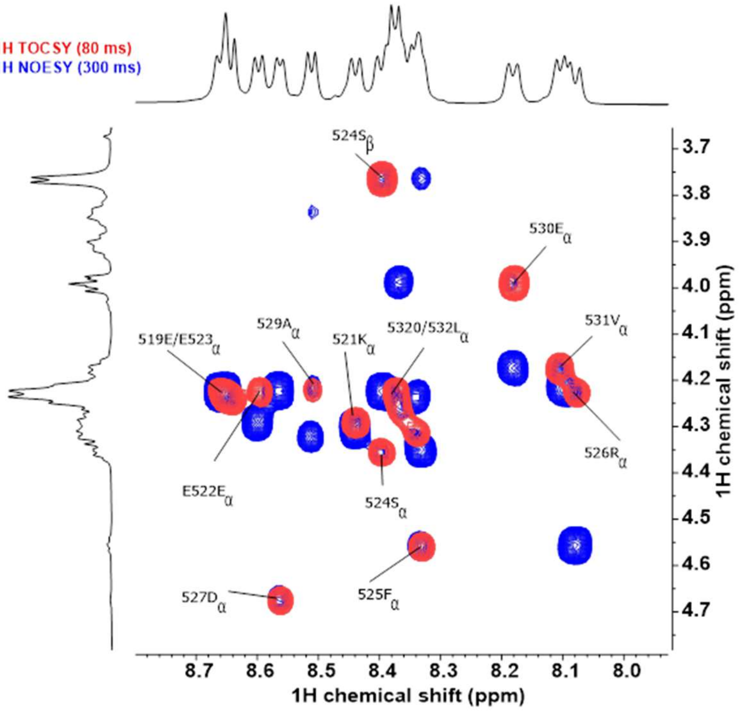

Figure S47. Magnified ^{1}H-^{1}H TOCSY spectra (red) and ^{1}H-^{1}H NOESY spectra (blue) of **TACC3$_{518-532}$** at the amide NH-Hα region showing ^{1}H signal assignments.

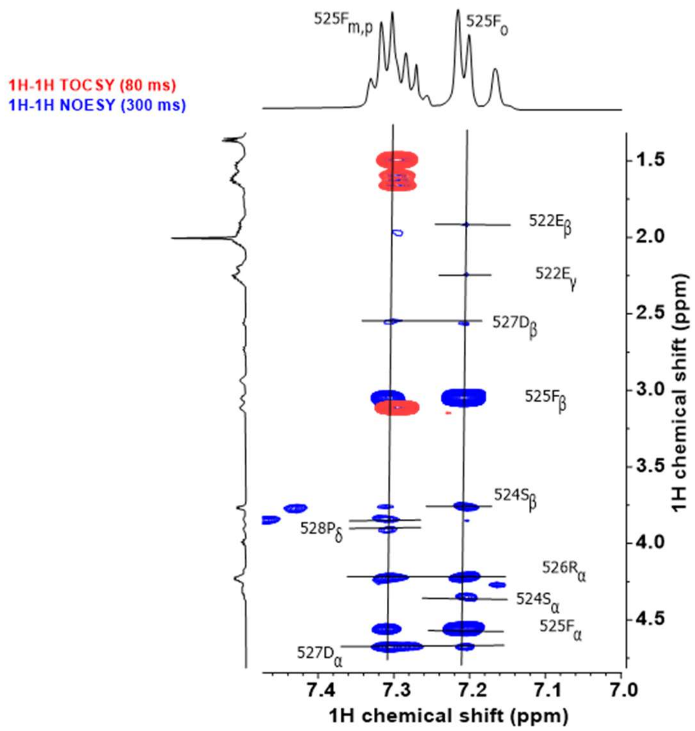

Figure S48. Magnified ^{1}H-^{1}H TOCSY spectra (red) and ^{1}H-^{1}H NOESY spectra (blue) of **TACC3$_{518\text{-}532}$** at the ^{525}F aromatic region.

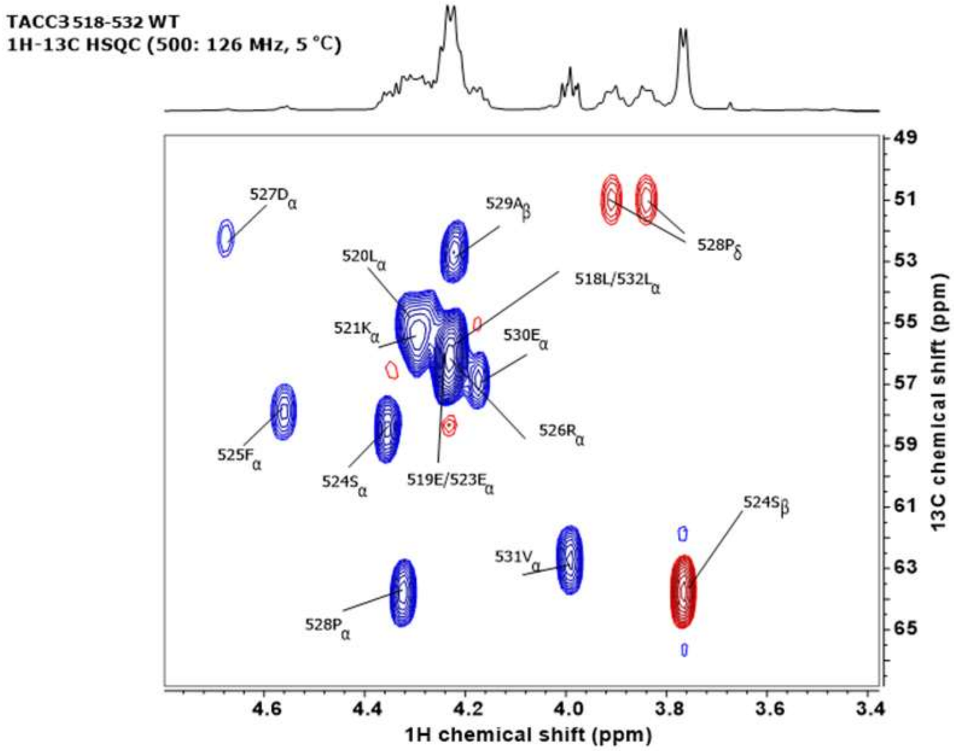

Figure S49. Magnified insert of the ^{1}H-^{13}C HSQC spectra of **TACC3$_{518\text{-}532}$** at the Cα region showing ^{13}C signal assignments.

NMR Peptide secondary structure in solution: Secondary $\Delta\delta_{C\alpha}$, $\Delta\delta_{H\alpha}$ and $\Delta\delta_{NH}$ Chemical Shifts (SCS)

The secondary chemical shift, $\Delta\delta_{C\alpha}$, experienced by the ^{13}C-NMR resonances of the α-carbon in proteins can be correlated to their backbone torsional angle *psi*, which dictates the orientation of the α-proton to the adjacent carbonyl group. In general, when compared to their chemical shift positions within a random coil structure ($\delta_{C\alpha,RC}$), Cα carbons in β-sheet regions experience shifts to lower chemical shift values ($\Delta\delta_{C\alpha} < 0$), whereas those in α-helical regions experience positive secondary shifts ($\Delta\delta_{C\alpha} > 0$).[3–5] The magnitude and sign of the changes experienced can be used as a predictive tool to assess the structural propensity of a peptide, with regions showing a continuous series of downfield Cα secondary shifts $\Delta\delta_{C\alpha} > 2$ indicating propensity for helix-formation. Within helices, opposing correlations have been found for the ^{1}H-NMR resonances of amide protons, H_N, and α-carbon protons, H_α, so regions of negative secondary chemical shifts, $\Delta\delta_{H\alpha}$ and $\Delta\delta_{HN}$, are expected for a peptide with helical propensity.

TACC3 518-532 WT Residue[a]	$\delta_{C\alpha,RC}$[b]	$\delta_{C\alpha}$, obs[c]	$\Delta\delta_{C\alpha}$[d]	$\delta_{H\alpha,RC}$ [b]	$\delta_{H\alpha}$, obs[c]	$\Delta\delta_{H\alpha}$[d]	$\delta_{HN,RC}$ [b]	δ_{HN}, obs[c]	$\Delta\delta_{HN}$[d]
L518	55.43	55.55	0.12	4.328	4.229	-0.108	8.469	8.374	-0.095
E519	56.462	56.53	0.068	4.276	4.235	-0.033	8.588	8.658	0.07
L520	55.119	55.01	-0.109	4.351	4.316	-0.131	8.371	8.342	-0.029
K521	56.372	55.72	-0.652	4.313	4.292	0.01	8.453	8.439	-0.014
E522	56.686	55.92	-0.766	4.278	4.224	-0.065	8.537	8.597	0.06
E523	56.965	56.53	-0.435	4.253	4.235	-0.03	8.609	8.644	0.035
S524	58.557	58.45	-0.107	4.364	4.358	-0.091	8.492	8.396	-0.096
F525	57.689	57.88	0.191	4.705	4.562	-0.076	8.369	8.331	-0.038
R526	55.908	56.02	0.112	4.304	4.227	-0.629	8.199	8.08	-0.119
D527	52.376	52.21	-0.166	4.834	4.676	-0.247	8.457	8.563	0.106
P528	63.215	63.86	0.645	4.429	4.323	-0.202	***	***	
A529	52.698	52.64	-0.058	4.25	4.22	-0.027	8.509	8.511	0.002
E530	56.551	56.73	0.179	4.28	4.175	-0.03	8.527	8.103	-0.424
V531	62.34	62.71	0.37	4.09	3.991	-0.029	8.343	8.181	-0.162
L532	55.154	55.55	0.396	4.353	4.229	-0.108	8.493	8.374	-0.119

Table S8. Secondary chemical shifts in TACC3$_{518-532}$. [a] One letter code for amino acids. [b] Theoretical random coil $\delta_{C\alpha,RC}$, $\delta_{H\alpha,RC}$ and $\delta_{NH,RC}$ chemical shifts according to https://www1.bio.ku.dk/english/research/bms/sbinlab/randomchemicalshifts2/,[6–8] as calculated for TACC3 $_{518-532}$ at 5 °C and pH= 7.5. [c] Observed experimental Cα, Hα and amide HN chemical shifts (ppm). [d] Calculated secondary ($\delta_{RC} - \delta_{obs}$) $\Delta\delta_{C\alpha}$, $\Delta\delta_{H\alpha}$ and $\Delta\delta_{NH}$ chemical shifts (ppm).

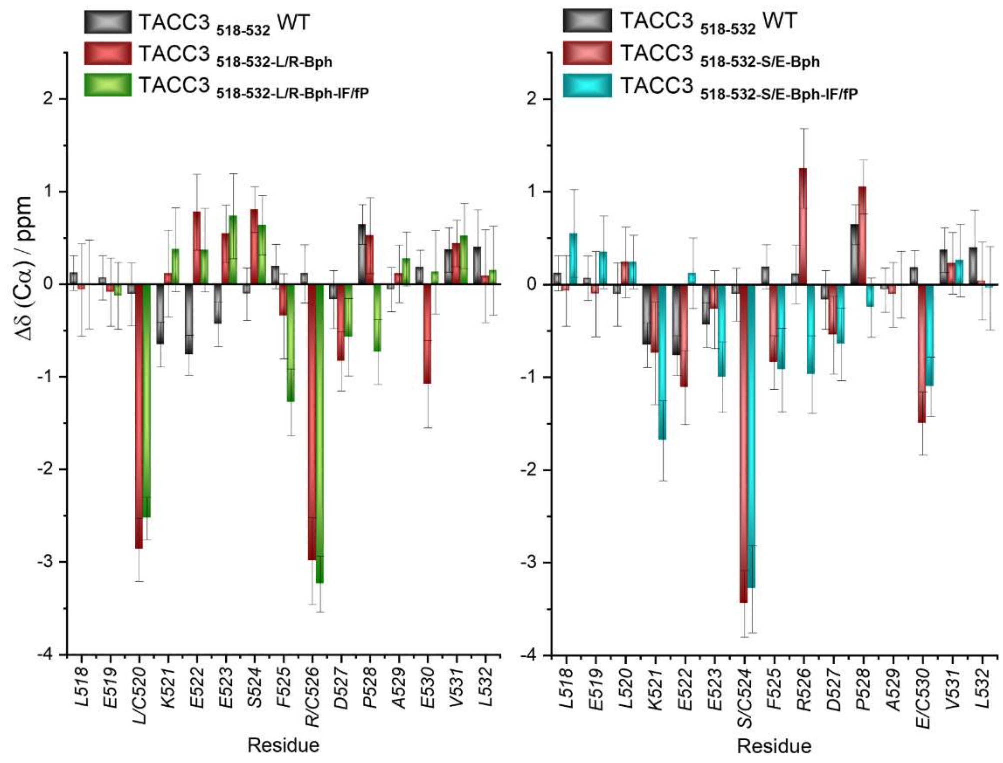

Figure S50. **Secondary ΔδCα chemical shifts by residue calculated for $TACC3_{518\text{-}532}$ and Bph- constrained variants based on their ^{1}H and $^{1}H\text{-}^{13}C$ HSQC NMR spectra.**

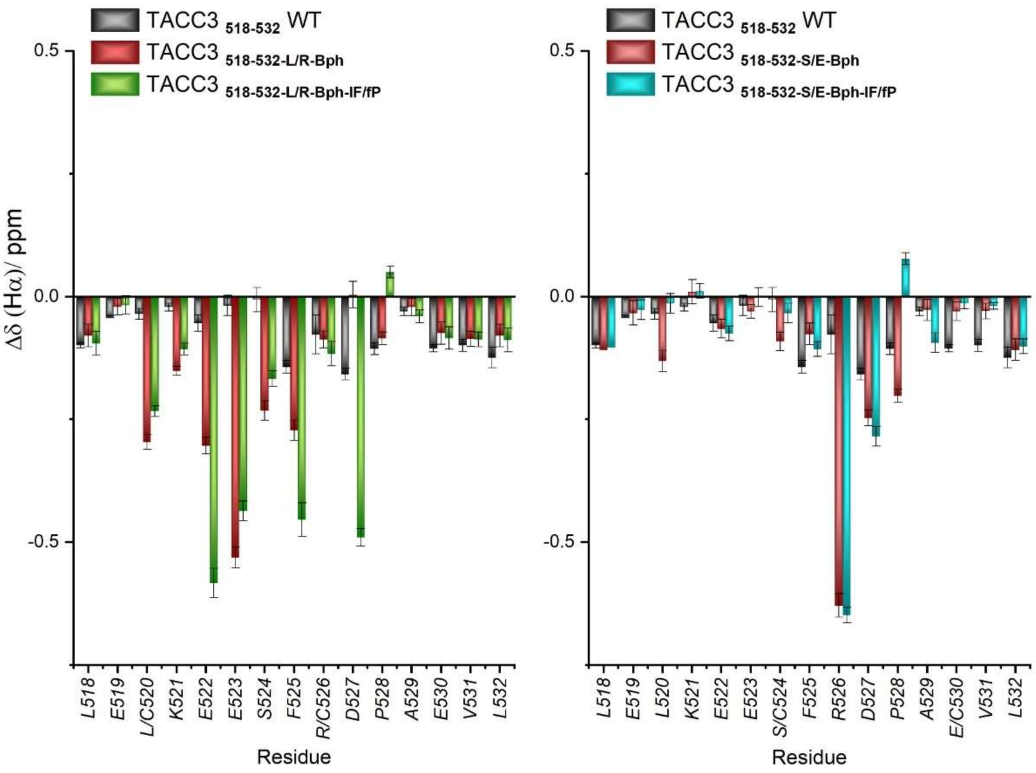

Figure S51. **Secondary ΔδHα chemical shifts by residue calculated for $TACC3_{518\text{-}532}$ and Bph- constrained variants as based on their ^{1}H and $^{1}H\text{-}^{13}C$ HSQC NMR spectra.**

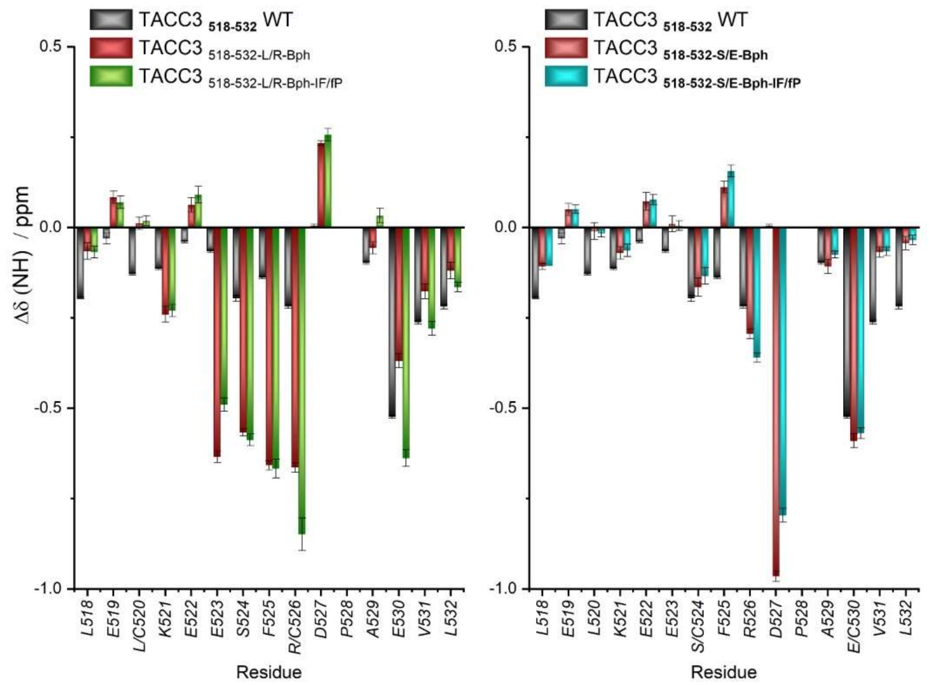

Figure S52. **Secondary ΔδN$_H$ chemical shifts by residue calculated for TACC3$_{518\text{-}532}$ and Bph- constrained variants as based in their ^{1}H and ^{1}H-^{13}C HSQC NMR spectra.**

NMR analysis: TACC3$_{518-532}$ Leu520Cys/Arg526Cys Bph constrained (4-I)Phe525/*trans*-(4-F)Pro528 variant

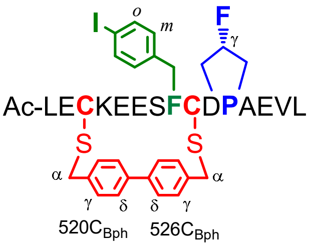

TACC3 $_{518-532-LR-Bph-I/fP}$ Residue[a]	$\delta_{NH,obs}$[b]	$\delta_{H\alpha,obs}$[b]	$\delta_{H\beta,obs}$[b]	$\delta_{H\gamma,obs}$[b]	$\delta_{H\delta,obs}$[b]	$\delta_{H\varepsilon,obs}$[b]	others
L518-	8.398	4.258	1.608	1.608	0.878		
E519-	8.694	4.285	1.993/1.924 (*a*,*b*)	2.241			
C520-	8.532	4.278	2.799				Bph_α= 3.852<br>Bph_γ= 7.446<br>Bph_δ= 7.561
K521	8.368	4.216	1.718	1.353	1.814	2.609	NH_ε= 7.164
E522-	8.647	3.7	1.835	2.172			
E523-	8.119	3.817	1.649	1.849			
S524-	7.901	4.216	3.693				
(4-F)F525-	7.747	4.285	2.943				Ar_o= 7.536<br>Ar_m = 6.856
C526-	8.002	4.367	2.661				Bph_α= 3.693<br>Bph_γ= 7.371<br>Bph_δ= 7.561
D527-	8.749	4.358	2.826/2.606 (*a*,*b*)				
P528		4.492	2.715/2.210 (A) (*a*,*b*)<br>2.694/2.126 (B) (*a*,*b*)	5.507 (A)<br>5.404 (B)	4.082 (A)<br>4.005 (B)		
A529-	8.542	4.21	1.374				
E530-	7.889	4.196	2.069/2.021 (*a*,*b*)	2.269/2.20 (*a*,*b*)			
V531-	8.064	4.003	2.055	0.899			
L532-	8.328	4.265	1.642/1.518 (*a*,*b*)	1.642	0.830		

Table S9. ^{1}H-NMR assignments in TACC3$_{518-532-LR-Bph-IF/fP}$. [a]One letter code for amino acids. [b]Experimental chemical shifts (ppm) as observed for the peptide in buffer/D_2O 90/10 vol/vol at 5 °C. Buffer: 25 mM potassium phosphate, 50 mM NaCl, 5 mM $MgCl_2$, and pH= 7.5.

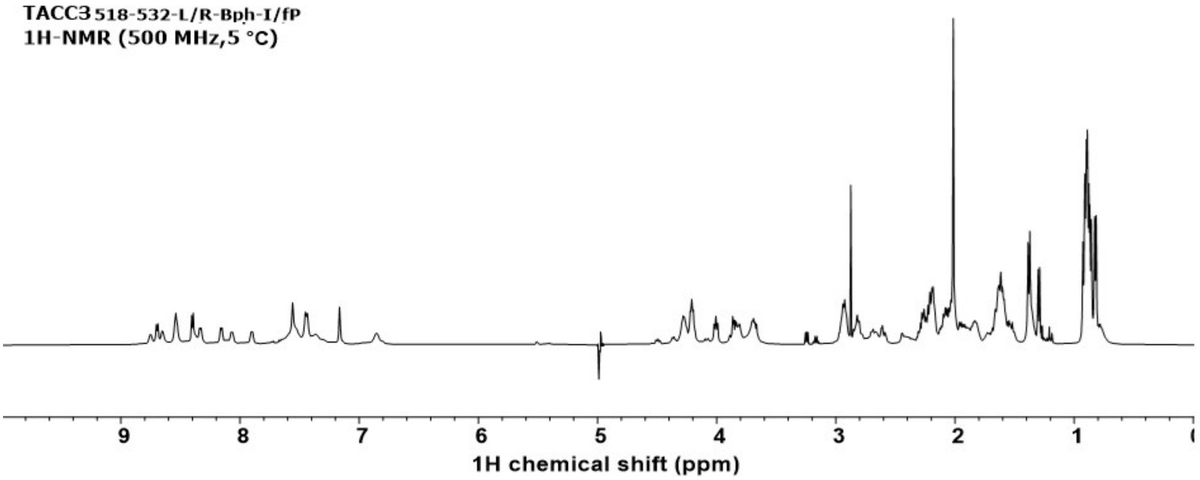

Figure S53. ^{1}H-NMR (500 MHz) trace of TACC3$_{518\text{-}532\text{-}L/R\text{-}Bph\text{-}I/fP}$ in buffer/D_2O 90/10 vol/vol at 5 °C. Buffer: 25 mM potassium phosphate, 50 mM NaCl, 5 mM $MgCl_2$, pH= 7.5.

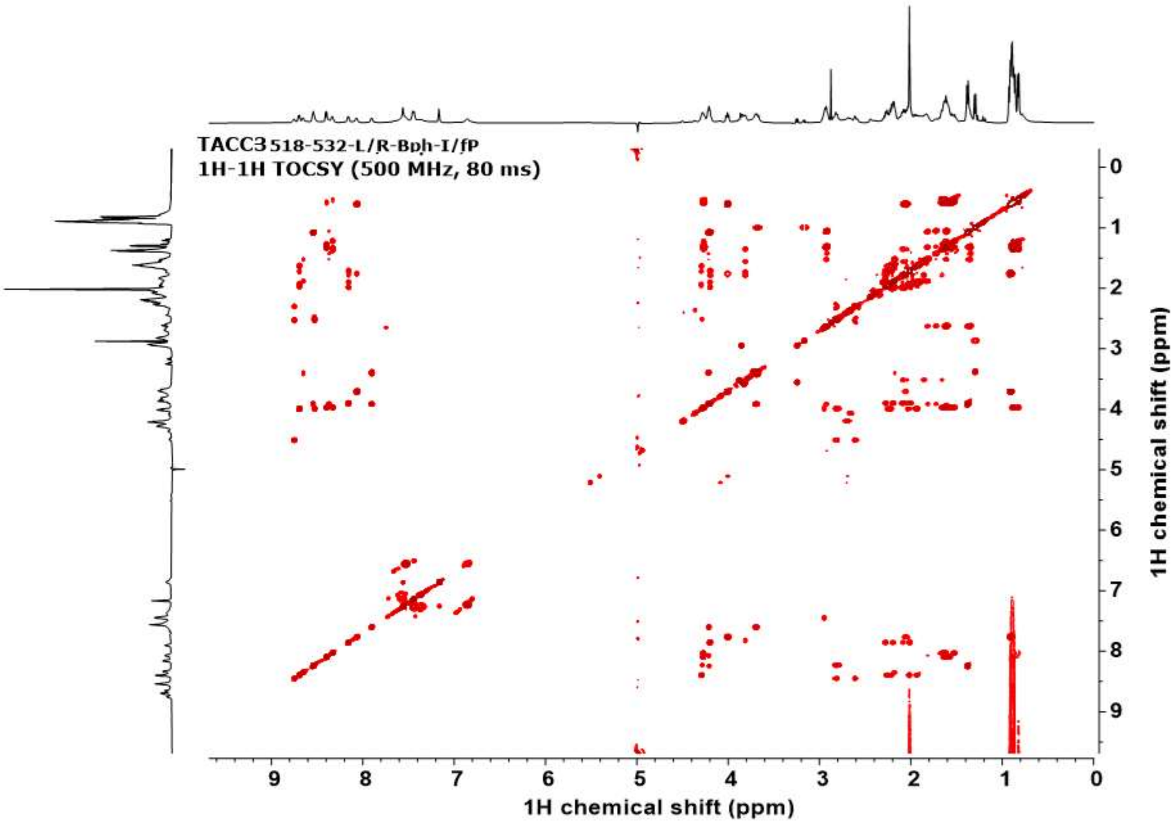

Figure S54. ^{1}H-^{1}H TOCSY NMR (500 MHz) trace of TACC3$_{518\text{-}532\text{-}L/R\text{-}Bph\text{-}I/fP}$ in buffer/D_2O 90/10 vol/vol at 5 °C. Buffer: 25 mM potassium phosphate, 50 mM NaCl, 5 mM $MgCl_2$, pH= 7.5.

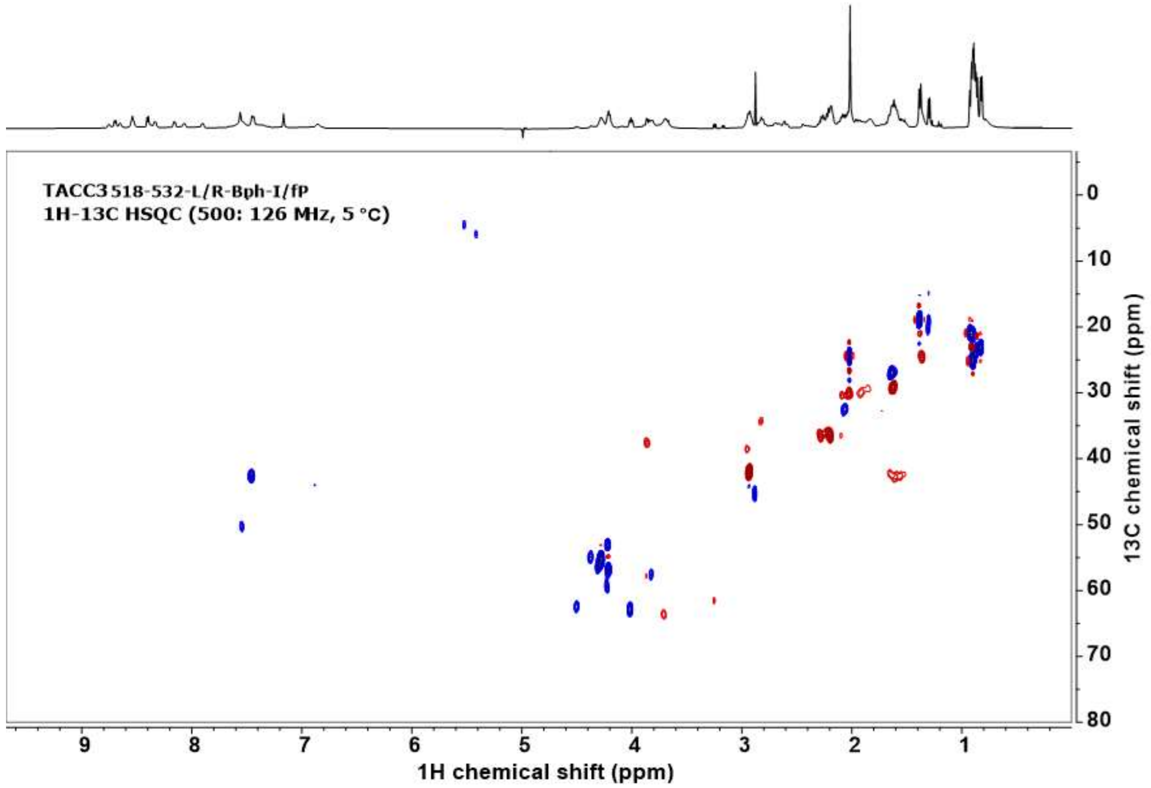

Figure S55. ^{1}H-^{13}C HSQC NMR (500:126 MHz) trace of TACC3$_{518\text{-}532\text{-}L/R\text{-}Bph\text{-}I/fP}$ in buffer/D_2O 90/10 vol/vol at 5 °C. Buffer: 25 mM potassium phosphate, 50 mM NaCl, 5 mM $MgCl_2$, pH= 7.5.

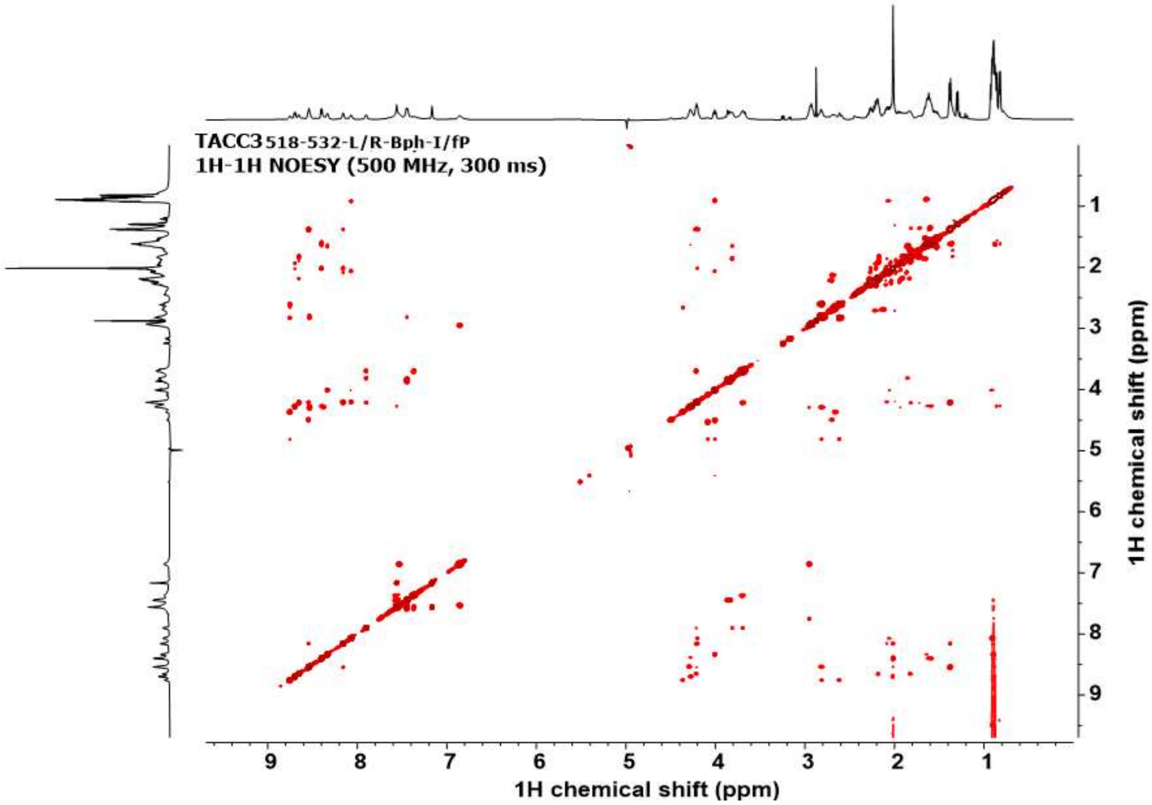

Figure S56. ^{1}H-^{1}H NOESY NMR (500 MHz) trace of TACC3$_{518\text{-}532\text{-}L/R\text{-}Bph\text{-}I/fP}$ in buffer/D_2O 90/10 vol/vol at 5 °C. Buffer: 25 mM potassium phosphate, 50 mM NaCl, 5 mM $MgCl_2$, pH= 7.5.

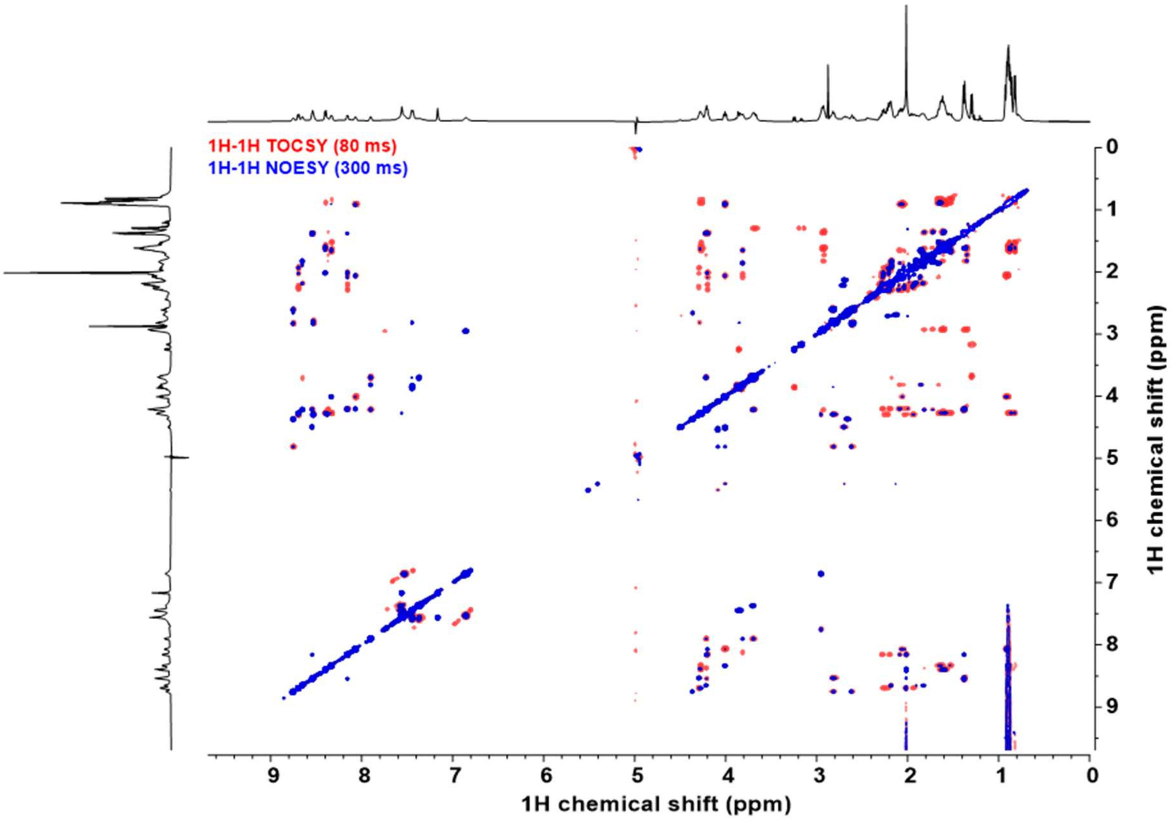

Figure S57. Overlaid ^{1}H-^{1}H TOCSY (in red) and ^{1}H-^{1}H NOESY spectra (in blue) of TACC3$_{518\text{-}532\text{-}L/R\text{-}Bph\text{-}I/fP}$ in buffer/D_2O 90/10 vol/vol at 5 °C. Buffer: 25 mM potassium phosphate, 50 mM NaCl, 5 mM $MgCl_2$, pH= 7.5.

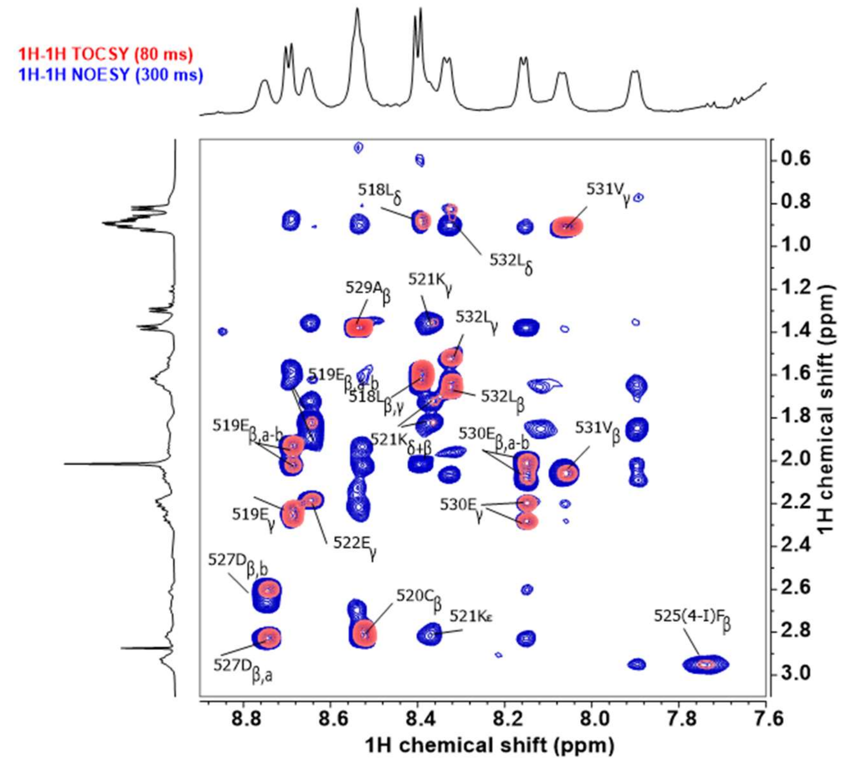

Figure S58. Magnified ^{1}H-^{1}H TOCSY spectra (red) and ^{1}H-^{1}H NOESY spectra (blue) of $\mathbf{TACC3}_{\mathbf{518\text{-}532\text{-}L/R\text{-}Bph\text{-}I/fP}}$ at the amide NH-side chain region showing sequence signal assignments.

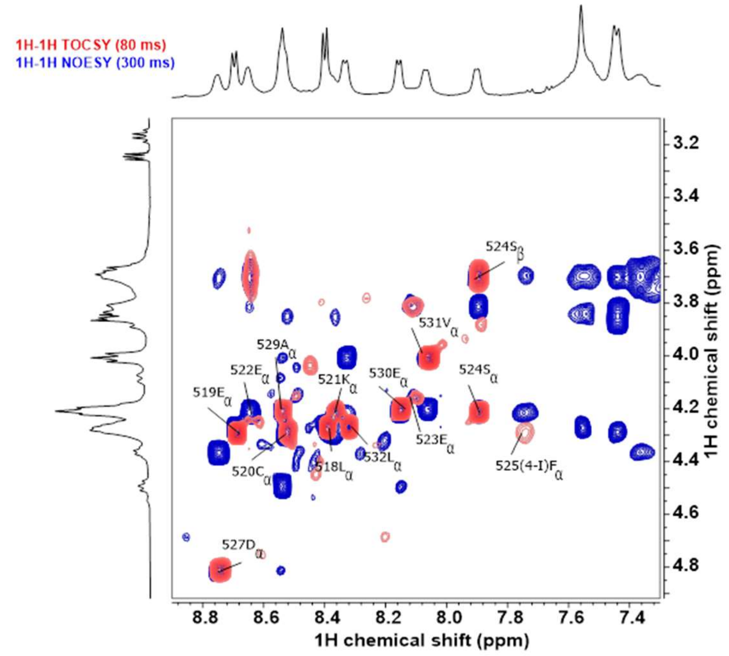

Figure S59. Magnified ^{1}H-^{1}H TOCSY spectra (red) and ^{1}H-^{1}H NOESY spectra (blue) of $\mathbf{TACC3}_{\mathbf{518\text{-}532\text{-}L/R\text{-}Bph\text{-}I/fP}}$ at the amide NH-Hα region showing ^{1}H signal assignments.

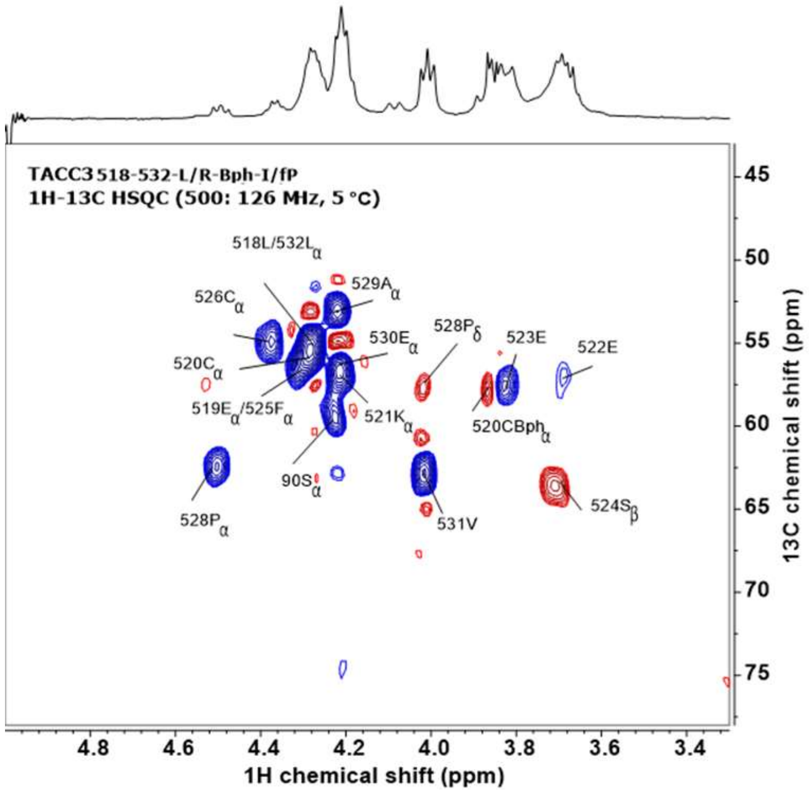

Figure S60. Magnified insert of the ^{1}H-^{13}C HSQC spectra of **TACC3$_{518\text{-}532\text{-}L/R\text{-}Bph\text{-}I/fP}$** at the Cα region showing ^{13}C signal assignments.

^{1}H (VT)-NMR of TACC3$_{518\text{-}532}$ Leu520Cys/Arg526Cys Bph- constrained (4-I)Phe525/*trans*-(4-F)Pro528 variant

VT-NMR experiments were carried out on the constrained peptides for two reasons. First to corroborate that broadened resonances observed for certain residues that seemed close to coalescence were associated with different conformers (as opposed to different compounds) in particular for this variant res Phe525, Glu523, Cys526, and the Bph- group and secondly to probe the thermal stability of the constrained peptides. As shown below, upon increasing the temperature from 5 °C to 25 °C there was (i) a clear increase in the overall signal intensity for key residues (including the Bph constraint) and (ii) general sharpening of the ^{1}H resonances indicating improved averaging of the different amide-bond populations. The characteristic ^{1}H-NMR resonance intensity and splitting of the Bph group was also restored upon increasing the temperature, indicating a restricted conformational environment for this group at 5 °C. In addition, the thermal stability of the peptide was found to be relatively high, as fast-exchange/average of most of the amide-bond protons with the solvent was not observed up to temperatures higher than 35-40°C.

VT-NMR experiments also allowed us to corroborate and monitor the efficacy of the stereoelectronic effects at the trans-(4-F)Pro528 residue. As shown in Fig. S1 and S62(a-b), we observed two well-resolved Hγ protons for the proline residue, corresponding to the *exo*-pucker/*trans*-amide conformer at higher chemical shift ($\delta H_{\gamma,exo}$= 5.5 ppm) and the *endo*-pucker/*cis*-amide isomer at a lower chemical shift, as elsewhere observed ($\delta H_{\gamma,endo}$= 5.4 ppm).[9] Upon increasing the tempearure the thermal energy increases and the energy gap between isomers decreases (i.e. equal population of exo/endo conformers). Notwithstanding this, the conformational bias to the *exo*-pucker conformation is still present at biologically relevant temperatures and up to approx. 50 °C.

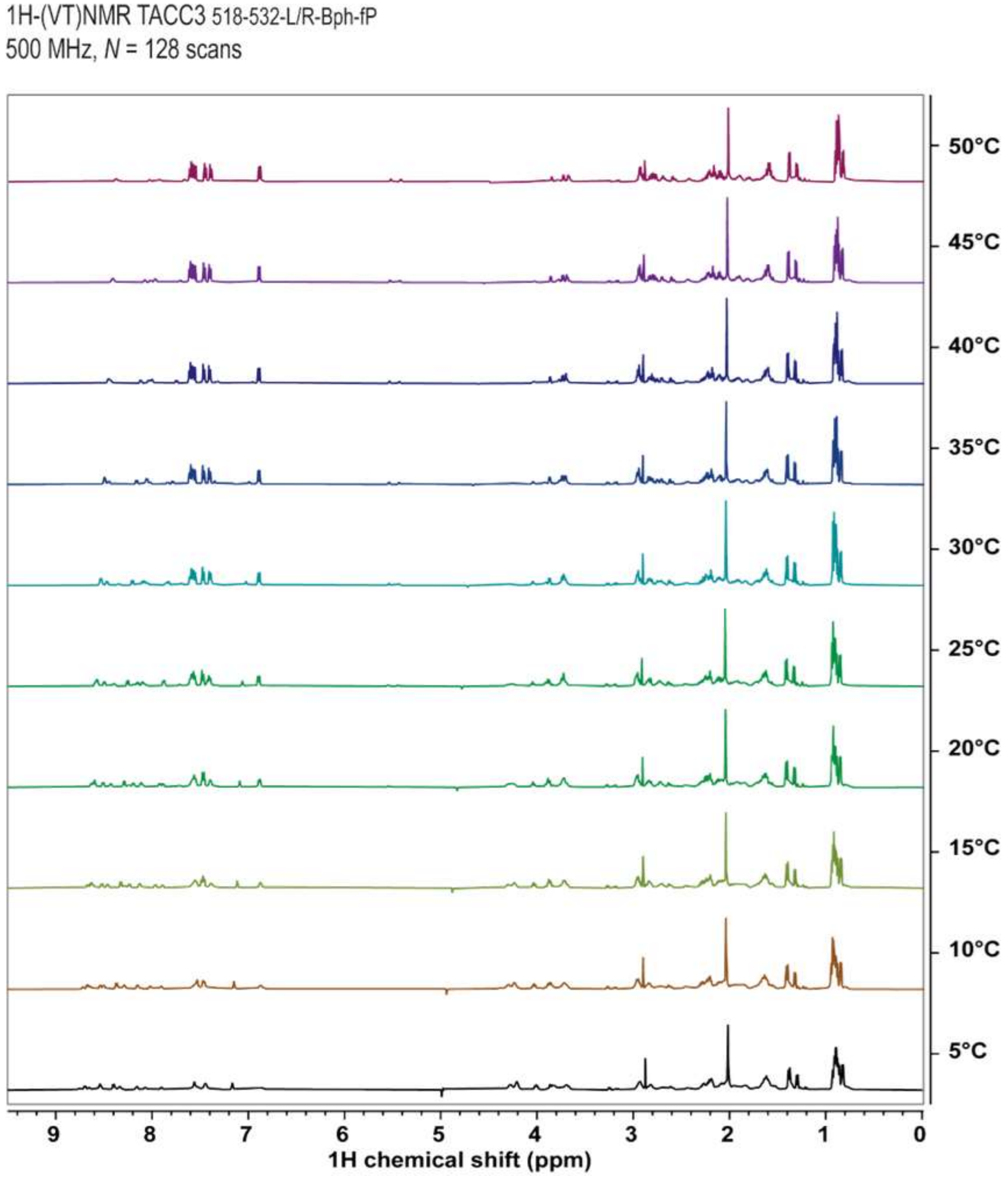

Figure S61. ^{1}H-VT NMR (500 MHz) traces observed for TACC3$_{518\text{-}532_LR_Bph_lF/fP}$ at increasing temperatures from 5 - 50 °C. Sample in 25 mM Tris, 150 mM NaCl, 5 mM $MgCl_2$, pH 7.5/ D_2O 90/10 v/v.

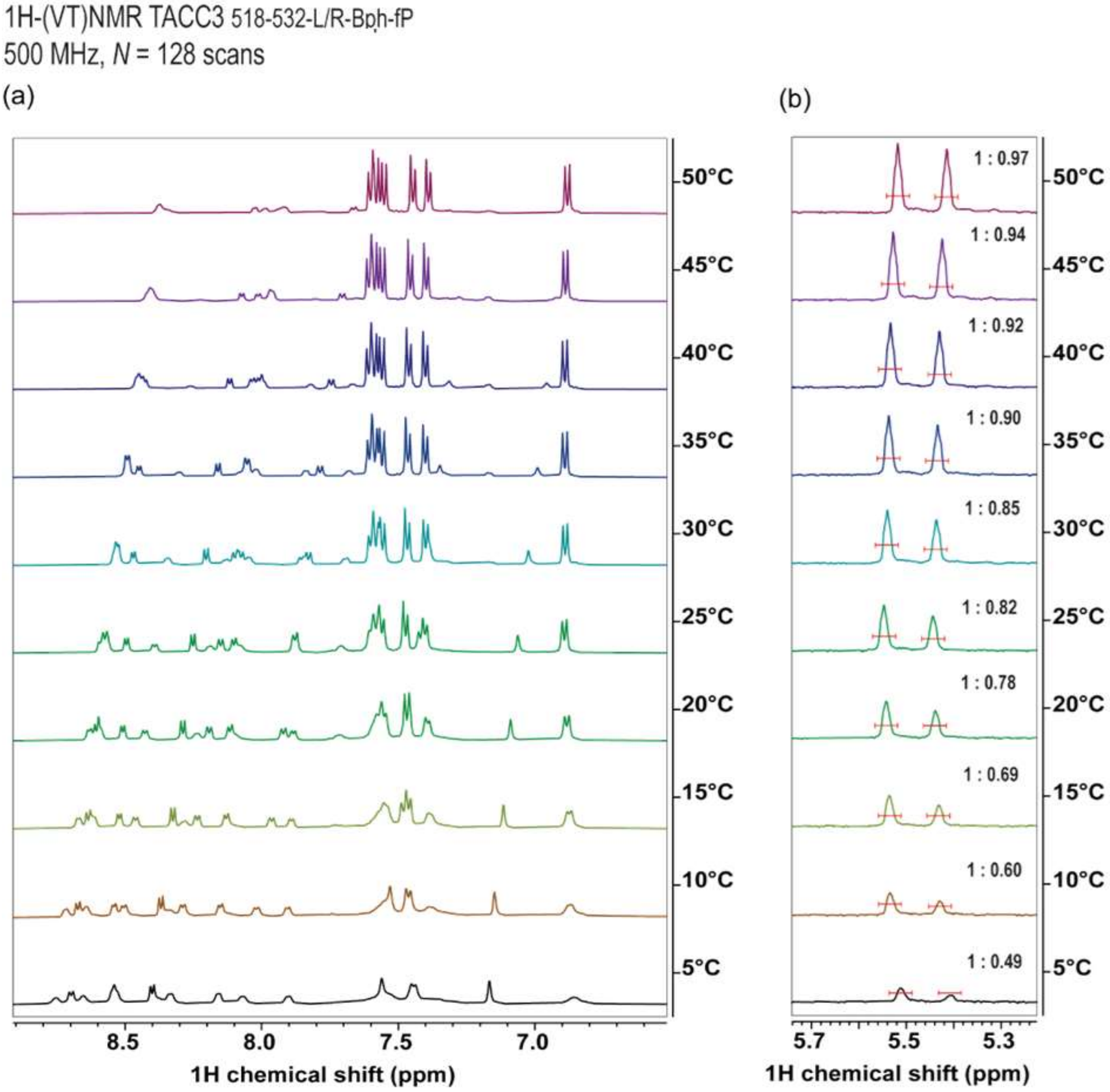

Figure S62. Insets of the ^{1}H-VT NMR (500 MHz) traces observed for TACC3$_{518\text{-}532_LR_Bph_IF/fP}$ at the amide NH region (a) and (b) (4-F)P528 Hγ proton region. Sample in 25 mM Tris, 150 mM NaCl, 5 mM $MgCl_2$, pH= 7.5/ D_2O 90/10 v/v.

TACC3 $_{518\text{-}532\text{-}L/R\text{-}Bph\text{-}IF/fP}$ Residue[a]	$\delta_{C\alpha,RC}$[b]	$\delta_{C\alpha, obs}$[c]	$\Delta\delta_{C\alpha}$[d]	$\delta_{H\alpha,RC}$[b]	$\delta_{H\alpha, obs}$[c]	$\Delta\delta_{H\alpha}$[d]	$\delta_{HN,RC}$[b]	$\delta_{HN, obs}$[c]	$\Delta\delta_{HN}$[d]
L518	55.303	55.3	-0.003	4.353	4.258	-0.095	8.466	8.398	-0.068
E519	56.656	56.53	-0.126	4.302	4.285	-0.017	8.624	8.694	0.07
**C520***	58.399	55.87	-2.529	4.511	4.278	-0.233	8.513	8.532	0.019
K521	56.606	56.98	0.374	4.323	4.216	-0.107	8.598	8.368	-0.23
E522	56.761	57.13	0.369	4.283	3.7	-0.583	8.556	8.647	0.091
E523	56.965	57.7	0.735	4.253	3.817	-0.436	8.609	8.119	-0.49
S524	58.514	59.15	0.636	4.383	4.216	-0.167	8.489	7.901	-0.588
(4-I)F525	57.806	56.53	-1.276	4.739	4.285	-0.454	8.414	7.747	-0.667
**C526***	58.26	55.02	-3.24	4.483	4.367	-0.116	8.216	7.367	-0.849
D527	52.533	51.96	-0.573	4.848	4.358	-0.49	8.492	8.749	0.257
trans-(4-F)P528	63.185	62.45	-0.735	4.442	4.492	0.05	***	***	***
A529	52.698	52.97	0.272	4.25	4.21	-0.04	8.509	8.542	0.033
E530	56.551	56.68	0.129	4.28	4.196	-0.084	8.527	7.889	-0.638
V531	62.34	62.86	0.52	4.09	4.003	-0.087	8.343	8.064	-0.279
L532	55.154	55.3	0.146	4.353	4.265	-0.088	8.493	8.328	-0.165

Table S10. Secondary chemical shifts in TACC3$_{518\text{-}532\text{-}L/R\text{-}Bph\text{-}IF/fP}$. [a] One letter code for amino acids. [b] Theoretical random coil $\delta_{C\alpha,RC}$, $\delta_{H\alpha,RC}$ and $\delta_{NH,RC}$ chemical shifts according to https://www1.bio.ku.dk/english/research/bms/sbinlab/randomchemicalshifts2/,[6–8] as calculated for TACC3 $_{518\text{-}532}$ at 5 °C and pH= 7.5. [c] Observed experimental Cα, Hα and amide HN chemical shifts (ppm). [d]Calculated secondary (δ_{RC} - δ_{obs}) $\Delta\delta_{C\alpha}$, $\Delta\delta_{H\alpha}$ and $\Delta\delta_{NH}$ chemical shifts (ppm). Note that in the absence of an appropriate reference for the constrained modified aminoacids, these have been calculated referenced to the chemical shift of a Cys residue. Values for these residues might be misrepresented and might be considered only orientative

^{1}H-(VT)-NMR: Conformational preference at Pro528

To evaluate to what extent the introduction of the fluorine-atom affects the conformational distribution of proline conformers, and estimate the temperature range in which the exo-pucker conformational preference is maintained, we calculated the corresponding free energy barrier for *exo/endo* isomer interconversion ($\Delta G_{exo/endo}$) at different temperatures. This can be assessed directly from the ^{1}H-NMR experiments, using the integrated ratios measured for the Hγ protons for each one of the isomers present according to:

$$\Delta G_{exo/endo} = -RT\ln[Q]$$

Where $\Delta G_{exo/endo}$ is the free energy for *endo/exo* interconversion at each given temperature; [Q] is the reaction quotient, R is the universal gas constant (1.987×10^{-3} kcal/mol K), and T is the temperature in kelvin. For each temperature at the equilibrium:

$$[Q] = K_{exo/endo} = \frac{Area_{\ isomer-ex}}{Area_{\ isomer-endo}}$$

Note that $\Delta G_{exo/endo}$ expresses the energy gap between isomers at each given temperature, and varies with T and differs from $\Delta G°$ (standard free energy difference between conformers) according to:

$$\Delta G_{exo/endo} = \Delta G° + RT\ln[Q]$$

and

$$\Delta G° = \Delta H° - T\Delta S° = -RT\ \ln[K_{exo/endo}]$$

For both constrained peptides, we measured a sustained preference for the *exo*-pucker conformation up to temperatures of 323 K (50 °C). As commonly observed, increasing the temperature resulted in smaller free energy barriers for conformer interconversion, as the internal energy contribution due to the entropic term increases, facilitating isomerization.

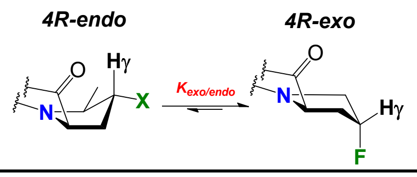

	TACC3 518-532-LR-Bph-IF/fP		TACC3 518-532-SE-Bph-IF/fP	
**T (K)**	$K_{exo/endo}$[a]	$\Delta G_{exo/endo}$ (kcal mol^{-1}) [b]	$K_{exo/endo}$ [a]	$\Delta G_{exo/endo}$ (kcal mol^{-1}) [b]
**278**	**2.041**	**-0.394**	**2.703**	**-0.549**
283	1.667	-0.287	2.000	-0.390
288	1.449	-0.212	1.667	-0.292
293.	1.282	-0.145	1.515	-0.242
**298**	**1.220**	**-0.118**	**1.389**	**-0.195**
303	1.163	-0.091	1.266	-0.142
308	1.111	-0.064	1.220	-0.121
313	1.087	-0.052	1.163	-0.094
318	1.064	-0.039	1.124	-0.074
323	1.031	-0.020	1.099	-0.061

Table S11. Proline pucker preference in constrained variants TACC3$_{518\text{-}532\text{-}L/R\text{-}Bph_IF/fP}$ and TACC3$_{518\text{-}532\text{-}S/E\text{-}Bph_IF/fP}$. [a] $K_{exo/endo}$ values as estimated from the corresponding integrated ratios between $H_{\gamma,exo}$ and $H_{\gamma,endo}$ protons signals at the given temperature. [b] $\Delta G_{exo/endo}$ calculated according to $\Delta G_{exo/endo}$= -R T Ln ($K_{exo/endo}$).

NMR analysis: Control TACC3 $_{518\text{-}532}$ Leu520Cys/Arg526Cys Bph constrained variant

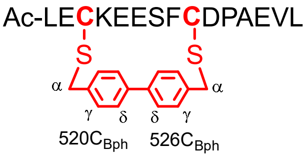

TACC3 $_{518\text{-}532\text{-}LR\text{-}Bph}$ Residue[a]	$\delta_{NH,obs}$[b]	$\delta_{H\alpha,obs}$[b]	$\delta_{H\beta,obs}$[b]	$\delta_{H\gamma,obs}$[b]	$\delta_{H\delta,obs}$[b]	$\delta_{H\varepsilon,obs}$[b]	others
L518-	8.401	4.274	1.615	1.621	0.897		
E519-	8.708	4.282	2.025/1.948 (*a*,*b*)	2.264			
C520-	8.525	4.215	2.822/2.736				Bph_α= 3.854<br>Bph_γ= 7.468<br>Bph_δ= 7.630
K521-	8.358	4.172	1.80/1.736	1.346	1.609	3.009/2.920	NH_ε= 7.596
E522-	8.619	3.98	2.008/1.902	2.226			
E523-	7.975	3.722	1.806/1.557	2.024/1.967			
S524-	7.922	4.151	3.592				
F525-	7.756	4.467	3.139/2.948				Ar= 7.260, 7.236<br>7.167
C526-	7.552	4.396	2.736/2.628				Bph_α= 3.775<br>Bph_γ= 7.429<br>Bph_δ= 7.746
D527-	8.726	4.852	2.804/2.528 (*a*,*b*)				
P528-		4.357	2.284	2.015	3.921/3.849		
A529-	8.453	4.229	1.383				
E530-	8.158	4.206	2.055/2.005 (*a*,*b*)	2.279/2.198 (*a*,*b*)			
V531-	8.166	4.004	2.049	0.904/0.907			
L532-	8.374	4.274	1.641/1.539 (*a*,*b*)	1.643	0.866/0.823		

Table S12. ^{1}H-NMR assignments in TACC3$_{518\text{-}532\text{-}L/R\text{-}Bph}$. [a]One letter code for amino acids. [b]Experimental chemical shifts (ppm) as observed for the peptide in buffer/D_2O 90/10 vol/vol at 5 °C. Buffer: 25 mM potassium phosphate, 50 mM NaCl, 5 mM $MgCl_2$, pH= 7.5 °C and pH= 7.5.

TACC3 $_{518\text{-}532\text{-}L/R\text{-}Bph}$ Residue[a]	$\delta_{C\alpha,RC}$[b]	$\delta_{C\alpha}$, obs[c]	$\Delta\delta_{C\alpha}$[d]	$\delta_{H\alpha,RC}$ [b]	$\delta_{H\alpha}$, obs[c]	$\Delta\delta_{H\alpha}$[d]	$\delta_{HN,RC}$ [b]	δ_{HN}, obs[c]	$\Delta\delta_{HN}$[d]
L518	55.303	55.24	-0.063	4.353	4.274	-0.079	8.466	8.401	-0.065
E519	56.656	56.57	-0.086	4.302	4.282	-0.02	8.624	8.708	0.084
**C520***	58.399	55.53	-2.869	4.511	4.215	-0.296	8.513	8.525	0.012
K521	56.606	56.72	0.114	4.323	4.172	-0.151	8.598	8.358	-0.24
E522	56.761	57.54	0.779	4.283	3.98	-0.303	8.556	8.619	0.063
E523	56.965	57.51	0.545	4.253	3.722	-0.531	8.609	7.975	-0.634
S524	58.514	59.32	0.806	4.383	4.151	-0.232	8.489	7.922	-0.567
F525	57.806	57.46	-0.346	4.739	4.467	-0.272	8.414	7.756	-0.658
**C526***	58.26	55.27	-2.99	4.483	4.396	-0.087	8.216	7.552	-0.664
D527	52.533	51.7	-0.833	4.848	4.852	0.004	8.492	8.726	0.234
P528	63.185	63.71	0.525	4.442	4.357	-0.085	***	***	***
A529	52.698	52.81	0.112	4.25	4.229	-0.021	8.509	8.453	-0.056
E530	56.551	55.47	-1.081	4.28	4.206	-0.074	8.527	8.158	-0.369
V531	62.34	62.78	0.44	4.09	4.004	-0.086	8.343	8.166	-0.177
L532	55.154	55.24	0.086	4.353	4.274	-0.079	8.493	8.374	-0.119

Table S13. Secondary chemical shifts in control TACC3$_{518\text{-}532\text{-}L/R\text{-}Bph}$. [a] One letter code for amino acids. [b] Theoretical random coil $\delta_{C\alpha,RC}$, $\delta_{H\alpha,RC}$ and $\delta_{NH,RC}$ chemical shifts according to https://www1.bio.ku.dk/english/research/bms/sbinlab/randomchemicalshifts2/,[6–8] as calculated for TACC3 $_{518\text{-}532}$ at 5 °C and pH= 7.5. [c] Observed experimental Cα, Hα and amide HN chemical shifts (ppm). [d]Calculated secondary (δ_{RC} - δ_{obs}) $\Delta\delta_{C\alpha}$, $\Delta\delta_{H\alpha}$ and $\Delta\delta_{NH}$ chemical shifts (ppm). Note that in the absence of an appropriate reference for the constrained modified aminoacids, these have been calculated referenced to the chemical shift of a Cys residue. Values for these residues might be misrepresented and might be considered only orientative.

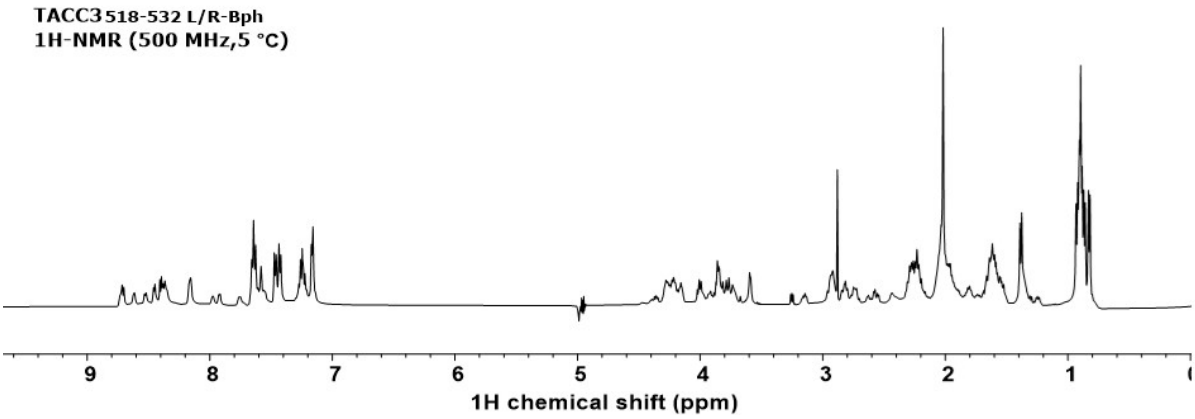

Figure S63. ^{1}H-NMR (500 MHz) trace of TACC3$_{518\text{-}532\text{-}L/R\text{-}Bph}$ in buffer/D_2O 90/10 vol/vol at 5 °C. Buffer: 25 mM potassium phosphate, 50 mM NaCl, 5 mM $MgCl_2$, pH= 7.5.

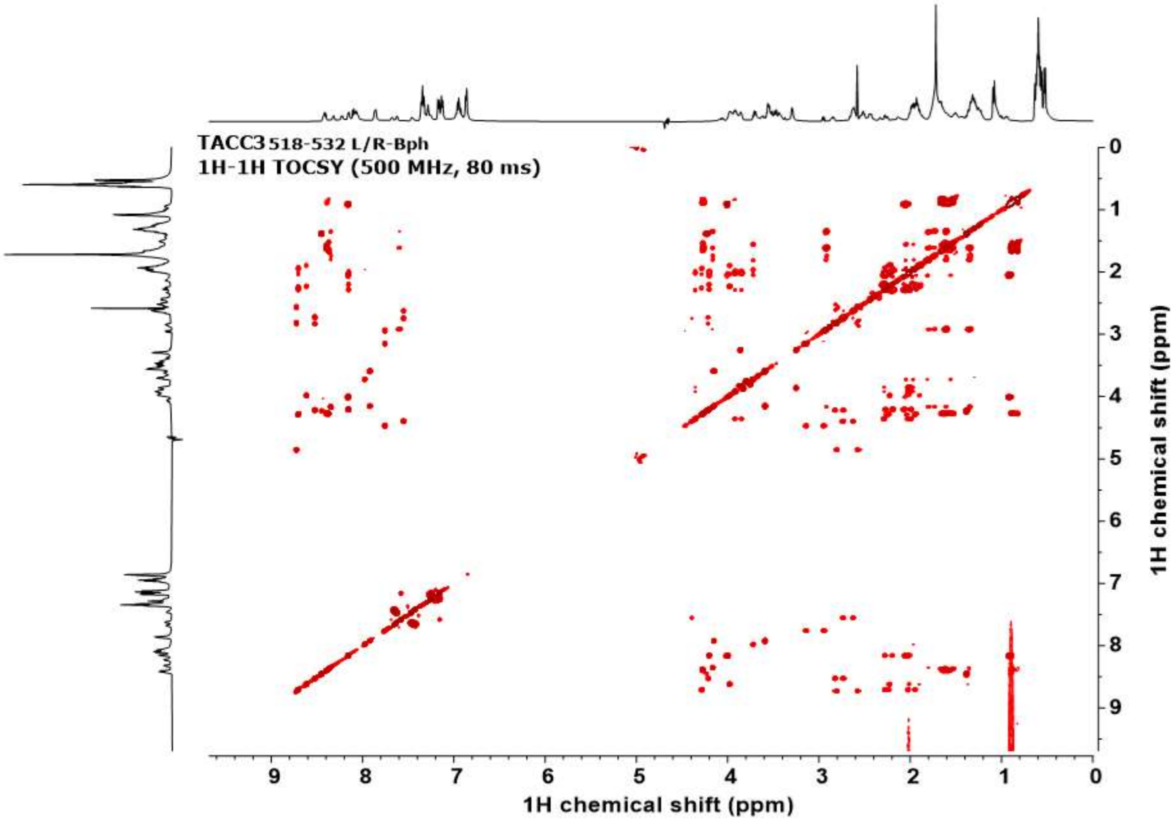

Figure S64. ^{1}H-^{1}H TOCSY NMR (500 MHz) trace of TACC3$_{518\text{-}532\text{-}L/R\text{-}Bph}$ in buffer/D_2O 90/10 vol/vol at 5 °C. Buffer: 25 mM potassium phosphate, 50 mM NaCl, 5 mM $MgCl_2$, pH= 7.5.

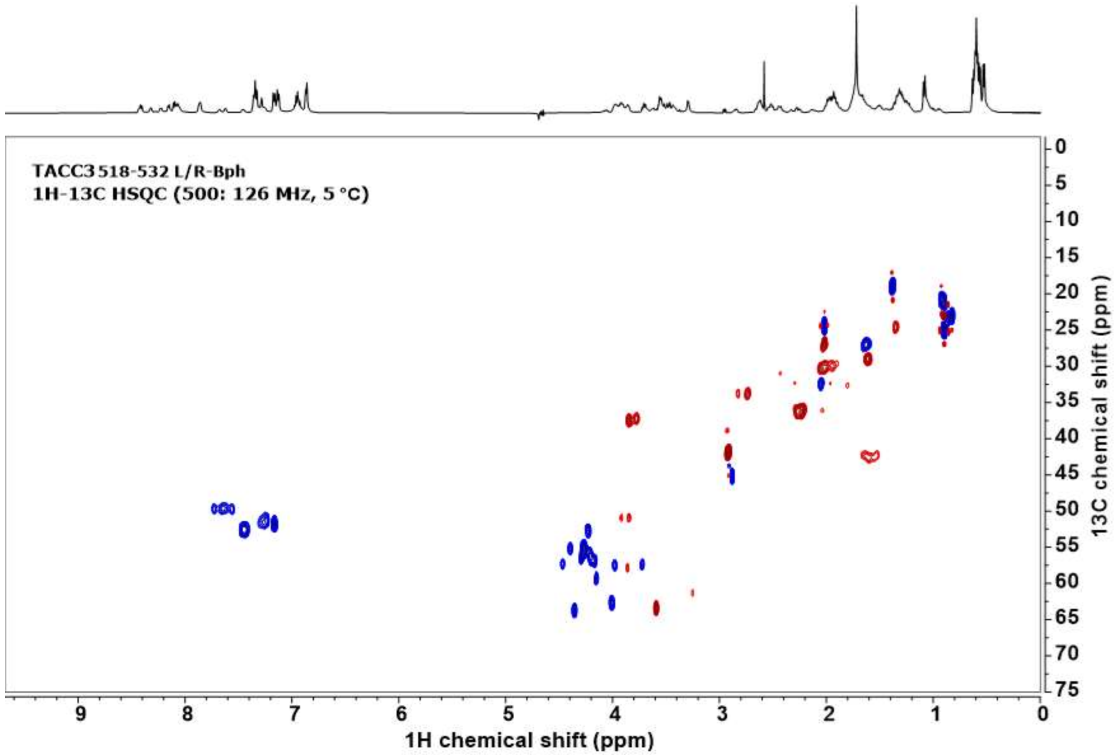

Figure S65. ^{1}H-^{13}C HSQC NMR (500:126 MHz) trace of TACC3$_{518\text{-}532\text{-}L/R\text{-}Bph}$ in buffer/D_2O 90/10 vol/vol at 5 °C. Buffer: 25 mM potassium phosphate, 50 mM NaCl, 5 mM $MgCl_2$, pH= 7.5.

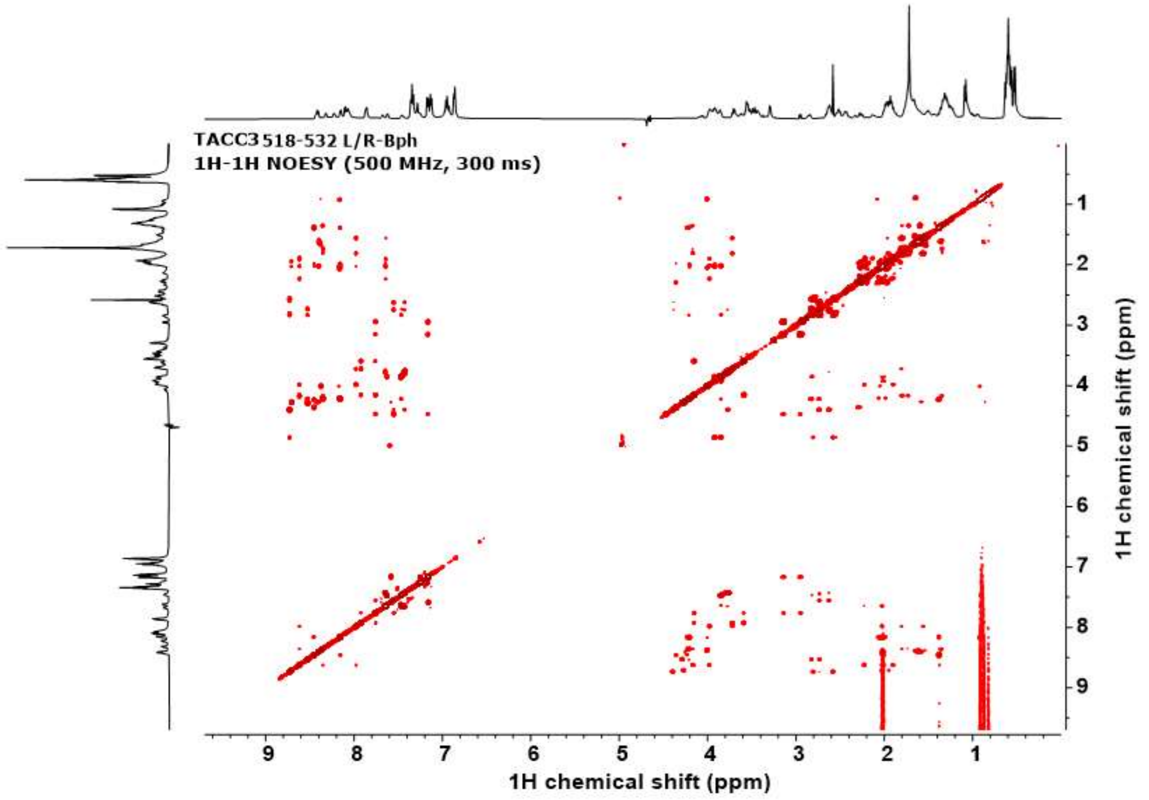

Figure S66. ^{1}H-^{1}H NOESY NMR (500 MHz) trace of TACC3$_{518\text{-}532\text{-}L/R\text{-}Bph}$ in buffer/D_2O 90/10 vol/vol at 5 °C. Buffer: 25 mM potassium phosphate, 50 mM NaCl, 5 mM $MgCl_2$, pH= 7.5.

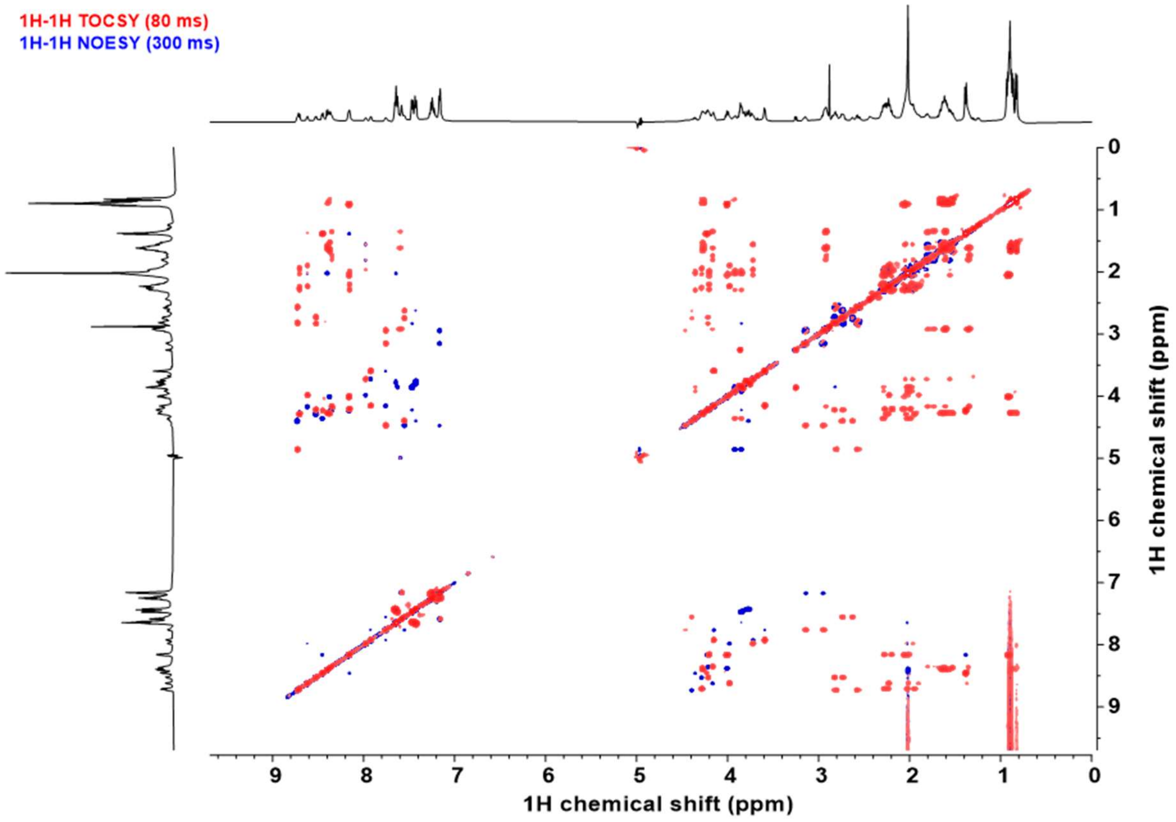

Figure S67. Overlaid ^{1}H-^{1}H TOCSY (in red) and ^{1}H-^{1}H NOESY spectra (in blue) of TACC3$_{518\text{-}532\text{-}L/R\text{-}Bph}$ in buffer/D_2O 90/10 vol/vol at 5 °C. Buffer: 25 mM potassium phosphate, 50 mM NaCl, 5 mM $MgCl_2$, pH= 7.5.

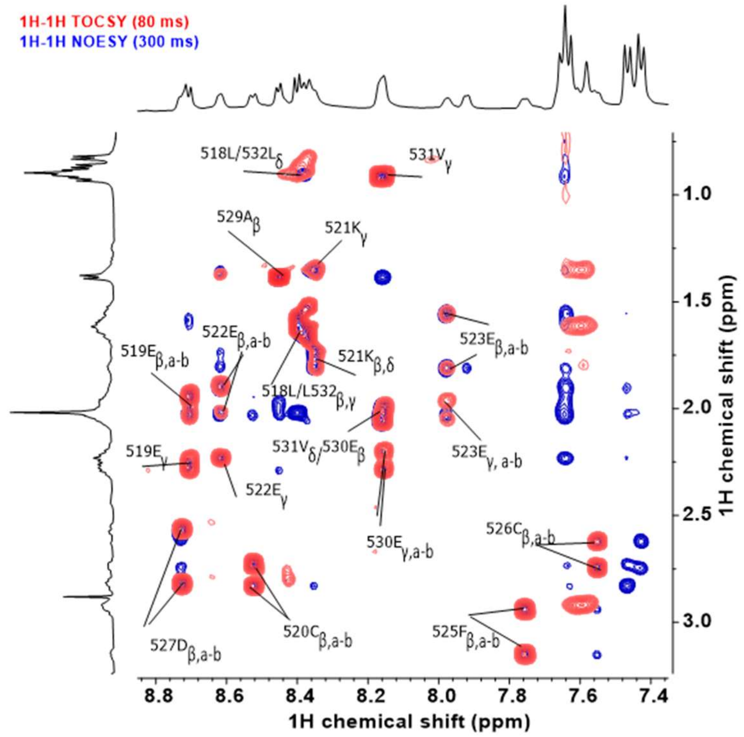

Figure S68. Magnified ^{1}H-^{1}H TOCSY spectra (red) and ^{1}H-^{1}H NOESY spectra (blue) of **TACC3$_{518\text{-}532\text{-}L/R\text{-}Bph}$** at the amide NH-side chain region showing sequence signal assignments.

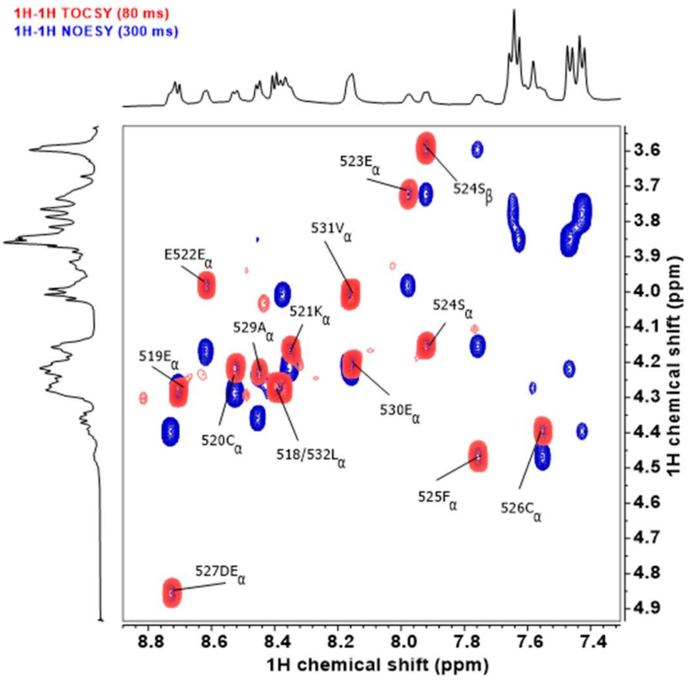

Figure S69. Magnified ^{1}H-^{1}H TOCSY spectra (red) and ^{1}H-^{1}H NOESY spectra (blue) of **TACC3$_{518\text{-}532\text{-}L/R\text{-}Bph}$** at the amide NH-Hα region showing ^{1}H signal assignments.

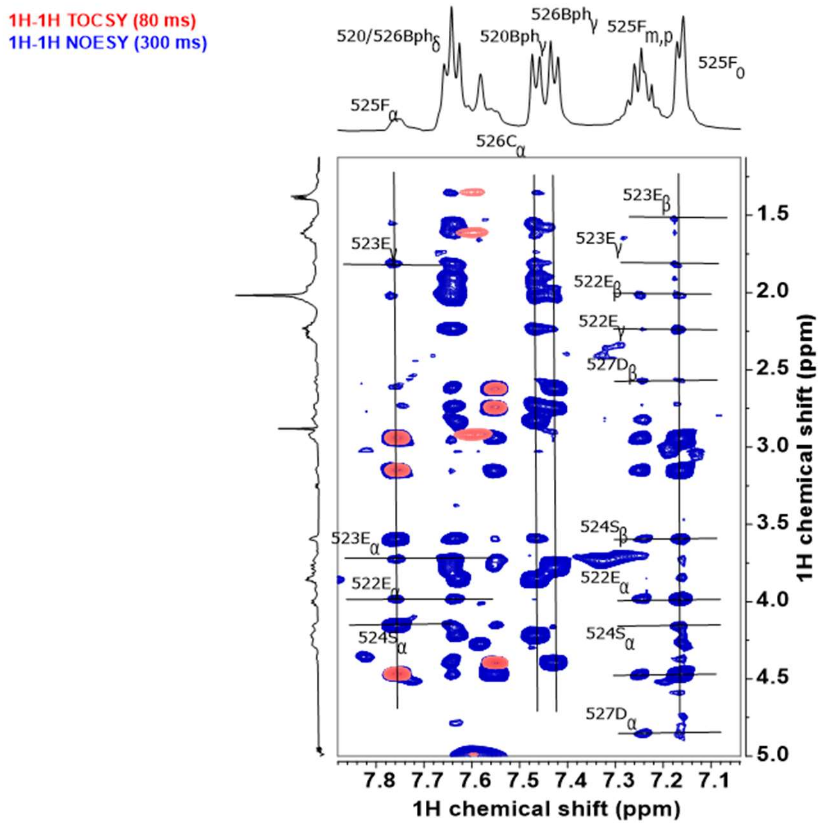

Figure S70. Magnified ^{1}H-^{1}H TOCSY spectra (red) and ^{1}H-^{1}H NOESY spectra (blue) of **TACC3$_{518\text{-}532\text{-}L/R\text{-}Bph}$** at the ^{525}F aromatic region.

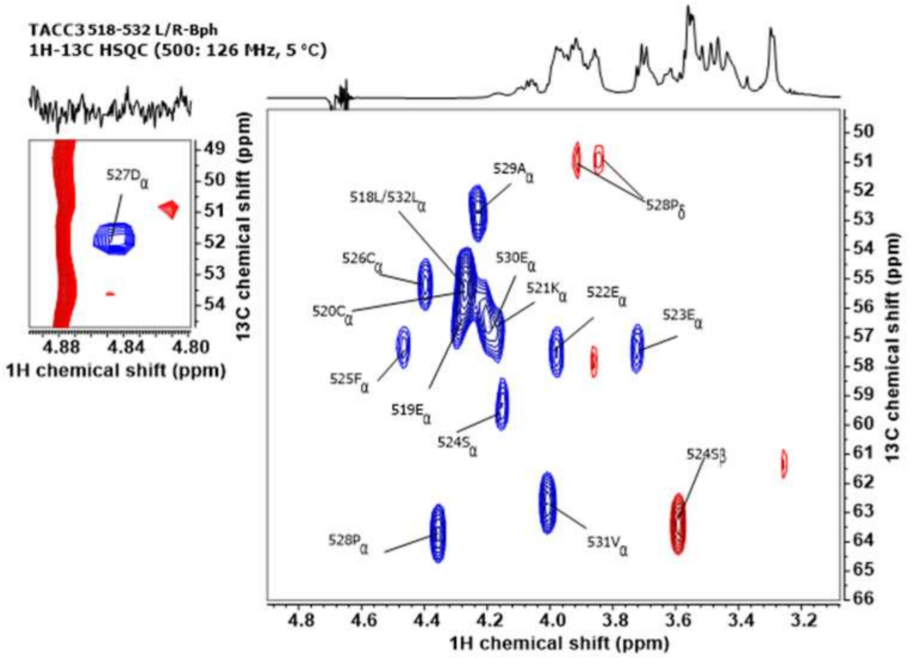

Figure S71. Magnified insert of the ^{1}H-^{13}C HSQC spectra of **TACC3$_{518\text{-}532\text{-}L/R\text{-}Bph}$** at the Cα region showing ^{13}C signal assignments.

NMR analysis: Control TACC3 $_{518\text{-}532}$ Ser524Cys/Glu530Cys Bph constrained variant

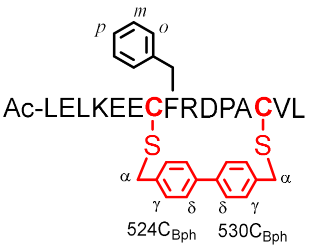

TACC3 $_{518\text{-}532\text{-SE-Bph}}$ Residue[a]	$\delta_{NH,obs}$[b]	$\delta_{H\alpha,obs}$[b]	$\delta_{H\beta,obs}$[b]	$\delta_{H\gamma,obs}$[b]	$\delta_{H\delta,obs}$[b]	$\delta_{H\varepsilon,obs}$[b]	others
L518 (overlaid)	8.361	4.22	1.571	1.571	0.897/0.84		
E519	8.638	4.243	1.977/1.931 (*a*,*b*)	2.228/2.211(*a*,*b*)			
L520 (overlaid)	8.361	4.22	1.571 (*a*,*b*)	1.571	0.897/0.84		
K521	8.383	4.323	1.816/1.764	1.418	1.576	2.955	NH_{ε}= 7.621
E522	8.597	4.213	2.002/1.946 (*a*,*b*)	2.256			
E523	8.697	4.196	1.90 (*a*,*b*)	2.20/2.136 (*a*,*b*)			
C524	8.369	4.334	2.729/2.537(*a*,*b*)				Bph_{α}= 3.789<br>Bph_{γ}= 7.420<br>Bph_{δ}= 7.638
F525	8.556	4.608	3.07/2.920				Ar_{p}= 7.244<br>Ar_{m}= 7.305,7.251,<br>Ar_{o}= 7.205, 7.171
R526	7.987	3.688	1.152 (*a*,*b*)	0.904	2.750		NH_{ε}= 7.05
D527	7.4913	4.587	2.761/2.595 (*a*,*b*)				
P528		4.223	2.247	1.952	3.855		
A529	8.445	4.256	1.358				
C530	7.874	4.488	2.805 (*a*,*b*)				Bph_{α}= 3.681<br>Bph_{γ}= 7.464<br>Bph_{δ}= 7.699
V531	8.348	4.083	2.086	0.893/0.804			
L532	8.434	4.256	1.614/1.554 (*a*,*b*)	1.614	0.803		

Table S14. ^{1}H-NMR assignments in TACC3$_{518\text{-}532\text{-S/E-Bph}}$. [a]One letter code for amino acids. [b]Experimental chemical shifts (ppm) as observed for the peptide in buffer/D_2O 90/10 vol/vol at 5 °C. Buffer: 25 mM potassium phosphate, 50 mM NaCl, 5 mM $MgCl_2$, pH= 7.5.

TACC3 $_{518\text{-}532\text{-}S/E\text{-}Bph}$ Residue[a]	$\delta_{C\alpha,RC}$[b]	$\delta_{C\alpha}$, obs[c]	$\Delta\delta_{C\alpha}$[d]	$\delta_{H\alpha,RC}$ [b]	$\delta_{H\alpha}$, obs[c]	$\Delta\delta_{H\alpha}$[d]	$\delta_{HN,RC}$ [b]	δ_{HN}, obs[c]	$\Delta\delta_{HN}$[d]
L518	55.43	55.36	-0.07	4.328	4.22	-0.108	8.469	8.361	-0.108
E519	56.462	56.36	-0.102	4.276	4.243	-0.033	8.588	8.638	0.05
L520	55.119	55.36	0.241	4.351	4.22	-0.131	8.371	8.361	-0.01
K521	56.372	55.63	-0.742	4.313	4.323	0.01	8.453	8.383	-0.07
E522	56.64	55.53	-1.11	4.278	4.213	-0.065	8.524	8.597	0.073
E523	56.978	56.71	-0.268	4.226	4.196	-0.03	8.687	8.697	0.01
**C524***	58.592	55.15	-3.442	4.425	4.334	-0.091	8.534	8.369	-0.165
F525	57.759	56.92	-0.839	4.684	4.608	-0.076	8.444	8.556	0.112
R526	55.879	57.13	1.251	4.317	3.688	-0.629	8.281	7.987	-0.294
D527	52.376	51.83	-0.546	4.834	4.587	-0.247	8.457	7.4913	-0.9657
P528	63.197	64.25	1.053	4.425	4.223	-0.202	***	***	***
A529	52.7	52.59	-0.11	4.283	4.256	-0.027	8.553	8.445	-0.108
**C530***	58.381	56.88	-1.501	4.518	4.488	-0.03	8.464	7.874	-0.59
V531	62.471	62.7	0.229	4.112	4.083	-0.029	8.416	8.348	-0.068
L532	55.191	55.23	0.039	4.364	4.256	-0.108	8.477	8.434	-0.043

Table S15. Secondary chemical shifts in control TACC3$_{518\text{-}532\text{-}S/E\text{-}Bph}$. [a] One letter code for amino acids. [b] Theoretical random coil $\delta_{C\alpha,RC}$, $\delta_{H\alpha,RC}$ and $\delta_{NH,RC}$ chemical shifts according to https://www1.bio.ku.dk/english/research/bms/sbinlab/randomchemicalshifts2/,[6–8] as calculated for TACC3 $_{518\text{-}532}$ at 5 °C and pH= 7.5. [c] Observed experimental Cα, Hα and amide HN chemical shifts (ppm). [d]Calculated secondary (δ_{RC} - δ_{obs}) $\Delta\delta_{C\alpha}$, $\Delta\delta_{H\alpha}$ and $\Delta\delta_{NH}$ chemical shifts (ppm). Note that in the absence of an appropriate reference for the constrained modified aminoacids, these have been calculated referenced to the chemical shift of a Cys residue. Values for these residues might be misrepresented and might be considered only orientative.

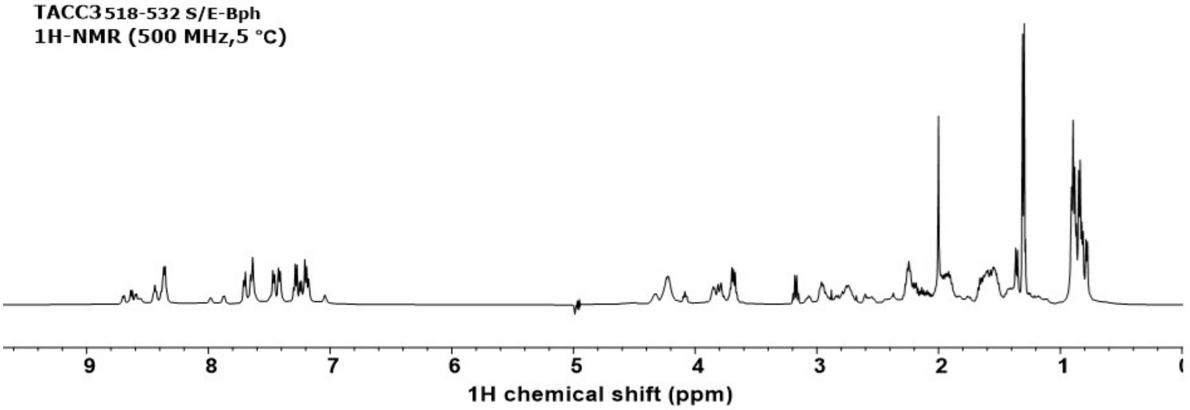

Figure S72. ^{1}H-NMR (500 MHz) trace of TACC3$_{518\text{-}532\text{-}S/E\text{-}Bph}$ in buffer/D_2O 90/10 vol/vol at 5 °C. Buffer: 25 mM potassium phosphate, 50 mM NaCl, 5 mM $MgCl_2$, pH= 7.5.

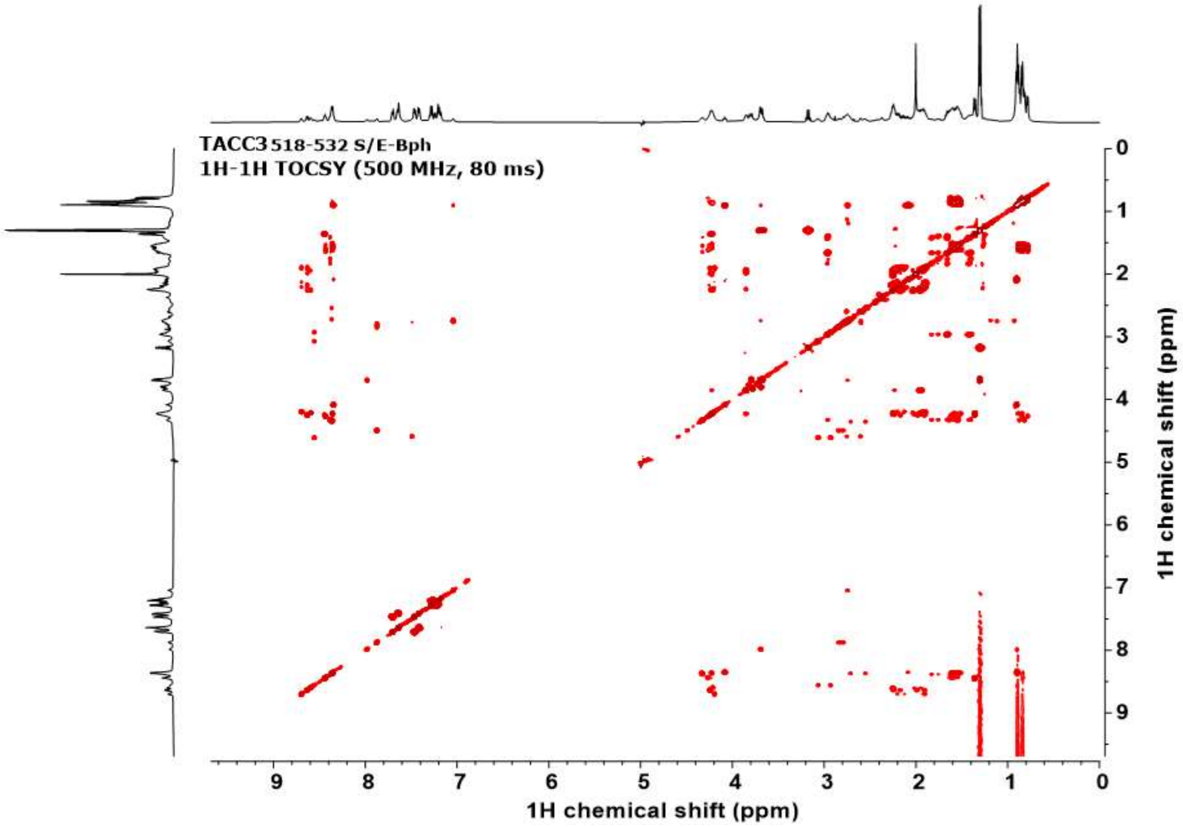

Figure S73. ^{1}H-^{1}H TOCSY NMR (500 MHz) trace of TACC3$_{518\text{-}532\text{-}S/E\text{-}Bph}$ in buffer/D_2O 90/10 vol/vol at 5 °C. Buffer: 25 mM potassium phosphate, 50 mM NaCl, 5 mM $MgCl_2$, pH= 7.5.

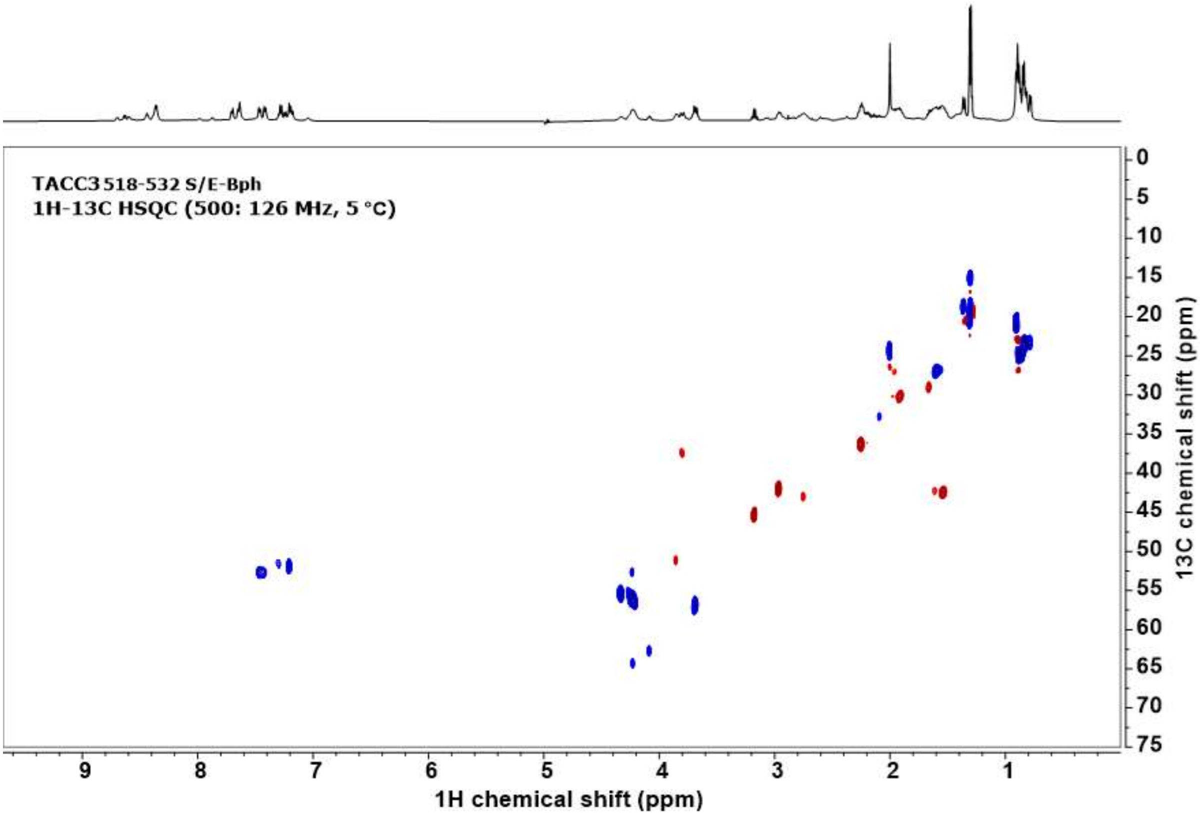

Figure S74. ¹H-¹³C HSQC NMR (500:126 MHz) trace of TACC3$_{518\text{-}532\text{-}S/E\text{-}Bph}$ in buffer/D_2O 90/10 vol/vol at 5 °C. Buffer: 25 mM potassium phosphate, 50 mM NaCl, 5 mM $MgCl_2$, pH= 7.5.

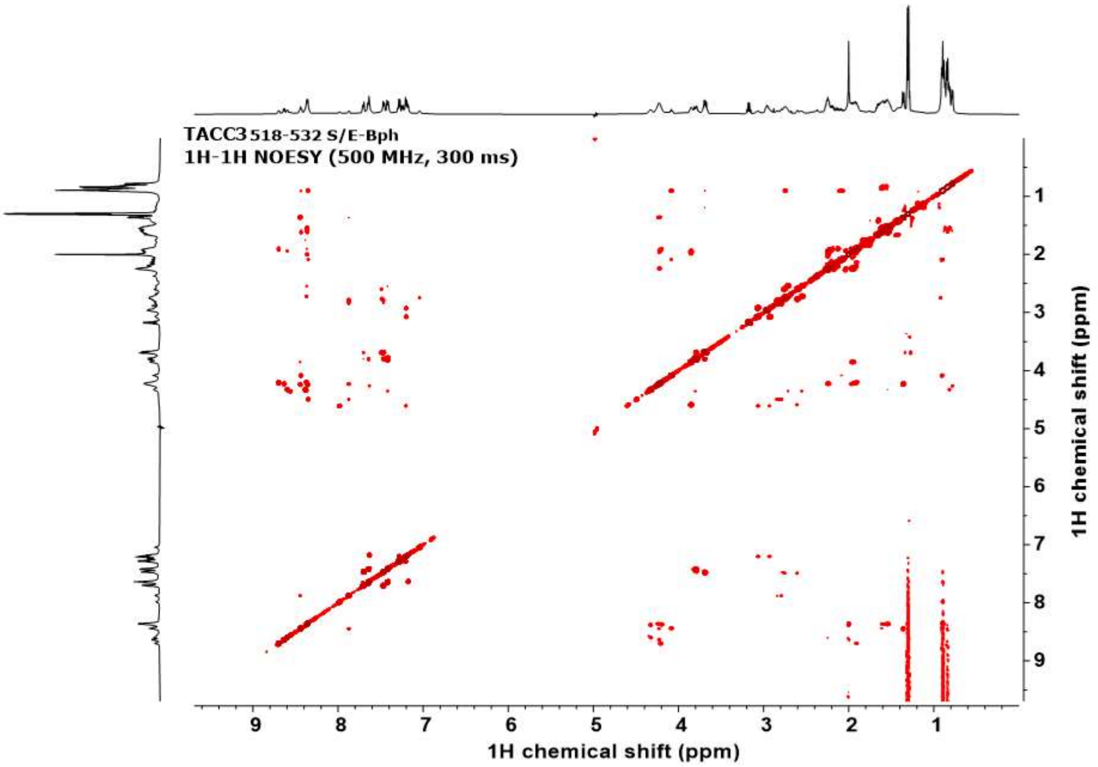

Figure S75. ¹H-¹H NOESY NMR (500 MHz) trace of TACC3$_{518\text{-}532\text{-}S/E\text{-}Bph}$ in buffer/D_2O 90/10 vol/vol at 5 °C. Buffer: 25 mM potassium phosphate, 50 mM NaCl, 5 mM $MgCl_2$, pH= 7.5.

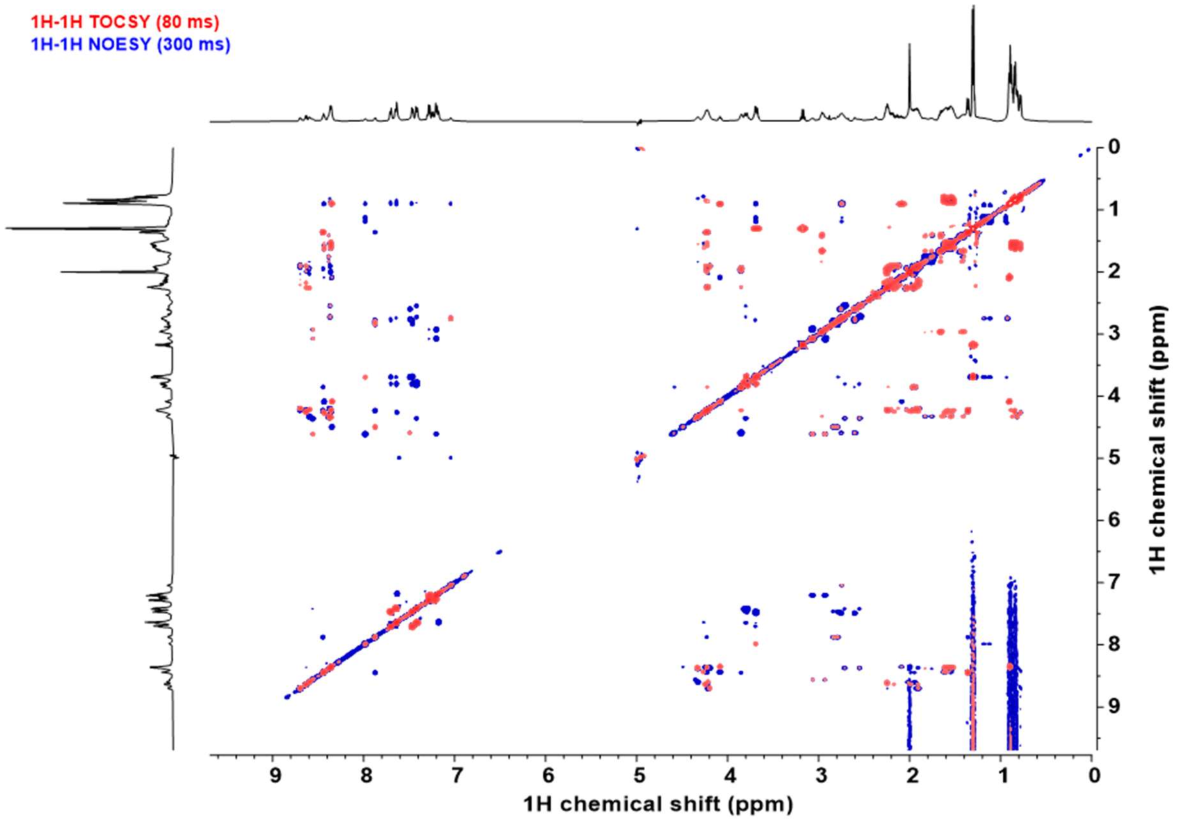

Figure S76. Overlaid ^{1}H-^{1}H TOCSY (in red) and ^{1}H-^{1}H NOESY spectra (in blue) of TACC3$_{518\text{-}532\text{-}S/E\text{-}Bph}$ in buffer/D_2O 90/10 vol/vol at 5 °C. Buffer: 25 mM potassium phosphate, 50 mM NaCl, 5 mM $MgCl_2$, pH= 7.5.

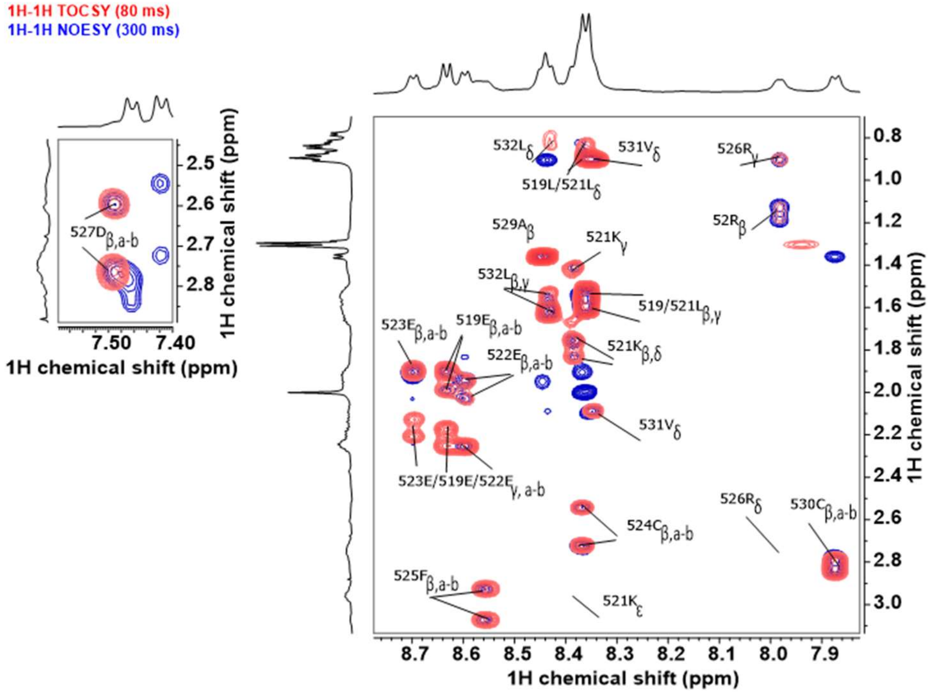

Figure S77. Magnified ^{1}H-^{1}H TOCSY spectra (red) and ^{1}H-^{1}H NOESY spectra (blue) of **TACC3$_{518\text{-}532\text{-}S/E\text{-}Bph}$** at the amide NH-side chain region showing sequence signal assignments.

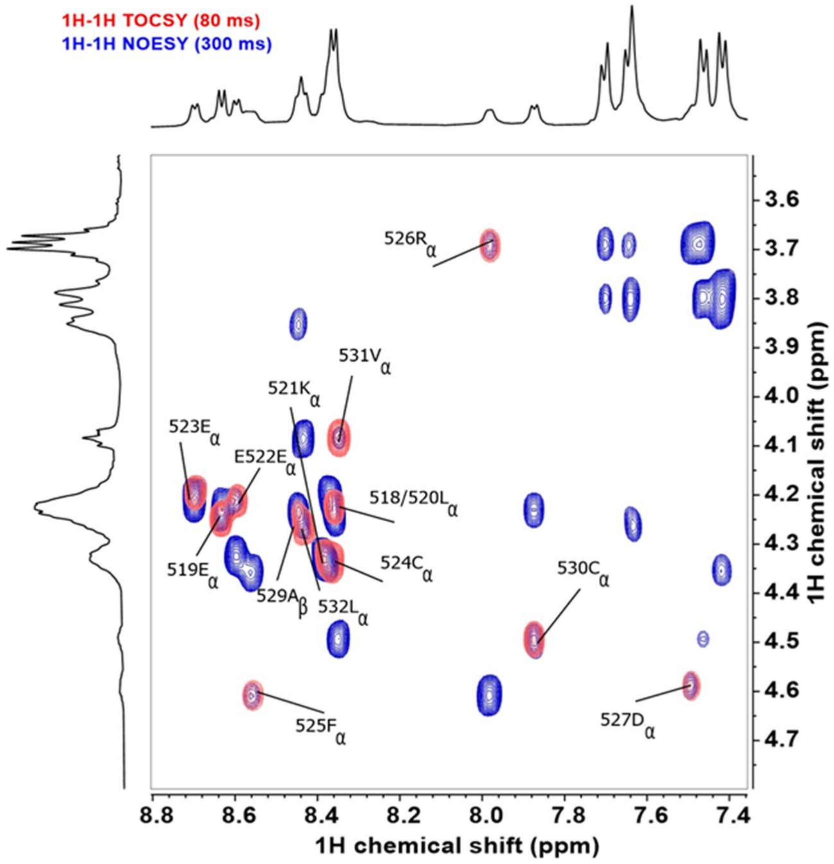

Figure S78. Magnified ^{1}H-^{1}H TOCSY spectra (red) and ^{1}H-^{1}H NOESY spectra (blue) of **TACC3$_{518\text{-}532\text{-}S/E\text{-}Bph}$** at the amide NH-Hα region showing ^{1}H signal assignments.

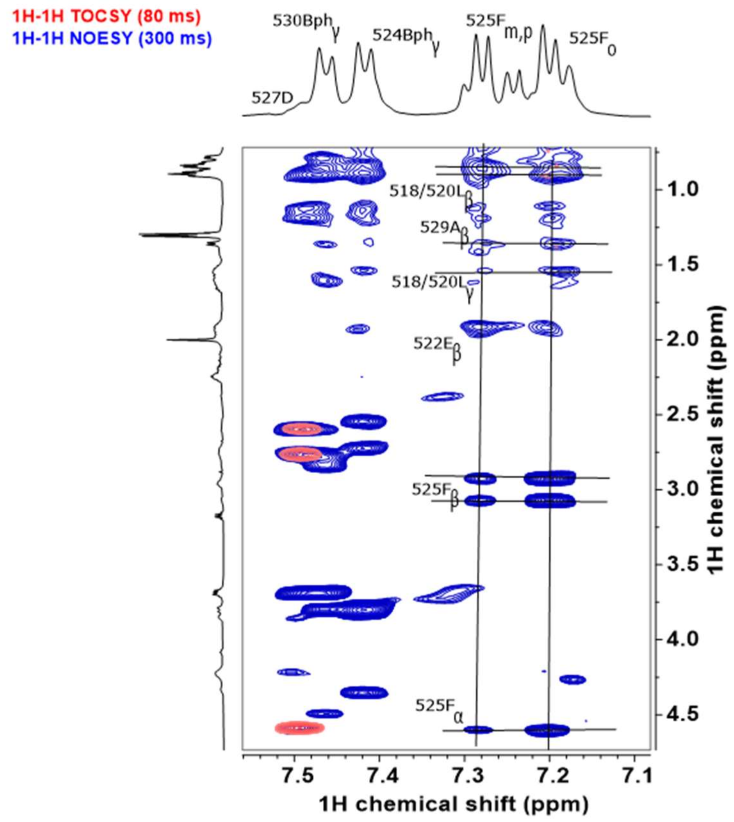

Figure S79. Magnified ^1H-^1H TOCSY spectra (red) and ^1H-^1H NOESY spectra (blue) of **TACC3$_{518\text{-}532\text{-}S/E\text{-}Bph}$** at the Phe525 aromatic region.

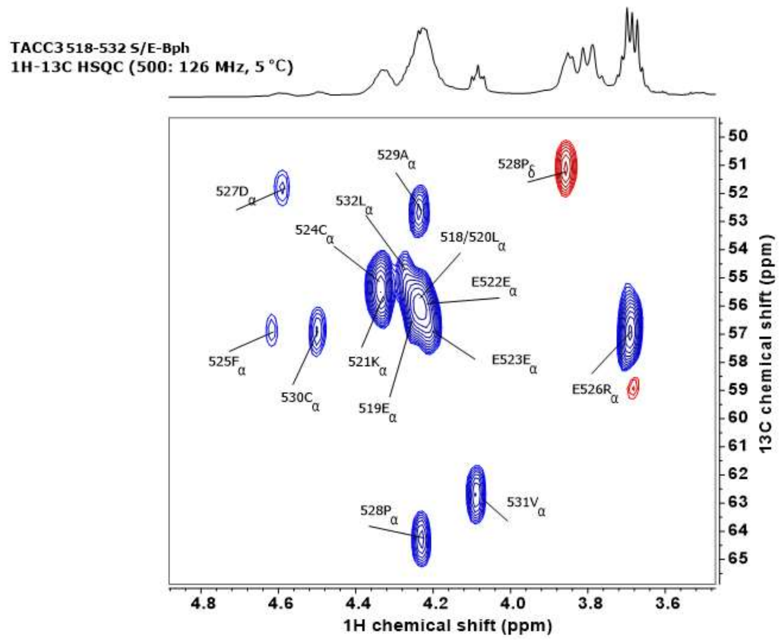

Figure S80. Magnified insert of the ^1H-^{13}C HSQC spectra of **TACC3$_{518\text{-}532\text{-}S/E\text{-}Bph}$** at the Cα region showing ^{13}C signal assignments.

NMR analysis: TACC3 $_{518\text{-}532}$ Ser524Cys/Glu530Cys Bph constrained (4-I)Phe525/*trans*-(4-F)Pro528 variant

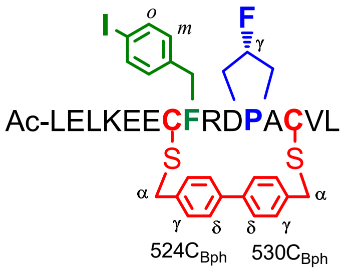

TACC3 $_{518\text{-}532\text{-}SE\text{-}Bph}$ Residue[a]	$\delta_{NH,obs}$[b]	$\delta_{H\alpha,obs}$[b]	$\delta_{H\beta,obs}$[b]	$\delta_{H\gamma,obs}$[b]	$\delta_{H\delta,obs}$[b]	$\delta_{H\varepsilon,obs}$[b]	others
L518	8.363	4.225	1.577 (*a*,*b*)	1.577	0.903/0.812		
E519	8.639	4.249	1.975/1.905 (*a*,*b*)	2.241/2.188(*a*,*b*)			
L520	8.355	4.337	1.577 (*a*,*b*)	1.577	0.903/0.812		
K521	8.39	4.325	1.789	1.413	1.678	2.661	NH_ε= 7.614
E522	8.602	4.203	2.016/1.946 (*a*,*b*)	2.255			
E523	8.692	4.225	1.939 (*a*,*b*)	2.231 (*a*,*b*)			
C524	8.4	4.392	2.720/2.516 (*a*,*b*)				Bph_α= 3.792<br>Bph_γ= 7.410<br>Bph_δ= 7.634
F525	8.601	4.577	2.949				Ar_m= 6.963<br>Ar_o= 7.631
R526	7.921	3.669	1.096/1.130 (*a*,*b*)	0.787	2.74		NH_ε= 7.018
D527	7.661	4.55	2.788/2.598 (*a*,*b*)				
P528		4.502 (A)<br>4.448 (B)	2.785/2.189 (A) (*a*,*b*)<br>2.764/2.109 (B) (*a*,*b*)	5.476 (A)<br>5.375 (B)	4.083 (A)<br>4.007 (B)		
A529	8.478	4.189	1.348				
C530	7.895	4.505	2.841 (*a*,*b*)				Bph_α= 3.651<br>Bph_γ= 7.464<br>Bph_δ= 7.683
V531	8.35	4.093	2.091	0.896/0.812			
L532	8.443	4.263	1.602/1.544	1.602	0.795		

Table S16. ^{1}H-NMR assignments in TACC3$_{518\text{-}532\text{-}S/E\text{-}Bph\text{-}IF/fP}$. [a]One letter code for amino acids. [b]Experimental chemical shifts (ppm) as observed for the peptide in buffer/D_2O 90/10 vol/vol at 5 °C. Buffer: 25 mM potassium phosphate, 50 mM NaCl, 5 mM $MgCl_2$, pH= 7.5.

TACC3 $_{518\text{-}532\text{-}S/E\text{-}Bph\text{-}IF/fP}$ Residue[a]	$\delta_{C\alpha,RC}$[b]	$\delta_{C\alpha}$, obs[c]	$\Delta\delta_{C\alpha}$[d]	$\delta_{H\alpha,RC}$ [b]	$\delta_{H\alpha}$, obs[c]	$\Delta\delta_{H\alpha}$[d]	$\delta_{HN,RC}$ [b]	δ_{HN}, obs[c]	$\Delta\delta_{HN}$[d]
L518	55.43	55.98	0.55	4.328	4.225	-0.103	8.469	8.363	-0.106
E519	56.462	56.81	0.348	4.276	4.249	-0.027	8.588	8.639	0.051
L520	55.119	55.36	0.241	4.351	4.337	-0.014	8.371	8.355	-0.016
K521	56.372	54.69	-1.682	4.313	4.325	0.012	8.453	8.39	-0.063
E522	56.64	56.76	0.12	4.278	4.203	-0.075	8.524	8.602	0.078
E523	56.978	55.98	-0.998	4.226	4.225	-0.001	8.687	8.692	0.005
**C524***	58.592	55.31	-3.282	4.425	4.392	-0.033	8.534	8.4	-0.134
(4-I)F525	57.759	56.84	-0.919	4.684	4.577	-0.107	8.444	8.601	0.157
R526	55.879	54.91	-0.969	4.317	3.669	-0.648	8.281	7.921	-0.36
D527	52.376	51.73	-0.646	4.834	4.55	-0.284	8.457	7.661	-0.796
trans-(4-F)P528	63.197	62.95	-0.247	4.425	4.502	0.077	***	0.3	#VALUE!
A529	52.7	52.7	0	4.283	4.189	-0.094	8.553	8.478	-0.075
**C530***	58.381	57.28	-1.101	4.518	4.505	-0.013	8.464	7.895	-0.569
V531	62.471	62.73	0.259	4.112	4.093	-0.019	8.416	8.35	-0.066
L532	55.191	55.15	-0.041	4.364	4.263	-0.101	8.477	8.443	-0.034

Table S17. Secondary chemical shifts in TACC3$_{518\text{-}532\text{-}S/E\text{-}Bph\text{-}IF/fP}$. [a] One letter code for amino acids. [b] Theoretical random coil $\delta_{C\alpha,RC}$, $\delta_{H\alpha,RC}$ and $\delta_{NH,RC}$ chemical shifts according to https://www1.bio.ku.dk/english/research/bms/sbinlab/randomchemicalshifts2/,[6–8] as calculated for TACC3 $_{518\text{-}532}$ at 5 °C and pH= 7.5. [c] Observed experimental Cα, Hα and amide HN chemical shifts (ppm). [d]Calculated secondary (δ_{RC} - δ_{obs}) $\Delta\delta_{C\alpha}$, $\Delta\delta_{H\alpha}$ and $\Delta\delta_{NH}$ chemical shifts (ppm). Note that in the absence of an appropriate reference for the constrained modified aminoacids, these have been calculated referenced to the chemical shift of a Cys residue. Values for these residues might be misrepresented and might be considered only orientative.

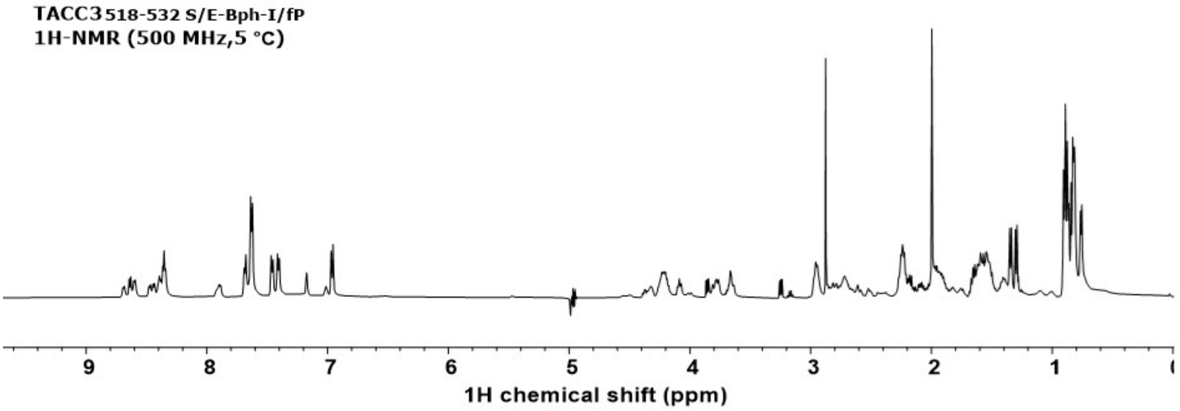

Figure S81. ^{1}H-NMR (500 MHz) trace of TACC3$_{518\text{-}532\text{-}S/E\text{-}Bph\text{-}I/fP}$ in buffer/D_2O 90/10 vol/vol at 5 °C. Buffer: 25 mM potassium phosphate, 50 mM NaCl, 5 mM $MgCl_2$, pH= 7.5.

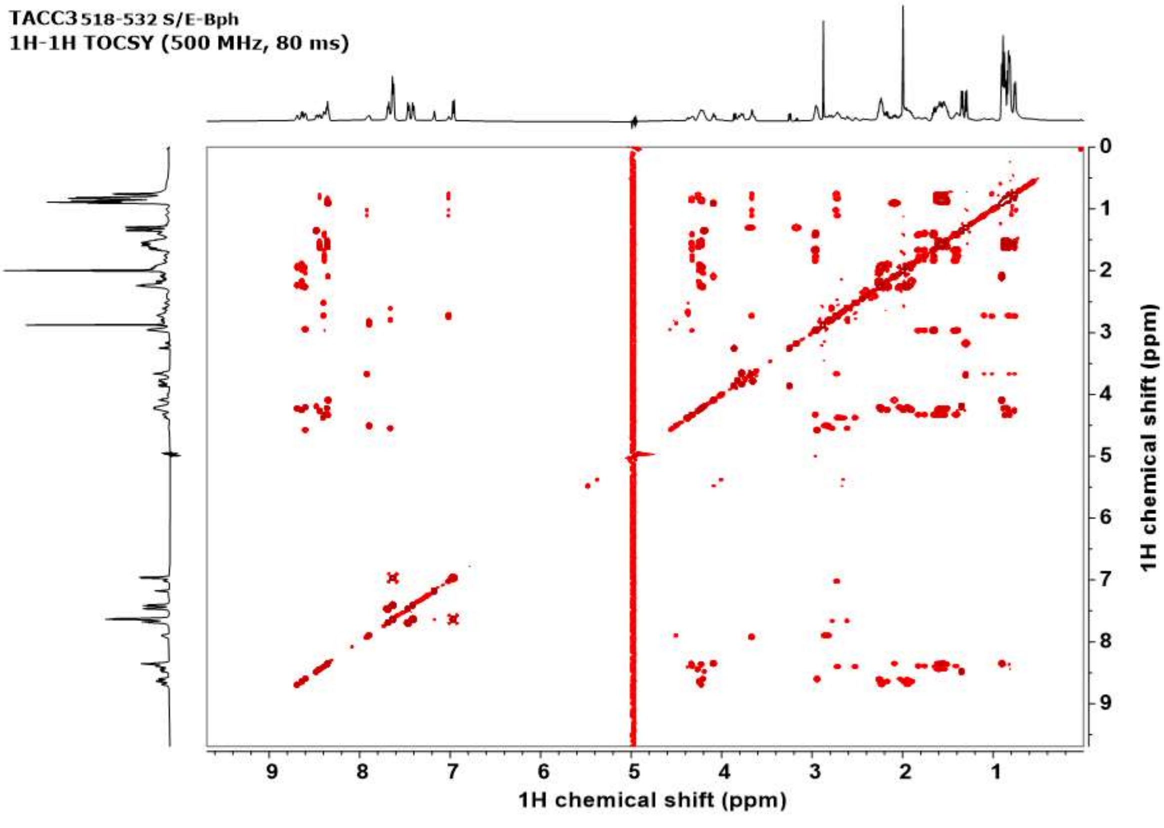

Figure S82. ^{1}H-^{1}H TOCSY NMR (500 MHz) trace of TACC3$_{518\text{-}532\text{-}S/E\text{-}Bph\text{-}I/fP}$ in buffer/D_2O 90/10 vol/vol at 5 °C. Buffer: 25 mM potassium phosphate, 50 mM NaCl, 5 mM $MgCl_2$, pH= 7.5.

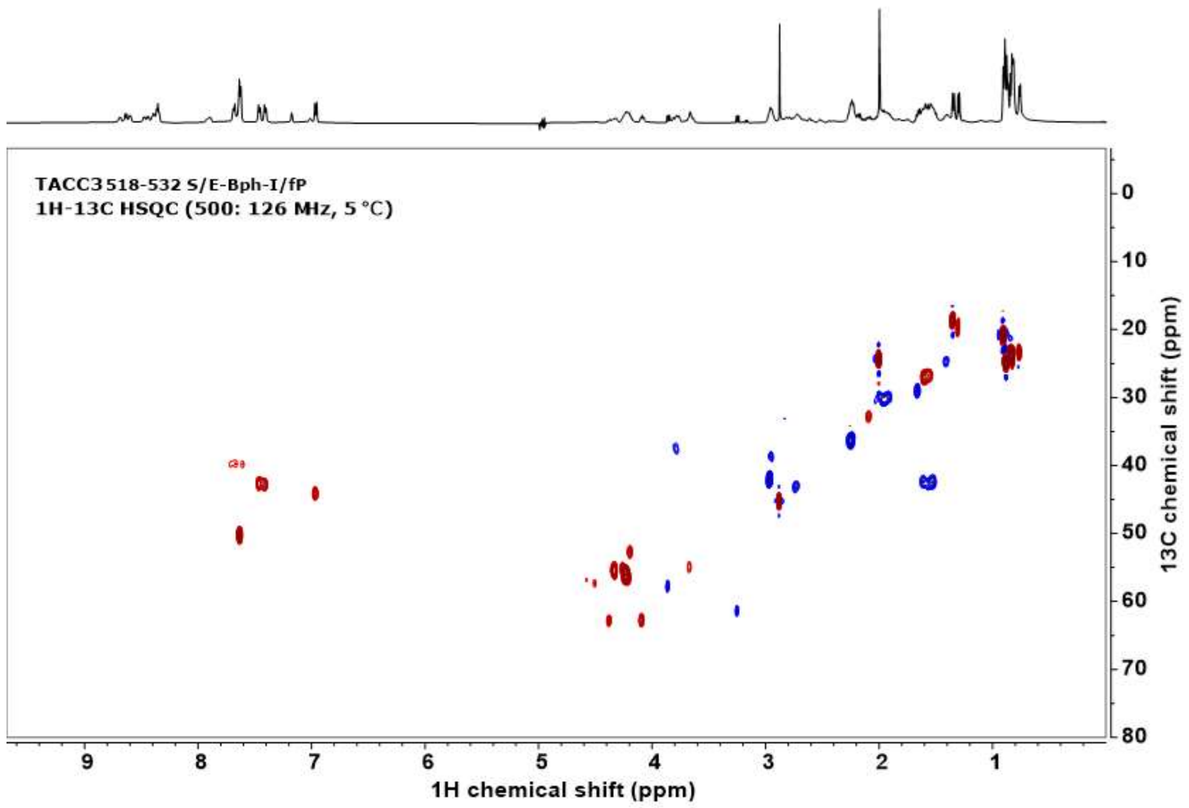

Figure S83. ^1H-^{13}C HSQC NMR (500:126 MHz) trace of TACC3 $_{518\text{-}532\text{-}S/E\text{-}Bph\text{-}I/fP}$ in buffer/D_2O 90/10 vol/vol at 5 °C. Buffer: 25 mM potassium phosphate, 50 mM NaCl, 5 mM $MgCl_2$, pH= 7.5.

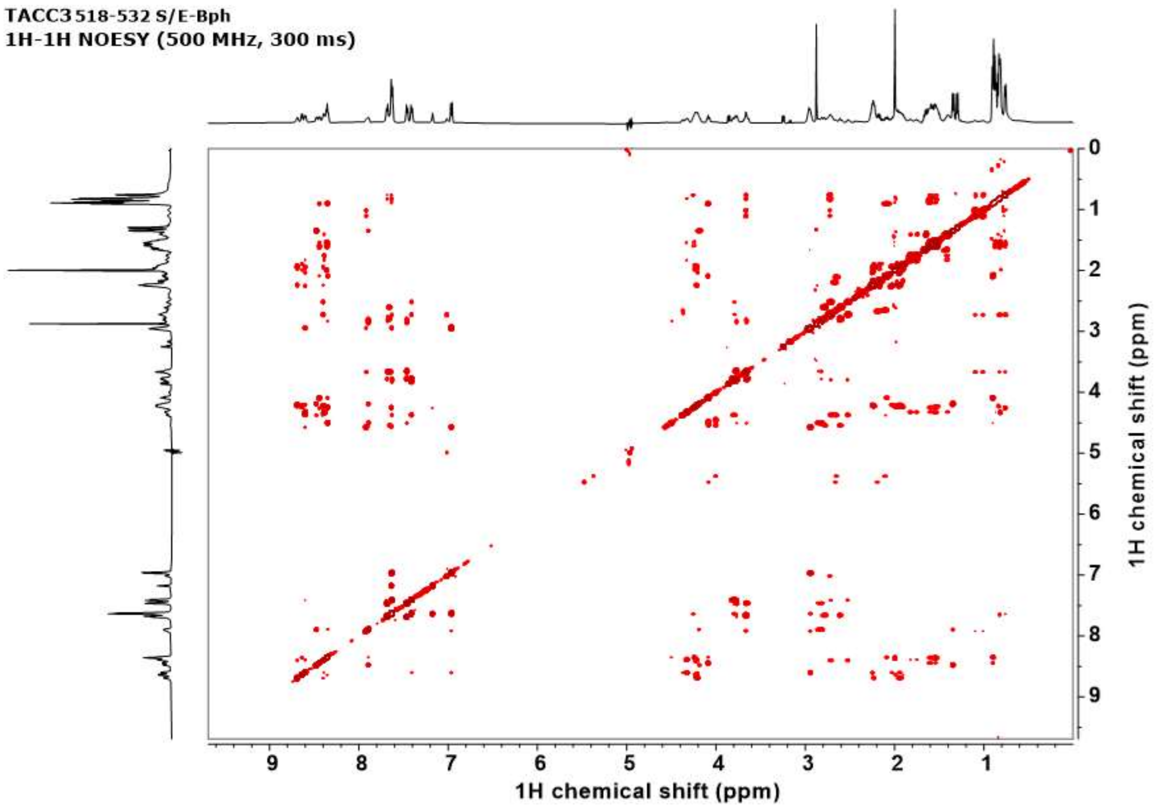

Figure S84. ^1H-^1H NOESY NMR (500 MHz) trace of TACC3$_{518\text{-}532\text{-}S/E\text{-}Bph\text{-}I/fP}$ in buffer/D_2O 90/10 vol/vol at 5 °C. Buffer: 25 mM potassium phosphate, 50 mM NaCl, 5 mM $MgCl_2$, pH= 7.5.

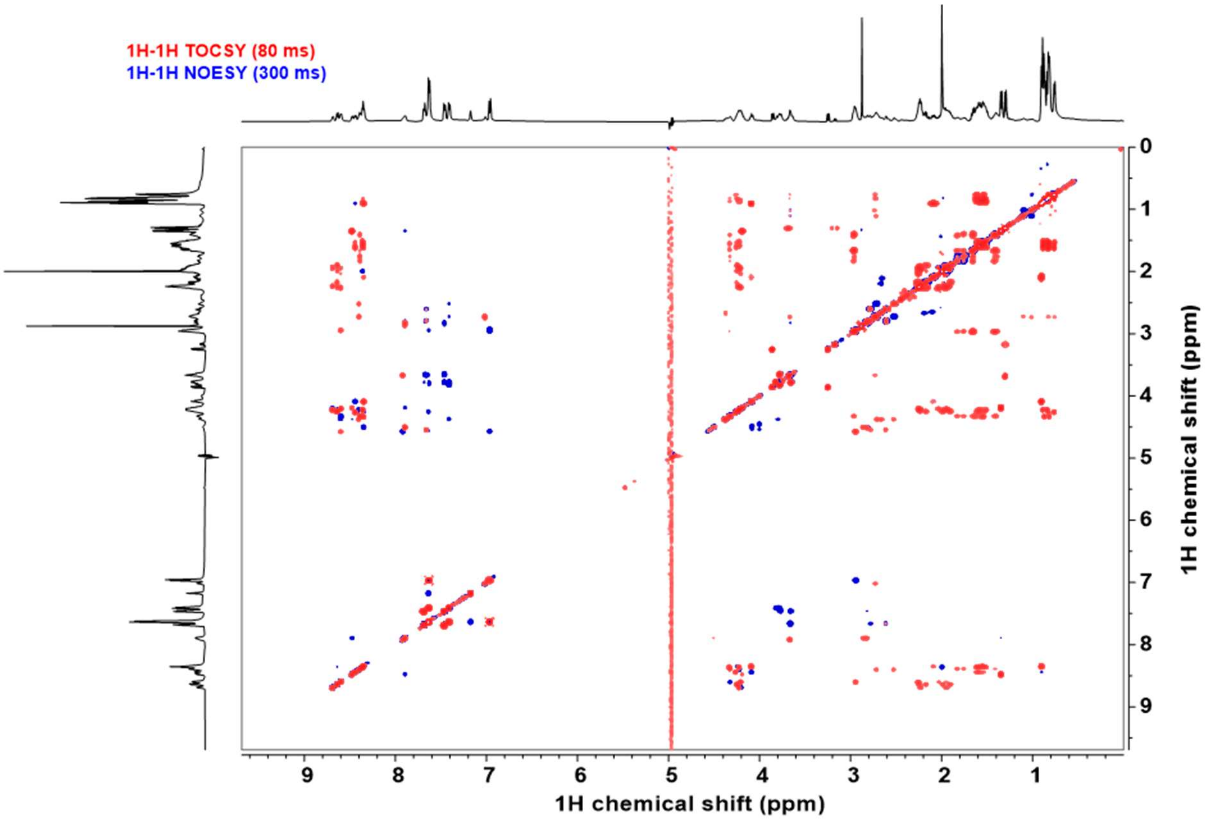

Figure S85. Overlaid ^1H-^1H TOCSY (in red) and ^1H-^1H NOESY spectra (in blue) of TACC3$_{518\text{-}532\text{-}S/E\text{-}Bph\text{-}I/fP}$ in buffer/D_2O 90/10 vol/vol at 5 °C. Buffer: 25 mM potassium phosphate, 50 mM NaCl, 5 mM $MgCl_2$, pH= 7.5.

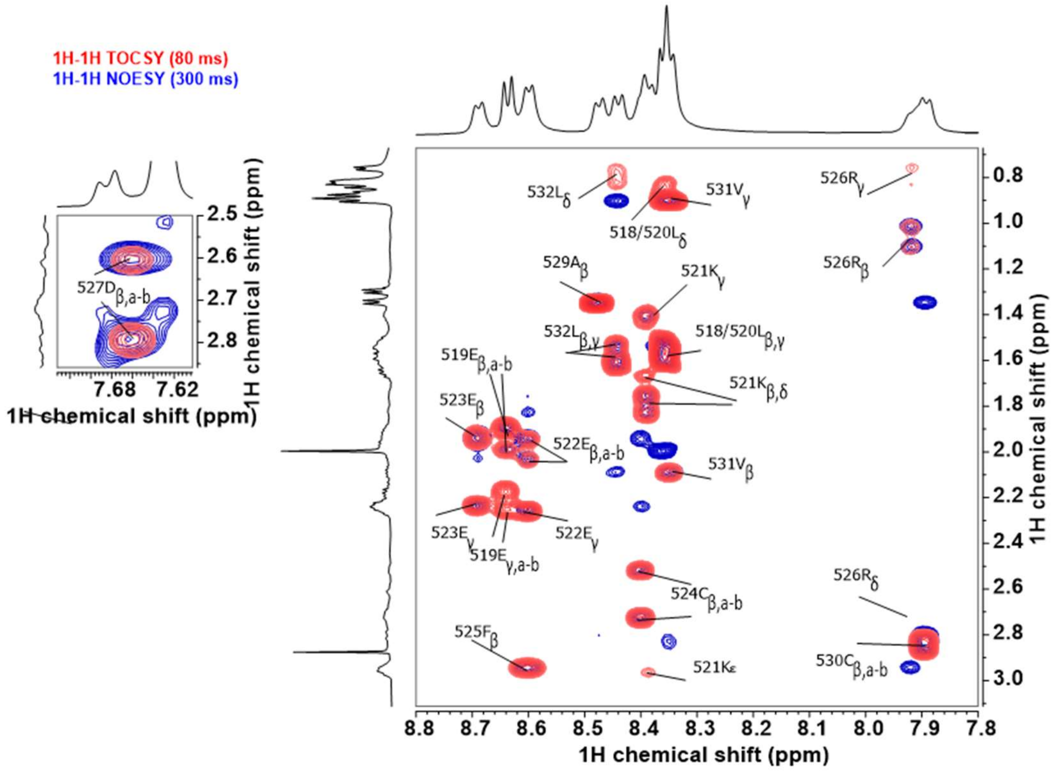

Figure S86. Magnified ^{1}H-^{1}H TOCSY spectra (red) and ^{1}H-^{1}H NOESY spectra (blue) of **TACC3$_{518\text{-}532\text{-}S/E\text{-}Bph\text{-}I/fP}$** at the amide NH-side chain region showing sequence signal assignments.

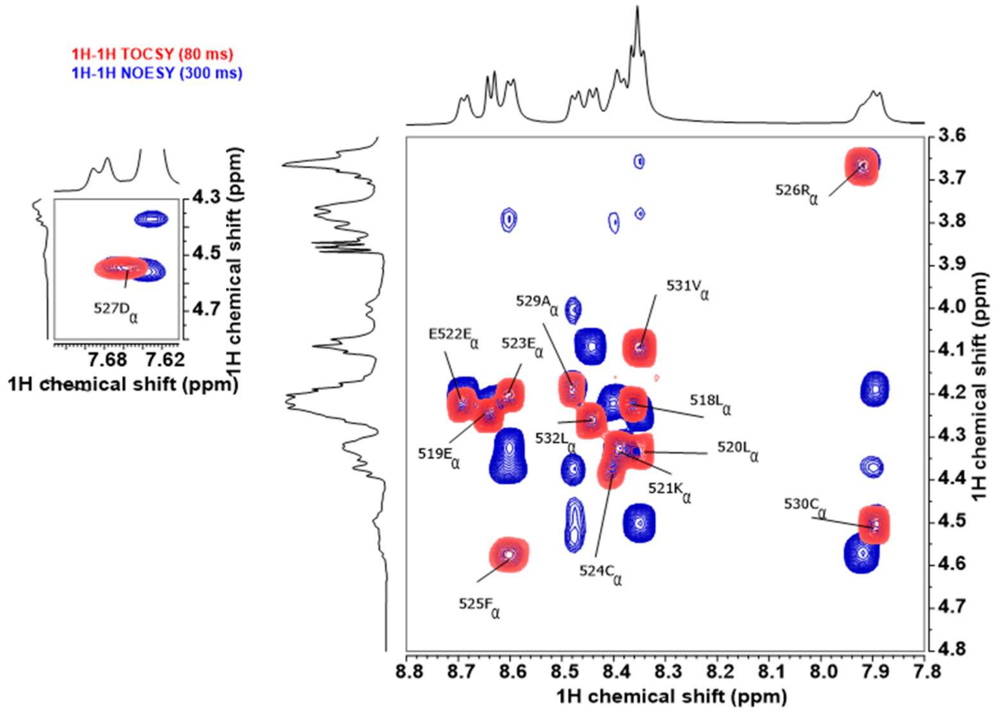

Figure S87. Magnified ^{1}H-^{1}H TOCSY spectra (red) and ^{1}H-^{1}H NOESY spectra (blue) of **TACC3$_{518\text{-}532\text{-}S/E\text{-}Bph\text{-}I/fP}$** at the amide NH-Hα region showing ^{1}H signal assignments.

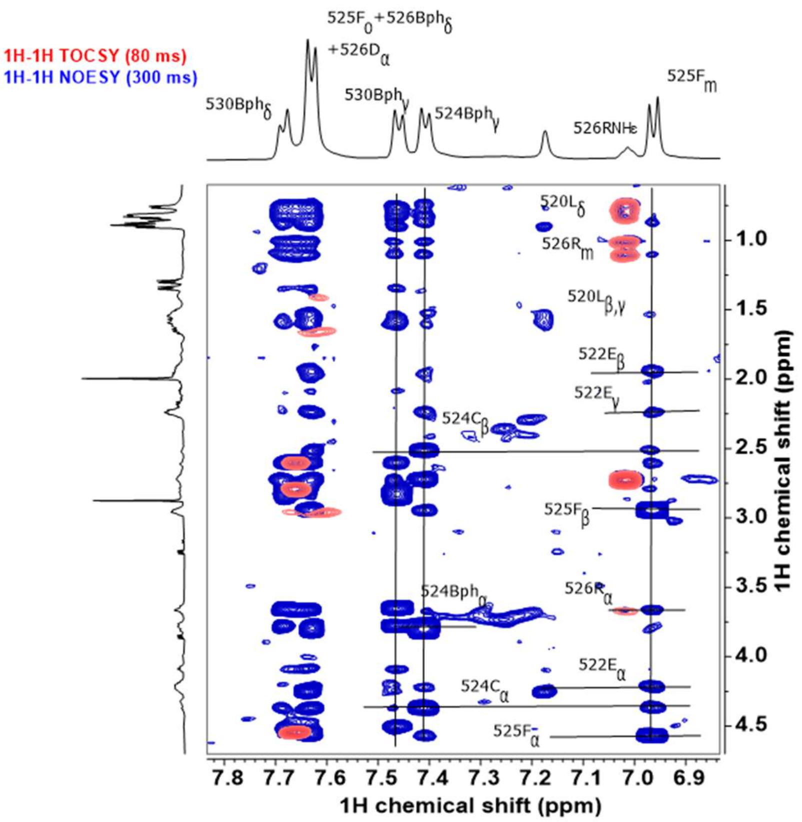

Figure S88. Magnified ^1H-^1H TOCSY spectra (red) and ^1H-^1H NOESY spectra (blue) of **TACC3$_{518\text{-}532\text{-}S/E\text{-}Bph\text{-}I/fP}$** at the ^{525}F aromatic region.

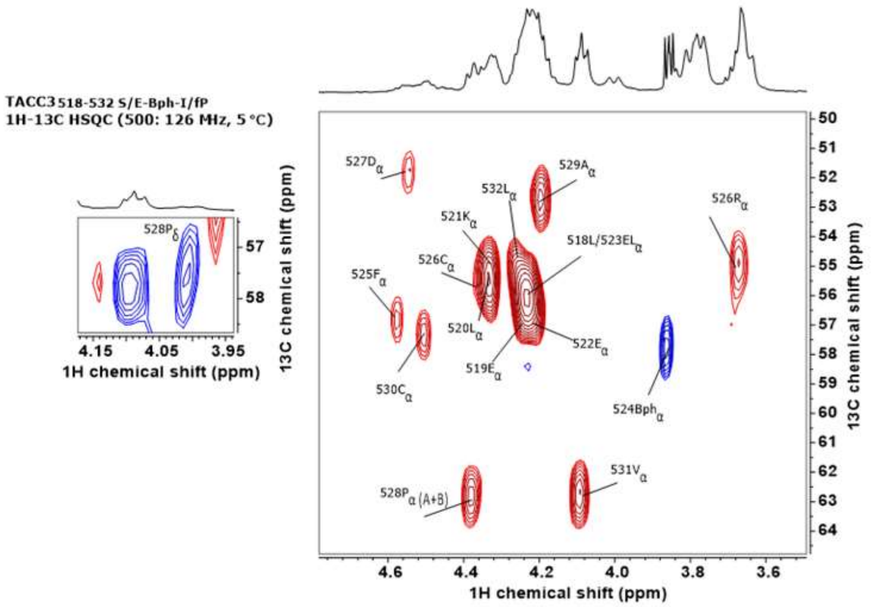

Figure S89. Magnified insert of the ^1H-^{13}C HSQC spectra of **TACC3$_{518\text{-}532\text{-}S/E\text{-}Bph\text{-}I/fP}$** at the Cα region showing ^{13}C signal assignments.

^{1}H (VT)-NMR of TACC3$_{518-532}$ Ser524Cys/Glu520Cys Bph- constrained (4-I)Phe525/*trans*-(4-F) Pro528 variant

Similar results to those explained for the alternative L/R constrained variants were measured upon analysis of TACC3 $_{518-532-S/E-Bph-IF/fP}$. In this case, it is worth noting that the Bph- grup did not presented signals as broadened as in the previous case, pointing out to a higher conformational mobility of the constraint when placed in these positions. Also, when compared to the L/R variant, we noticed a subtle change in the residues that were in apperance most affected by the rigidification imposed by the constraint, which were, in particular: Phe525, Asp527 and Arg526, all within the peptide region shielded by the constrain.

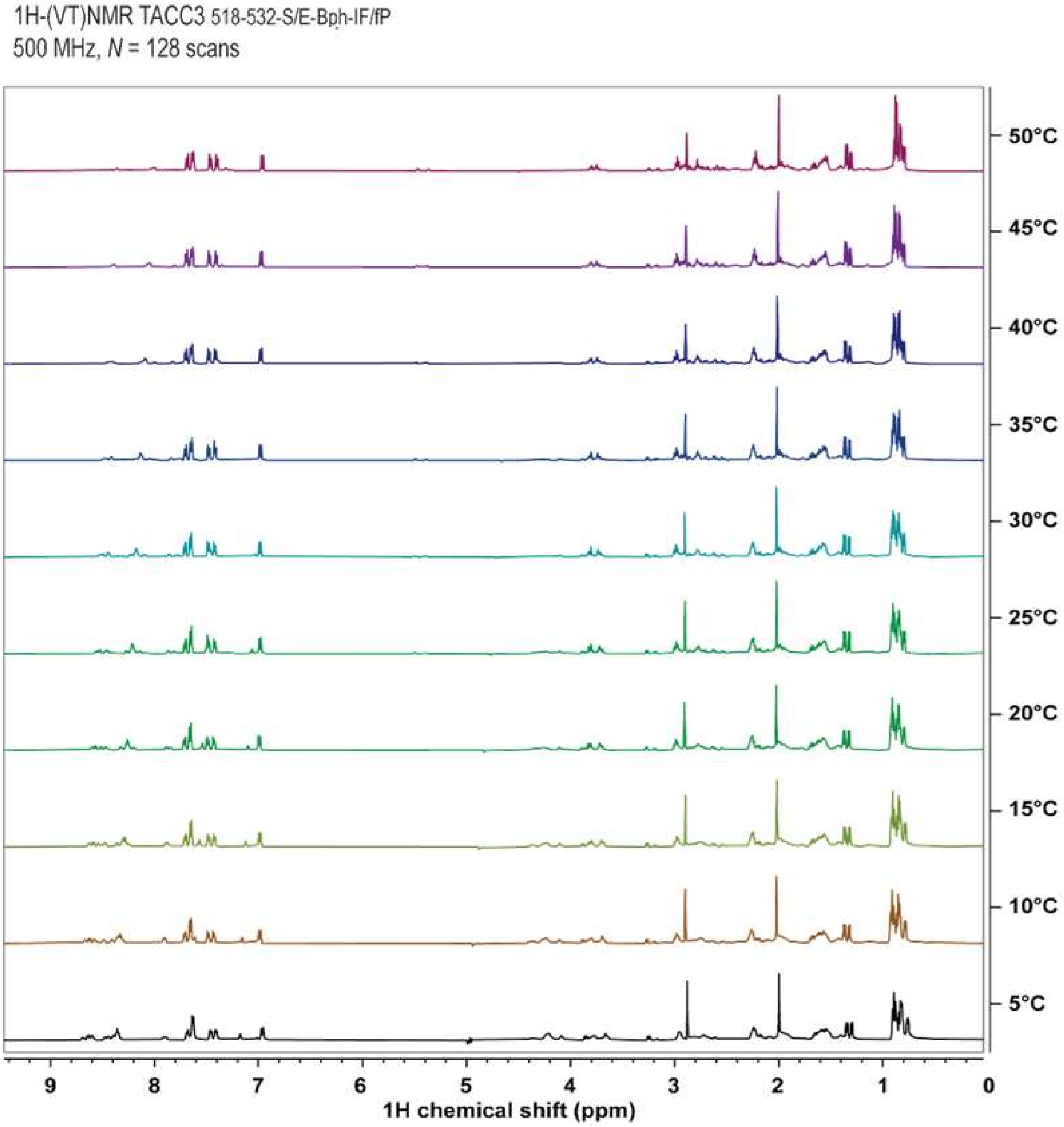

Figure S90. ^{1}H-VT NMR (500 MHz) traces observed for TACC3$_{518-532_SE_Bph_IF/fP}$ at increasing temperatures from 5 - 50 °C. Sample in 25 mM Tris, 150 mM NaCl, 5 mM $MgCl_2$, pH= 7.5/ D_2O 90/10 v/v.

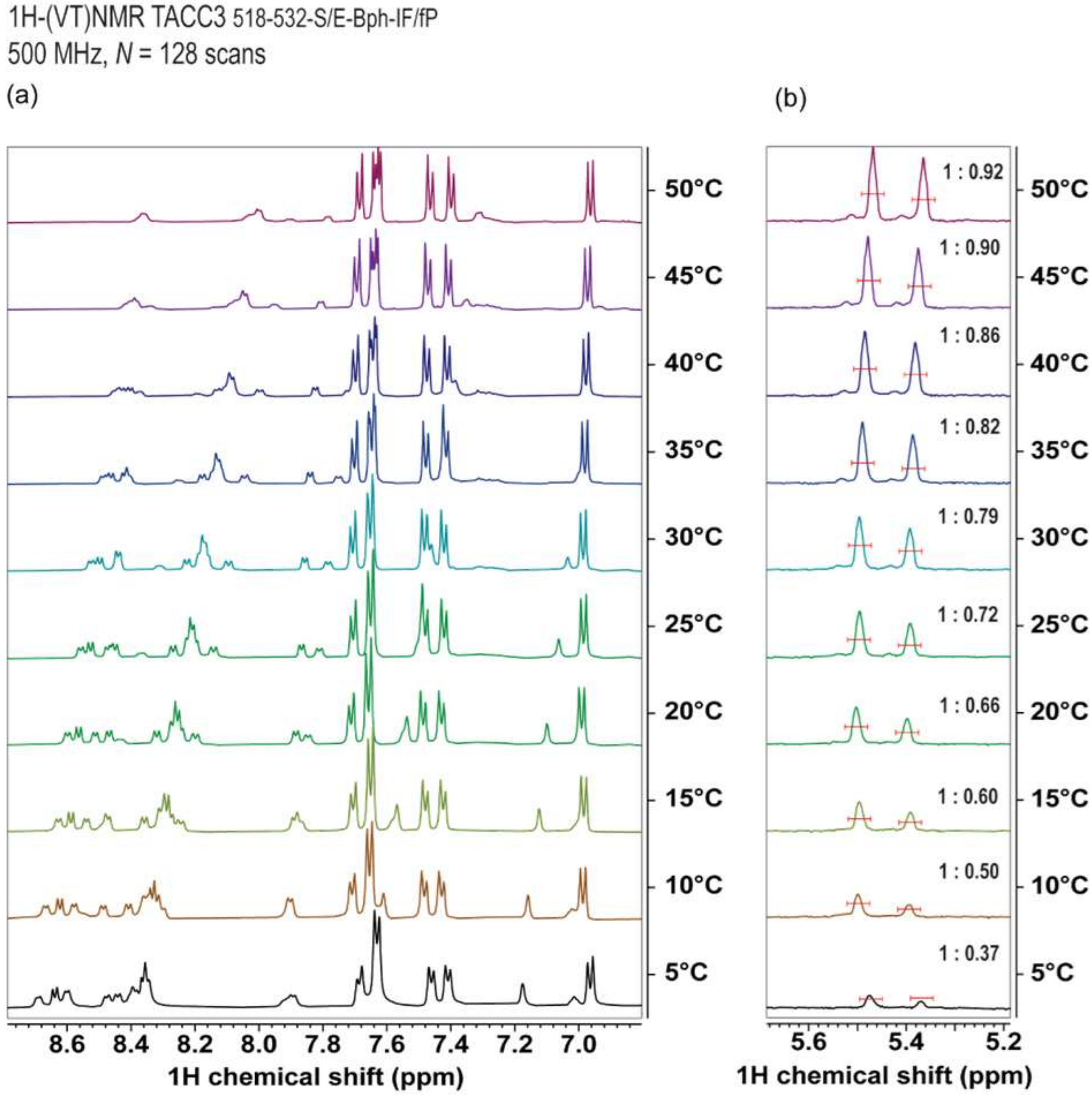

Figure S91. Insets of the ^{1}H-VT NMR (500 MHz) traces observed for **TACC3$_{518\text{-}532_SE_Bph_IF/fP}$** at the amide NH region (a) and (b) (4-F)P528 Hγ proton region. Sample in 25 mM Tris, 150 mM NaCl, 5 mM $MgCl_2$, pH= 7.5/ D_2O 90/10 v/v.

Isothermal titration calorimetry (ITC)

Peptide[a]	Sequence		*N restrained fit* K_d [b] (μM)	ΔG°[c] (kJ mol^{-1})	ΔH°[c] (kJ mol^{-1})	-TΔS°[c] (kJ mol^{-1})	*N*	*N unrestrained fit* K_d [b] (μM)	ΔG°[c] (kJ mol^{-1})	ΔH°[c] (kJ mol^{-1})	-TΔS°[c] (kJ mol^{-1})	*N*
TACC3 $_{522\text{-}536}$	Ac-EESFRDPAEVLGTGA-NH_2		57.4	-24.2	-21.5	-2.6	1.0	58.6	-24.1	-22.2	-1.93	0.97
TACC3 $_{518\text{-}532}$	Ac-LELKEESFRDPAEVL-NH_2	Run 1	30.3	-25.8	-11.5	-14.3	1.0	46.0	-25.6	-10.60	-13.0	0.911
		Run 2	34.5	-25.5	-9.7	-15.7	1.0	36.8	-25.3	-10.5	-14.8	0.931
TACC3 $_{518\text{-}532\text{-}L/R\text{-}Bph\text{-}lF/fP}$	Ac-LELCKEES^{l}FCD*f*PAEVL-NH_2 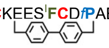	Run 1	5.6	-30.0	-6.9	-23.0	1.0	4.6	-30.5	-6.48	-23.9	1.06
		Run 2	5.9	-29.9	-7.5	-22.3	1.0	5.5	-30.0	-7.3	-22.7	1.02
TACC3 $_{518\text{-}532\text{-}S/E\text{-}Bph\text{-}lF/fP}$	Ac-LELKEEC^{l}FRD*f*PACVL-NH_2 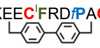							16.7	-27.3	-8.6	-18.7	1.62

Table S18. ITC analysis and thermodynamic parameters of selected linear and constrained peptides binding to Aurora-$A_{122\text{-}403\text{-}C290A/C393A}$. [a] One letter code for amino acids. [b] K_d values and [c] main ΔG°, ΔH°, and -TΔS° given as determined from direct isothermal titration assays of Aurora-$A_{122\text{-}403\text{-}C290A/C393A}$ (25 °C; 46 μM) using the corresponding acetyl-capped peptides (1.25 mM). All assays were performed in 25 mM Tris, 150 mM NaCl, 5 mM $MgCl_2$, 5% v/v glycerol; pH= 7.5.

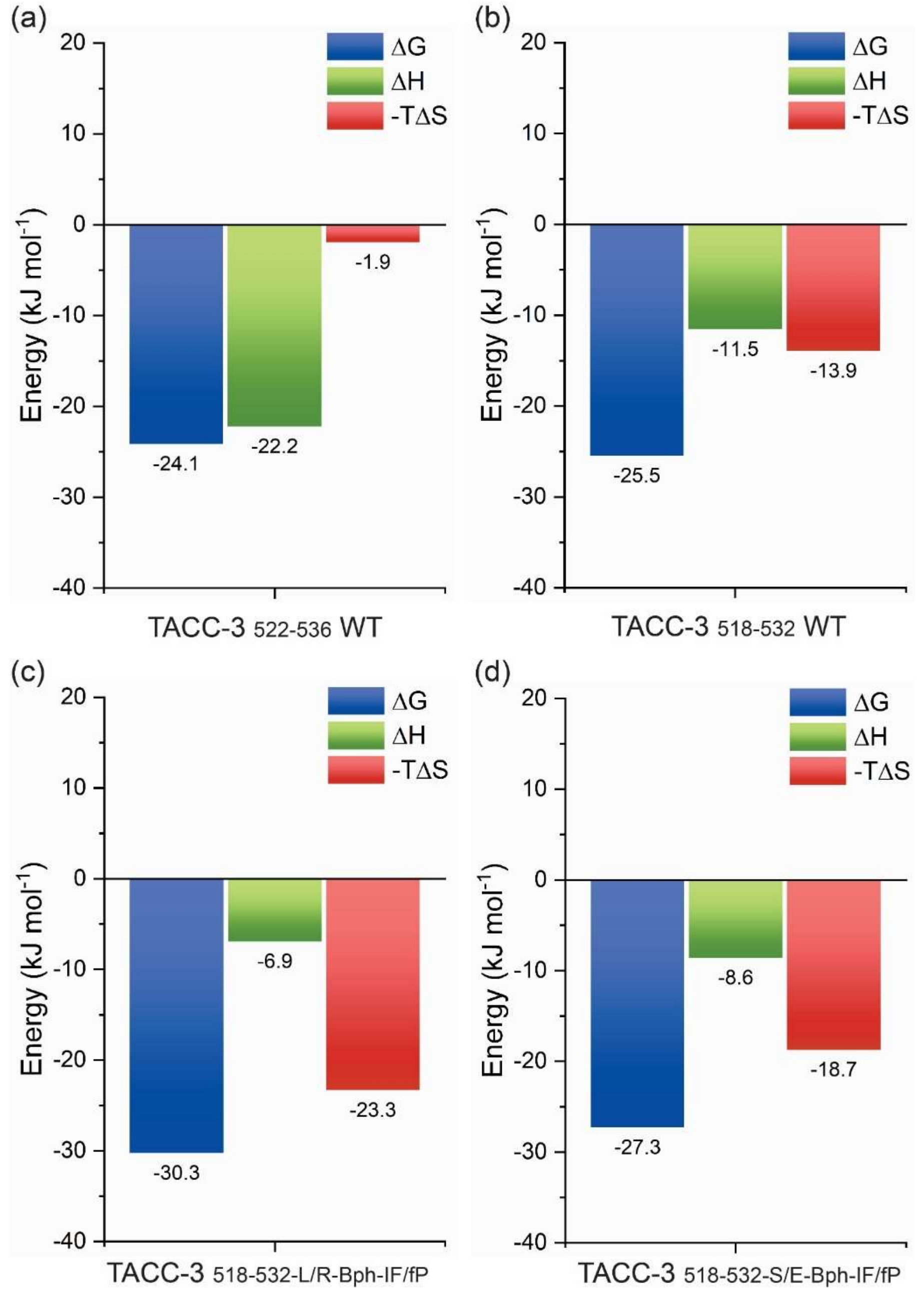

Figure S92. Thermodynamic ITC signatures of linear (a) $TACC3_{522\text{-}536}$ and (b) TACC3 $_{518\text{-}532}$ and Bph-constrained lead peptides $TACC3_{518\text{-}532\text{-}L/R\text{-}Bph\text{-}IF/fP.}$ (c) and $TACC3_{518\text{-}532\text{-}S/E\text{-}Bph\text{-}IF/fP.}$ All samples were measured in 25 mM Tris, 150 mM NaCl, 5 mM $MgCl_2$, 5% v/v glycerol; pH= 7.5, average values shown where applicable).

TACC3 $_{522\text{-}536}$ WT:

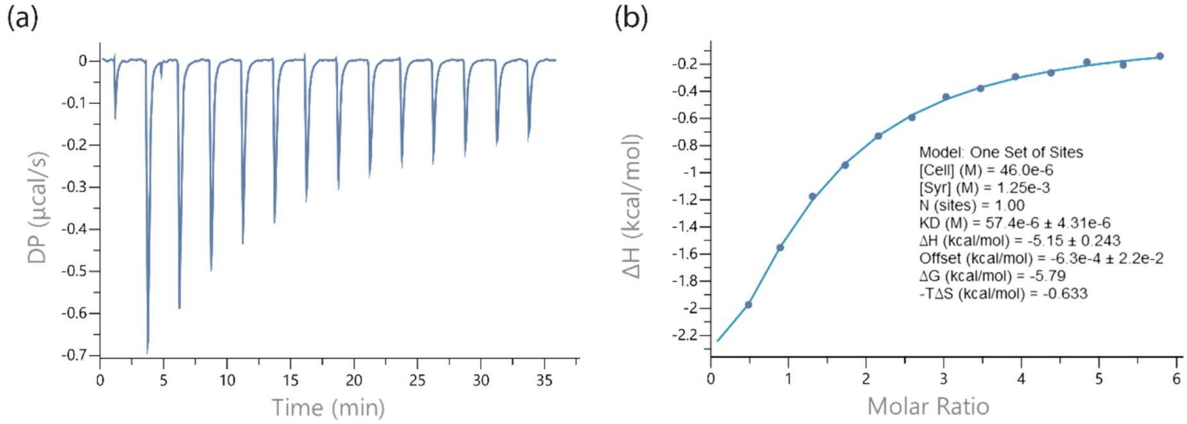

Figure S93. Isothermal titration calorimetry of TACC3$_{522\text{-}536}$ and Aurora-A$_{122\text{-}403\text{-}C290A/C393A}$. (a) Raw heat and (b) integrated, baseline-corrected heats per injection and corresponding fit on the right panel. Listed with each titration are the concentrations of the protein in the syringe and in the cell, as well as the parameters of the fit (stoichiometry *N*, dissociation constant K_d).

TACC3 $_{518\text{-}532}$ WT:

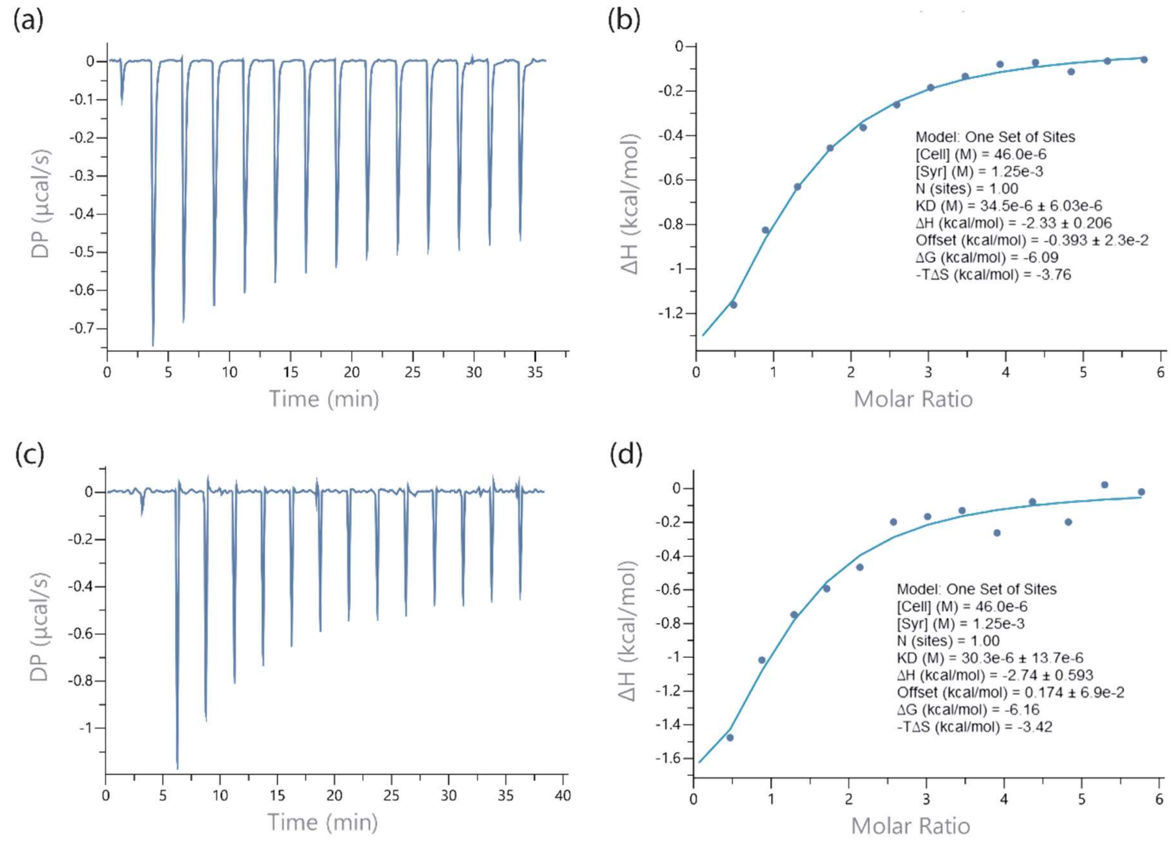

Figure S94. Duplicate isothermal titration calorimetry of TACC3$_{518\text{-}532}$ WT and Aurora-A$_{122\text{-}403\text{-}C290A/C393A}$. (a, c) Raw heat plots and (b, d) integrated, baseline-corrected heats per injection and corresponding fit on the right panels. Listed with each titration are the concentrations of the protein in the syringe and in the cell, as well as the parameters of the fit (stoichiometry *N*, dissociation constant K_d).

TACC3 $_{518\text{-}532\text{-}L/R\text{-}Bph\text{-}IF/fP}$:

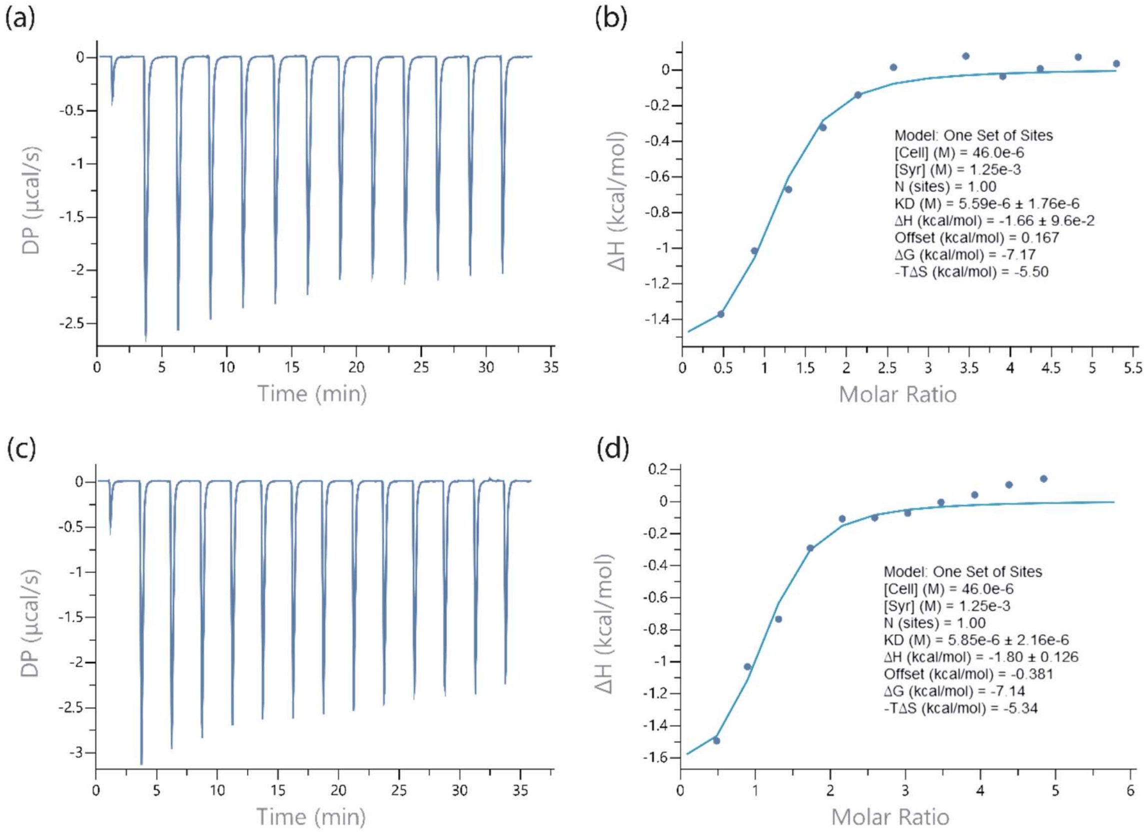

Figure S95. Duplicate isothermal titration calorimetry of constrained TACC3$_{518\text{-}532\text{-}L/R\text{-}Bph\text{-}IF/fP}$ and Aurora-A$_{122\text{-}403\text{-}C290A/C393A}$. (a, c) Raw heat plots and (b, d) integrated, baseline-corrected heats per injection and corresponding fit on the right panels. Listed with each titration are the concentrations of the protein in the syringe and in the cell, as well as the parameters of the fit (stoichiometry *N*, dissociation constant K_d).

TACC3 $_{518\text{-}532\text{-}S/E\text{-}Bph\text{-}IF/fP}$:

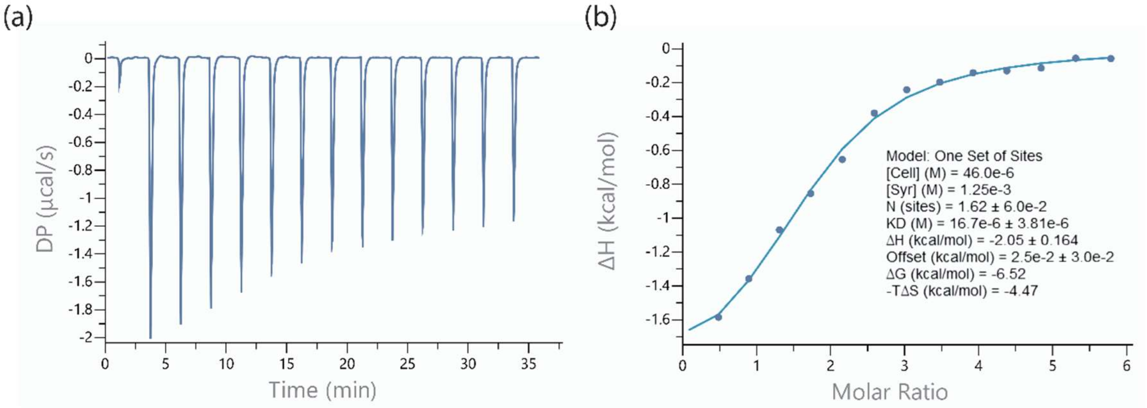

Figure S96. Isothermal titration calorimetry of constrained TACC3$_{518\text{-}532\text{-}S/E\text{-}Bph\text{-}IF/fP}$ and Aurora-A$_{122\text{-}403\text{-}C290A/C393A}$. (a) Raw heat and (b) integrated, baseline-corrected heats per injection and corresponding fit on the right panel. Listed with each titration are the concentrations of the protein in the syringe and in the cell, as well as the parameters of the fit (stoichiometry *N*, dissociation constant K_d).

Variable temperature fluorescence anisotropy titrations (VT-FA)

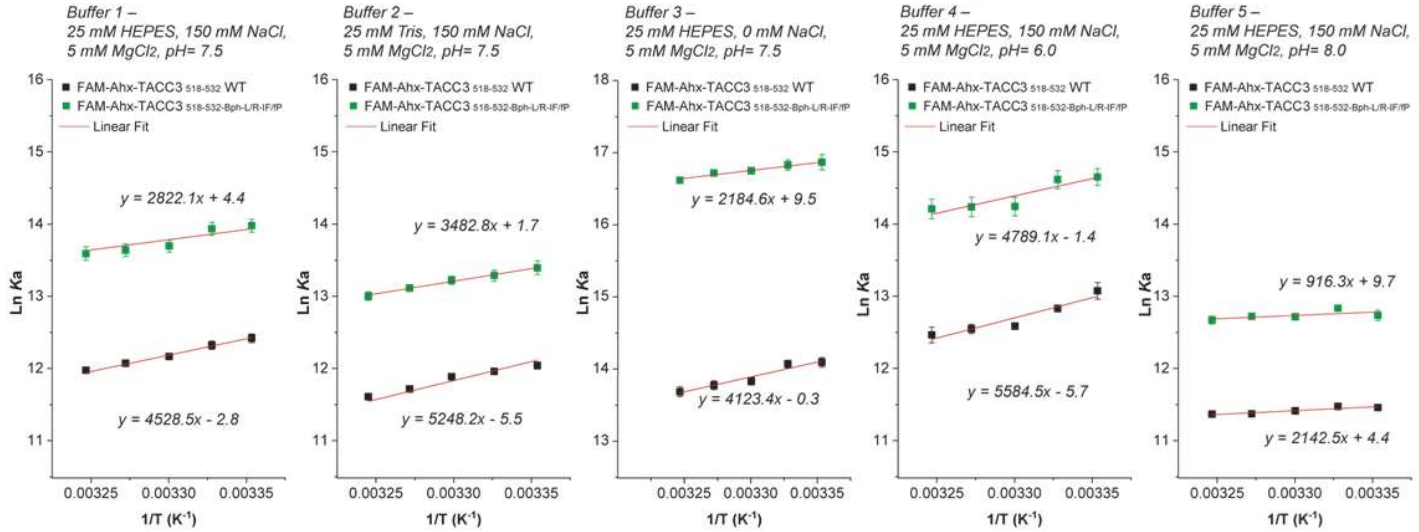

Buffer	$\Delta\Delta G_{Constrained\text{-}WT}$ [c] (kJ mol^{-1})	$\Delta\Delta H_{Constrained\text{-}WT}$ [c] (kJ mol^{-1})	$\Delta T\Delta S_{Constrained\text{-}WT}$ [c] (kJ mol^{-1})
Buffer 1 – 25 mM HEPES, 150 mM NaCl, 5 mM $MgCl_2$, pH= 7.5	-4.50 ± 3.6	14.18 ± 2.5	-17.91 ± 2.4
Buffer 2 – 25 mM Tris, 150 mM NaCl, 5 mM $MgCl_2$, pH= 7.5	-3.17 ± 2.0	14.67 ± 0.4	-17.85 ± 1.6
Buffer 3 – 25 mM HEPES, 5 mM $MgCl_2$, pH= 7.5	-6.82 ± 5.9	16.12 ± 3.0	-22.94 ± 2.9
Buffer 4– 25 mM HEPES, 150 mM NaCl, 5 mM $MgCl_2$, pH= 6.0	-4.06 ± 4.1	7.27 ± 2.1	-11.33 ± 2.0
Buffer 5– 25 mM HEPES, 150 mM NaCl, 5 mM $MgCl_2$, pH= 8.0	-3.02 ± 1.8	10.19 ± 0.9	-13.22 ± 0.9

Figure S97. Solvent effects: FA-VT Van't Hoff analysis of linear FAM-Ahx-TACC3$_{518\text{-}532}$ and constrained FAM-Ahx-TACC3$_{518\text{-}532\text{-}L/R\text{-}Bph\text{-}IF/fP}$. The K_d values were collected from the results of titrating Aurora-A$_{122\text{-}403\text{-}C290A/C393A}$ to 50 nM tracers in different buffers at temperatures between 25- 35 °C to ensure that no thermal unfolding of the protein was induced ($T_{M,\ AurA}$ ~ 45 °C).[10] Represented are the means ± SD of a triplicate experiment. For comparison, linear fit to the data points is shown in red. Note that due to its increased buffering capacity and better stability to temperature variations, HEPES was selected over Tris to perform solvent variations. Similar K_d values were measured in the presence of both buffers at pH= 7.5.

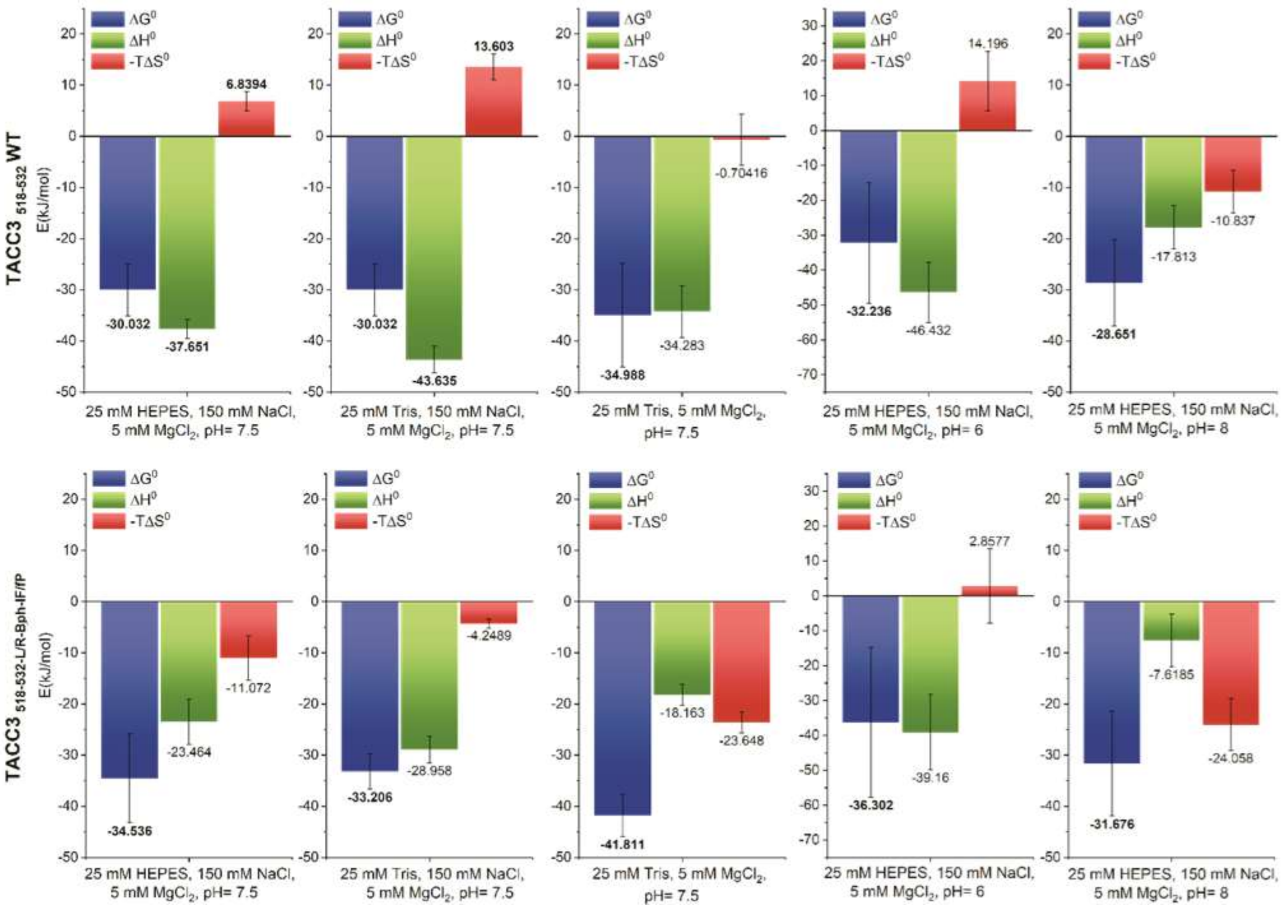

Figure S98. Thermodynamic VT-FA signatures of (a) linear FAM-Ahx-TACC3$_{518\text{-}532}$ and (b) constrained FAM-Ahx-TACC3$_{518\text{-}532\text{-}L/R\text{-}Bph\text{-}IF/fP}$. The K_d values were collected from the results of titrating Aurora-A $_{122\text{-}403\text{-}C290A/C393A}$ to 50 nM tracers in different buffers (Buffer 1 – 25 mM HEPES, 150 mM NaCl, 5 mM $MgCl_2$, pH= 7.5; Buffer 2 – 25 mM Tris, 150 mM NaCl, 5 mM $MgCl_2$, pH= 7.5; Buffer 3 – 25 mM HEPES, 5 mM $MgCl_2$, pH= 7.5; Buffer 4– 25 mM HEPES, 150 mM NaCl, 5 mM $MgCl_2$, pH= 6.0; Buffer 4– 25 mM HEPES, 150 mM NaCl, 5 mM $MgCl_2$, pH= 8.0) between 25- 37.5 °C. The ΔG°, ΔH° and -T*ΔS° were further calculated using the Arrhenius equation and the Gibbs free energy change equation for T= 298 K.

Kinetic Analyses of Kinase Activity

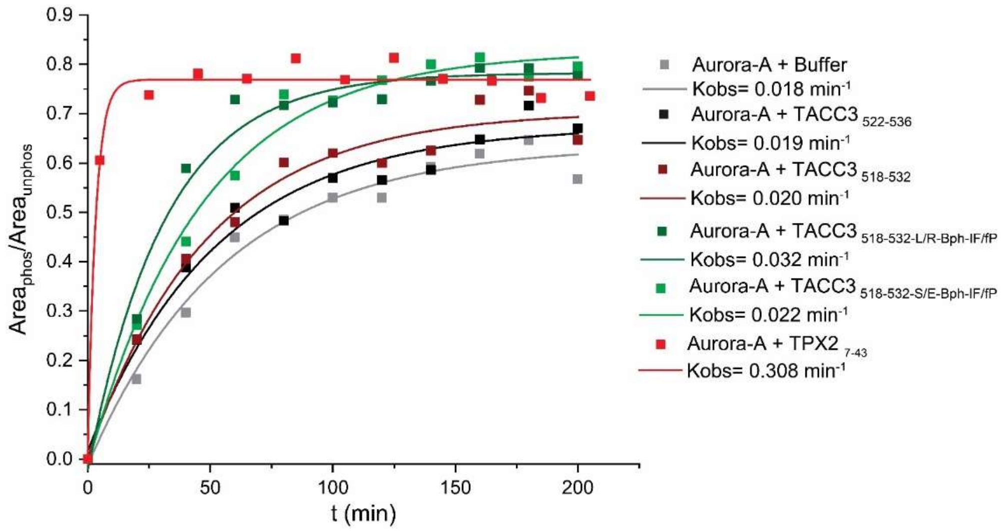

Figure S99. Kinetic analyses of Kemptide phosphorylation by Aurora-A in the presence of different client peptides (errors bars indicate the average of two experiments(100 nM Aurora-$A_{122\text{-}403\text{-}290A/C393A}$, 100 µM peptide, 100 µM ATP and 50 µM kemptide, 40 mM Tris, 150 mM NaCl, 10 mM $MgCl_2$, 1mM DTT, 0.1 mg/ml BSA, 0.01% Tween 20, pH= 7.5 at 25 °C.

FA competition assays in the presence of FAM-Ahx-TPX2 $_{7\text{-}47}$

To test the selectivity of the constrained peptides against the Aurora-A N-lobe $TPX2_{7\text{-}47}$ binding pocket, we conducted further competition experiments in the presence of fluorescently-labeled $TPX2_{7\text{-}47}$. All constrained peptides showed good selectivity for the TACC3 binding pocket, as IC_{50} values against the TPX2 peptide were more than 20-fold higher than those observed against control $TACC3_{522\text{-}563.}$ TPX2 IC_{50} values for all peptides were also 10-fold higher against TPX2 than that measured for the control sequence (shown in black).

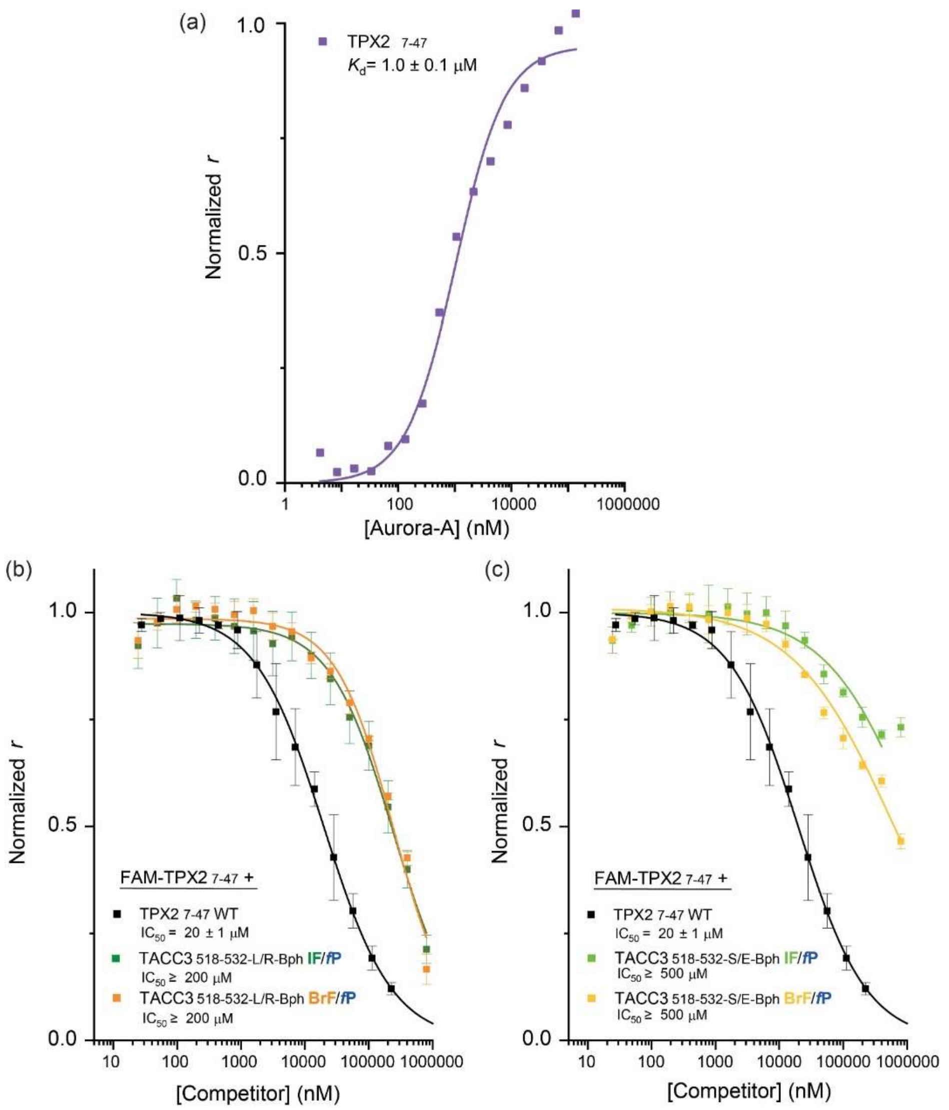

Figure S100. (a) FA direct binding titration of fluorescently-labeled TPX2$_{7\text{-}47}$ with Aurora-A. (b) Competition FA assay of L/R Bph-constrained variants and (c) S/E constrained peptides against TPX2$_{7\text{-}47}$ in the presence of Aurora-A (5 µM Aurora-A $_{122\text{-}403\text{-}C290A/C393A}$, 200 nM N-Myc$_{61\text{-}89}$ Ahx-FAM). All experiments measured in 25 mM Tris, 150 mM NaCl, 5 mM $MgCl_2$, pH= 7.5 at 25 °C.

FA competition assays in the presence of FAM-Ahx-N-Myc$_{61\text{-}89}$

To test the selectivity of the constrained peptides against the Aurora-A N-Myc binding pocket, we conducted competition experiments in the presence of fluorescently-labeled N-Myc$_{61\text{-}89}$. All peptides were able to displace N-Myc at relatively low concentrations. However, as shown below, we noticed that for the S/E constrained peptides there was also a substantial level of nonspecific peptide-tracer association in the absence of the protein contributing to the FA signal (represented in grey for each of the iodo-variants respectively). Based on these results we considered the L/R-constrained peptide a more suitable variant for further experiments, as it showed only marginally higher IC$_{50}$ values.

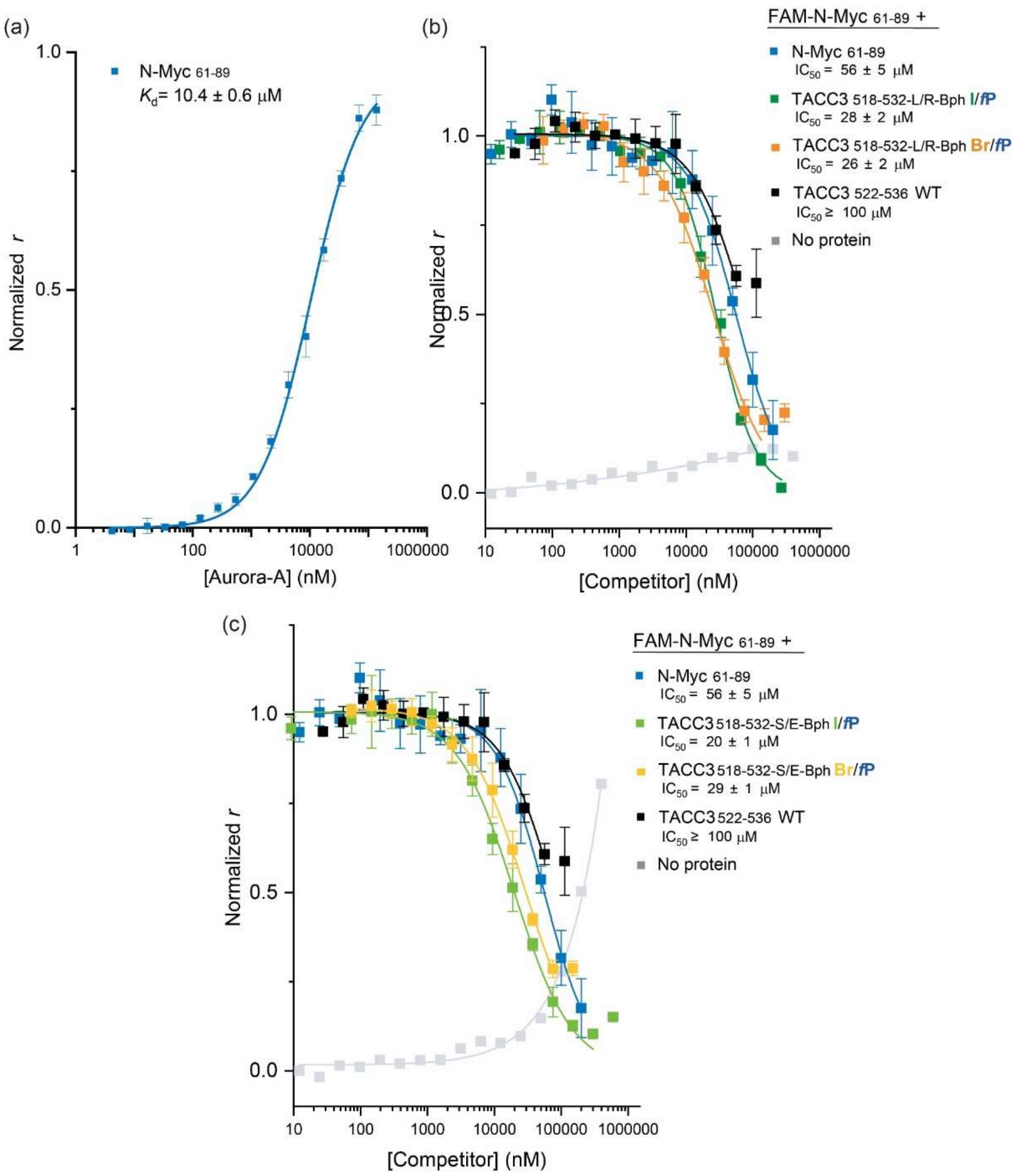

Figure S101. (a) FA direct binding titration of fluorescently-labeled N-Myc$_{61\text{-}89}$ with Aurora-A. (b) Competition FA assay of L/R Bph-constrained variants and (c) S/E constrained peptides against N-Myc $_{61\text{-}89}$ in the presence of Aurora-A (5 µM Aurora-A$_{122\text{-}403\text{-}C290A/C393A}$, 200 nM N-Myc$_{61\text{-}89}$ Ahx-FAM). All experiments measured in 25 mM Tris, 150 mM NaCl, 5 mM $MgCl_2$, pH= 7.5 at 25 °C.

Allosteric inhibition: N-Myc $_{61\text{-}89}$ FA competition assays in the presence of FAM-Ahx- TACC3 $_{518\text{-}532\text{-}L/R\text{-}Bph\text{-}IF/fP}$

An allosteric modulator can affect substrate binding. In its most simple expression, the relationship between a substrate (agonist) and an inhibitory ligand (modulator), as defined for enzymes, is given by the Cheng-Prusoff equation:

$$\frac{IC_{50}}{K_i} = 1 + \frac{[S]}{[K_m]} \qquad Eq.(\mathrm{SI}-1)$$

where, K_i is the inhibitory constant, defined as the equilibrium concentration of an inhibitory ligand when 50% of the receptor sites are occupied if no competing substrate is present. IC_{50} is the concentration at which the inhibitory ligand displaces 50% of the substrate. [S] is the concentration of the substrate used in the binding assay; and K_m is the affinity constant of the substrate, defined as the equilibrium concentration that results in substrate occupying 50% of the receptor sites in the absence of competition.

A corresponding relationship for allosteric ligands was derived by A. Christopoulos and T. Kenakin to study allosterism in G protein-coupled receptors (tertiary complex model), where the equilibrium dissociation constant for the agonist binding (K_B) is changed by an allosteric constant (α) upon binding of the allosteric modulator (A) and similarly, the dissociation constant for the modulator binding (K_A) is changed by a reciprocal α factor upon binding the agonist (B):

$$\mathrm{IC}_{50} = K_B\left[\frac{[A] + K_A}{\alpha[A] + K_A}\right] \qquad Eq.(\mathrm{SI}-2)$$

IC_{50} denotes here the concentration of allosteric agonist needed to produce 50 % inhibition of modulator binding and $[A]$ is the total concentration of the modulator. The relationship between the experimental IC_{50} values measured at different concentrations of the modulator is hyperbolic in nature for any value of $\alpha \neq 1$ or 0, with $\alpha > 1$ indicating positive cooperativity and $\alpha < 1$ indicating negative cooperativity (lower binding). In a full non-competitive model the allosteric constant equals $\alpha = 1$ (allosteric interaction that results in unaltered ligand affinity at the equilibrium), and eq. SI-2 simplifies to:

$$\mathrm{IC}_{50} = K_B \qquad Eq.(\mathrm{SI}-3)$$

where the IC_{50} values for the agonist are independent of the modulator concentration. In addition, in a full orthosteric competitive model (i.e. both substrates compete for the same binding pocket), $\alpha = 0$, and Eq. SI-2 can be re-organized as:

$$\mathrm{IC}_{50} = K_B\left[1 + \frac{[A]}{K_A}\right] \qquad Eq.(\mathrm{SI}-4)$$

which gives a linear correlation between the IC_{50} values for the agonist and the modulator concentration (orthosteric inhibitor).

Based on the IC_{50} vs. [A] correlation experimentally observed it is then possible to discriminate competitive ($\alpha = 0$) from allosteric inhibitors ($\alpha < 1$), and from this latest group, it is possible to discern a mixed inhibition mechanism ($\alpha \neq 1$) from "pure" non-competitive inhibition ($\alpha = 1$).

We decided then to measure the IC_{50} values in FA competition assays of N-Myc $_{61\text{-}89}$ against the fluorescently-labeled FAM-Ahx-TACC3 $_{518\text{-}532\_Bph\_L/R\_IF/fP}$ constrained peptide. This selection, and not the reverse, was motivated by two factors: firstly, the constrained peptide is a more efficient tracer at relatively low protein concentrations, as its K_d is 10-fold lower than that of N-Myc $_{61\text{-}89}$ and secondly, it ensured a starting point for the competition assays where the protein remains functionally active according to our kinase activity assay data. Following are shown the results from this study:

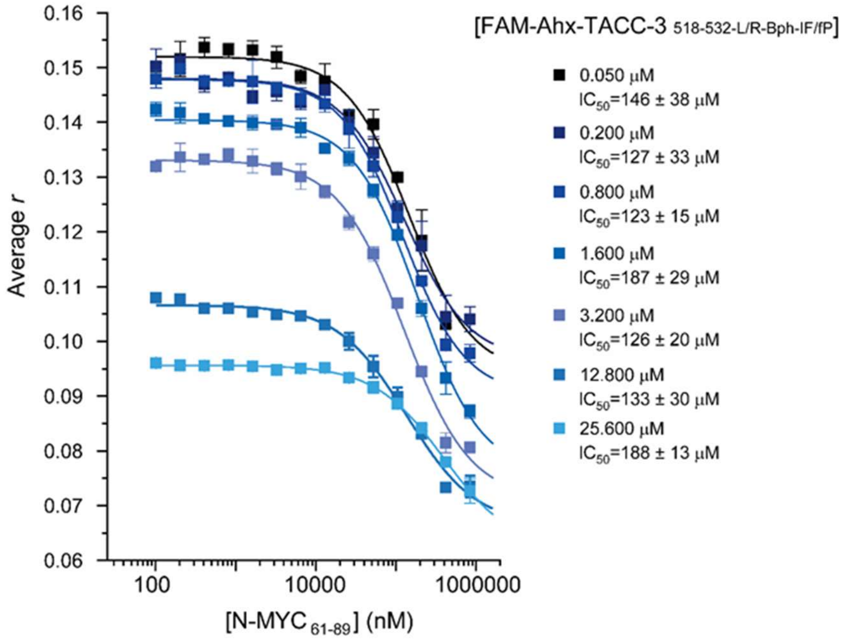

Figure S102. Competition FA assays of N-Myc$_{61\text{-}89}$ against increasing concentrations of TACC3$_{518\text{-}532\text{-}L/R\text{-}Bph\text{-}4I/fP}$ in the presence of Aurora-A$_{122\text{-}403\text{-}C290A/C393A}$ (5 µM). All experiments were measured in 25 mM Tris, 150 mM NaCl, 5 mM $MgCl_2$, pH= 7.5 at 25 °C. Data is shown as the means of the anisotropy signal of a triplicate experiment ± SD at each tracer concentration. From these, IC_{50} values were calculated by fitting a sigmoidal logistic model (Eq. SI-5).

It is worth noting that as the concentration of the tracer was increased, we measured a progressive decay in the raw FA signal. This is explained by the fraction of tracer bound decreasing as [tracer] >> [Aurora-A] (i.e. more tracer is free in excess to the protein which then becomes the limiting reagent). This gave a good indication of the starting point of the titration regime for the experiment, where the concentration of the tracer exceeds so largely that of the protein that the IC_{50} values can not be estimated (the concentration of the bound tracer equals the concentration of the protein regardless of the total tracer concentration in the sample, which only causes a further reduction in the fraction of tracer bound and hence a decrease of the FA signal). Measures beyond a tracer concentration of 25.6 µM were tested in this experiment but, as explained, they did not enable the accurate estimation of the IC_{50} values and were discarded for analysis.

Materials and methods

General materials and methods

All Fmoc-protected amino acids and coupling reagents were purchased from Fluorochem. DMF used for peptide synthesis was ACS grade from VWR and solvents used for purification were HPLC quality and provided by Sigma Aldrich or Fisher.

LC/(ESI+)MS analyses were performed using a Bruker maXis Impact QTOF mass spectrometer (electrospray ionization source), with a Dionex UltiMate 3000 liquid chromatography system (Thermo Scientific), equipped with a Waters Acquity Protein BEH C4 Column (300 Angstrom pore size, 1.7um particle size, 2.1mm x 50mm), running a gradient between water and acetonitrile, both supplemented with 0.1% formic acid.

Preparative purifications have been, in general, performed using RP-HPLC on an Agilent 1260 Infinity system equipped with a diode array UV detector and a Kinetex EVO C18 (250mm x 21.2 mm, Phenomenex) column at 12 ml min^{-1} flow rate. Eluents employed were A: 0.1% TFA in H_2O and B: 0.1% TFA acid in acetonitrile using a gradient of 10-40 or 10-45% B over 45 minutes. Pooled fractions were analysed using LC/(ESI+)MS (Eluents were A: 0.1% formic acid in H_2O and B: 0.1% formic acid in acetonitrile), and fractions corresponding to single chromatographic peaks combined together and freeze dried.

To confirm the final purity of each peptide, analytical RP-HPLC analyses of TACC3 peptides were performed on an Agilent 1260 infinity HPLC system, using an Ascentis® Express C18, 2.7 µm columm (Supelco), and diode array detection at λ = 220 nm. N-Myc peptides were analyzed on a Shimadzu LCMS-2050 system using an Ascentis C18 (250mm x 4.6 mm, 5µm, Supelco) column at 1.5 ml/min flow rate, with diode array detection at λ = 214 nm.

In addition, high resolution mass spectrometry analyses, (HR-(ESI+)LC/MS), used to confirm the identity of each peptide, were performed on Bruker maXis II™ ESI–QToF mass spectrometer.

Solid phase peptide synthesis

Peptides were synthesised using a Liberty Blue microwave-assisted automated peptide synthesiser (CEM Corporation) on a rink amide 100-200 mesh MBHA resin (238 mg, loading 0.42 mol g^{-1}, 0.1 mmol scale) using standard Fmoc-coupling chemistry and 5 eq. amino acid excess and DIC/Oxyma (5 mol eq. each) as coupling reagents. Single couplings were performed at 90 °C for 60s. Arginine was double coupled at 75 °C for 5 minutes. Deprotection was accomplished by double treatment with 20/5% piperidine/formic acid *v/v* in DMF at 90 °C for 1 minute to prevent aspartimide formation. Coupling efficiency was monitored, in all cases, using UV absorbance after Fmoc deprotection.

For FAM-Ahx- labeled peptides, once the automated synthesis was completed, the Fmoc deprotected peptides were transferred into manual SPE synthesis fritted tubes fitted with taps and then washed with DMF (2 x 5 mL x 2 min). 5 mol eq. of Fmoc-Aminohexanoic acid, 5 eq of OXYMA and 5 eq of DIC were added to each resin and the mixtures left to react on the rotary shaker for 1 h. Once completed, the solutions were drained and the resins were thoroughly washed with DMF (4 x 5 mL x 2min). The Fmoc group was then removed using 2 mL of a 20% piperidine solution in DMF (2 x 3 mL x 15 min) and the resin washed with DMF (4 x 5 mL x 2 min). Then, 3 mol eq. of 5(6)-carboxyfluorescein, DIC and OXYMA were added, and the mixtures were left to react in the rotary shaker (protected from any source of light) overnight. Upon completion, the mixtures were drained and the resins washed sequentially with DMF (2 x 5 mL x 2 min),

20% piperidine in DMF (2 x 3 mL x 20 min) and CH_2Cl_2 (4 x 5 mL x 2 min), before being finally left to dry under reduced pressure.

Alternatively, when needed, N-terminal acetylation of the peptides was accomplished by using 50 mol equivalents of acetic anhydride and DIPEA in DMF (2 x 1mL x 30 minutes).

Peptides were cleaved using a TFA:TIPS:H_2O:DODT% v/v solution for 3 hours at room temperature (2 mL per 100 mg of peptide-resin). The cleavage cocktails were filtered over Et_2O (30 mL) and the final crude materials left to precipitate overnight at -20 °C. The precipitated peptides were recovered by centrifugation (6000 r.p.m; 5 min) and washed with chilled Et_2O (-20 °C; 2 x 15 mL). Finally, the solid crude materials were dissolved in a mixture of H_2O:MeCN 75:25% v/v and lyophilized.

Peptide constraint using 2,3-Dibromomaleimide (mal)

Crude linear TACC3 peptides (35 mg; ~0.022 mmol) were dissolved in 6 mL of phosphate buffer (40 mM, 200 mM NaCl, pH= 7.5)/MeCN 50/50% v/v and incubated in the presence of 1.1 mol equivalent of TCEP (7.3 mg; 0.025 mmol) for 1 hour at room temperature. Then, 1.1 mol eq of 2,3-dibromomaleimide (6.5 mg; 0.025 mmol) was added to each reaction in one solid portion and the mixtures allowed to react for another hour. Then, sample aliquots of the reactions were withdrawn and the formation of the target products was verified by LC/(ESI+)MS. Upon completion, samples were freeze-dried and redissolved in H_2O/MeCN/TFA 90/10/0.1% v/v for preparative HPLC purification.

Peptide constraint using 4,4'-bis(bromomethyl)biphenyl (Bph)

Crude linear TACC3 peptides (35 mg; ~0.022 mmol) were dissolved in 6 mL of phosphate buffer (40 mM, 200 mM NaCl, pH= 7.5)/MeCN 50/50% v/v and incubated in the presence of 1.1 mol eq, of TCEP (7.3 mg; 0.025 mmol) for 1 hour at room temperature. Then, 100 µl of DIPEA and 1.2 mol eq of 4,4'-bis(bromomethyl)biphenyl (9 mg; 0.026 mmol) were added to each reaction, and the mixtures allowed to react for 2 hours. Upon completion, samples were freeze-dried and the solid residue was washed three times with cold Et_2O (-20 °C, 15 mL) to clean the unreacted bromide. Then, a sample of the crude materials was withdrawn and the presence of the desired constrained products was assessed by LC/(ESI+)MS. The bulk of the reaction crude material was then redissolved in H_2O/MeCN/TFA 90/10/0.1% v/v for preparative HPLC purification.

MW assisted peptide constraint using 1,8-dibromooctane (Oct) and 2,2'-(Ethylenedioxy)diethyl ditosylate (PEG).

Constraining using 1,8-dibromoctane was carried out directly in the solid-phase using an Anton Paar Monowave 50 synthesis reactor. To this purpose, the resin containing the precursor Cys-Trt protected peptide (0.1 mmol) was selectively deprotected in the presence of diluted TFA/DCM (7% v/v, 5X, 2 mL, 1 min), neutralized for 30 min in the presence of DIPEA/DCM 5% v/v (10 mL), washed (DCM, 5X, 5 mL) and dried under vacuum. The resulting Cys thiol-free linear peptide was then transferred into a suitable microwave reaction vial containing a stir-bar, to which NaI (1.5 g, 100 mol eq.) and DMF (5 mL) were subsequently added while keeping the mixture stirring at all times. Then, TCEP (3 mol eq., 75 mg) was added in one solid portion and the resulting suspension bubbled under nitrogen for 15 min. DIPEA (35 mol eq.) was then added and the resulting mixture was allowed to stir under N_2 for another 30 min. Then 3 mol eq. of either 1,8-dibromooctane (55 µL; **Oct**) or 2,2'-(Ethylenedioxy)diethyl ditosylate (150 mg; **PEG**) were added to the mixture and the vial sealed with a rubber cap. The suspensions were reacted under microwave irradiation using a temperature gradient from rt to 110°C in 5 minutes, followed by 15 minutes at 110°C.

Once completed, the mixtures were transferred into SPPS fritted reaction vessels and washed with H_2O (5X, 10 mL, 2 min), DMF (5X, 5mL, 2 min) and DCM (5X, 5 mL, 2 min). The clean resins were dried under vacuum and the cyclic peptides cleaved from the resin following the general procedures described in previous sections. After Et_2O precipitation, the crude materials were freeze-dried and redissolved in H_2O/MeCN/TFA 90/10/0.1% v/v for preparative HPLC purification.

Peptide oxidation (ox).

All disulfide peptide variants (ox) were produced *in situ* from their free-thiol solid precursors. For this, 2 mg of the pure peptide materials were dissolved in aqueous DMSO 33% v/v to a 10 mM concentration and were left open to air oxidation at room temperature overnight. Quantitative formation of the desired disulfide products was verified by accurate mass HR-LC/(ESI+)MS spectroscopy and the stock solutions of the peptides were employed for FA competition assays and IC_{50} evaluation without further additional purification.

Fluorescence anisotropy (FA) assays – General information.

All assays were performed using 384-well plates(Greiner Bio-one, UK). Aurora-A $_{122\text{-}403\text{-}290A/C393A}$ protein was produced as described previously.[11] All samples were prepared in 25 mM Tris,150 mM NaCl, 5 mM $MgCl_2$, pH= 7.5, unless otherwise stated, and tested in triplicate using an EnVision^{TM} 2103 MultiLabel plate reader (PerkinElmer; Waltham, MA, USA).The parameters were set as follows: Excitation wavelength = 480 nm (30 nm bandwidth) and emission wavelength = 535 nm (30 nm bandwidth). Measured data were processed and analysed as previously described.[12] Specifically, the perpendicular intensity (*P*) and parallel intensity (*S*) were subtracted by the control values and used for calculations of intensity and anisotropy using the following Equations 1, 2, 3, 4, and 5:

$$I = (2PG) + S \qquad Eq.\,(\mathrm{SI}-5)$$

$$r = (S - PG) \qquad Eq.\,(\mathrm{SI}-6)$$

$$L_b = \frac{r - r_{min}}{\lambda(r_{max} - r) + r - r_{min}} \qquad Eq.\,(\mathrm{SI}-7)$$

$$y = \frac{(k + x + [FL]) - \sqrt{(k + x + [FL]^2 - 4 * x * [FL])}}{2} \qquad Eq.\,(\mathrm{SI}-8)$$

$$y = r_{max} + \frac{r_{min} - r_{max}}{1 + (x/x_0)^p} \qquad Eq.\,(\mathrm{SI}-9)$$

Where *I* = total intensity; *P* = perpendicular intensity; *S* = parallel intensity; *G* = instrument factor; *r* = anisotropy; L_b = ligand bound fraction; λ = change in intensity between bound and unbound states which was 1 in this instance, [*FL*] = fluorescent ligand concentration; *k* = K_d; *y* = L_b *[*FL*] and *x* = added protein concentration.

FA direct binding assays.

Direct FA binding assays were performed with the concentration of Aurora-A $_{122\text{-}403\text{-}290A/C393A}$ typically starting from 700 µM, diluted over 24 points in a 1/2 regime using the FAM-Ahx-labeled peptides at a final fixed concentration of 50 nM per well.

FA competition assays.

FA competition assays were performed using the same plates and buffer as described above, with the initial concentration of the inhibitor typically starting from 1.2-2.4 mM depending on solubility, and diluted over 14 points in a 1/2 regime. Unless otherwise stated, FAM-Ahx-TACC3 $_{522\text{-}536}$ tracer and Aurora-A$_{122\text{-}403\text{-}290A/C393A}$ were fixed at final concentrations of 200 nM and 5 µM per well respectively. Plates were read after 4 h of incubation to enable appropriate sample equilibration. To prevent sample oxidation, when sulfhydryl peptides were analyzed DTT was added to the buffer (5 mM). In competition mode, the average anisotropy and the average standard deviation of the values derived from equation SI-6 were calculated and fit to a sigmoidal logistic model (equation SI-9) using Origin 2021. When using peptides for which full displacement of the tracer was not observed, the minimum anisotropy measured for a control TACC3 full competitor (Ac-CEP192$_{500\text{-}533}$, r_{min} = -0.029) was used to restrain the fitting. For consistency, all the data plotted is given normalized to the anisotropy window of a full competitor (r_{max} = -0.006 ; r_{min} = -0.029). Results are reported as IC_{50} ± SD, with data points representing the mean of three replicates and error bars indicating the corresponding SD.

Molecular dynamics (MD) analyses: All peptide–protein complexes were subjected to duplicate MD simulations using YASARA structure.[13] Starting from the reported x-ray crystal structure of one molecule of WT TACC3 $_{522\text{-}563}$ in complex with Aurora-A $_{122–403\ C290A/C393A/D274N}$ (PDB: 5ODT), single amino acid modifications were introduced by direct replacement of the relevant atoms within the structure. When needed, biphenyl constraints were modeled in YASARA by swapping the pertinent amino acids for cysteines and connecting them to biphenyl- fragments. In addition, in TACC3 $_{518\text{-}532}$ variants residues 518-521 were modelled prior to any analysis using helical dihedral angles (res 518–521 are not resolved in the original crystal structure), and then minimised structures were generated using the energy minimization function with default settings. The modelled complexes were subjected to MD simulations using YASARA structure macro for fast MD run (www.yasara.org/md_runfast.mcr).[14] Briefly, the AMBER14 forcefield was used and the temperature was set as 298.0 K with the time-step set at 1*2.50 fs per frame. Each complex was run for 150 ns (600 frames). Minimum energy, average structures, and per-residue number of contacts between the interactors were analysed from these experiments (www.yasara.org/md_analyze.mcr), and figures created using the same software.

Peptide NMR analysis – General information: Peptide structure NMR studies were recorded on a Bruker AV4 NEO 11.75 T (500 MHz ^{1}H) NMR spectrometer (500-4C) at either 278 or 298 K, using water suppression by means of excitation sculpting with gradients using perfect echo.[15,16] ^{1}H-NMR spectra were obtained at 500 MHz using 128 scans with a relaxation delay of 1 s. ^{13}C{^{1}H}-NMR was obtained at 126 MHz with 0.8 s of relaxation delay. Bi-dimensional ^{1}H-^{1}H TOCSY experiments were performed using mixing times of 20 and 80 ms, a spectral width of 5,000 Hz in both dimensions and a minimum of 16 transients with 16 x 512 increments. Final minimum FT size = 2048 x 1024 points. Bi-dimensional ^{1}H-^{1}H COSY were obtained with a spectral width of 5,000 Hz in both dimensions using a minimum of 16 transients with 16 x 512 increments. Final minimum FT size = 2048 x 1024 points. Bi-dimensional ^{1}H-^{13}C HSQC experiments were performed with a spectral width of 5,000 x 10,000 Hz in both dimensions and a minimum of 64 transients with 64 x 64 increments. Final minimum FT size = 4096 x 1024 points. ^{1}H-^{1}H NOESY experiments were performed with a mixing time of 300 ms, a spectral width of 5,000 Hz in both dimensions and a minimum of 16 transients with 16 x 512 increments. Final minimum FT size = 2048 x 1024 points. All NMR data was processed using Topspin 4.1.4 and Mestrenova analysis software.

Peptide NMR analysis – sample preparation: For NMR analysis, 4 mg of the pure peptides were dissolved in 0.55 mL of Aq. Buffer/D_2O 90/10 v/v% to achieve a minimum final peptide concentration of 4 mM (Aq. Buffer: Potassium phosphate 25 mM, NaCl 50 mM, $MgCl_2$ 5mM, pH= 7.5 for control and constrained variants or Potassium phosphate 25 mM, NaCl 50 mM, MgCl2 5mM, 2 mM DTT, pH= 7.5 for free-sulfhydryl variants). All samples were filtered and degassed before their analysis. For each sample, a full set of experiments was done, where ^1H-NMR, ^1H-^1H TOCSY (20 and 80 ms) and ^1H-^1H COSY were employed to assign the identity of each amino acid present in the peptide sequence, and ^1H-^1H NOESY and ^1H-^1H ROESY were employed to establish the inter-residue connectivity (NH-NH-i, and NH-Cα-i walk-throughs) and identify their corresponding spatial correlations between residues. Folded and unfolded ^1H-^{13}C{1H} HSQC NMR was used in all cases to characterize the ^{13}C nuclei and to support full ^1H assignation.

Isothermal titration calorimetry (ITC). ITC experiments were carried out using a MicroCal PEAQ-ITC system (Malvern Panalytical) at 25 °C. A stock solution of Aurora-A $_{122\text{-}403\text{-}290A/C393A}$ was dialyzed into ITC buffer overnight (25 mM Tris buffer, pH= 7.5; 150 mM NaCl; 5 mM $MgCl_2$; 5 % v/v glycerol), and the same buffer was employed to resuspend a solid portion of the testing peptides. For each experiment, initially, the cell was filled with 275 µl of Aurora-A$_{122\text{-}403\text{-}290A/C393A}$ (46 µM) and the syringe was loaded with the corresponding peptide at 1.25 mM concentration. An initial injection of 0.5 µL was then followed by 13 injections of 3 µL, every 150 s, with a constant syringe rotation speed of 750 rpm throughout. The signal for the enthalpy of dilution of each peptide into buffer was independently measured in a control titration experiment and then subtracted from the experimental binding traces. The K_d, ΔG°, ΔH° and -TΔS° values were determined via the MicroCal PEAQ-ITC analysis software (Malvern Paranalytical) using a single-site binding model. For all experiments, the stoichiometry was fixed at N=1 for curve fitting purposes except for constrained peptide S/E_Bph_IF/fP, which was not restrained and showed an N= 1.62. The values reported are either the mean of duplicate measurements ± standard deviation or, where only one experiment was performed, ± the error of curve fitting.

Variable-temperature fluorescence anisotropy (VT-FA) Van't Hoff analysis. Variable temperature-fluorescent anisotropy assays were performed in 384-well plates (Greiner Bio-one) with Aurora-A $_{122\text{-}403\text{-}290A/C393A}$ protein dialyzed into different assay buffers before use. In this experiment, we prepared five buffers, including:

Buffer 1 – 25 mM HEPES, 150 mM NaCl, 5 mM $MgCl_2$, pH= 7.5,
Buffer 2 – 25 mM Tris, 150 mM NaCl, 5 mM $MgCl_2$, pH= 7.5,
Buffer 3 – 25 mM HEPES, 5 mM $MgCl_2$, pH= 7.5,
Buffer 4– 25 mM HEPES, 150 mM NaCl, 5 mM $MgCl_2$, pH= 6.0,
Buffer 5– 25 mM HEPES, 150 mM NaCl, 5 mM $MgCl_2$, pH= 8.0,

The protein concentration in the first well started at 237 µM in the corresponding buffer, and was diluted over 16 points in a 1/2 dilution regime. The concentration of the labeled peptide was fixed at 50 nM per well. After incubation of the plate at room temperature for 1 h, the plates were read at increasing intervals of 2.5°C, following a minimum period of 5 min equilibration at each temperature. The data was processed as previously described and the ln K_a at each temperature plotted against 1/T (K). The ΔG°, ΔH° and -T*ΔS° were calculated according to Van't Hoff's equation (6):

$$\ln K_a = \left(\frac{\Delta H^\circ}{R}\right)\frac{1}{T} + \frac{\Delta S^\circ}{R} \qquad (6)$$

Results are reported as the mean value from three replicates, with the error bars indicating the corresponding ± SD.

Peptide enzymatic degradation assays. α-chymotrypsin (bovine pancreas) and Trypsin were prepared as stock solutions (1.5 mg/mL) by resuspension of the lyophilized proteins (Sigma) in 10 mM HCl, and frozen into individual aliquots at -20 °C. Peptides were dissolved in digestion buffer (HEPES 25 mM, pH= 7.5) up to a concentration of 1 mg mL^{-1} concentration. The digestion samples were immediately prepared by mixing in a polypropylene microcentrifuge tube an aliquot of the peptide and enzyme stock solutions in cold to achieve a final peptide concentration of 100 µM, and a peptide/enzyme molar ratio of [P]/[E]= 25. Samples were then incubated at 25 °C for 6 hours. At specific time points, 35 µL of each proteolysis reaction was withdrawn from the incubates and quenched with 35 µL of a 2% v/v TFA solution. Stability assays in the presence of Trypsin were carried out following a similar protocol, but incubating the samples at 37 °C in a thermo-shaker for 6 hours. Quenched samples were directly analyzed by analytical RP-HPLC using a relatively slow gradient running from 10% to 70% v/v acetonitrile : H_2O over 10 min. To determine the identity of the digested products, samples were analyzed by HR-LC/(ESI+)MS, using a Bruker maXis Impact QTOF mass spectrometer (electrospray ionization source), with a Dionex UltiMate 3000 liquid chromatography system (Thermo Scientific), equipped with a Waters Acquity Protein BEH C4 Column (300 Angstrom pore size, 1.7um particle size, 2.1mm x 50mm).
The peaks corresponding to the unmodified peptides in the raw analytical chromatograms were baseline corrected and their areas were integrated and normalized to the starting initial area of each peptide in the presence of enzyme (t=0). The resulting data has been fitted to an exponential decay function:

$$r = r_0 + A\,x\,e^{-x/t_1} \qquad \textit{Eq. (SI - 10)}$$

$$\tau = t_1 \ln(2) \qquad \textit{Eq. (SI - 11)}$$

where r_0 is the normalized undegraded peptide area at the plateau, t_1 is the normalized time-constant (min^{-1}) and τ is the half-life of the compound (min). For all fitted data, r_0 was given a lower bound limit of 0. For each peptide, a minimum of three independent assays were performed. In all cases, control samples were run in parallel by subjecting each peptide to the same procedure and conditions in the absence of enzyme. Results are reported as the % of undegraded peptide ± SD, with data points representing the mean of three replicates and error bars indicating the corresponding SD.

Aurora-A autophosphorylation assays. *In vitro* Aurora-A autophosphorylation assays were performed by incubating 2.5 µM of unphosphorylated human Aurora-A $_{122\text{-}403}$ in the presence of 10 µM of the control linear TACC3 $_{522\text{-}536}$ peptide or the constrained variants for 1 hr at room temperature. Assays were carried out in 20 mM Tris, 20 mM $MgCl_2$, 150mM NaCl, pH= 7.5. Reactions were stopped by the addition of SDS-loading buffer and separated by SDS-PAGE. Western blots were performed using an anti-Phospho-Thr288 Aurora-A antibody (1:1000 dilution, Cell Signalling Technology 2914S) and visualised using goat anti-rabbit StarBright Blue 700 secondary (1:1000 dilution, Bio-Rad 12004161) on an iBright system (Thermo Fisher Scientific).

ADP-Glow end-point kinase activity assay. Kinase activity assays in the presence of Aurora-A $_{122\text{-}403\text{-}290A/C393A}$ were performed in a 384-well microplate by pre-mixing the protein (20 nM), kemptide as the kinase substrate (200 µM) and double the desired amounts of the corresponding peptides into 7.5 µL of kinase buffer (40 mM Tris, 150 mM NaCl, 10 mM $MgCl_2$, 1mM DTT, 0.1 mg/ml BSA, 0.01% Tween 20, pH= 7.5). Then, assays were immediately started by the addition of 7.5 µL of ATP 200 µM prepared in the same buffer (Promega #V915A), and samples were incubated at room temperature for 45 min. Then, 5 µL of each sample was transferred to a clean 384-well microplate, and the reactions stopped by addition of 5 µL ADP-Glo™ Reagent (Promega #V912C) followed by incubation at room temperature for 30 min. Kinase Detection Reagent (10 µL) (Promega #V917A) was added to convert ADP to ATP by incubating at room temperature for 45 min, and then the luminescence was recorded immediately. All values are given as the means ± SD at each concentration of the peptides from duplicate experiments.

Kinase activity kinetics. Kinase activity assays were performed by premixing in a low volume LC/MS vial 100 µl of Aurora-A $_{122\text{-}403\text{-}290A/C393A}$ (300 nM) with 100 µl of a stock solution of the corresponding peptides (300 µM) in kinase buffer (40 mM Tris, 150 mM NaCl, 10 mM $MgCl_2$, 1mM DTT, 0.1 mg/ml BSA, 0.01% Tween 20, pH= 7.5). Then, the samples were left to equilibrate at 25 °C for 15 minutes in the temperature-controlled sample loading chamber of the LC/MS system. Once equilibrated, assays were started by the addition of 100 µl of a mixture of ATP (300 µM) and kemptide (150 µM), and 5 µl of the mixture was immediately measured by LC/MS. Each sample was re-injected at 20 min time intervals for a total period of 200 min. BPI chromatograms were automatically integrated to calculate the areas under peaks for unmodified ($Area_{unphos}$; $[M+H]^{+}$= 813.50 Da ; $[M+Na]^{+}$= 835.50 Da, $[M+2H]^{2+}$= 407.25 Da) and phosphorylated kemptide ($Area_{phos}$; $[M+H]^{+}$= 893.50 Da ; $[M+Na]^{+}$= 915.50 Da, $[M+2H]^{2+}$= 447.25 Da). The automatically calculated areas were converted to ratios in Origin and plotted as the change in the total substrate phosphorylation over time.

Protein Expression. Human Aurora-A kinase domain 122-403 wild-type or C290A/ C393A mutant in an N-terminal His-tagged vector (pet30TEV) were transformed into *E. coli* RIL cells alongside the pCDF vector encoding lambda phosphatase to generate dephosphorylated protein. The protein was overexpressed in LB, with growth at 37 °C until the O.D. at 600 nm reached 0.6–0.8. Expression was then induced with 0.5 mM IPTG overnight at 20 °C. The pelleted cells were resuspended in 10 ml of ice-cold lysis buffer per litre of grow (50 mM Tris pH 7.5, 250 mM NaCl, 20 mM imidazole, 10% glycerol, 5 mM magnesium chloride, one EDTA-free protease inhibitor tablet per 50 ml of buffer). The resuspended cells were sonicated at 60% amplitude for 10 sec on, 20 sec off, 5 min total. The soluble lysate was collected at 30,000*g* for 45 min in a JA 17 rotor (Beckman Coulter). After filtering (0.45 µm) the solution was loaded on to a 5 ml HisTrap HP (Cytiva) equilibrated in lysis buffer. Any bound protein was eluted using a gradient of lysis buffer containing 500 mM Imidazole. The His-tag was then cleaved overnight using TEV protease in dialysis at 4 °C into 50 mM tris pH 7.5, 250 mM NaCl, 10% glycerol, 5 mM magnesium chloride, 1 mM TCEP. After dialysis the cleaved protein was rebound to the HisTrap HP equilibrated in dialysis buffer. The Aurora still interacted with the HisTrap after cleavage so a gradient of 500 mM imidazole was used to elute off the tag-free protein. The tag-free Aurora was concentrated down (10 kDa cut-off concentrator, Amicon) and loaded onto a HiLoad 16/600 Superdex 200 column (Cytiva) equilibrated with 50 mM tris pH 7.5, 250 mM NaCl, 10% glycerol, 5 mM magnesium chloride, 1 mM TCEP. In the final step Aurora-A was concentrated down again and flash-frozen before storage at -80°C.

Abbreviations

DMF Dimethylformamide
DIPEA N,*N*-Diisopropylethylamine
Oxyma Ethyl cyanohydroxyiminoacetate
DIC N,N'-Diisopropylcarbodiimide
TFA Trifluoroacetic acid
TIPS Triisopropyl silane
DODT 3,6-dioxa-1,8-octanedithiol
TCEP Tris Carboxy Ethyl Phosphene
Tris Tris(hydroxymethyl)aminomethane
HEPES 4-(2-hydroxyethyl)-1-piperazineethanesulfonic acid

References

1 A. Sukhwal and R. Sowdhamini, *Bioinforma. Biol. Insights*, 2015, **9**, 141–151.
2 C. W. Wood, A. A. Ibarra, G. J. Bartlett, A. J. Wilson, D. N. Woolfson and R. B. Sessions, *Bioinformatics*, 2020, **36**, 2917–2919.
3 D. S. Wishart and B. D. Sykes, *J. Biomol. NMR*, 1994, **4**, 171–180.
4 D. S. Wishart, B. D. Sykes and F. M. Richards, *Biochemistry*, 1992, **31**, 1647–1651.
5 D. S. Wishart, B. D. Sykes and F. M. Richards, *J. Mol. Biol.*, 1991, **222**, 311–333.
6 M. Kjaergaard and F. M. Poulsen, *J. Biomol. NMR*, 2011, **50**, 157–165.
7 M. Kjaergaard, S. Brander and F. M. Poulsen, *J. Biomol. NMR*, 2011, **49**, 139–149.
8 S. Schwarzinger, G. J. A. Kroon, T. R. Foss, J. Chung, P. E. Wright and H. J. Dyson, *J. Am. Chem. Soc.*, 2001, **123**, 2970–2978.
9 C. Mycroft, M. Nilsson, G. A. Morris and L. Castañar, *ChemPhysChem*, 2022, **23**, e202200495.
10 *PLOS ONE*, 2013, **8**, e58485.
11 S. G. Burgess and R. Bayliss, *Acta Crystallogr. Sect. F Struct. Biol. Commun.*, 2015, **71**, 315–319.
12 K. Hetherington, Z. Hegedus, T. A. Edwards, R. B. Sessions, A. Nelson and A. J. Wilson, *Chem. – Eur. J.*, 2020, **26**, 7638–7646.
13 E. Krieger and G. Vriend, *Bioinforma. Oxf. Engl.*, 2014, **30**, 2981–2982.
14 E. Krieger and G. Vriend, *J. Comput. Chem.*, 2015, **36**, 996–1007.
15 T. L. Hwang and A. J. Shaka, *J. Magn. Reson.*, 1995, **112**, 275–279.
16 R. W. Adams, C. M. Holroyd, J. A. Aguilar, M. Nilsson and G. A. Morris, *Chem. Commun.*, 2012, **49**, 358–360.

Summary of peptide characterization

Below are tabulated HRMS data for all peptides within this work. Peptide identity was confirmed by the inspection of multiple charge states which are quoted as the monoisotopic peak for the expected (Exp) and observed (Obs) masses.

Table S18. A summary of HRMS data of the synthesized peptides.

Peptide	$[M+H]^{1+}$ Obs	$[M+H]^{1+}$ Exp	$[M+2H]^{2+}$ Obs	$[M+2H]^{2+}$ Exp	$[M+3H]^{3+}$ Obs	$[M+3H]^{3+}$ Exp	$[M+4H]^{4+}$ Obs	$[M+4H]^{4+}$ Exp	$[M+5H]^{5+}$ Obs	$[M+5H]^{6+}$ Exp
TACC3 $_{\text{522-536}}$ Ahx-FAM	2047.8920	2047.8941	1024.4496	1024.4554	683.3022	683.3014				
TACC3 $_{\text{522-536}}$	1618.7741	1618.7707	809.8895	809.8890	-	-				
TACC3 $_{\text{522-536\_(4-I)525F}}$	1745.6674	1745.6752	873.3412	873.3164	-	582.2273				
TACC3 $_{\text{522-536\_(4-Br)525F}}$	1696.6750	1696.6813	848.8389	848.8443	-	566.2319				
TACC3 $_{\text{522-536\_(4-Cl)525F}}$	1652.6972	1652.7318	826.8508	826.8695	-	551.5821				
TACC3 $_{\text{522-536\_(4-F)525F}}$	1636.7326	1636.7613	818.8677	818.8843	-	546.2586				
TACC3 $_{\text{522-536\_(4-CF3)525F}}$	1686.7496	1686.7581	843.8766	843.8827	-	562.9242				
TACC3 $_{\text{522-536\_5}\textit{f}\text{-525F}}$	1709.6909	1709.7315	855.3508	855.3694	-	570.5820				
TACC3 $_{\text{522-536\_(}\textit{p}\text{Bnz) -525F}}$	-	1723.8048	862.3792	862.4060	-	574.9372				
TACC3 $_{\text{522-536\_(}\alpha\text{-Me) -525F}}$		1632.9878	817.3840	816.8968	-	544.9336				
TACC3 $_{\text{522-536\_(4-CN) -525F}}$	-	1643.7660	822.8667	822.3866	-	548.5935				
TACC3 $_{\text{522-536\_F525Y}}$	1635.7390	1635.7735	818.3718	818.3904	-	545.9293				
TACC3 $_{\text{522-536\_F525Y(OMe)}}$	1648.7683	1648.7813	824.8848	824.8943	-	550.6012				
TACC3 $_{\text{522-536\_}\textit{cis}\text{-(4-F)528P}}$	1636.7637	1636.7313	818.8858	818.8843	-	546.2586				
TACC3 $_{\text{522-536\_}\textit{trans}\text{-(4-F)528P}}$	1636.7649	1636.7313	818.8860	818.8843	-	546.2586				
TACC3 $_{\text{522-536\_ D527G/A529G/E530G}}$	1474.7296	1474.7285	737.8699	737.8679						
TACC3 $_{\text{522-536\_V531I}}$	1632.7914	1632.7864	816.8998	816.8969	-	544.9336				
TACC3 $_{\text{522-536\_V531nL}}$	1632.7912	1632.7864	816.8988	816.8969	-	544.9336				
TACC3 $_{\text{522-536\_L532nL}}$	1618.7767	1618.7707	809.8912	809.8890	-	540.2618				
TACC3 $_{\text{522-563}}$ Ahx-FAM	-	5242.5208	-	2621.7640	-	1748.1784	1311.3896	1311.3857	1049.3150	1049.3100
TACC3 $_{\text{522-552}}$ Ahx-FAM	-	3894.7181	1947.8650	1947.8627	1298.9149	1298.9109	974.4377	974.4350	779.7494	779.7498
TACC3 $_{\text{522-532}}$ Ahx-FAM	1761.7654	1761.7646	881.3880	881.3858	-	587.9263				
TACC3 $_{\text{522-542}}$ Ahx-FAM	-	2796.2199	1398.6067	1398.6136	932.7452	932.7448	-	699.8104		
TACC3 $_{\text{518-532}}$ Ahx-FAM	-	2245.0699	1123.0398	1123.038	749.0296	749.0282	-	562.0229		
TACC3 $_{\text{536-563}}$ Ahx-FAM	-	3754.8316	1877.9221	1877.9194	1252.2824	1252.2820	939.4654	939.4633	752.7735	751.7721
TACC3 $_{\text{522-536 E523C/R526C}}$	1539.6470	1539.6434	770.3260	770.3263						
TACC3 $_{\text{522-536 S524C/D527C}}$	1622.7352	1622.7301	811.8729	811.8687						
TACC3 $_{\text{522-536 F525C/P528C}}$	1580.6687	1580.6679	790.8370	790.8376						
TACC3 $_{\text{522-536 R526C/A529C}}$	1597.6611	1597.6509	799.3298	799.3291						
TACC3 $_{\text{522-536 D527C/E530C}}$	1580.7195	1580.7196	790.8330	790.8334						
TACC3 $_{\text{522-536 P528C/V531C}}$	1628.6705	1628.6679	814.8376	814.8376						
TACC3 $_{\text{522-536 A529C/L532C}}$	1840.6707	1640.6670	820.8381	820.8676						
TACC3 $_{\text{522-536 E530C/G533C}}$	1631.7282	1631.7251	819.8664	819.8662						
TACC3 $_{\text{522-536 V531C/T534C}}$	1624.6720	1624.6730	812.8399	812.8401						
TACC3 $_{\text{522-536 L532C/G535C}}$	1654.6854	1654.6836	827.8457	827.8454						
TACC3 $_{\text{522-536 G533C/A536C}}$	1696.7324	1696.7305	848.8693	848.8689						
TACC3 $_{\text{522-536 R526C/V531C}}$	1596.6195	1569.6196	785.3133	785.3134						
TACC3 $_{\text{522-536 E522C/P528C}}$	1527.6572	1527.6566	764.3312	764.3320						
TACC3 $_{\text{522-536 A529C/G535C}}$	1696.7328	1696.7305	848.8691	848.8689						
TACC3 $_{\text{522-536 E523C/R526C mal}}$	1632.6300	1632.6305	816.8185	816.8189						
TACC3 $_{\text{522-536 S524C/D527C mal}}$	1715.7176	1715.7152	858.3623	858.3612						
TACC3 $_{\text{522-536 F525C/P528C mal}}$	1673.6557	1673.6530	837.3317	837.3301						
TACC3 $_{\text{522-536 R526C/A529C mal}}$	1690.5363	1690.6390	845.8216	845.8216						
TACC3 $_{\text{522-536 D527C/E530C mal}}$	1673.7062	1673.7047	837.3562	837.3560						
TACC3 $_{\text{522-536 P528C/V531C mal}}$	1721.6548	1721.6530	861.3317	861.3301						
TACC3 $_{\text{522-536 A529C/L532C mal}}$	1733.6510	1733.6530	867.3318	867.3301						
TACC3 $_{\text{522-536 E530C/G533C mal}}$	1731.7121	1731.7101	866.3596	866.3587						
TACC3 $_{\text{522-536 V531C/T534C mal}}$	1717.6613	1717.6581	859.3341	859.2242						
TACC3 $_{\text{522-536 L532C/G535C mal}}$	1747.6726	1747.6687	874.3404	874.3380						
TACC3 $_{\text{522-536 G533C/A536C mal}}$	1789.7193	1789.7156	895.3630	895.3614						
TACC3 $_{\text{522-536 R526C/V531C Bph}}$	1747.6995	1747.6978	874.3541	874.3526						
TACC3 $_{\text{522-536 E522C/P528C Bph}}$	1776.7747	1776.7720	888.8909	888.8896						
TACC3 $_{\text{522-536 A529C/G535C Bph}}$	1874.88127	1874.8088	937.9107	937.9080						
TACC3 $_{\text{518-532}}$	1815.9302	1815.9487	908.4679	908.4780						
TACC3 $_{\text{518-532 K521C/D527C}}$	1178.8347	1778.8452	889.9194	889.9262						
TACC3 $_{\text{518-532 L520C/R526C}}$	1752.7757	1752.7819	876.8896	876.8946						
TACC3 $_{\text{518-532 S524C/E530C}}$	1805.8815	1805.8925	903.4446	903.4499						
TACC3 $_{\text{518-532 K521C/D527C Bph}}$	1956.91	1956.9234	978.9629	978.9653	-	652.9793				
TACC3 $_{\text{518-532 L520C/R526C Bph}}$	1930.8516	1930.8601	965.9299	965.9337	-	644.2916				
TACC3 $_{\text{518-532 S524C/E530C Bph}}$	-	1983.9707	992.4850	992.4890	661.9909	661.9951				
TACC3 $_{\text{518-532 L520C/R526C Oct}}$	1862.8926	1862.8914	931.9523	931.9494	621.6359	621.6353				
TACC3 $_{\text{518-532 S524C/E530C Oct}}$	-	1916.0020	958.5075	958.5046	639.3308	639.3389				
TACC3 $_{\text{518-532 L520C/R526C PEG}}$	1866.8514	1866.8500	933.9295	933.9286	622.9550	622.9548				
TACC3 $_{\text{518-532 S524C/E530C PEG}}$	1919.9636	1919.9605	960.4884	960.4839	640.6590	640.4839				
TACC3 $_{\text{518-532 L520C/R526C Bph (4-I)525F}}$	2056.7629	2056.7568	1028.8854	1028.8820	686.2569	686.2571				
TACC3 $_{\text{518-532 S524C/E530C Bph (4-I)525F}}$	-	2108.8601	1055.4433	1055.4373	703.9634	703.9606				
TACC3$_{\text{518-532-L520C/R526C Bph (4-Br)525F}}$	2008.7747	2008.7707	1004.8922	1004.8890	670.2620	670.2617				
TACC3$_{\text{518-532 S524C/E530C Bph (4-Br)525F}}$	-	2061.8812	1031.4981	1031.4442	687.9988	687.9653				
TACC3$_{\text{518-532 L520C/R526C Bph (4-I)525F/(4-F)528P}}$	2074.7497	2074.7474	1037.8812	1037.8773	692.2538	692.2540				

**TACC3 $_{518\text{-}532}$ S524C/E530C Bph (4-I)525F/(4-F)528P**	**2127.8625**	**2127.8579**	**1064.4368**	**1064.4326**	**709.9508**	**709.9575**				
**TACC3 $_{518\text{-}532}$ L520C/R526C Bph (4-Br)525F/(4-F)528P**	**2026.7611**	**2026.7612**	**1013.8872**	**1013.8843**	**676.2577**	**676.2586**				
**TACC3 $_{518\text{-}532}$ S524C/E530C Bph (4-Br)525F/(4-F)528P**	**2079.8737**	**2079.8718**	**1040.4427**	**1040.4395**	**693.9615**	**693.9621**				
**TACC3 $_{518\text{-}532}$ L520C/R526C Bph (4-I)525F/(4-F)528P Ahx-FAM**	**-**	**2503.8686**	**1252.4426**	**1252.4379**	**835.2951**	**835.2944**				
**TACC3 $_{518\text{-}532}$ S524C/E530C Bph (4-I)525F/(4-F)528P Ahx-FAM**	**-**	**2556.9792**	**1278.9918**	**1278.9932**	**853.0006**	**852.9979**				
**TACC3 $_{518\text{-}532}$ L520C/R526C Bph (4-Br)525F/(4-F)528P Ahx-FAM**	**-**	**2455.8825**	**1228.4487**	**1228.4449**	**819.2997**	**819.2990**				
**TACC3 $_{518\text{-}532}$ S524C/E530C Bph (4-Br)525F/(4-F)528P Ahx-FAM**	**-**	**2508.9930**	**1255.0016**	**1255.0002**	**837.0043**	**837.0025**				
**N-Myc$_{61\text{-}89}$**	**-**	**3326.5787**	**1663.7944**	**1663.7930**	**1109.5300**	**1109.5311**	**832.4011**	**832.4001**		
**N-Myc$_{61\text{-}89}$ Ahx-FAM**	**-**	**3755.6999**	**1878.3467**	**1878.3536**	**1252.5671**	**1252.5715**	**939.6745**	**939.6804**		

Analytical HPLC traces and high-resolution mass spectra of synthesised peptides

FAM-Ahx-TACC3 $_{522\text{-}536}$

Sequence: FAM-Ahx-EESFRDPAEVLGTGA

HPLC (λ = 220 nm) tR = 5.8min. HR-QToF (ESI) m/z: [M+2H]2+ Calc. for $C_{94}H_{126}N_{20}O_{32}$: 1024.4496; Found:1024.9554.

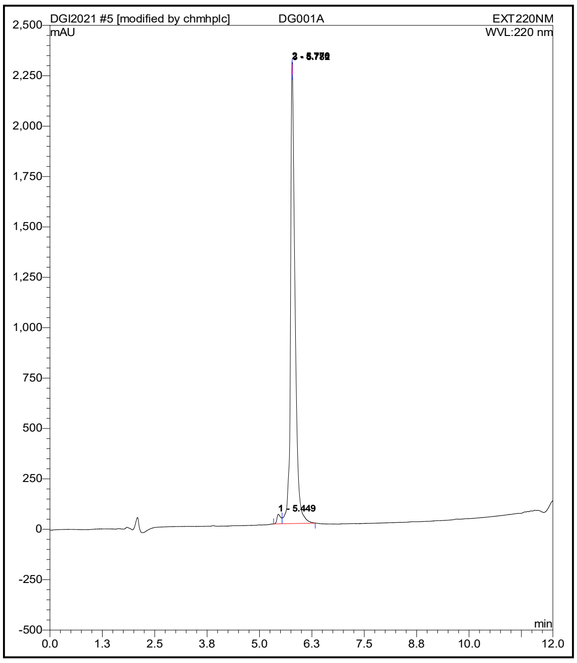

Analytical HPLC trace at λ= 220 nm of purified peptide TACC3 $_{522\text{-}536}$ Ahx-FAM.

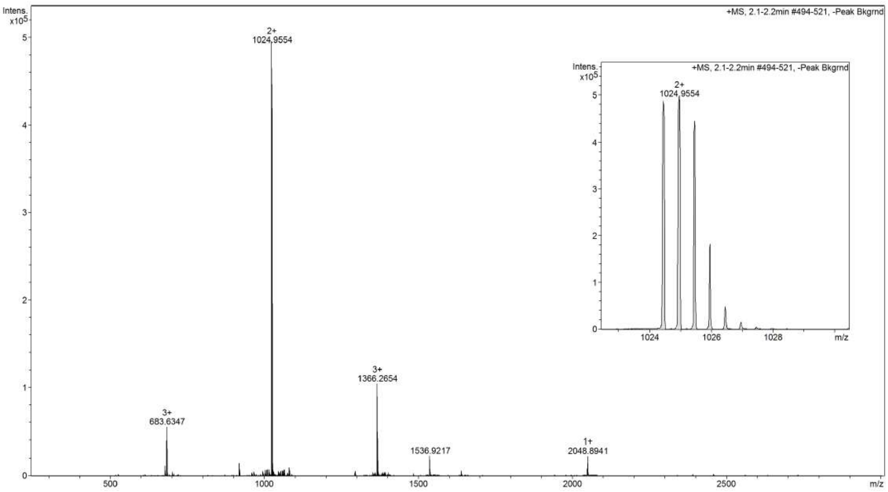

HR-QToF(ESI+)MS analysis of purified peptide TACC3 $_{522\text{-}536}$ Ahx-FAM.

TACC3 $_{522\text{-}536}$

Sequence: Ac- EESFRDPAEVLGTGA

HPLC (λ = 220 nm) tR = 3.165 min. HR-QToF (ESI) m/z: [M+H]+ Calc. for $C_{69}H_{107}N_{19}O_{26}$: 809.8890; Found:809.8895.

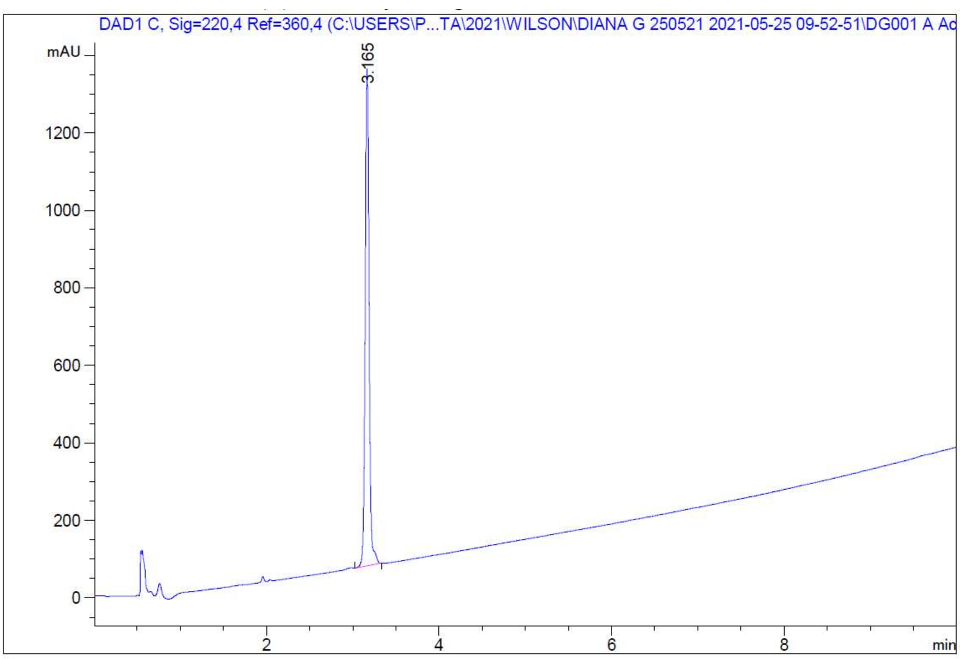

Analytical HPLC trace at λ= 220 nm of purified peptide TACC3 $_{522\text{-}536}$.

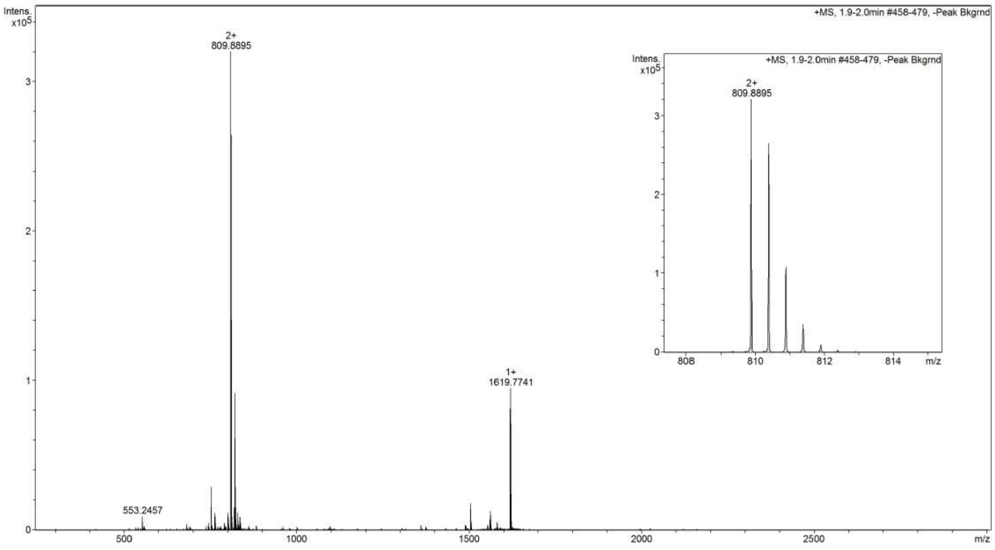

HR-QToF(ESI+)MS analysis of purified peptide TACC3 $_{522\text{-}536}$.

TACC3 $_{522\text{-}536\text{-}(4\text{-}I)Phe525}$:

Sequence: Ac- EES(4I-F)RDPAEVLGTGA

HPLC (λ = 220 nm) tR = 3.657min. HR-QToF (ESI) m/z: [M+2H]2+ Calc. for $C_{69}H_{106}IN_{19}O_{26}$: 873.3174; Found: 873.3072.

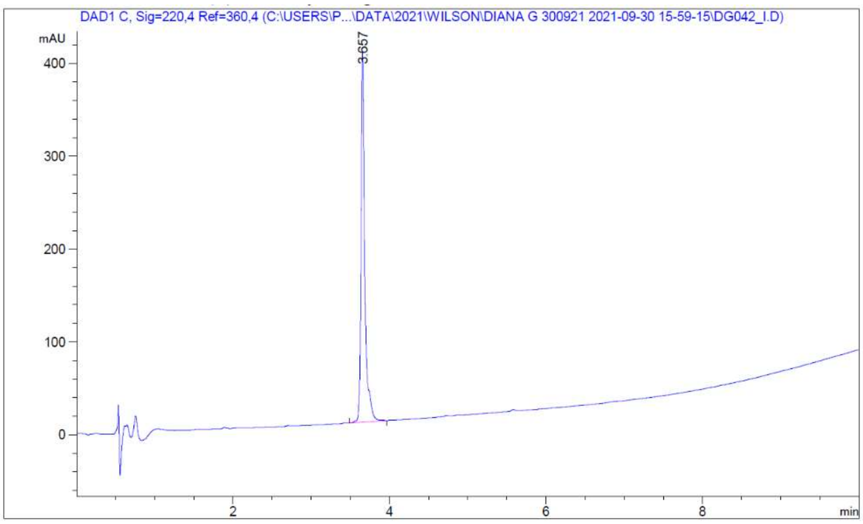

Analytical HPLC trace at λ= 220 nm of purified peptide **TACC3 $_{522\text{-}536\_(4\text{-}I)525F}$**

.

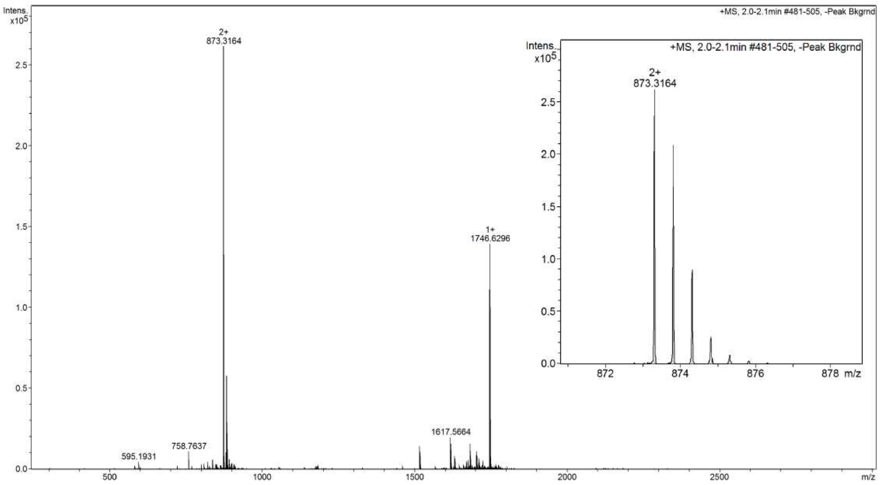

HR-QToF(ESI+)MS analysis of purified peptide **TACC3 $_{522\text{-}536\_(4\text{-}I)525F}$.**

PEPTIDE TACC3 $_{522\text{-}536\_(4\text{-}Br)525F}$:

Sequence: Ac- EES(4Br-F)RDPAEVLGTGA

HPLC (λ = 220 nm) tR = 3.490min. HR-QToF (ESI) m/z: [M+2H]2+ Calc. for $C_{69}H_{106}BrN_{19}O_{26}$: 849.8443; Found: 849.8397.

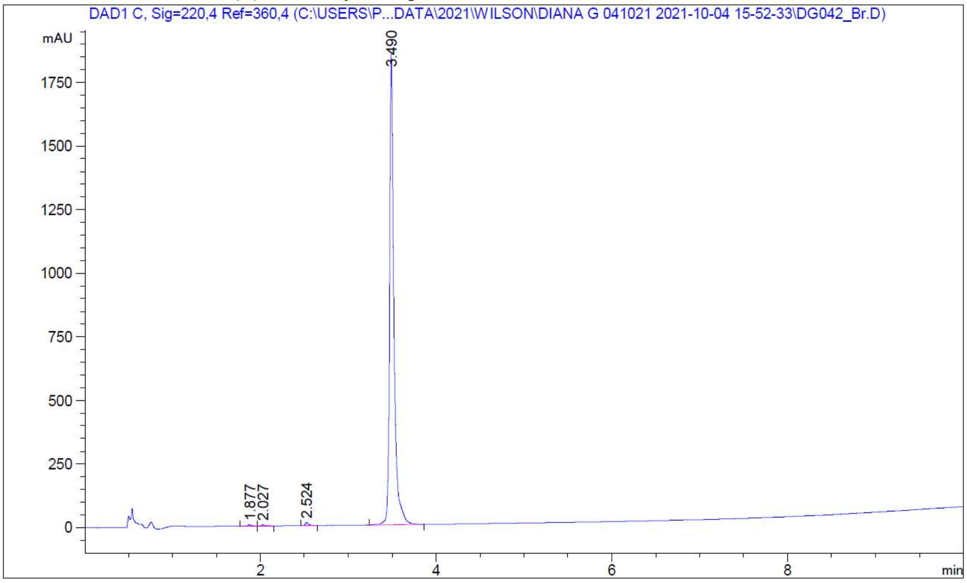

Analytical HPLC trace at λ= 220 nm of purified peptide **TACC3 $_{522\text{-}536\_(4\text{-}Br)525F}$**.

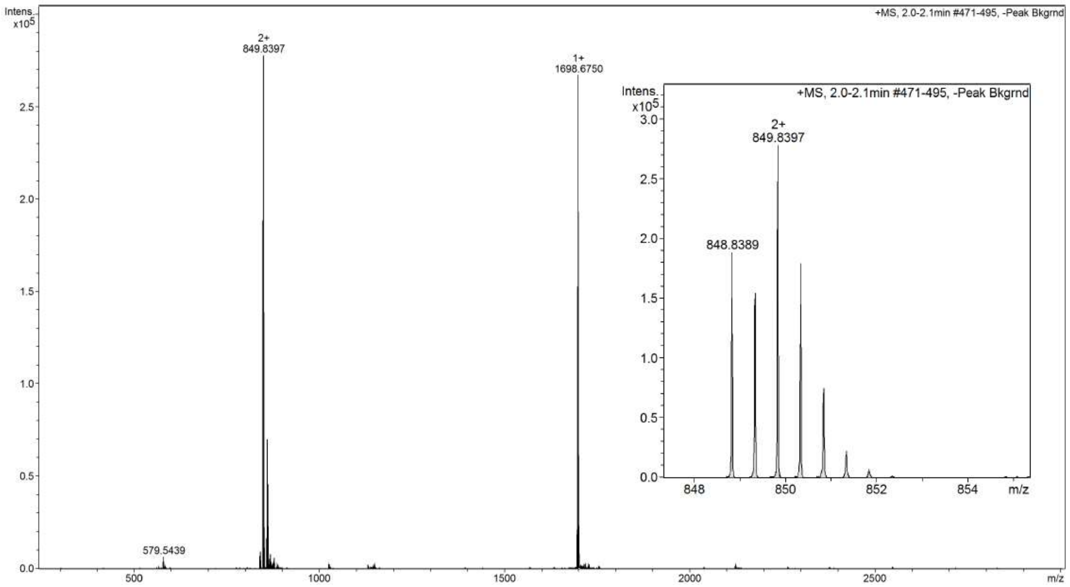

HR-QToF(ESI+)MS analysis of purified peptide **TACC3 $_{522\text{-}536\_(4\text{-}Br)525F}$**

TACC3 $_{522-536\_(4-Cl)525F}$**:**

Sequence: Ac- EES(4-Cl)RDPAEVLGTGA

HPLC (λ = 220 nm) tR = 3.442min. HR-QToF (ESI) m/z: [M+2H]2+ Calc. for $C_{69}H_{106}ClN_{19}O_{26}$: 826.8695; Found: 826.8508.

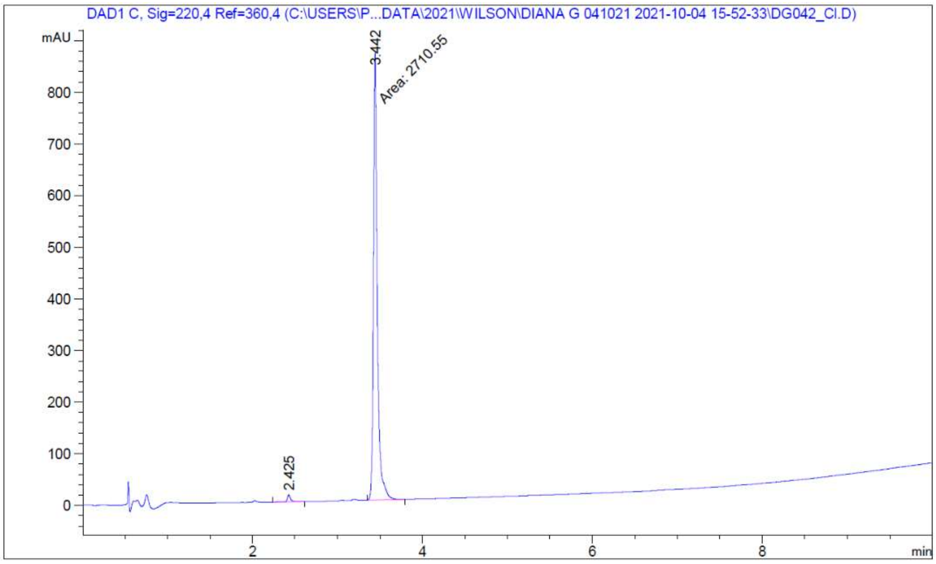

Analytical HPLC trace at λ= 220 nm of purified peptide **TACC3** $_{522-536\_(4-Cl)525F}$.

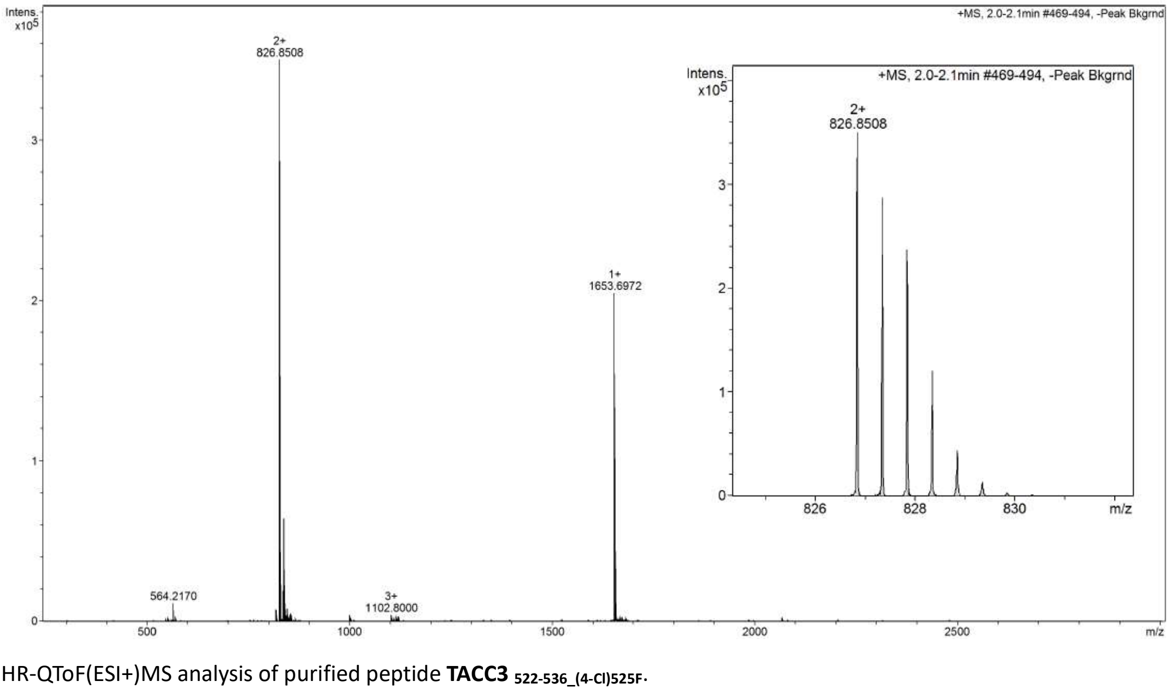

HR-QToF(ESI+)MS analysis of purified peptide **TACC3** $_{522-536\_(4-Cl)525F}$.

TACC3 $_{522\text{-}536\_(4\text{-}F)525F}$**:**

Sequence: Ac- EES(4F-F)RDPAEVLGTGA

HPLC (λ = 220 nm) tR = 3.190min. HR-QToF (ESI) m/z: [M+2H]2+ Calc. for $C_{69}H_{106}FN_{19}O_{26}$: 826.8843; Found: 818.8677.

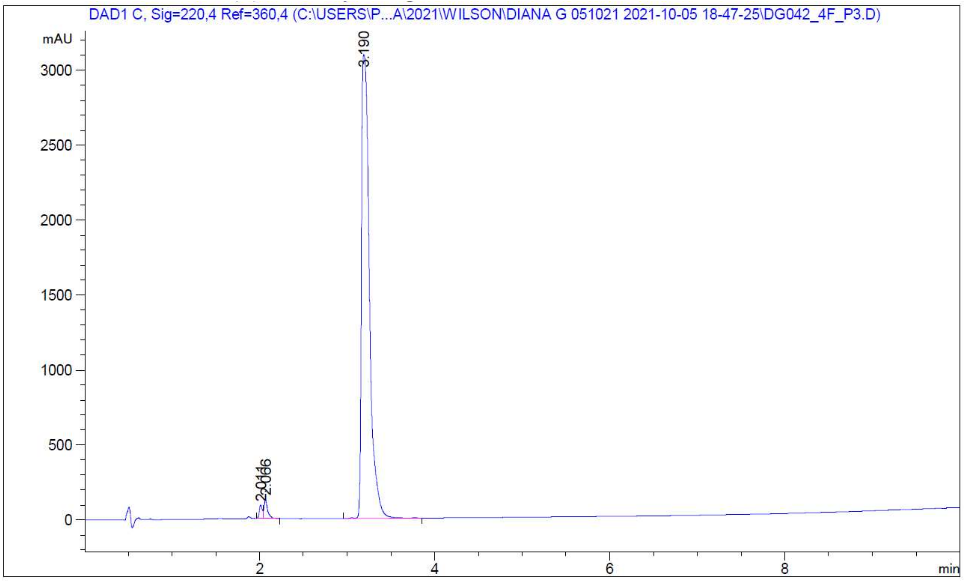

Analytical HPLC trace at λ= 220 nm of purified peptide **TACC3** $_{522\text{-}536\_(4\text{-}F)525F}$.

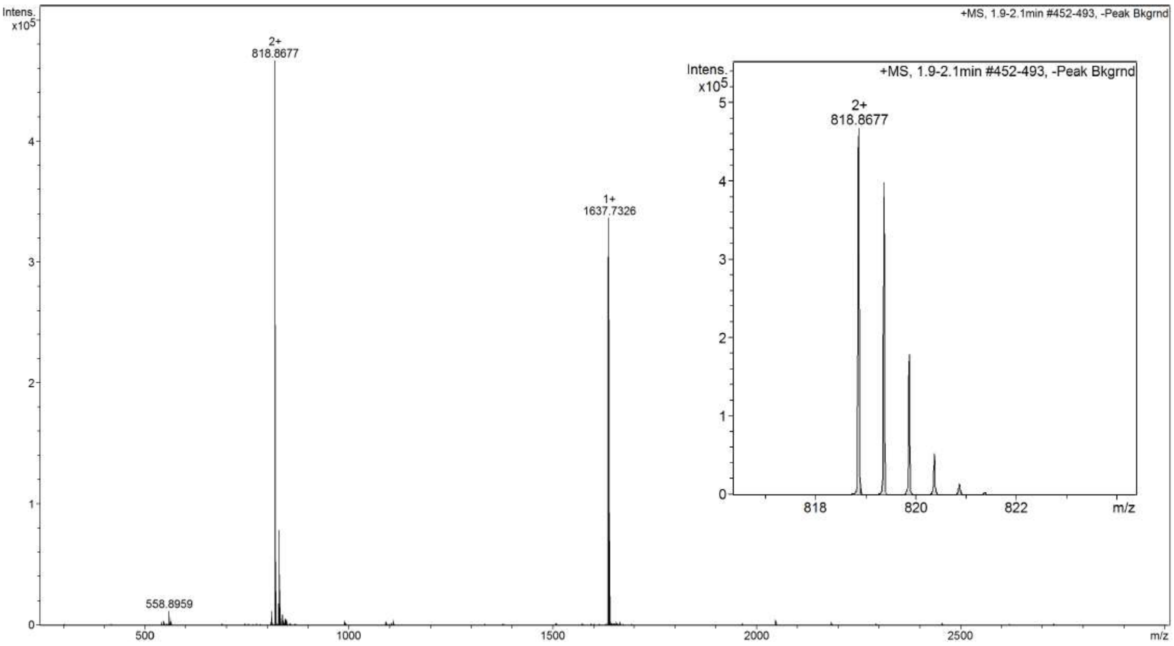

HR-QToF(ESI+)MS analysis of purified peptide **TACC3** $_{522\text{-}536\_(4\text{-}F)525F}$.

TACC3 $_{522\text{-}536\_(4\text{-}CF3)525F}$:

Sequence: Ac- EES(4CF_3-F)RDPAEVLGTGA

HPLC (λ = 220 nm) tR = 3.632min. HR-QToF (ESI) m/z: [M+2H]2+ Calc. for $C_{70}H_{106}F_3N_{19}O_{26}$: 826.8827; Found: 843.8766.

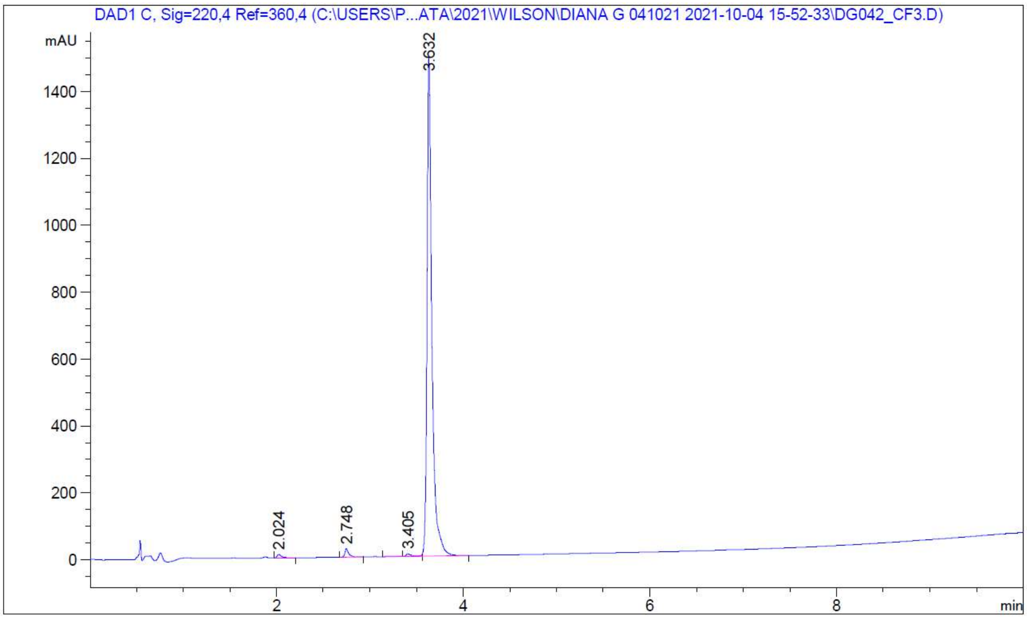

Analytical HPLC trace at λ= 220 nm of purified peptide **TACC3 $_{522\text{-}536\_(4\text{-}CF3)525F}$.**

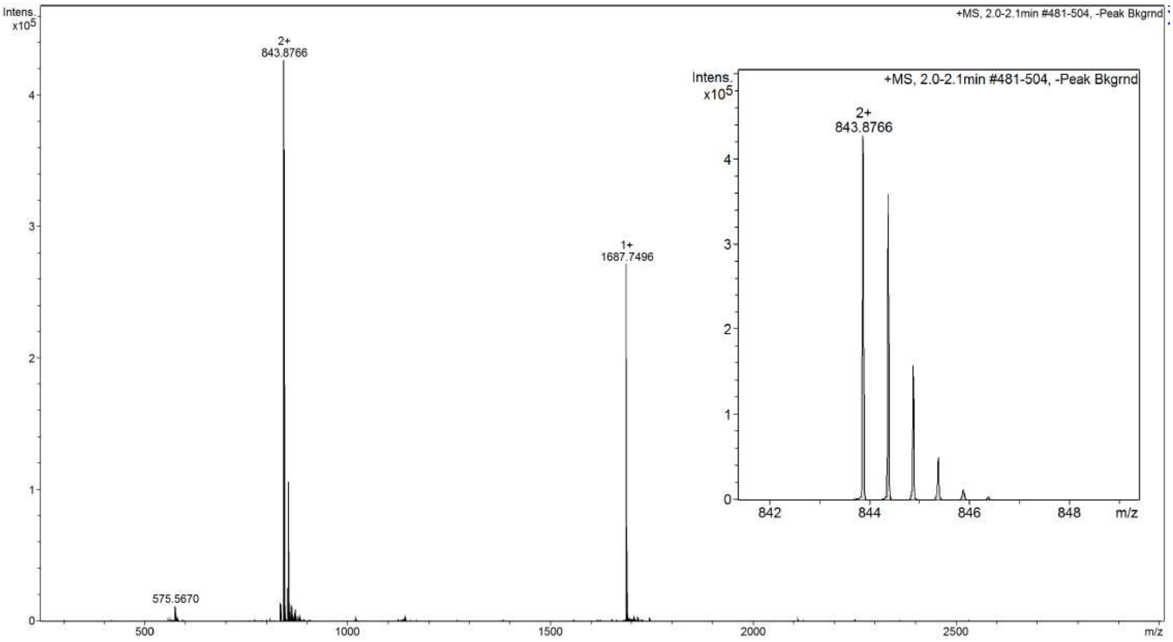

HR-QToF(ESI+)MS analysis of purified peptide **TACC3 $_{522\text{-}536\_(4\text{-}CF3)525F}$.**

TACC3 $_{522\text{-}536\_5f\text{-}525F}$:

Sequence: Ac- EES(4-5*f*Phe)RDPAEVLGTGA

HPLC (λ = 220 nm) tR = 3.601min. HR-QToF (ESI) m/z: [M+2H]2+ Calc. for $C_{69}H_{102}F_5N_{19}O_{26}$: 855.3666; Found: 855.3508.

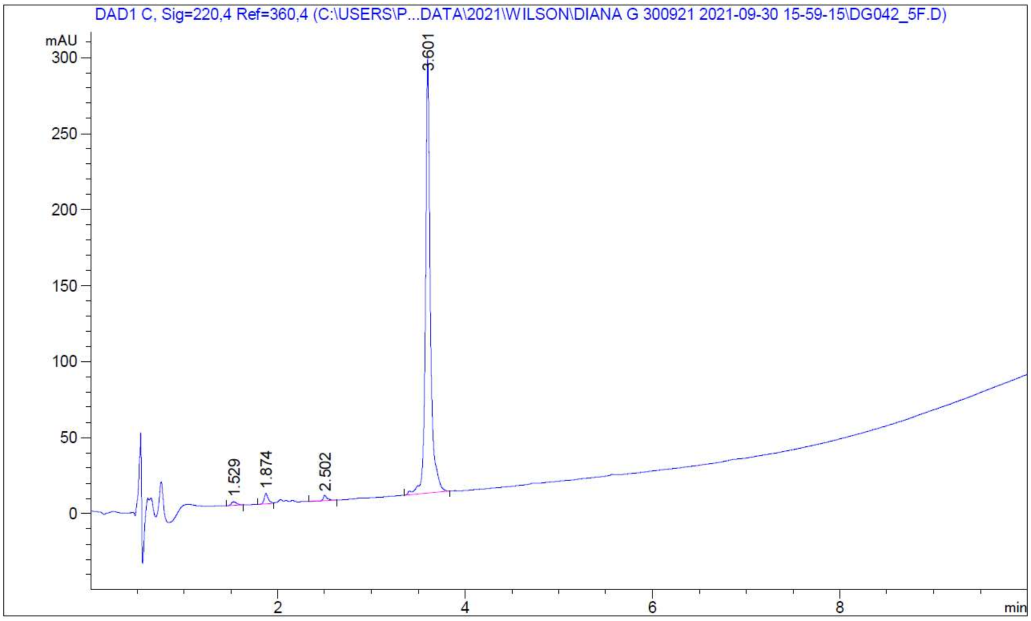

Analytical HPLC trace at λ= 220 nm of purified peptide **TACC3 $_{522\text{-}536\_5f\text{-}525F}$**

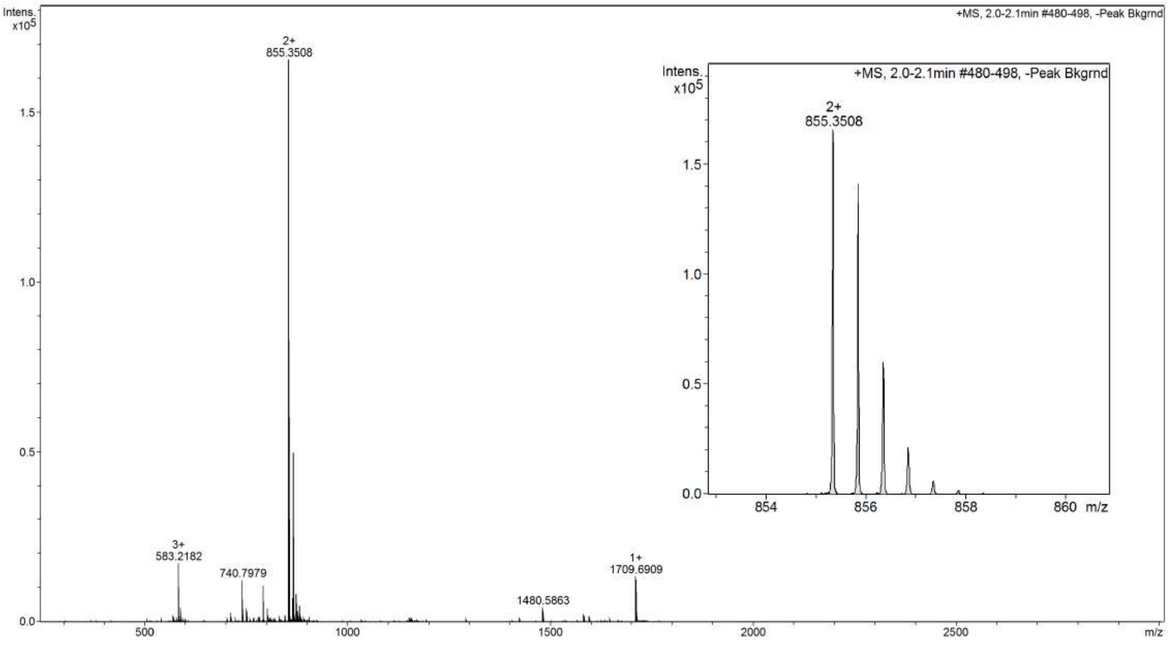

HR-QToF(ESI+)MS analysis of purified peptide **TACC3 $_{522\text{-}536\_5f\text{-}525F}$**

TACC3 $_{\text{522-536\_(}p\text{Bnz) -525F}}$

Sequence: Ac- EES(pBnz-Phe)RDPAEVLGTGA

HPLC (λ = 220 nm) tR = 3.740min. HR-QToF (ESI) m/z: [M+2H]2+ Calc. for $C_{76}H_{111}N_{19}O_{27}$: 862.4034; Found: 862.3792.

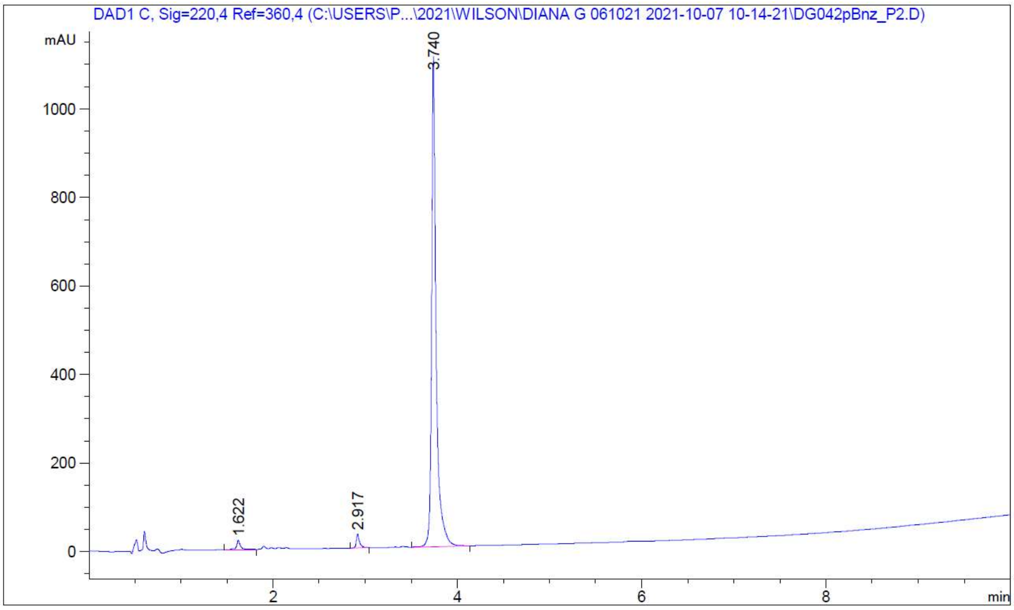

Analytical HPLC trace at λ= 220 nm of purified peptide **TACC3** $_{\text{522-536\_(}p\text{Bnz) -525F}}$

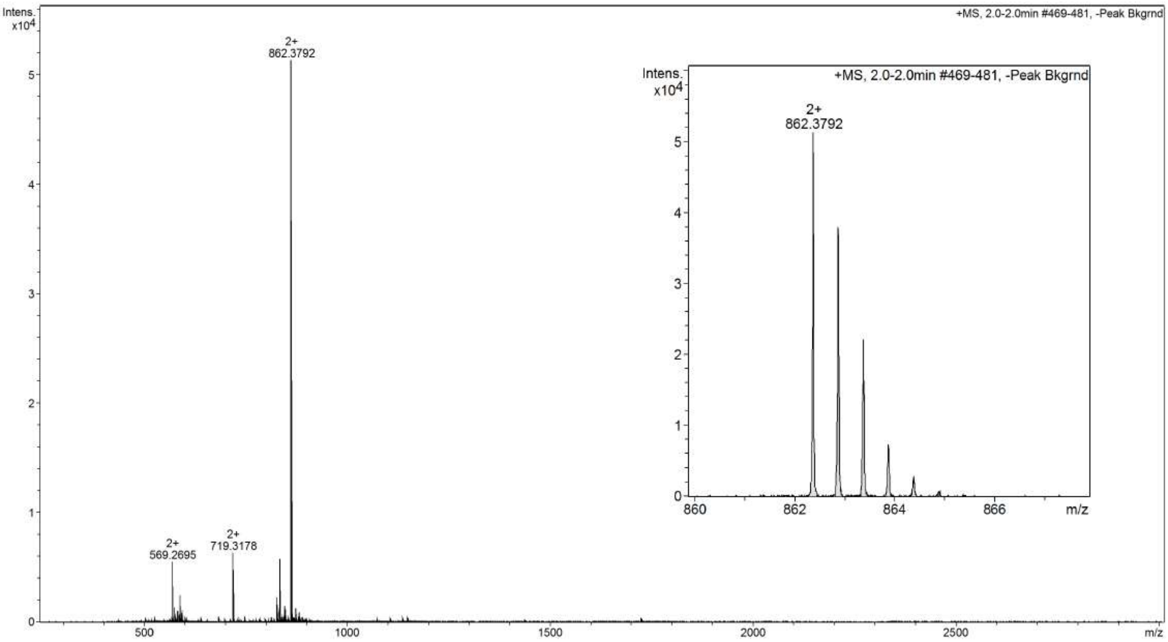

HR-QToF(ESI+)MS analysis of purified peptide **TACC3** $_{\text{522-536\_(}p\text{Bnz) -525F}}$

TACC3 $_{522\text{-}536\_(\alpha\text{-Me})\text{-}525F}$

Sequence: Ac- EES(αMe-Phe)RDPAEVLGTGA

HPLC (λ = 220 nm) tR = 3.394min. HR-QToF (ESI) m/z: [M+2H]2+ Calc. for $C_{70}H_{109}N_{19}O_{26}$: 816.8978; Found: 817.3840.

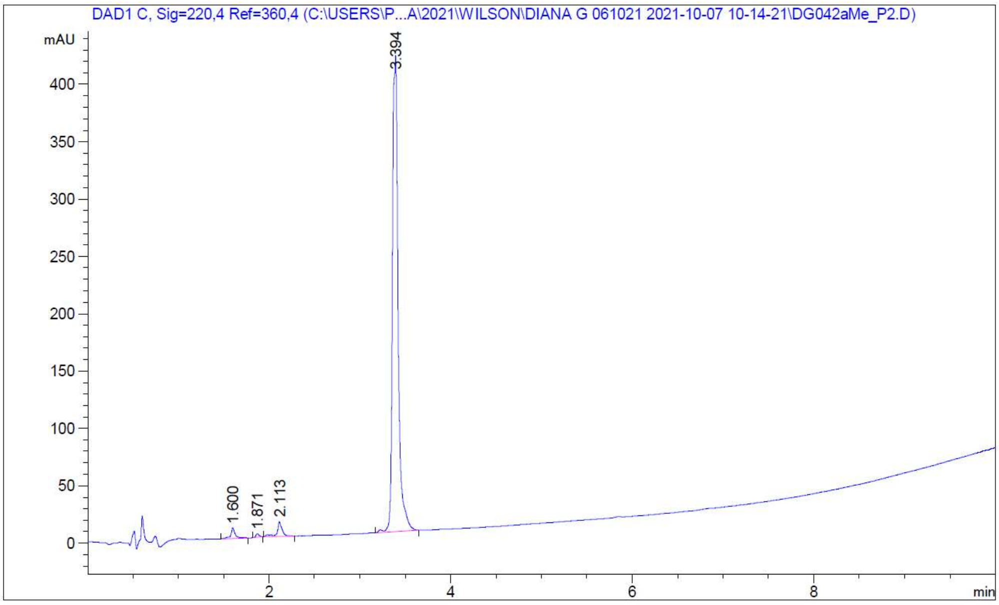

Analytical HPLC trace at λ= 220 nm of purified peptide **TACC3** $_{522\text{-}536\_(\alpha\text{-Me})\text{-}525F}$.

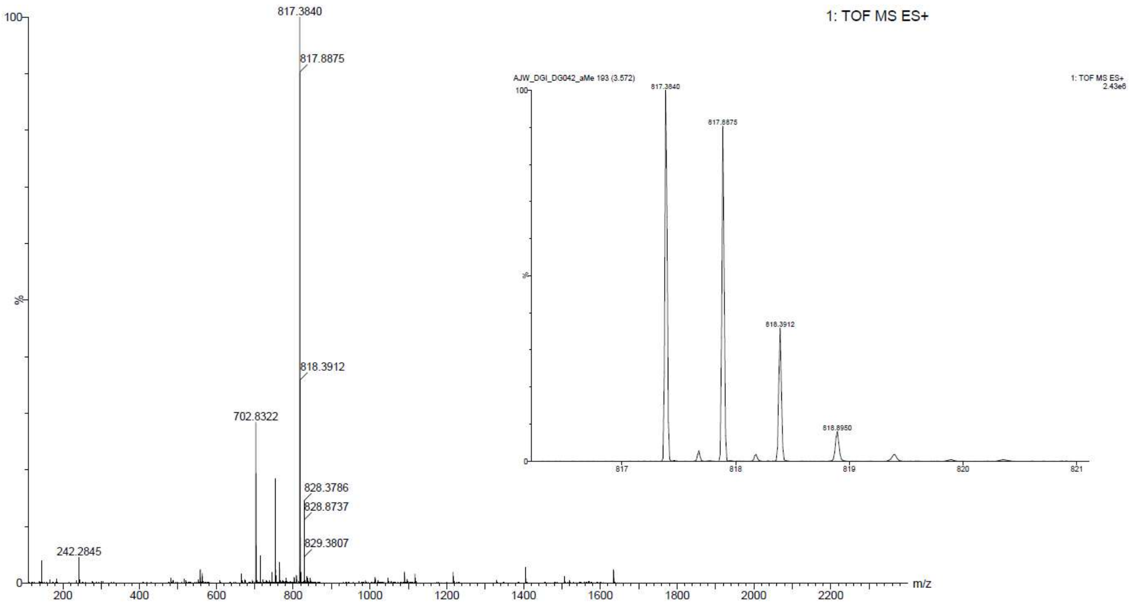

HR-QToF(ESI+)MS analysis of purified peptide **TACC3** $_{522\text{-}536\_(\alpha\text{-Me})\text{-}525F}$.

TACC3 $_{522\text{-}536\_(4\text{-}CN)\ \text{-}525F}$

Sequence: Ac- EES(4-CN-Phe)RDPAEVLGTGA

HPLC (λ = 220 nm) tR = 3.394min. HR-QToF (ESI) m/z: [M +2H]2+ Calc. for $C_{70}H_{106}N_{20}O_{26}$: 822.8878; Found: 822.8667.

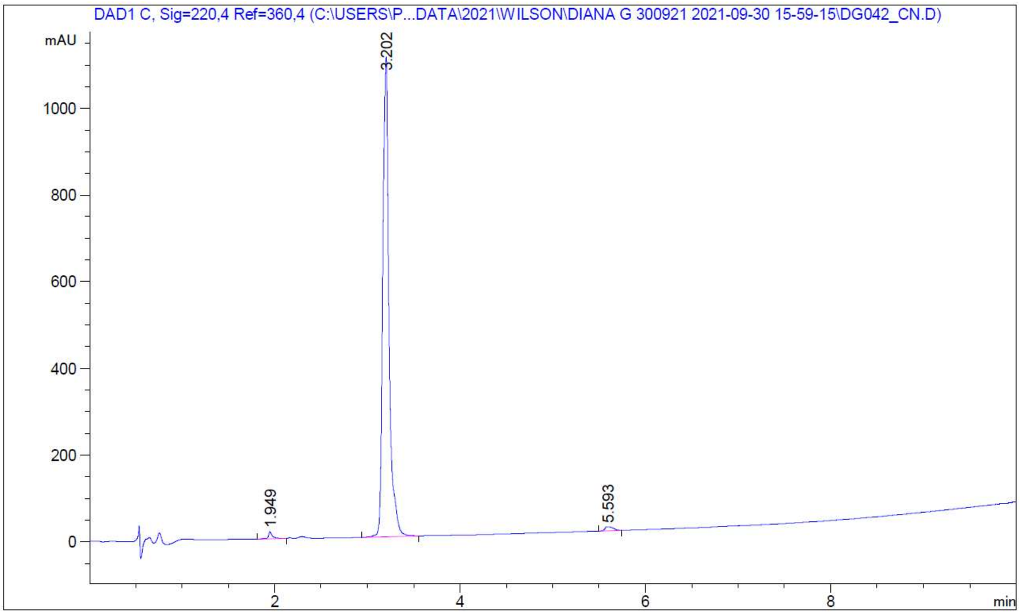

Analytical HPLC trace at λ= 220 nm of purified peptide **TACC3** $_{522\text{-}536\_(4\text{-}CN)\ \text{-}525F}$

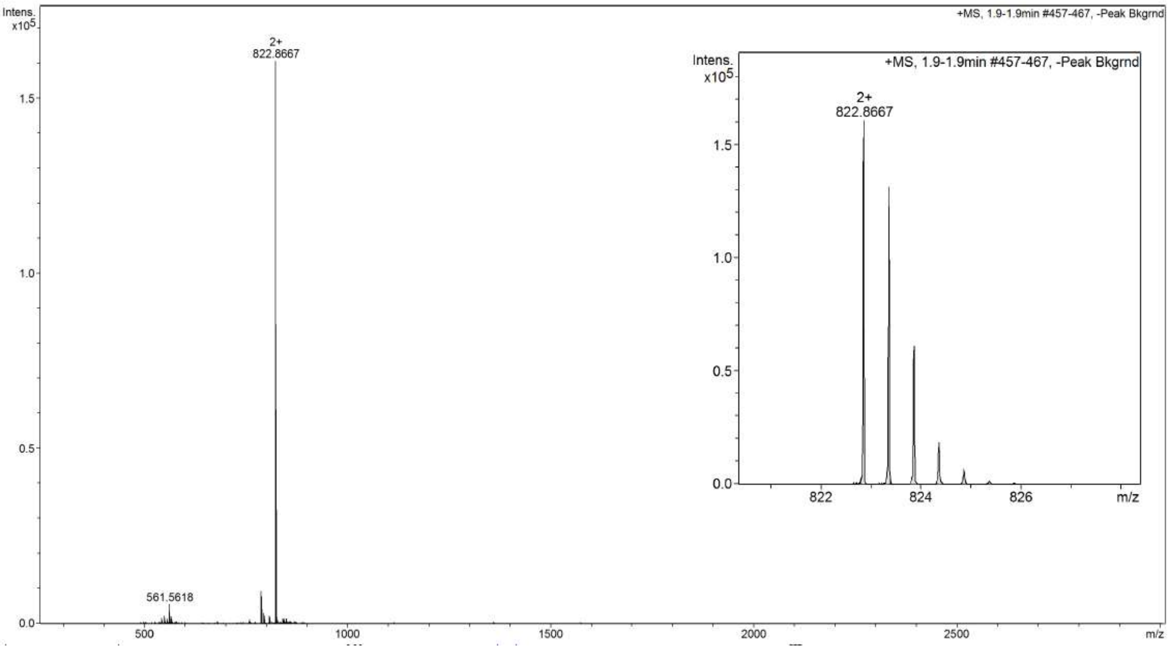

HR-QToF(ESI+)MS analysis of purified peptide **TACC3** $_{522\text{-}536\_(4\text{-}CN)\ \text{-}525F}$

TACC3 522-536_F525Y

Sequence: Ac- EES(Y)RDPAEVLGTGA

HPLC (λ = 220 nm) tR = 2.937min. HR-QToF (ESI) m/z: [M +2H]2+ Calc. for $C_{69}H_{107}N_{19}O_{27}$: 818.3586; Found: 818.3718.

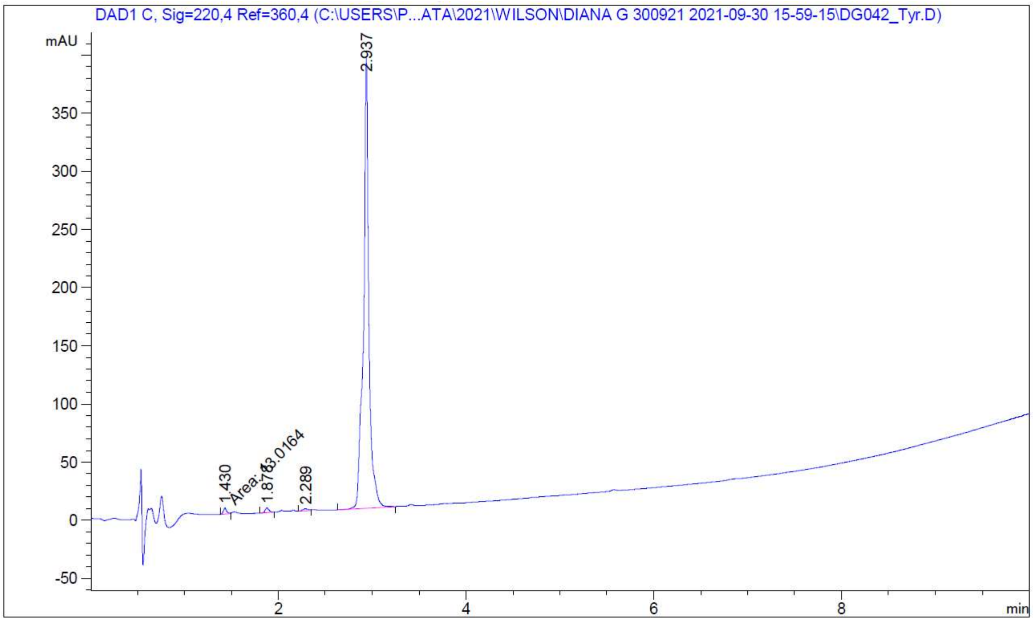

Analytical HPLC trace at λ= 220 nm of purified peptide **TACC3 522-536_F525Y**

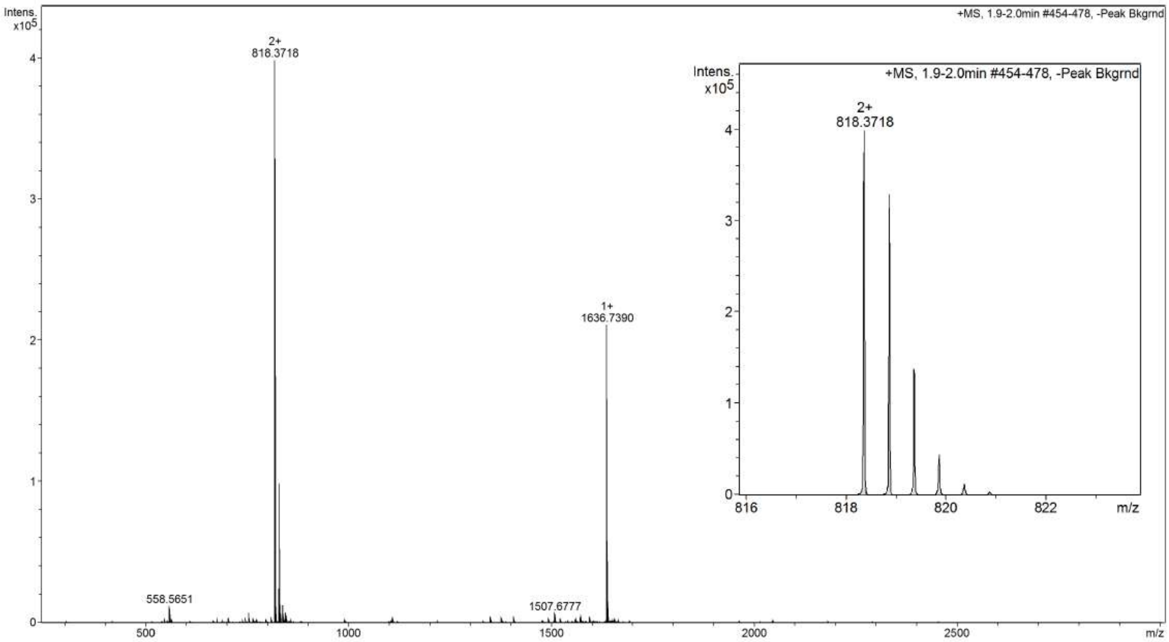

HR-QToF(ESI+)MS analysis of purified peptide **TACC3 522-536_F525Y**

TACC3 522-536_F525Y(OMe)

Sequence: Ac- EES(Y(OMe))RDPAEVLGTGA

HPLC (λ = 220 nm) tR = 2.937min. HR-QToF (ESI) m/z: [M +2H]2+ Calc. for $C_{70}H_{109}N_{19}O_{27}$: 824.8943; Found: 824.8848.

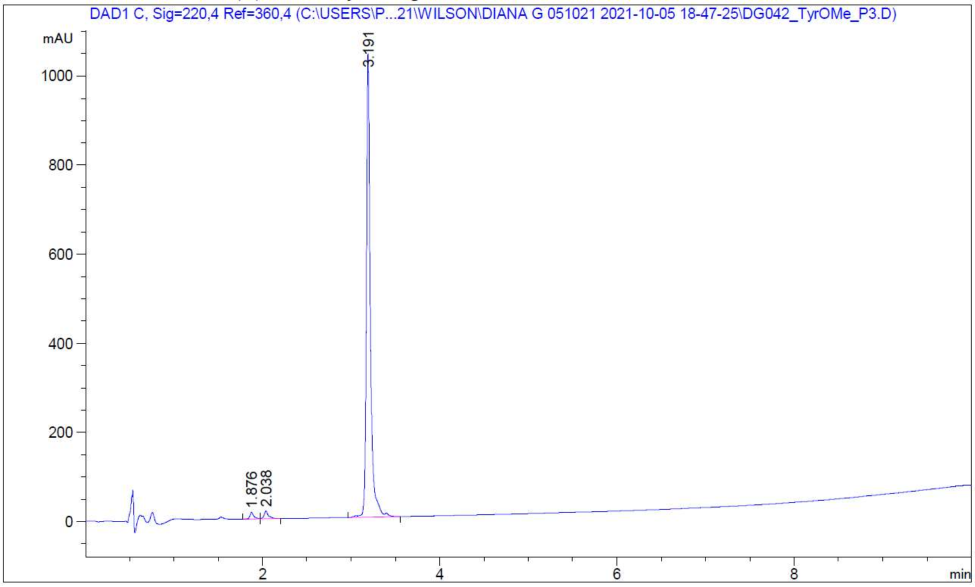

Analytical HPLC trace at λ= 220 nm of purified peptide **TACC3 522-536_F525Y(OMe)**.

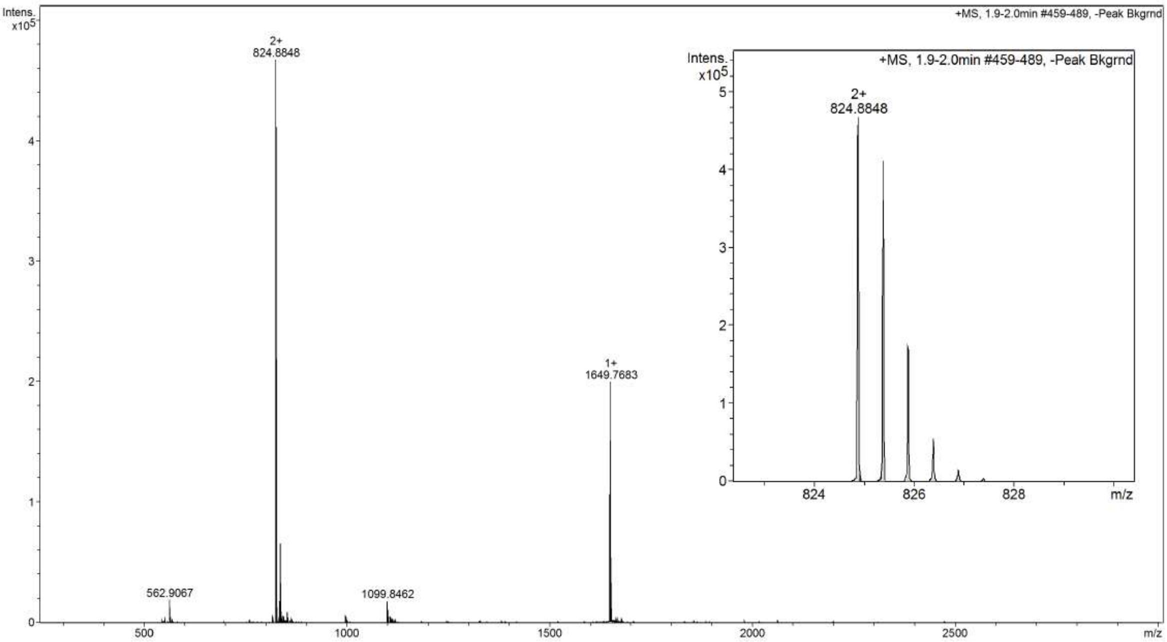

HR-QToF(ESI+)MS analysis of purified peptide **TACC3 522-536_F525Y(OMe)**

TACC3 $_{522\text{-}536\_cis\text{-}(4\text{-}F)528P}$

Sequence: Ac- EESFRD(*cis*-4F-P)AEVLGTGA

HPLC (λ = 220 nm) tR = 3.129 min. HR-QToF (ESI) m/z: [M+2H]2+ Calc. for $C_{69}H_{106}FN_{19}O_{26}$: 818.8843; Found: 818.8856.

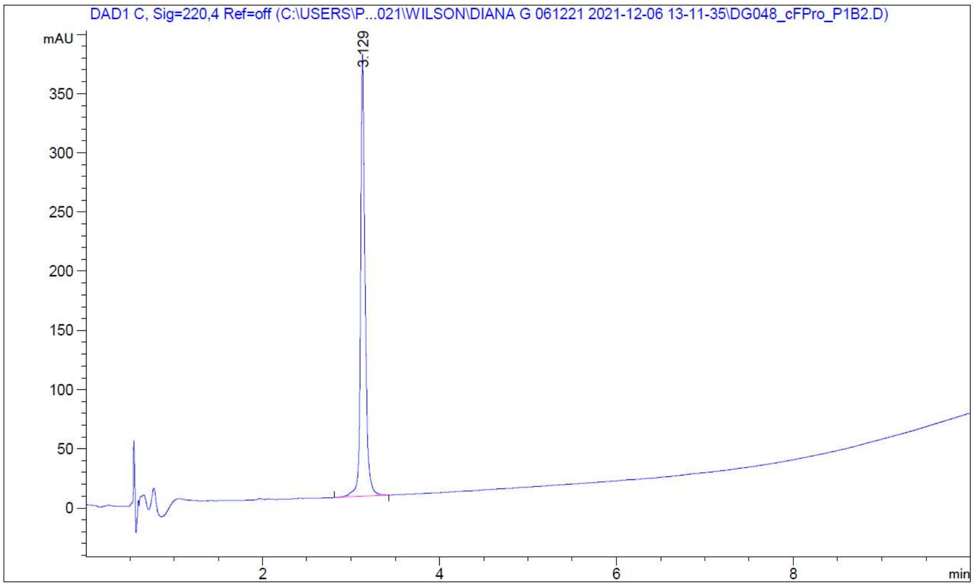

Analytical HPLC trace at λ= 220 nm of purified peptide **TACC3 $_{522\text{-}536\_cis\text{-}(4\text{-}F)528P}$**

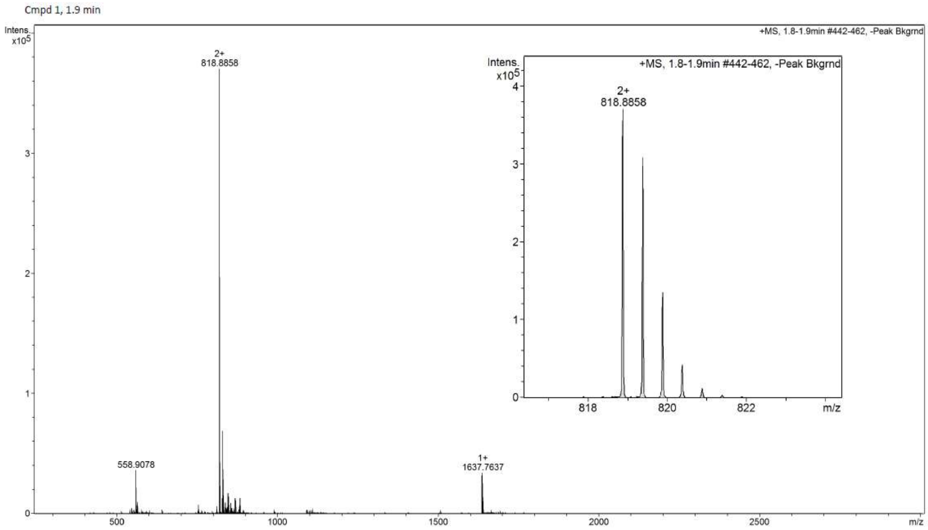

HR-QToF(ESI+)MS analysis of purified peptide **TACC3 $_{522\text{-}536\_cis\text{-}(4\text{-}F)528P}$**

TACC3 $_{522\text{-}536\_trans\text{-}(4\text{-}F)528P}$

Sequence: Ac- EESFRD(*trans*-4F-P)AEVLGTGA

HPLC (λ = 220 nm) tR = 3.106 min. HR-QToF (ESI) m/z: [M+2H]2+ Calc. for $C_{69}H_{106}FN_{19}O_{26}$: 818.8843; Found: 818.8860.

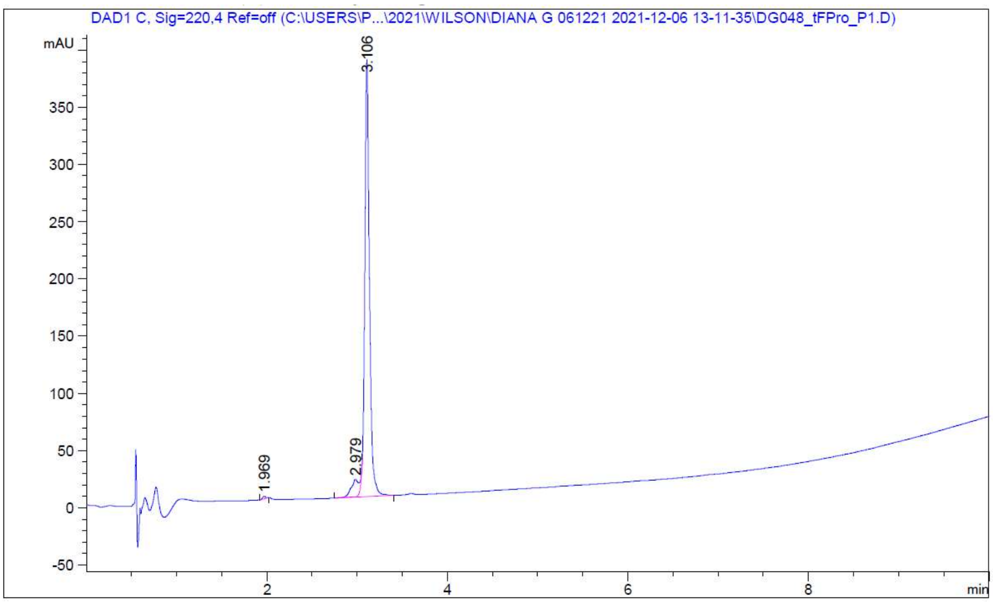

Analytical HPLC trace at λ= 220 nm of purified peptide **TACC3 $_{522\text{-}536\_trans\text{-}(4\text{-}F)528P}$**

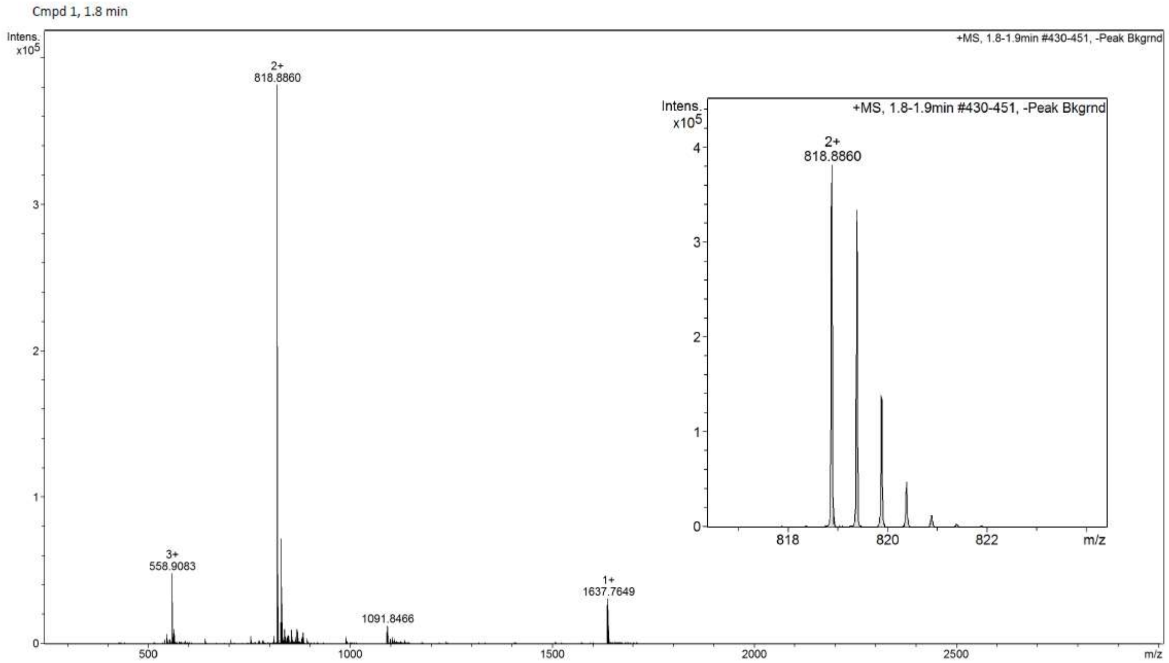

HR-QToF(ESI+)MS analysis of purified peptide **TACC3 $_{522\text{-}536\_trans\text{-}(4\text{-}F)528P}$**

TACCC3 $_{522\text{-}536\_D527G/A529G/E530G}$

Sequence: Ac- EESFRGPGGVLGTGA

HPLC (λ = 220 nm) tR = 2.949 min. HR-QToF (ESI) m/z: [M+2H]2+ Calc. for $\mathbf{C_{63}H_{99}N_{19}O_{22}}$: 737.8679; Found: 737.8699.

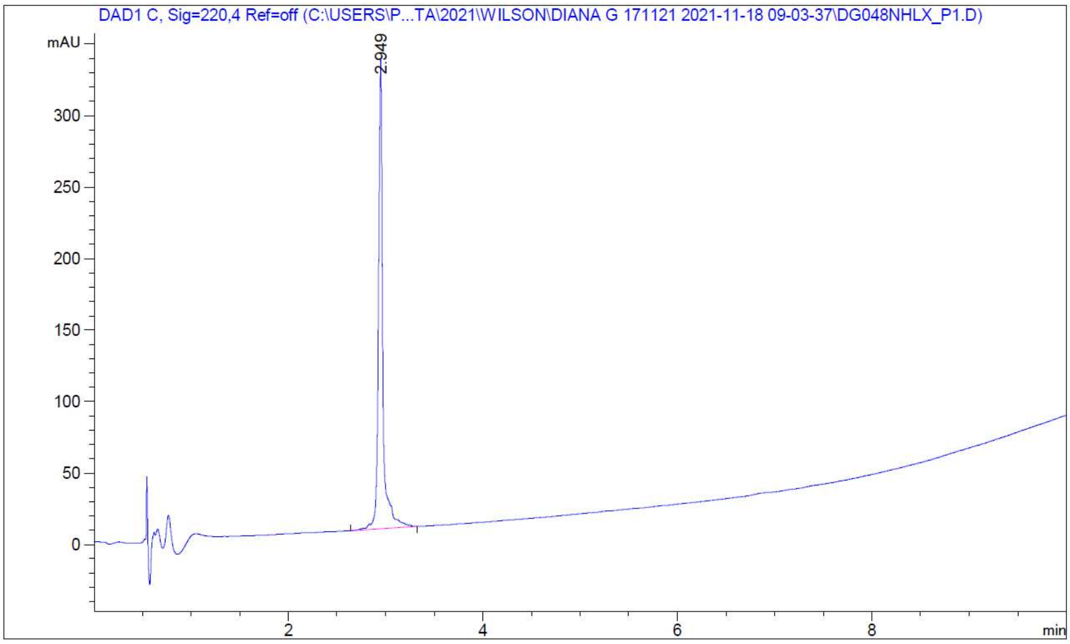

Analytical HPLC trace at λ= 220 nm of purified peptide **TACC3** $_{522\text{-}536\_D527G/A529G/E530G}$

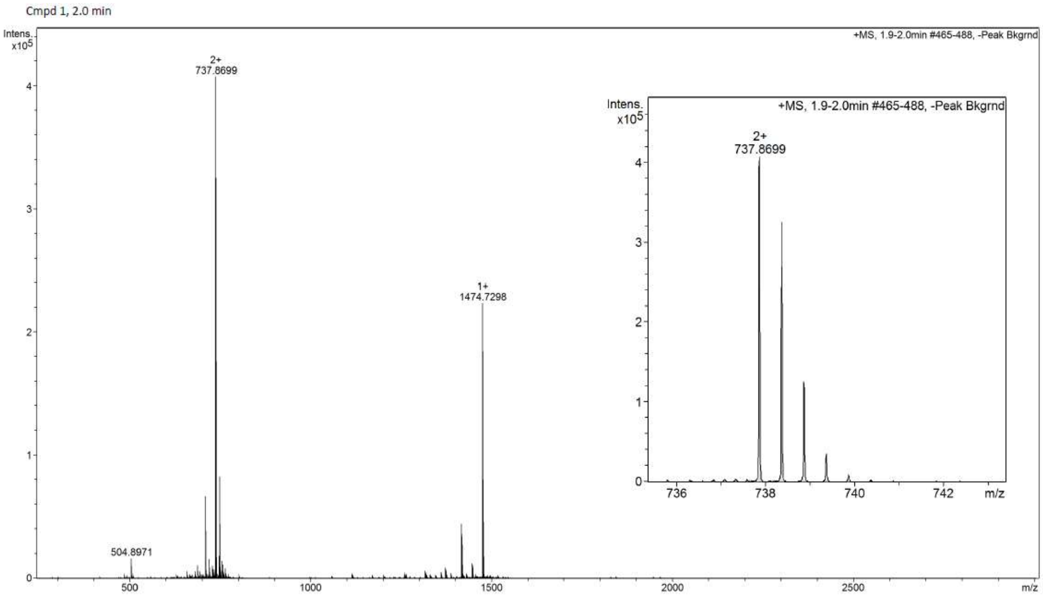

HR-QToF(ESI+)MS analysis of purified peptide **TACC3** $_{522\text{-}536\_D527G/A529G/E530G}$

TACCC3 $_{522\text{-}536\_V531I}$

Sequence: Ac- EESFRDPAEILGTGA

HPLC (λ = 220 nm) tR = 3.380 min. HR-QToF (ESI) m/z: [M+2H]2+ Calc. for $\mathbf{C_{70}H_{109}N_{19}O_{26}}$: 816.8968; Found: 816.8998.

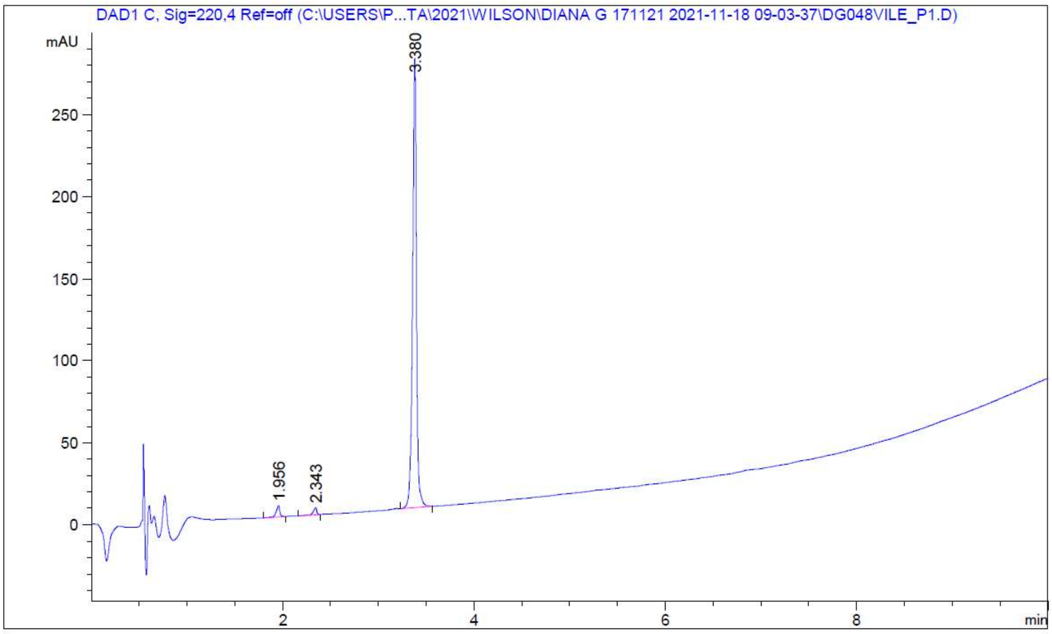

Analytical HPLC trace at λ= 220 nm of purified peptide **TACC3** $_{522\text{-}536\_V531I}$

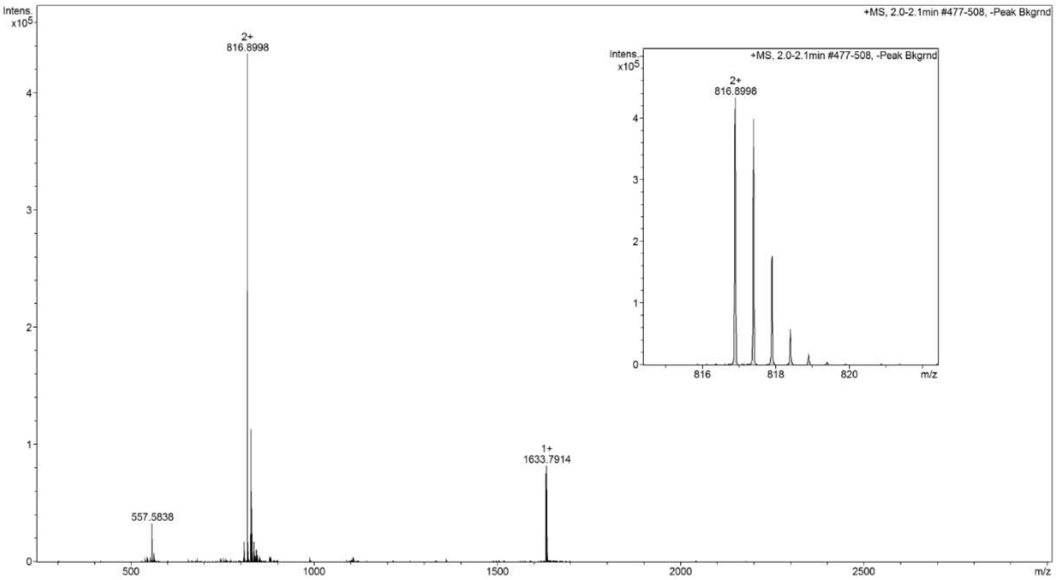

HR-QToF(ESI+)MS analysis of purified peptide **TACC3** $_{522\text{-}536\_V531I}$

TACC3 $_{522\text{-}536\_V531nL}$

Sequence: Ac- EESFRDPAE(nL)LGTGA

HPLC (λ = 220 nm) tR = 3.442 min. HR-QToF (ESI) m/z: [M+2H]2+ Calc. for $\mathbf{C_{70}H_{109}N_{19}O_{26}}$: 816.8968; Found: 816.8988.

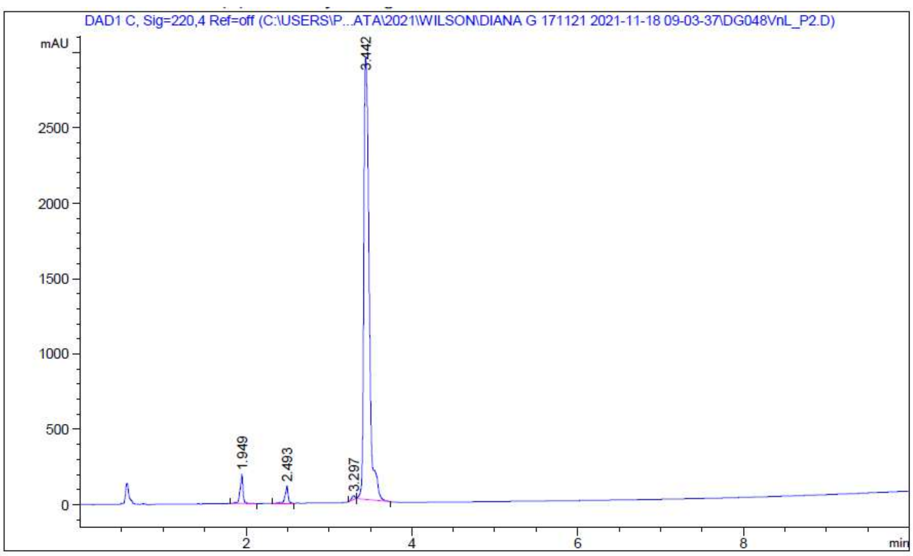

Analytical HPLC trace at λ= 220 nm of purified peptide **TACC3 $_{522\text{-}536\_V531nL}$**

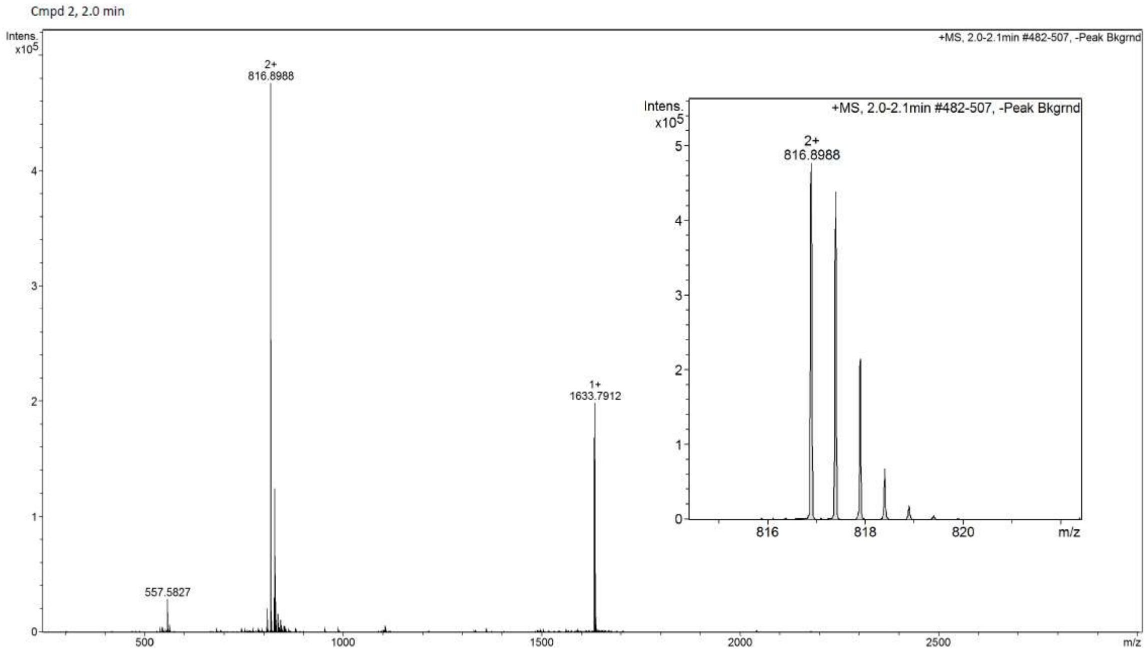

HR-QToF(ESI+)MS analysis of purified peptide **TACC3 $_{522\text{-}536\_V531nL}$**

TACC3 $_{522\text{-}536\_L532nL}$

Sequence: Ac- EESFRDPAEV(nL)GTGA

HPLC (λ = 220 nm) tR = 3.202 min. HR-QToF (ESI) m/z: [M+2H]2+ Calc. for $\mathbf{C_{69}H_{107}N_{19}O_{26}}$: 809.8890; Found: 809.8912.

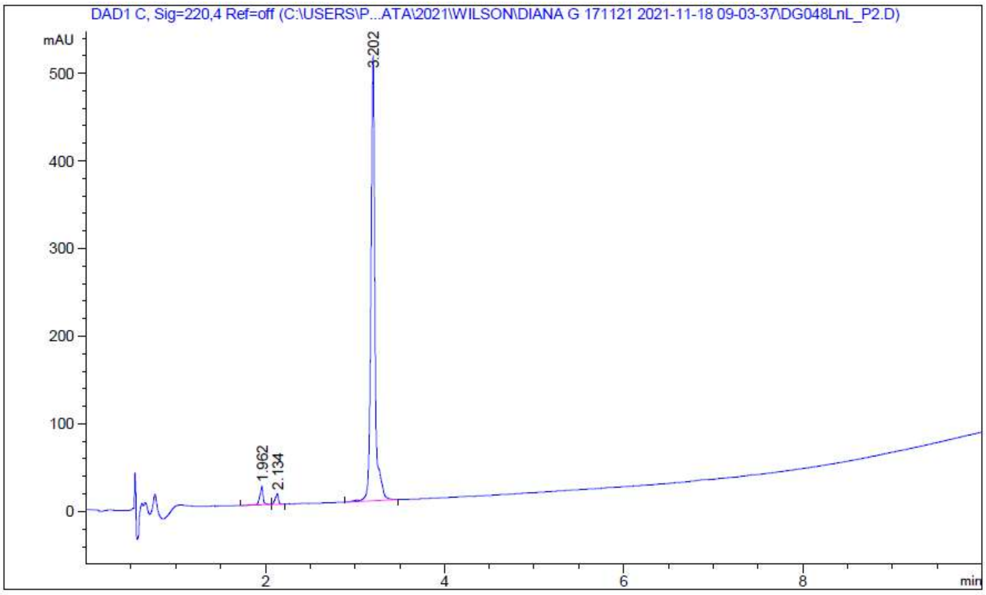

Analytical HPLC trace at λ= 220 nm of purified peptide **TACC3** $_{522\text{-}536\_L532nL}$

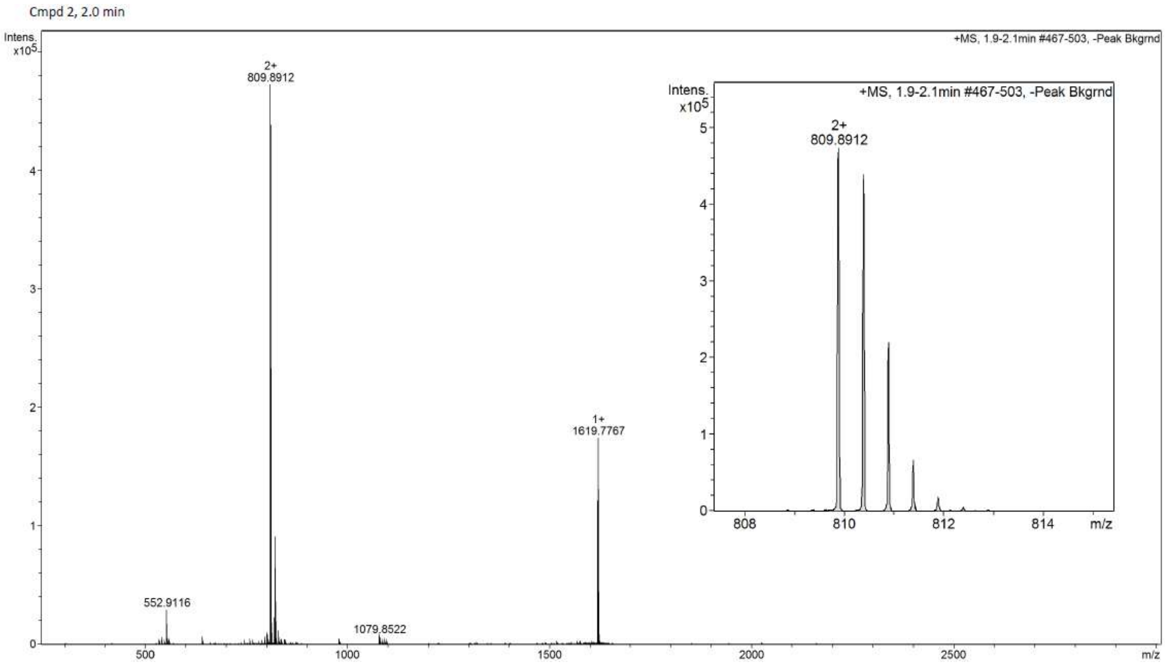

HR-QToF(ESI+)MS analysis of purified peptide **TACC3** $_{522\text{-}536\_L532nL}$

TACC3 $_{522\text{-}563}$ Ahx-FAM

Sequence: FAM-Ahx- EESFRDPAEVLGTGAEVDYLEQFGTSSFKESALRKQSLYLKF

HPLC (λ = 220 nm) tR = 4.680min. HR-QToF (ESI) m/z: [M+5H]5+ Calc. for $C_{242}H_{349}N_{55}O_{76}$: 1049.9099; Found: 1049.9150.

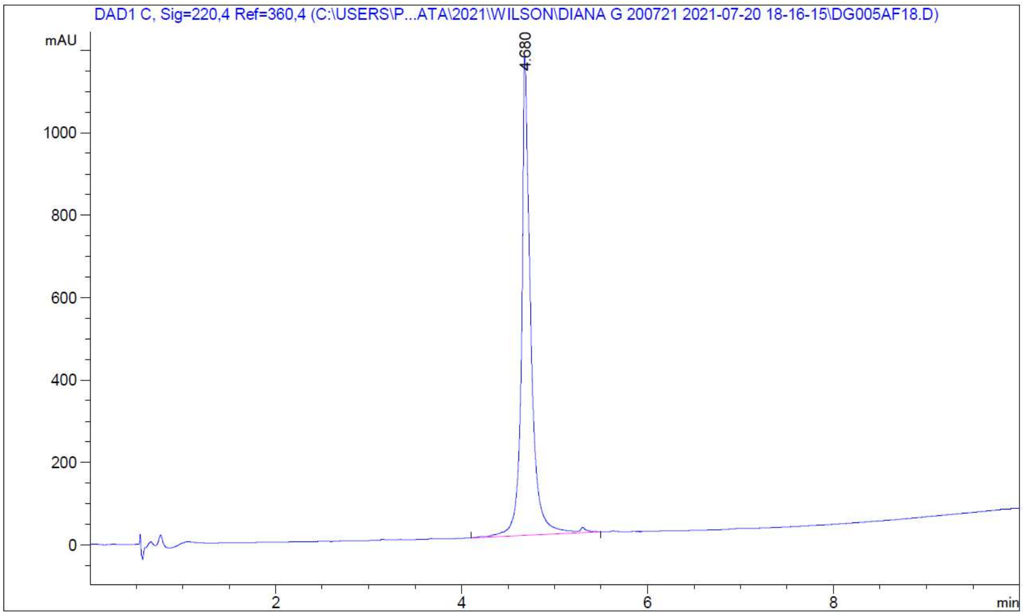

Analytical HPLC trace at λ= 220 nm of purified peptide **TACC3 $_{522\text{-}563}$ Ahx-FAM**

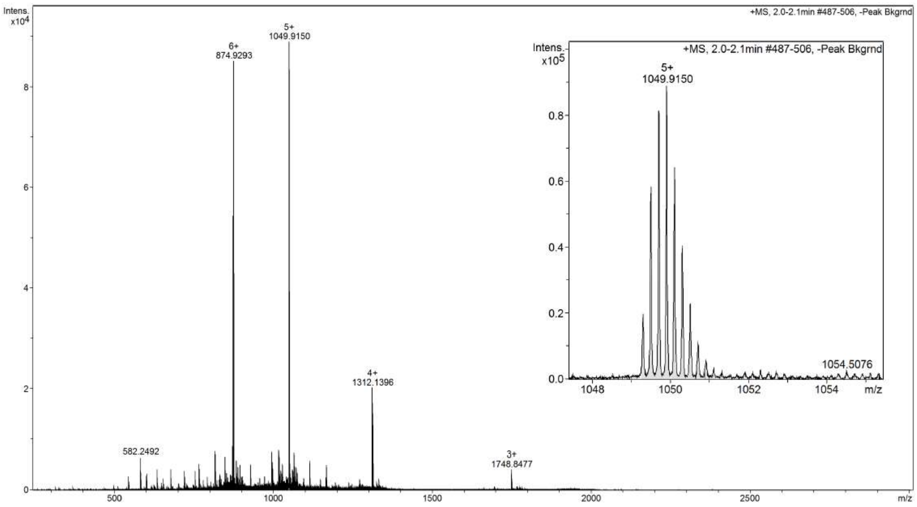

HR-QToF(ESI+)MS analysis of purified peptide **TACC3 $_{522\text{-}563}$ Ahx-FAM**.

TACC3 $_{522\text{-}552}$ Ahx-FAM

Sequence: FAM-Ahx- EESFRDPAEVLGTGAEVDYLEQFGTSSFKES

HPLC (λ = 220 nm) tR = 7.41min. HR-QToF (ESI) m/z: [M+3H]3+ Calc. for $C_{177}H_{244}N_{38}O_{62}$: 1299.5798; Found: 1299.5815.

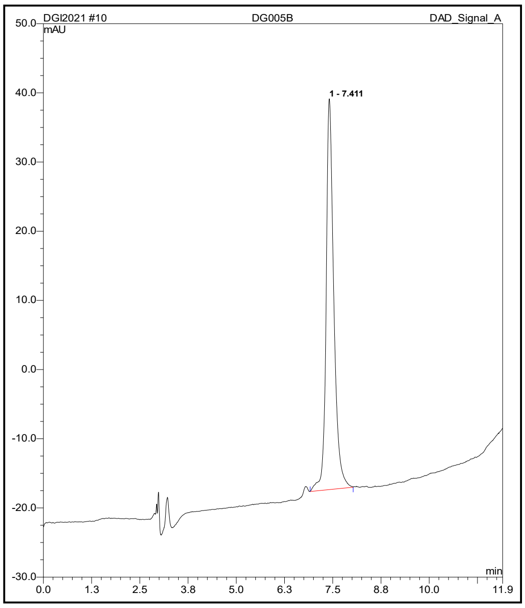

Analytical HPLC trace at λ= 220 nm of purified peptide **TACC3 $_{522\text{-}552}$ Ahx-FAM**

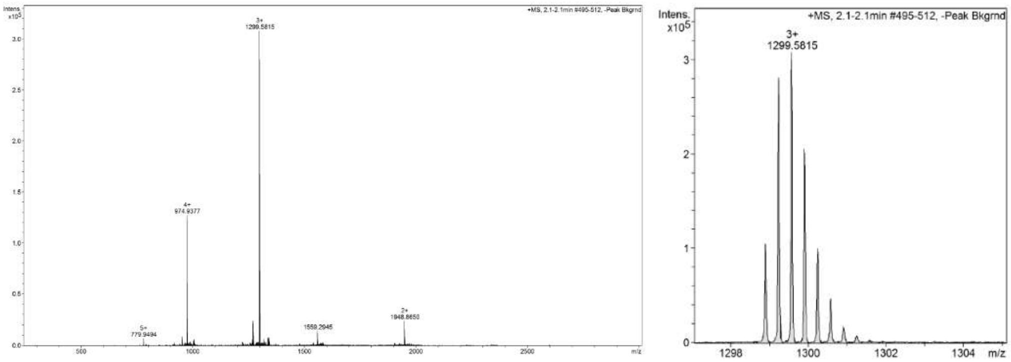

HR-QToF(ESI+)MS analysis of purified peptide **TACC3 $_{522\text{-}552}$ Ahx-FAM**

TACC3 $_{522\text{-}532}$ Ahx-FAM

Sequence: FAM-Ahx- EESFRDPAEVL

HPLC (λ = 220 nm) tR = 4.146min. HR-QToF (ESI) m/z: [M+2H]2+ Calc. for $C_{83}H_{108}N_{16}O_{27}$: 881.3858; Found: 881.3880.

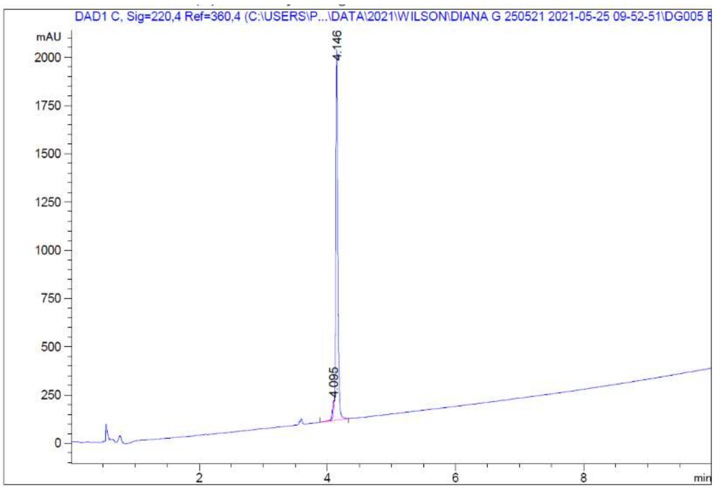

Analytical HPLC trace at λ= 220 nm of purified peptide **TACC3 $_{522\text{-}532}$ Ahx-FAM**

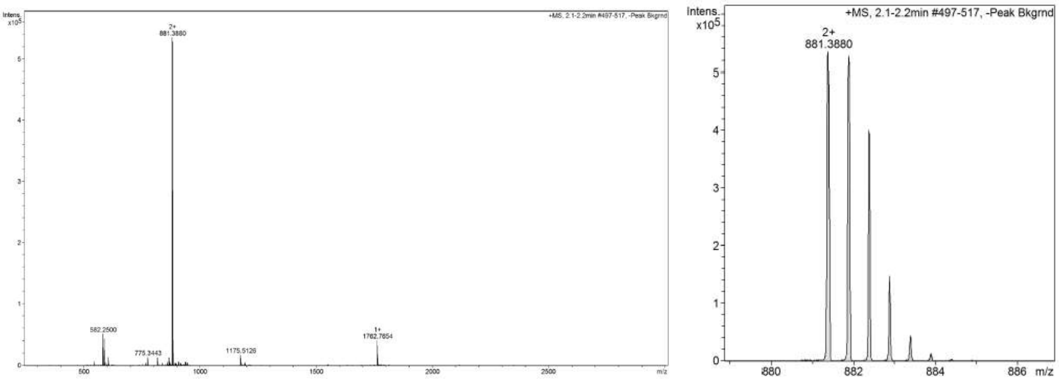

HR-QToF(ESI+)MS analysis of purified peptide **TACC3 $_{522\text{-}532}$ Ahx-FAM**

TACC3 $_{522\text{-}542}$ Ahx-FAM

Sequence: FAM-Ahx- EESFRDPAEVLGTGAEVDYLE

HPLC (λ = 220 nm) tR = 6.21 min. HR-QToF (ESI) m/z: [M+2H]2+ Calc. for $C_{128}H_{174}N_{26}O_{45}$: 1398.6136; Found: 1398.6136.

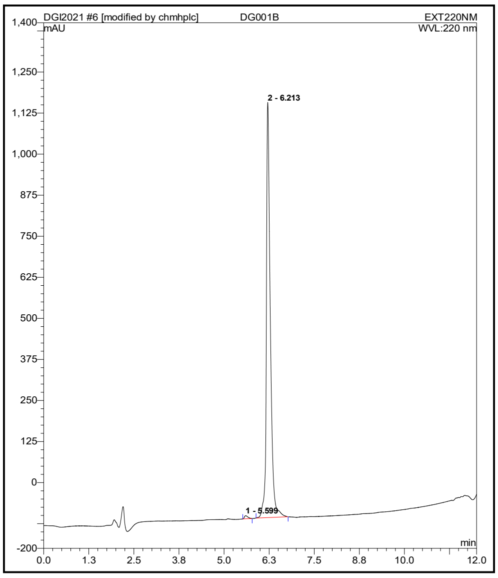

Analytical HPLC trace at λ= 220 nm of purified peptide **TACC3 $_{522\text{-}542}$ Ahx-FAM**

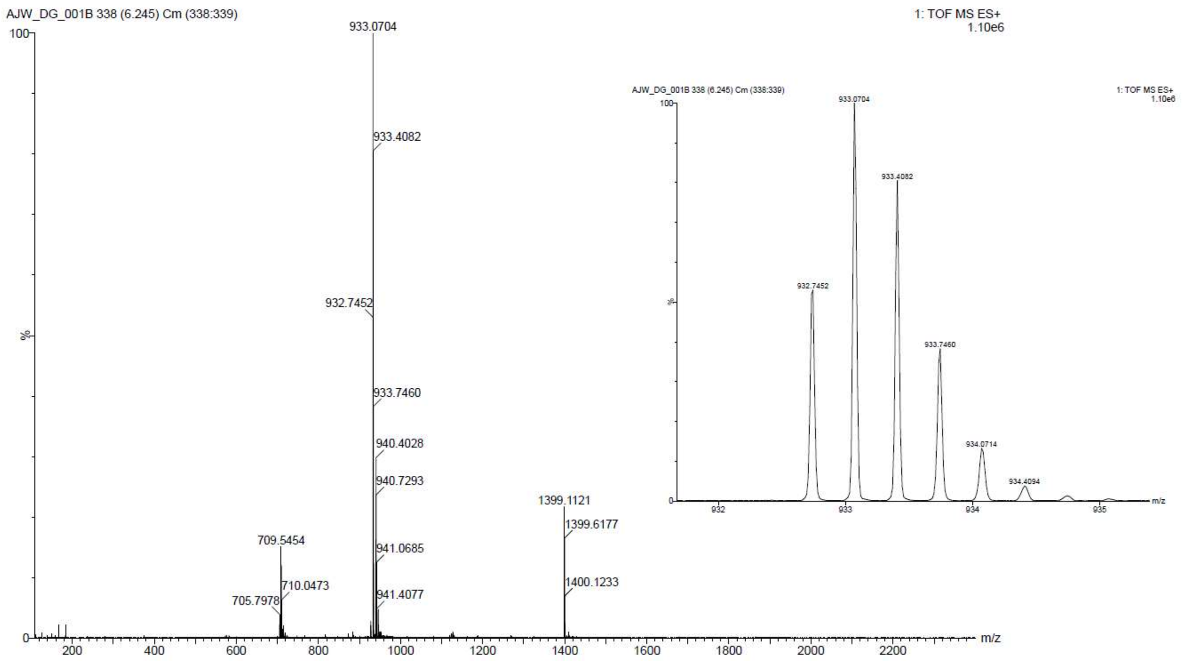

HR-QToF(ESI+)MS analysis of purified **TACC3 $_{522\text{-}542}$ Ahx-FAM**

TACC3 $_{518\text{-}532}$ Ahx-FAM

Sequence: FAM-Ahx- LELKEESFRDPAEVL

HPLC (λ = 220 nm) tR = 6.164 min. HR-QToF (ESI) m/z: [M+2H]2+ Calc. for $C_{106}H_{149}N_{21}O_{33}$: 1123.038; Found: 1123.0398.

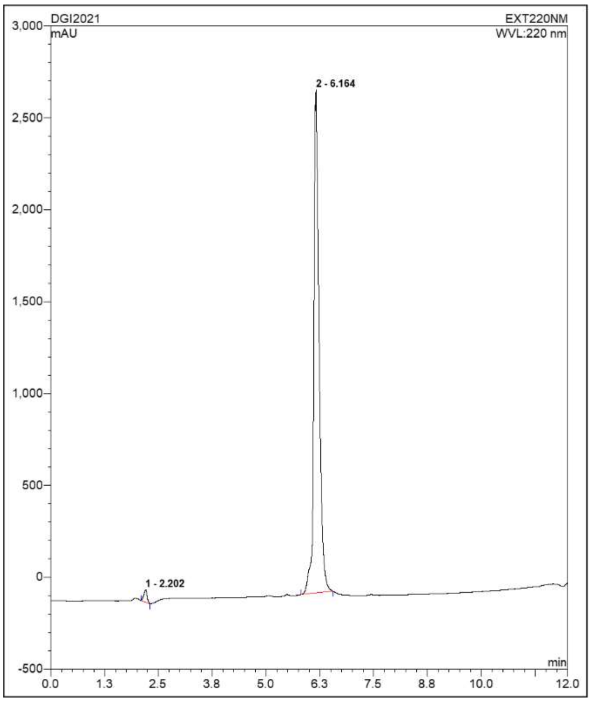

Analytical HPLC trace at λ= 220 nm of purified peptide **TACC3 $_{518\text{-}532}$ Ahx-FAM**

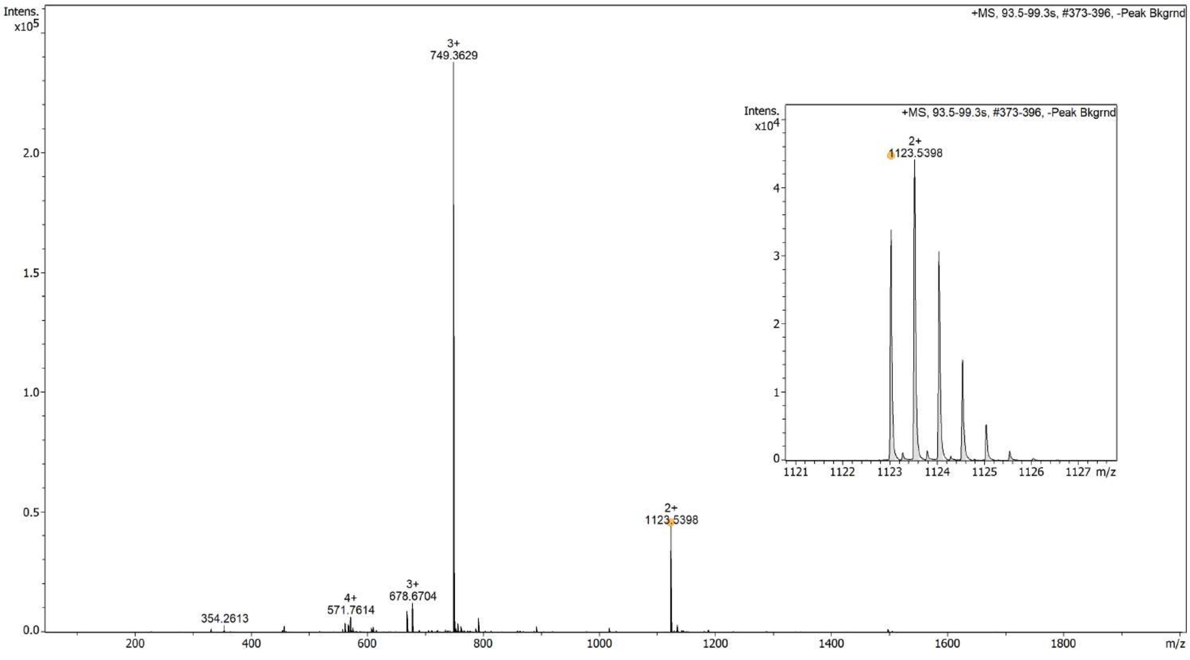

HR-QToF(ESI+)MS analysis of purified peptide **TACC3 $_{518\text{-}532}$ Ahx-FAM**

TACC3 $_{536\text{-}563}$ Ahx-FAM

Sequence: FAM-Ahx- AEVDYLEQFGTSSFKESALRKQSLYLKF

HPLC (λ = 220 nm) tR = 6.25min. HR-QToF (ESI) m/z: [M+5H]5+ Calc. for $C_{177}H_{244}N_{38}O_{62}$: 752.1735; Found: 752.1735.

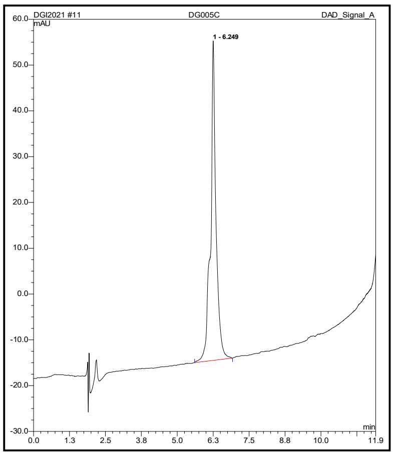

Analytical HPLC trace at λ= 220 nm of purified peptide **TACC3 $_{536\text{-}563}$ Ahx-FAM**

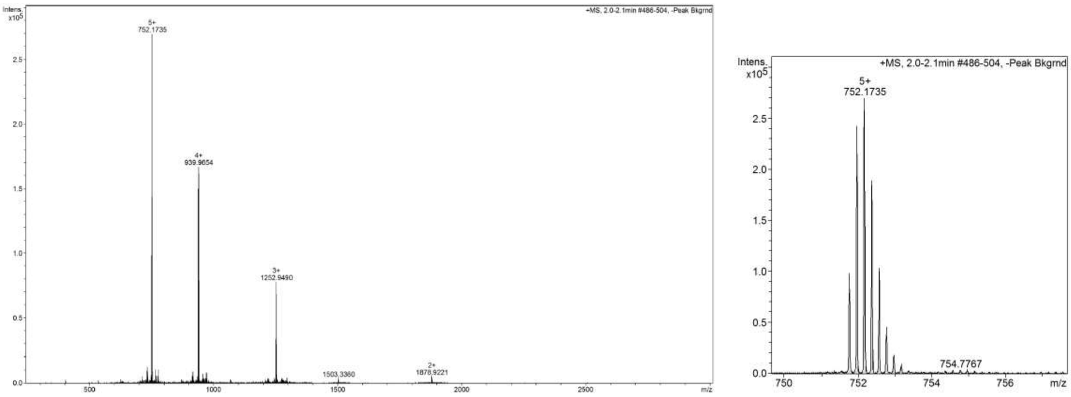

HR-QToF(ESI+)MS analysis of purified peptide **TACC3 $_{536\text{-}563}$ Ahx-FAM**

TACC3 $_{522\text{-}536\ E523C/R526C}$

Sequence: Ac- ECSFCDPAEVLGTGA

HPLC (λ = 220 nm) tR = 4.82min. HR-QToF (ESI) m/z: [M+H]+ Calc. for $C_{64}H_{98}N_{16}O_{24}S_2$: 1539.6454; Found:1539.6470.

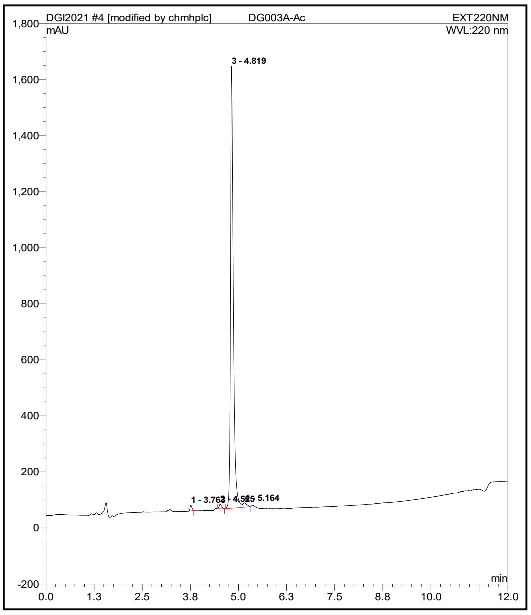

Analytical HPLC trace at λ= 220 nm of purified peptide **TACC3 $_{522\text{-}536\ E523C/R526C}$**

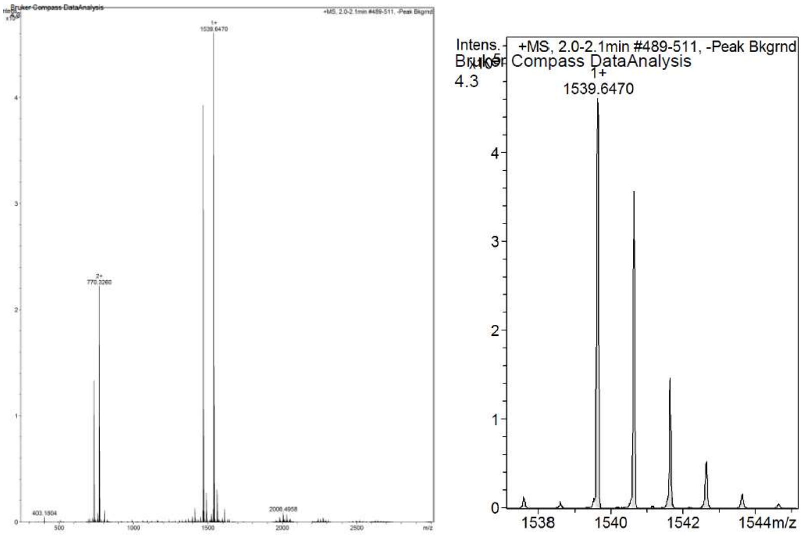

HR-QToF(ESI+)MS analysis of purified peptide **TACC3 $_{522\text{-}536\ E523C/R526C}$**

TACC3 $_{522\text{-}536\ S524C/D527C}$

Sequence: Ac- EECFRCPAEVLGTGA

HPLC (λ = 220 nm) tR = 6.72min. HR-QToF (ESI) m/z: [M+2H]2+ Calc. for $C_{68}H_{107}N_{19}O_{23}S_2$: 812.3729; Found:812.3729.

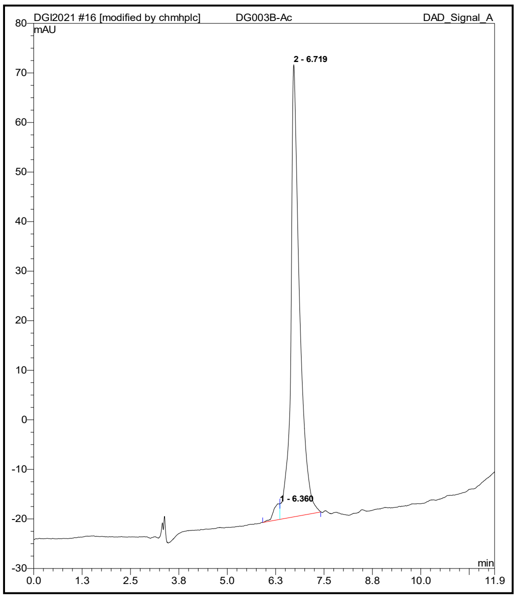

Analytical HPLC trace at λ= 220 nm of purified peptide **TACC3 $_{522\text{-}536\ S524C/D527C}$**

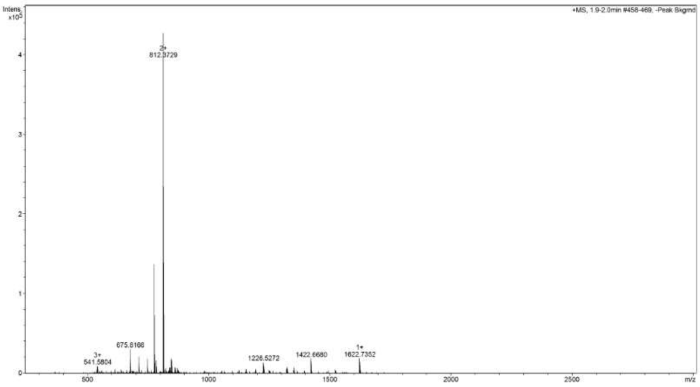

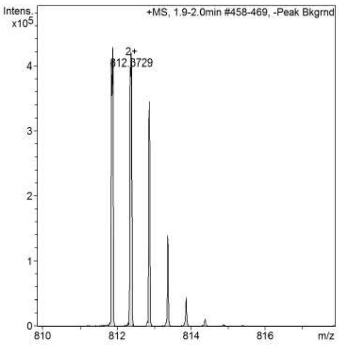

HR-QToF(ESI+)MS analysis of purified peptide **TACC3 $_{522\text{-}536\ S524C/D527C}$**

TACC3 $_{\mathbf{522\text{-}536\ F525C/P528C}}$

Sequence: Ac- EESCRDCAEVLGTGA

HPLC (λ = 220 nm) tR = 6.72min. HR-QToF (ESI) m/z: [M+2H]2+ Calc. for $C_{61}H_{101}N_{19}O_{26}S_2$: 790.8376; Found:790.8370.

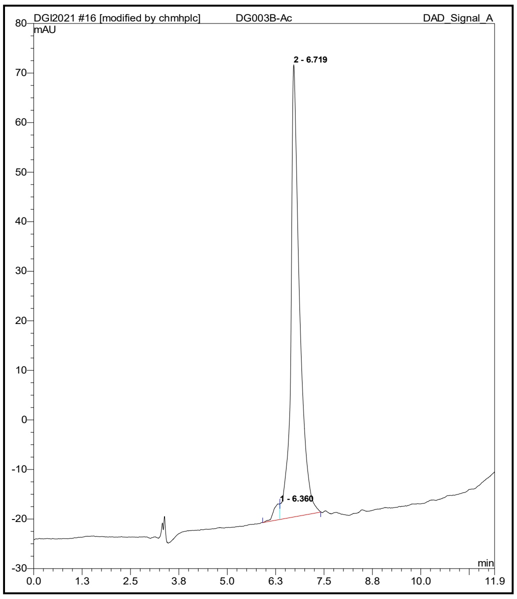

Analytical HPLC trace at λ= 220 nm of purified peptide **TACC3** $_{\mathbf{522\text{-}536\ F525C/P528C}}$

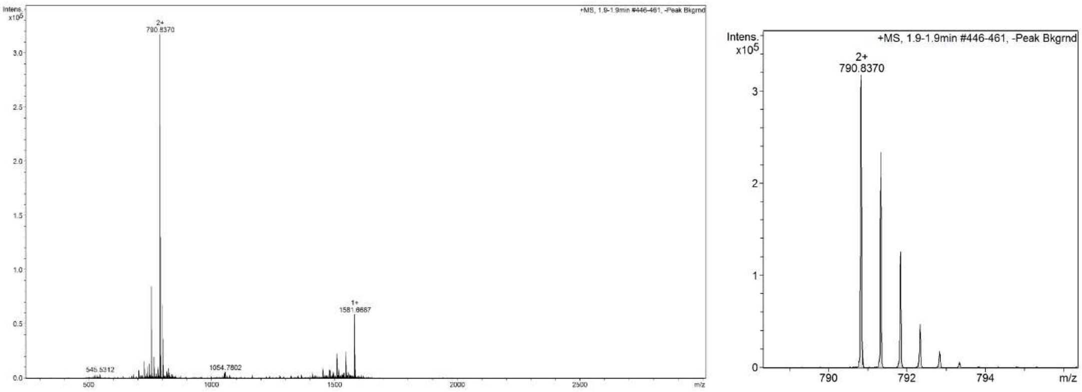

HR-QToF(ESI+)MS analysis of purified peptide **TACC3** $_{\mathbf{522\text{-}536\ F525C/P528C}}$

TACC3 $_{522\text{-}536\ R526C/A529C}$

Sequence: Ac- EESFCDPCEVLGTGA

HPLC (λ = 220 nm) tR = 6.452min. HR-QToF (ESI) m/z: [M+H] + Calc. for $C_{66}H_{100}N_{16}O_{26}S_2$: 1597.6509; Found:1597.6511.

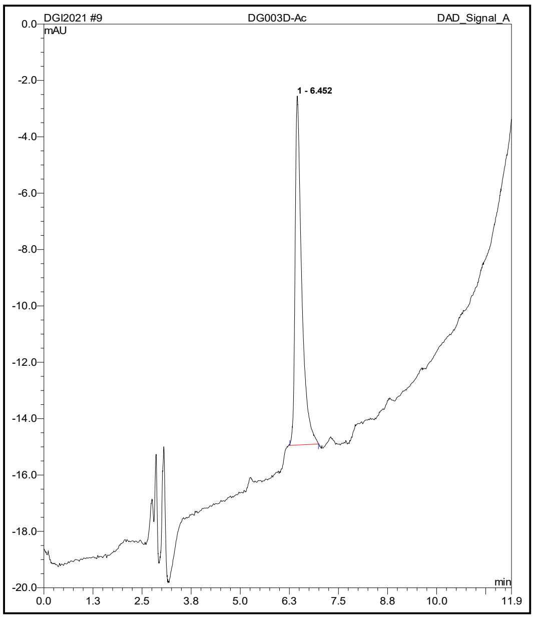

Analytical HPLC trace at λ= 220 nm of purified peptide **TACC3 $_{522\text{-}536\ R526C/A529C}$**

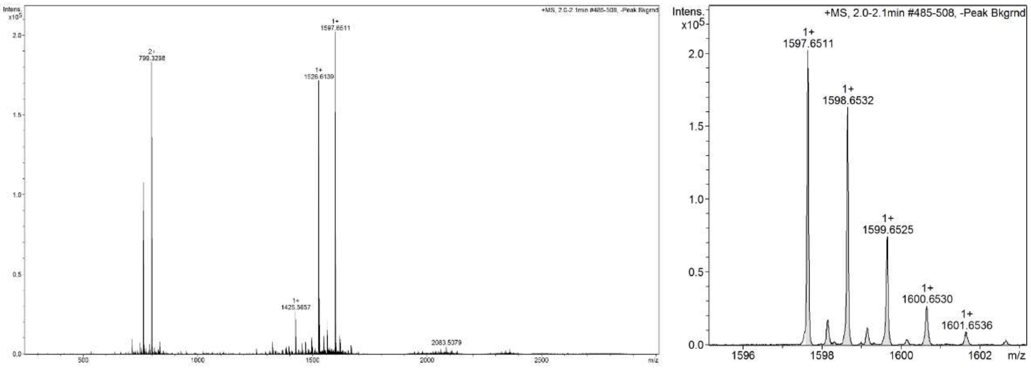

HR-QToF(ESI+)MS analysis of purified **TACC3 $_{522\text{-}536\ R526C/A529C}$**

TACC3 $_{\mathbf{522\text{-}536\ D527C/E530C}}$

Sequence: Ac- EESFRCPACVLGTGA

HPLC (λ = 220 nm) tR = 6.217min. HR-QToF (ESI) m/z: [M+H] + Calc. for $C_{66}H_{105}N_{19}O_{22}S_2$: 1580.7156; Found:1580.7195.

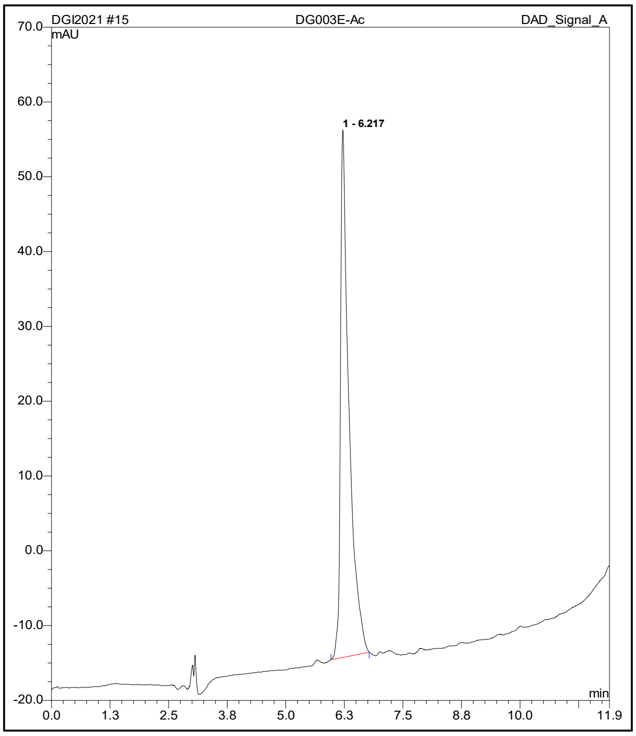

Analytical HPLC trace at λ= 220 nm of purified **TACC3** $_{\mathbf{522\text{-}536\ D527C/E530C}}$

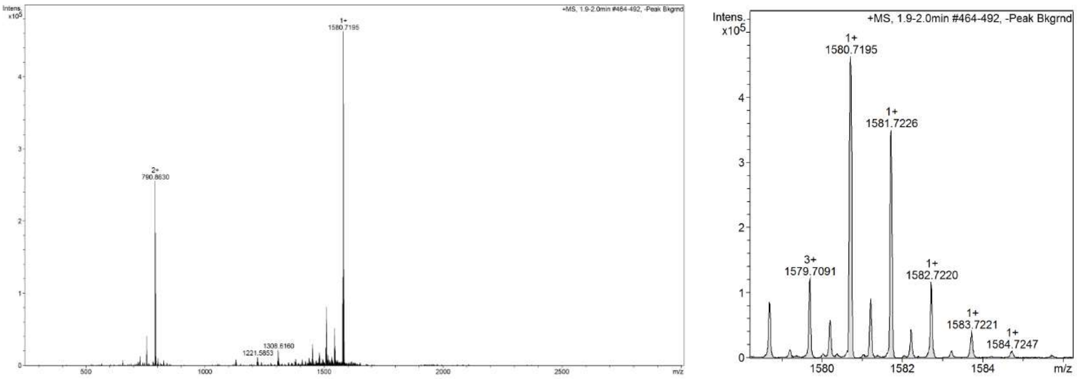

HR-QToF(ESI+)MS analysis of purified **TACC3** $_{\mathbf{522\text{-}536\ D527C/E530C}}$

TACC3 522-536 P528C/V531C

Sequence: Ac- EESFRDCAECLGTGA

HPLC (λ = 220 nm) tR = 6.021min. HR-QToF (ESI) m/z: [M+2H]2+ Calc. for $C_{65}H_{101}N_{19}O_{26}S_2$: 814.8376; Found:814.8376.

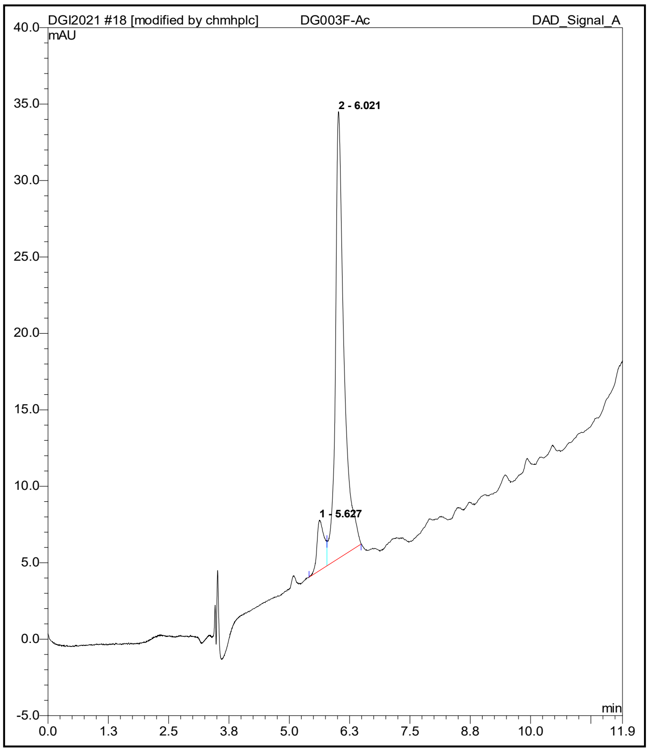

Analytical HPLC trace at λ= 220 nm of purified peptide **TACC3 522-536 P528C/V531C**

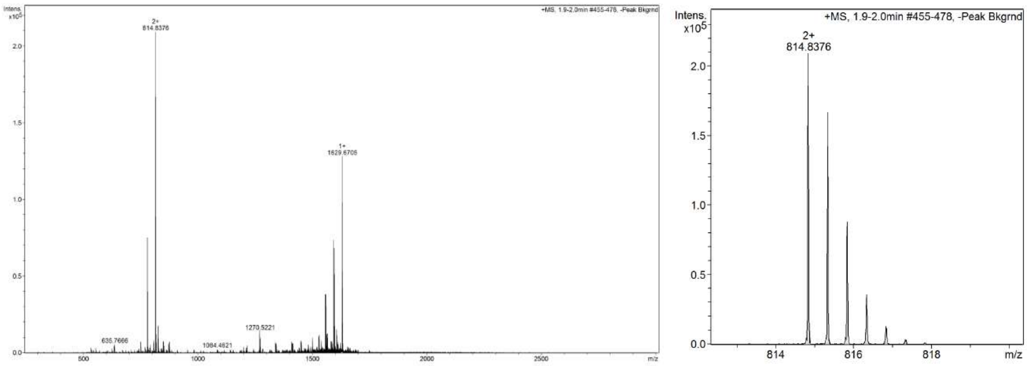

HR-QToF(ESI+)MS analysis of purified peptide **TACC3 522-536 P528C/V531C**

TACC3 **522-536 A529C/L532C**

Sequence: Ac- EESFRDPCEVCGTGA

HPLC (λ = 220 nm) tR = 5.458min. HR-QToF (ESI) m/z: [M+2H]2+ Calc. for $C_{66}H_{101}N_{19}O_{26}S_2$: 820.9376; Found:820.9381.

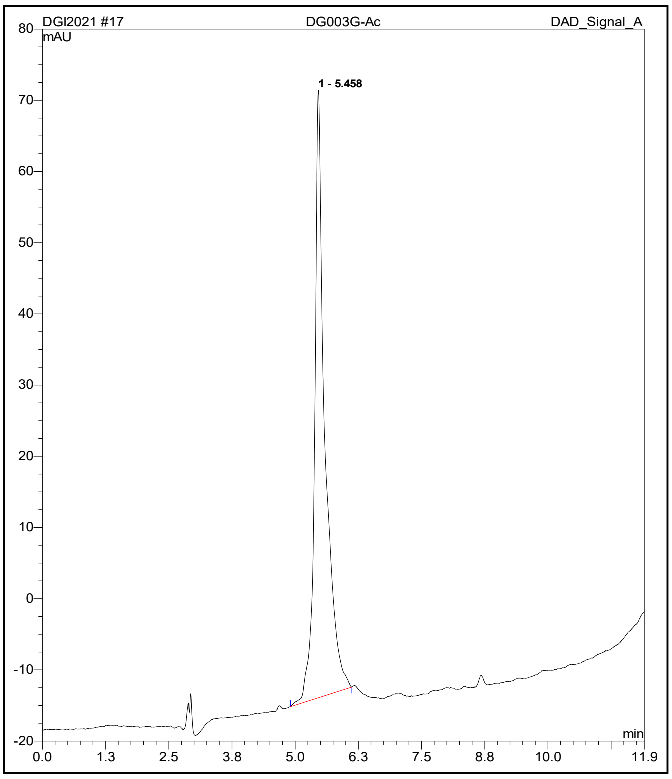

Analytical HPLC trace at λ= 220 nm of purified peptide **TACC3** **522-536 A529C/L532C**

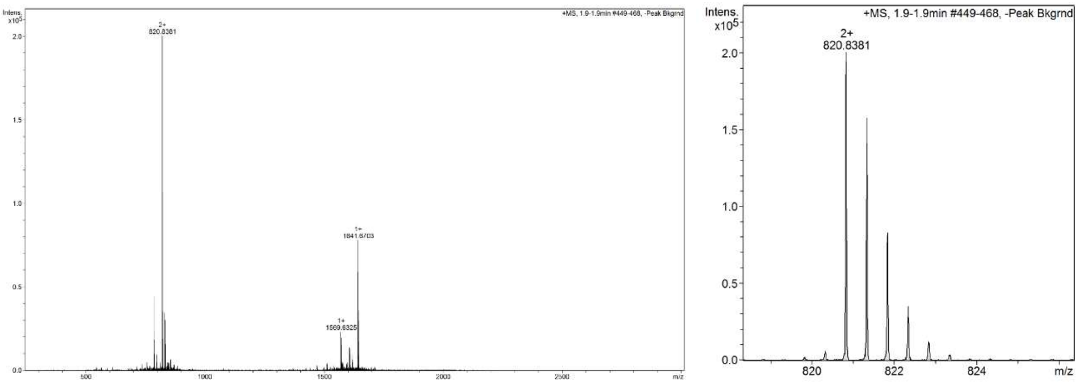

HR-QToF(ESI+)MS analysis of purified peptide **TACC3** **522-536 A529C/L532C**

TACC3 $_{522\text{-}536\ E530C/G533C}$

Sequence: Ac- EESFRDPACVLCTGA

HPLC (λ = 220 nm) tR = 3.477min. HR-QToF (ESI) m/z: [M+2H]2+ Calc. for $C_{68}H_{107}N_{19}O_{24}S_2$: 819.8662; Found:819.8664.

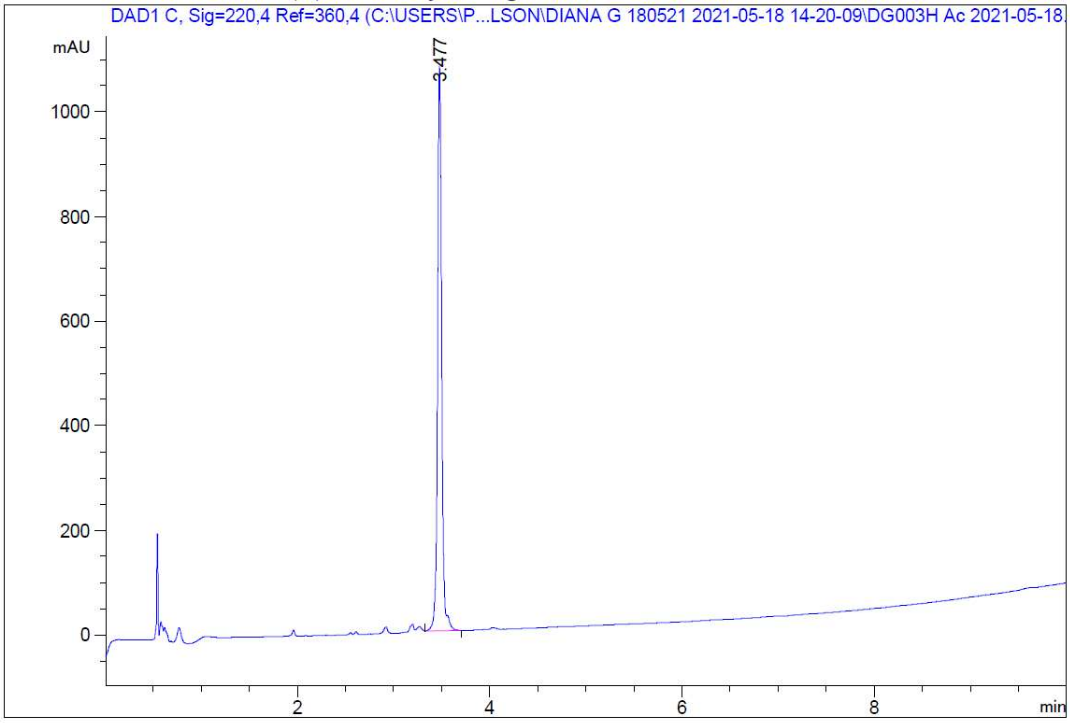

Analytical HPLC trace at λ= 220 nm of purified peptide **TACC3 $_{522\text{-}536\ E530C/G533C}$**

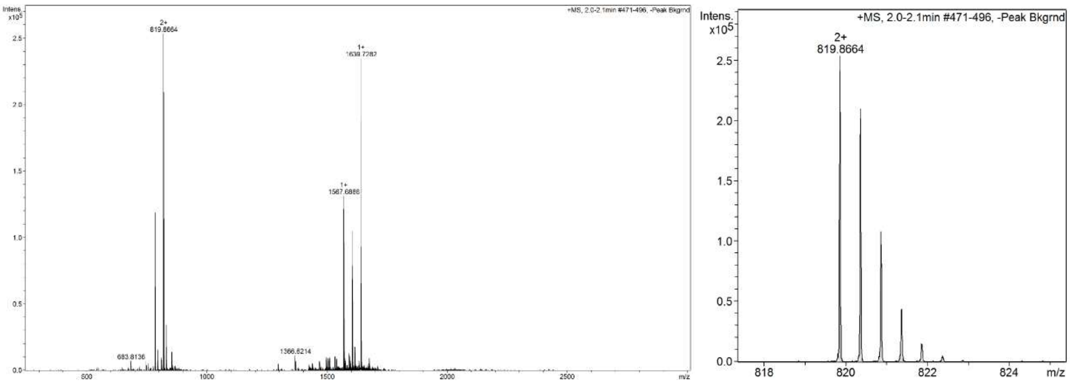

HR-QToF(ESI+)MS analysis of purified **TACC3 $_{522\text{-}536\ E530C/G533C}$**

TACC3 522-536 V531C/T534C

Sequence: Ac- EESFRDPAECLGCGA

HPLC (λ = 220 nm) tR = 3.477min. HR-QToF (ESI) m/z: [M+2H]2+ Calc. for $C_{66}H_{101}N_{19}O_{25}S_2$: 812.8401; Found:812.8399.

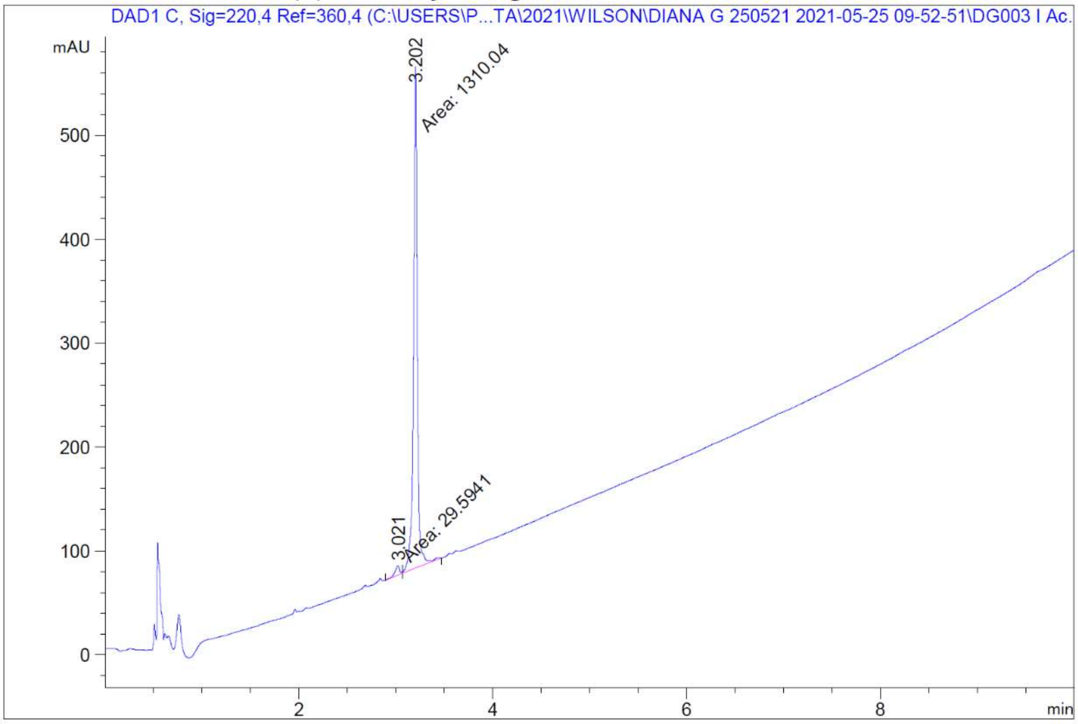

Analytical HPLC trace at λ= 220 nm of purified peptide **TACC3 522-536 V531C/T534C**

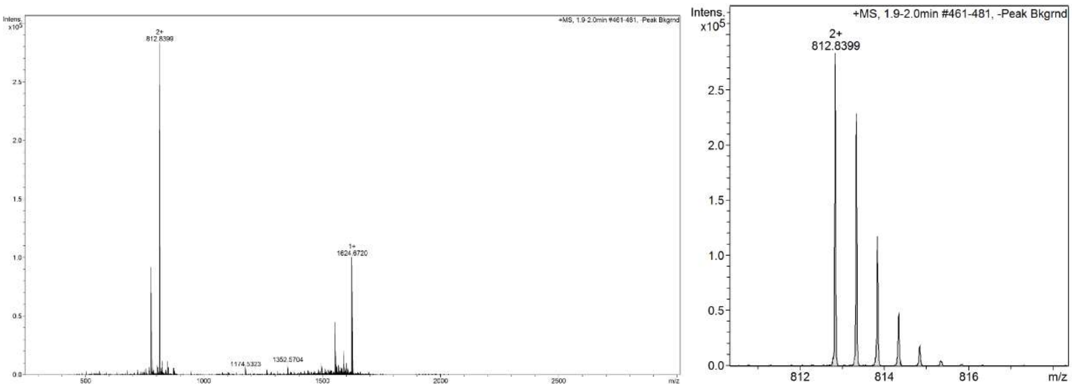

HR-QToF(ESI+)MS analysis of purified **TACC3 522-536 V531C/T534C**

TACC3 **$_{522\text{-}536\ L532C/G535C}$**

Sequence: Ac- EESFRDPAEVCGTCA

HPLC (λ = 220 nm) tR = 2.919min. HR-QToF (ESI) m/z: [M+2H]2+ Calc. for $C_{67}H_{103}N_{19}O_{26}S_2$: 827.8454; Found:827.8457.

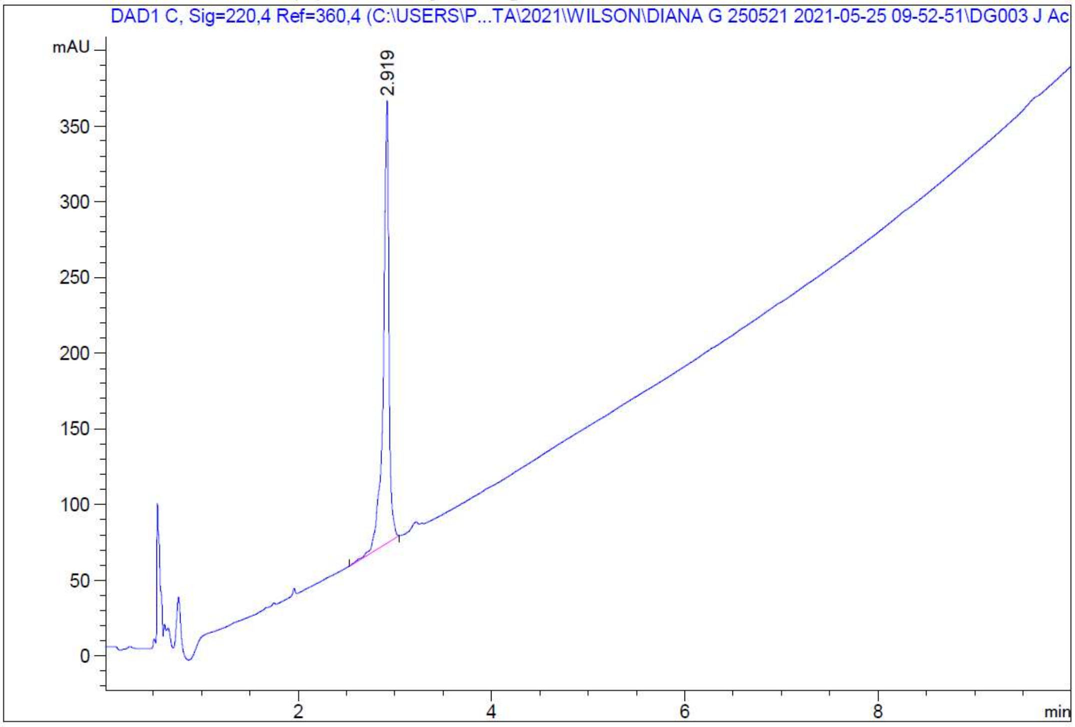

Analytical HPLC trace at λ= 220 nm of purified peptide **TACC3** **$_{522\text{-}536\ L532C/G535C}$**

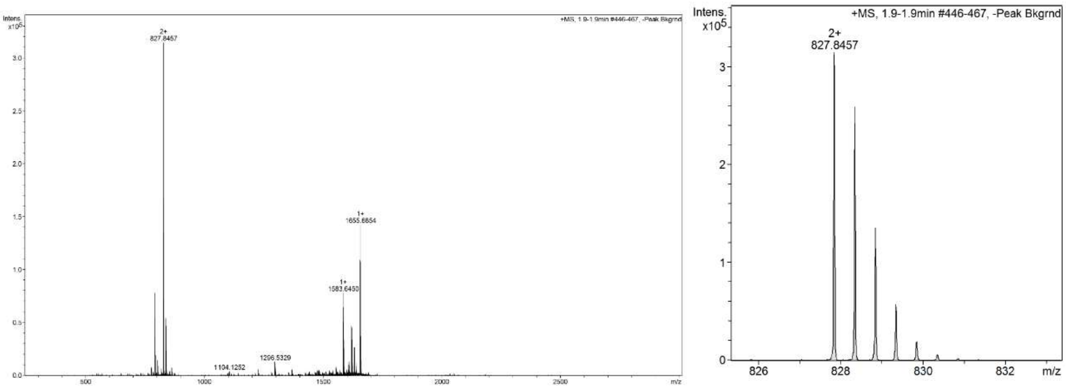

HR-QToF(ESI+)MS analysis of purified peptide **TACC3** **$_{522\text{-}536\ L532C/G535C}$**

TACC3 522-536 G533C/A536C

Sequence: Ac- EESFRDPAEVLCTGC

HPLC (λ = 220 nm) tR = 3.603min. HR-QToF (ESI) m/z: [M+2H]2+ Calc. for $C_{70}H_{109}N_{19}O_{26}S_2$: 848.8698; Found:848.8693.

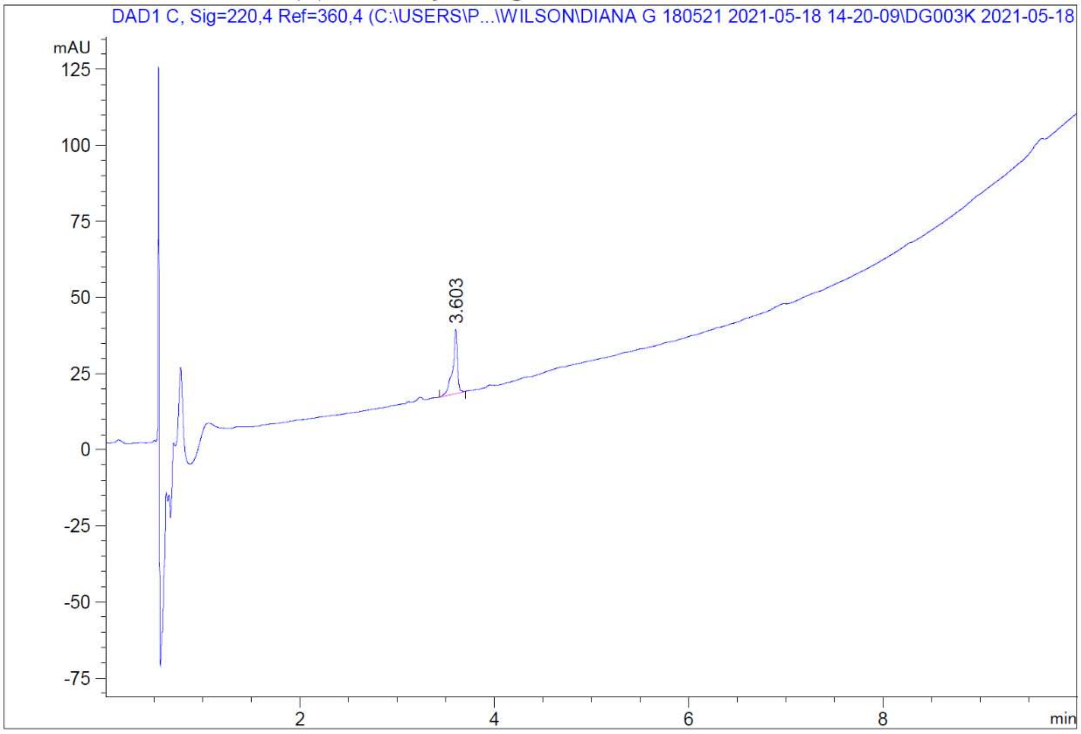

Analytical HPLC trace at λ= 220 nm of purified peptide **TACC3 522-536 G533C/A536C**

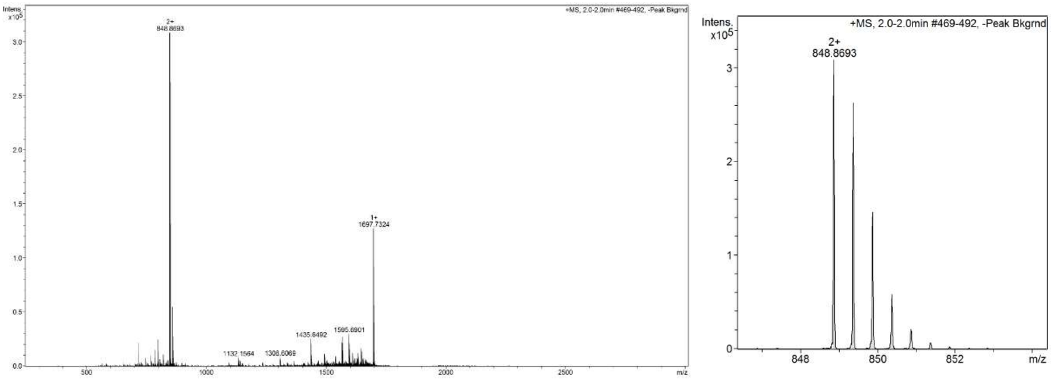

HR-QToF(ESI+)MS analysis of purified peptide **TACC3 522-536 G533C/A536C**

TACC3 $_{522\text{-}536\ R526C/V531C}$

Sequence: Ac- EESF**C**DPAE**C**LGTGA

HPLC (λ = 220 nm) tR = 3.339min. HR-QToF (ESI) m/z: [M+2H]2+ Calc. for $C_{64}H_{96}N_{16}O_{26}S_2$: 785.3134; Found:785.3133.

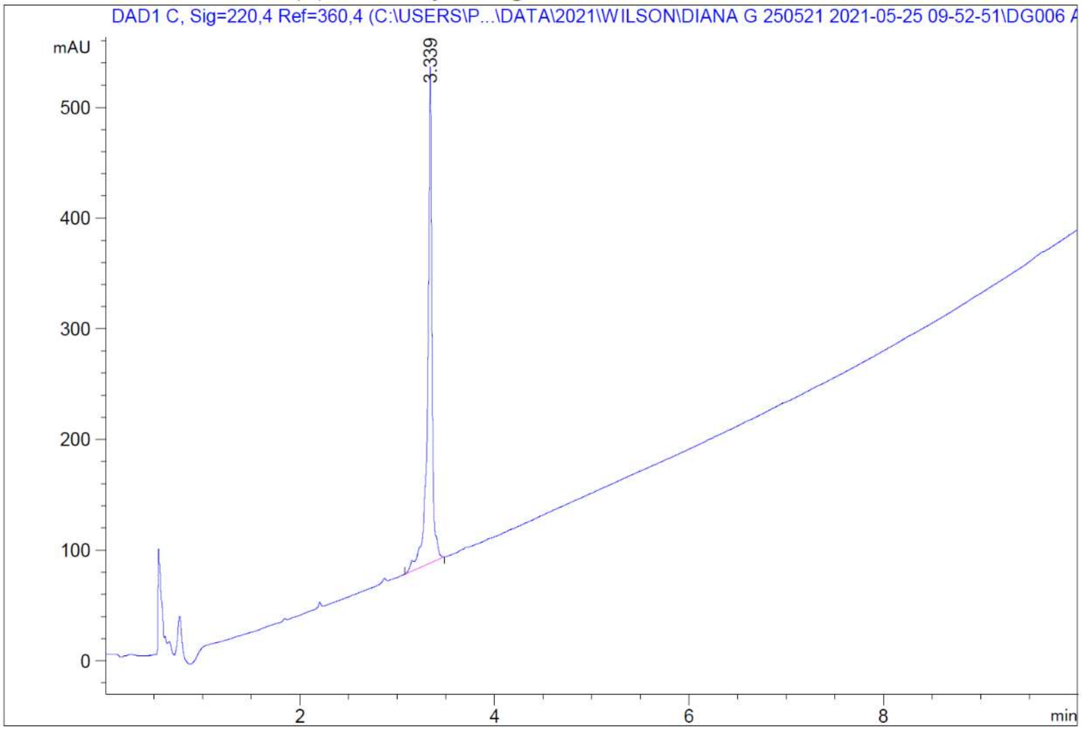

Analytical HPLC trace at λ= 220 nm of purified peptide **TACC3 $_{522\text{-}536\ R526C/V531C}$**

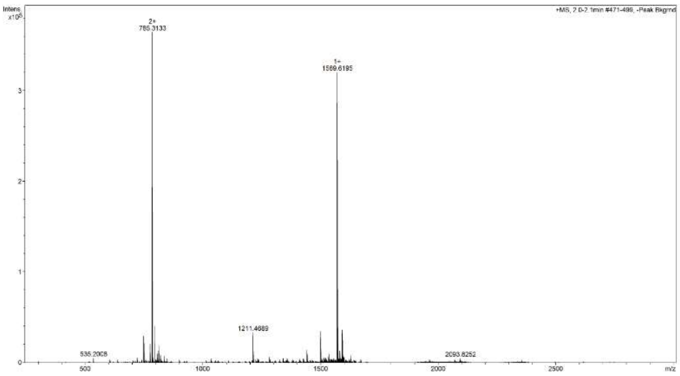

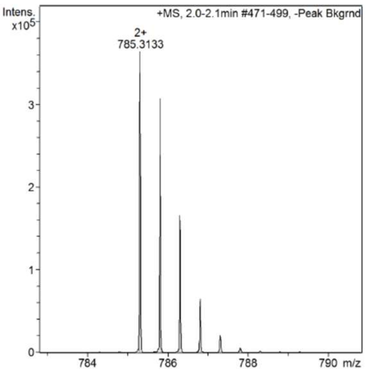

HR-QToF(ESI+)MS analysis of purified peptide **TACC3 $_{522\text{-}536\ R526C/V531C}$**

TACC3 $_{522\text{-}536\ E522C/P528C}$

Sequence: Ac- **C**ESFRD**C**AEVLGTGA

HPLC (λ = 220 nm) tR = 3.475min. HR-QToF (ESI) m/z: [M-Ala+2H]2+ Calc. for $C_{62}H_{98}N_{18}O_{23}S_2$: 764.3320; Found:764.3312.

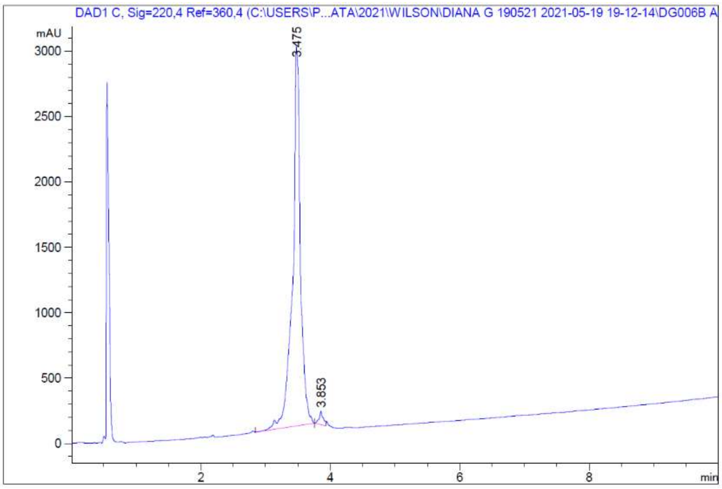

Analytical HPLC trace at λ= 220 nm of purified peptide **TACC3 $_{522\text{-}536\ E522C/P528C}$**

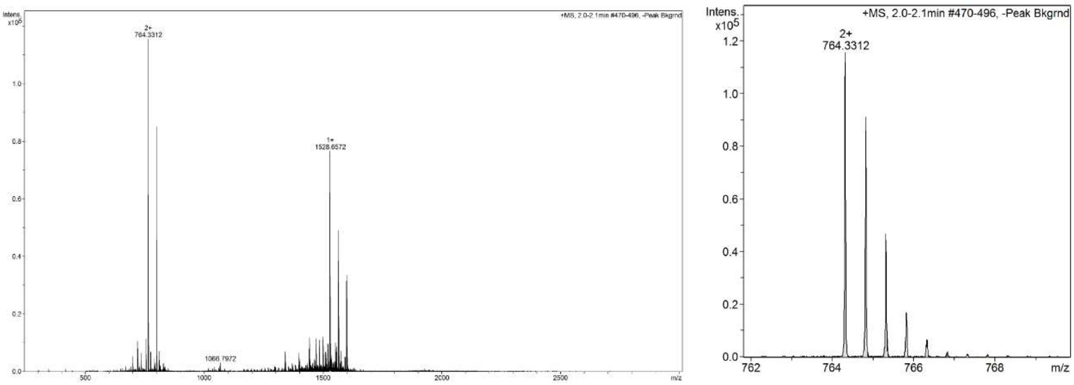

HR-QToF(ESI+)MS analysis of purified peptide **TACC3 $_{522\text{-}536\ E522C/P528C}$**

TACC3 $_{522\text{-}536\ A529C/G535C}$

Sequence: Ac- EESFRDP**C**EVLGT**C**A

HPLC (λ = 220 nm) tR = 3.503min. HR-QToF (ESI) m/z: [M+2H]2+ Calc. for $C_{70}H_{109}N_{19}O_{26}S_2$: 848.8689; Found:848.8691.

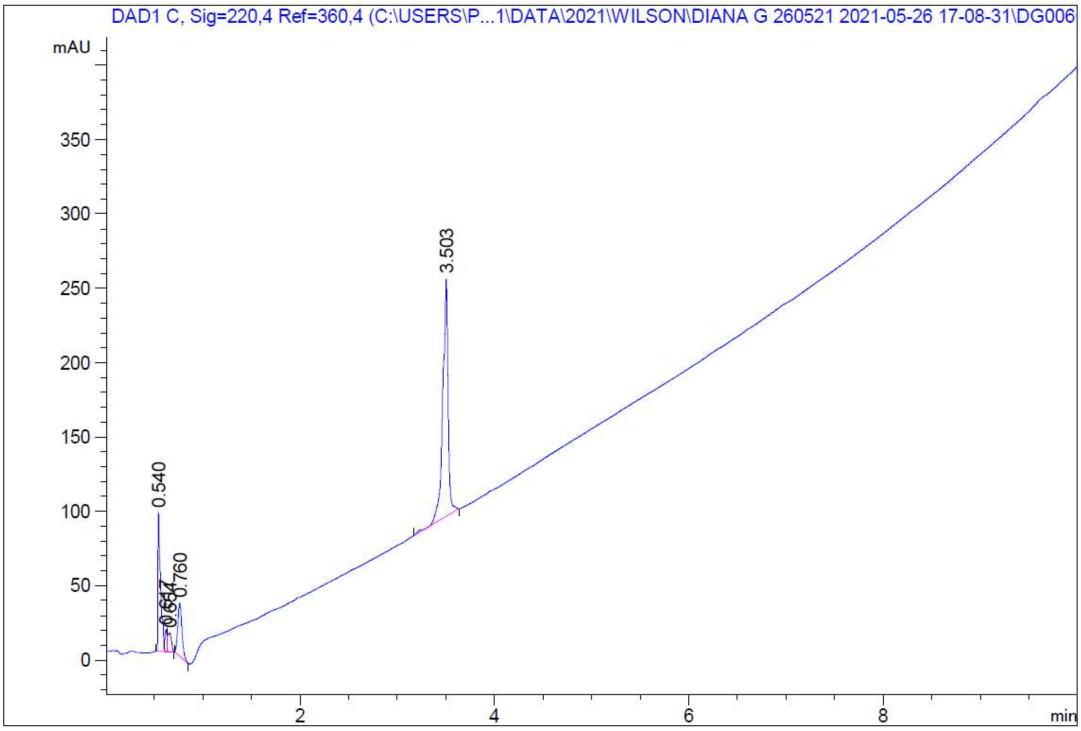

Analytical HPLC trace at λ= 220 nm of purified peptide **TACC3 $_{522\text{-}536\ A529C/G535C}$**

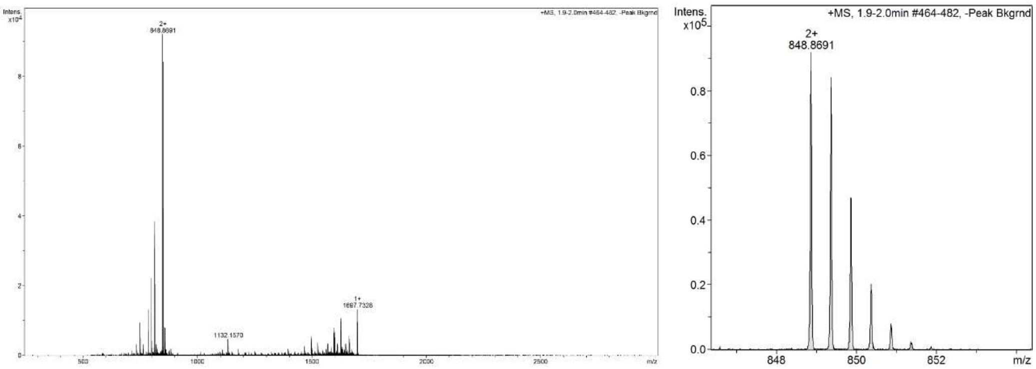

HR-QToF(ESI+)MS analysis of purified peptide **TACC3 $_{522\text{-}536\ A529C/G535C}$**

TACC3 **522-536 E523C/R526C mal**

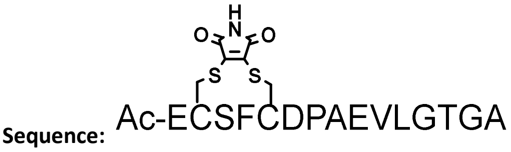

HPLC (λ = 220 nm) tR = 4.82min. HR-QToF (ESI) m/z: [M+2H]2+ Calc. for $C_{68}H_{97}N_{17}O_{26}S_2$: 816.8189; Found: 816.8189.

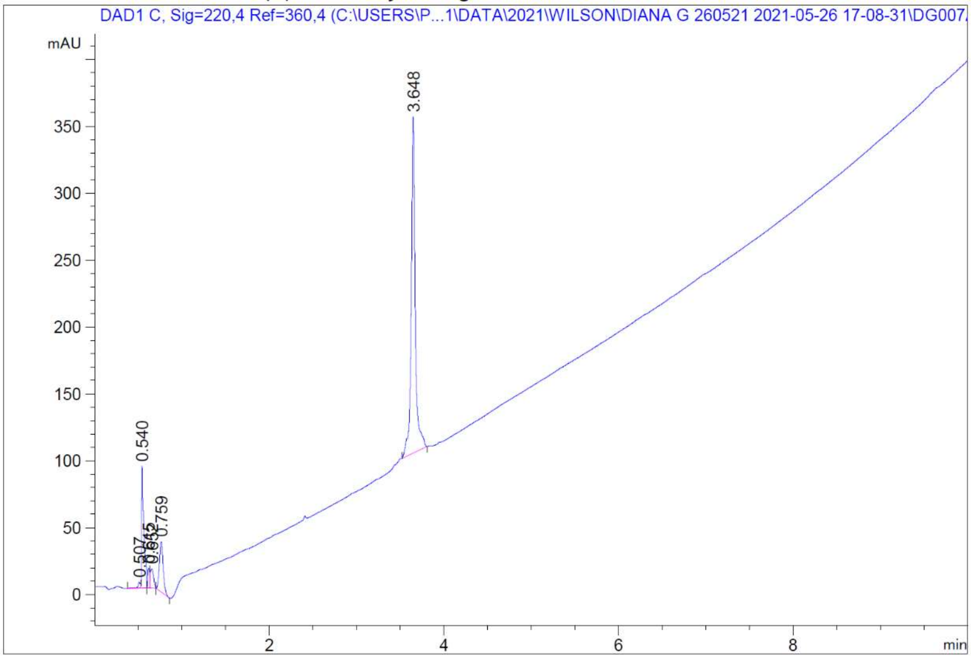

Analytical HPLC trace at λ= 220 nm of purified **TACC3** **522-536 E523C/R526C mal**

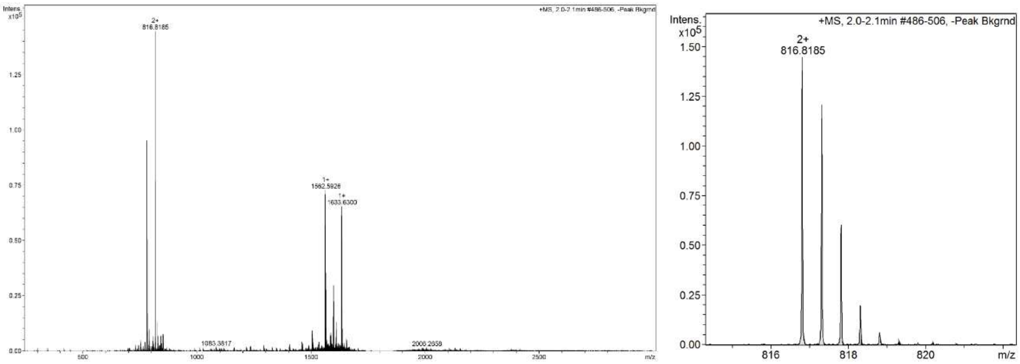

HR-QToF(ESI+)MS analysis of purified **TACC3** **522-536 E523C/R526C mal**

TACC3 **522-536 S524C/D527C mal**

Sequence: 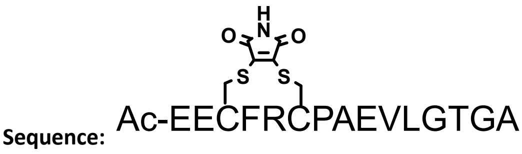

HPLC (λ = 220 nm) tR = 4.82min. HR-QToF (ESI) m/z: [M+2H]2+ Calc. for $C_{72}H_{106}N_{20}O_{25}S_2$: 858.3612; Found: 858.3623.

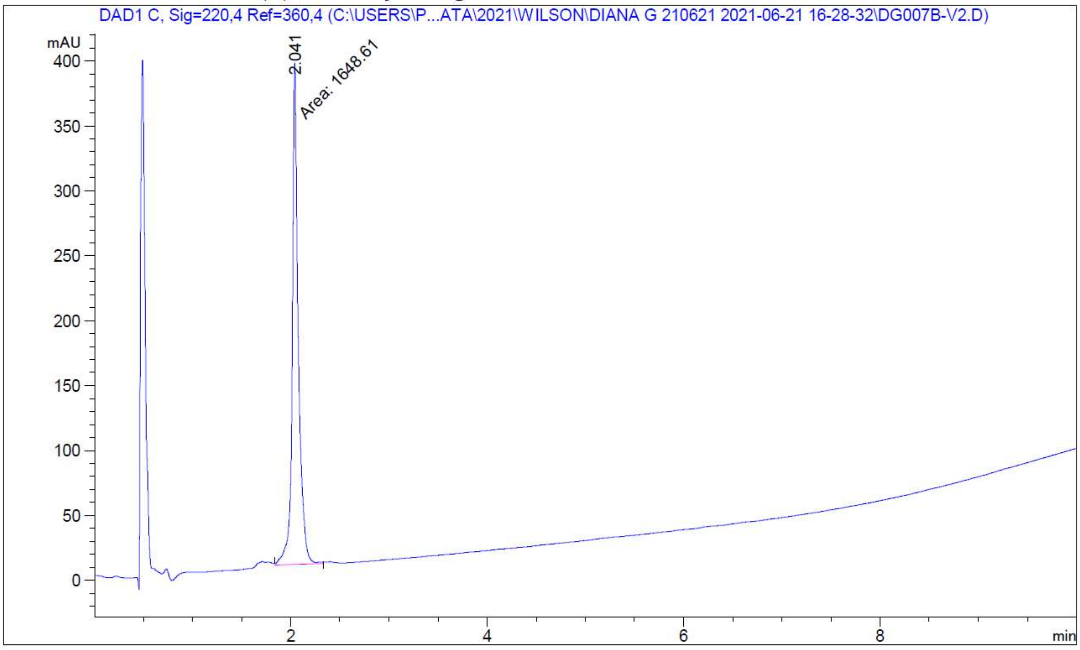

Analytical HPLC trace at λ= 220 nm of purified peptide **TACC3** **522-536 S524C/D527C mal**

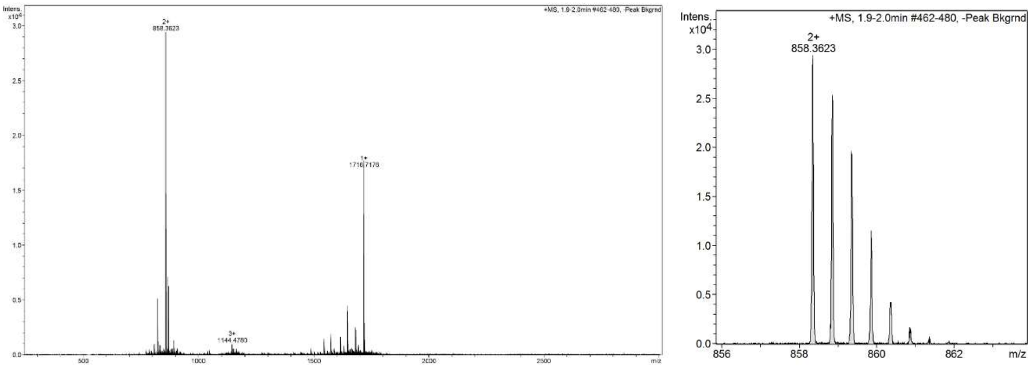

HR-QToF(ESI+)MS analysis of purified peptide **TACC3** **522-536 S524C/D527C mal**

TACC3 $_{522\text{-}536\ F525C/P528C\ mal}$

Sequence: 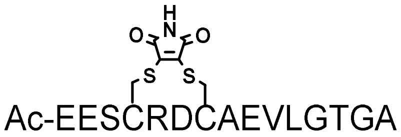

HPLC (λ = 220 nm) tR = 1.640min. HR-QToF (ESI) m/z: [M+2H]2+ Calc. for $C_{65}H_{100}N_{20}O_{28}S_2$: 837.3301; Found: 837.3317.

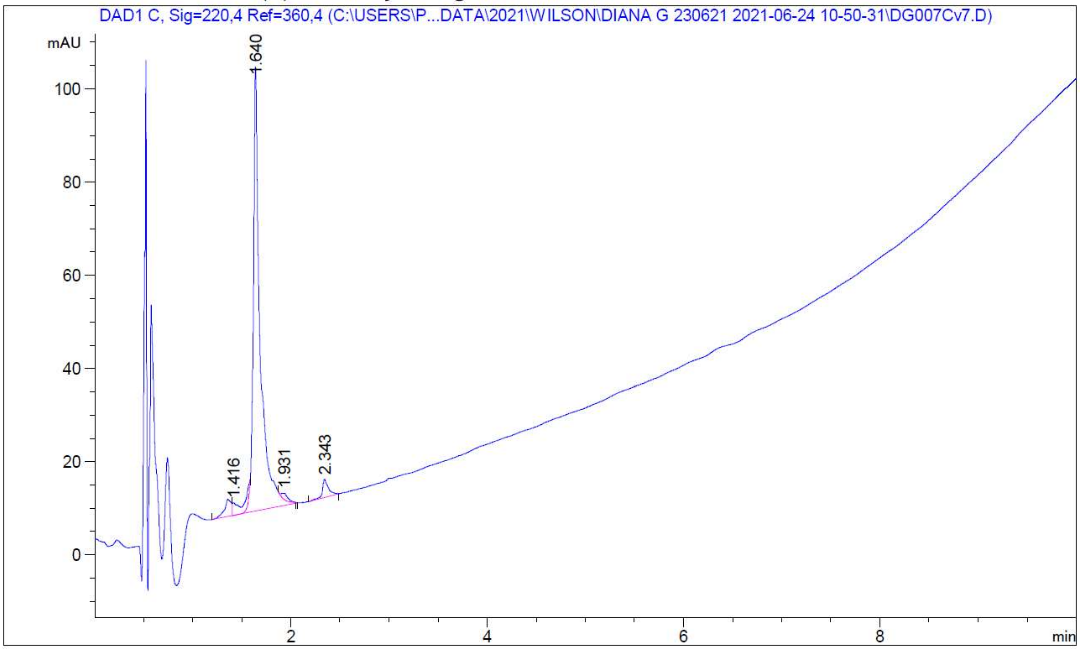

Analytical HPLC trace at λ= 220 nm of purified **TACC3 $_{522\text{-}536\ F525C/P528C\ mal}$**

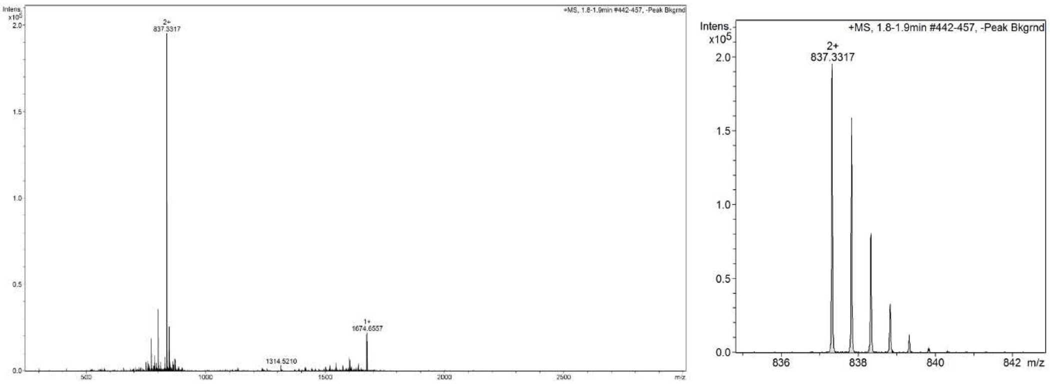

HR-QToF(ESI+)MS analysis of purified **TACC3 $_{522\text{-}536\ F525C/P528C\ mal}$**

TACC3 **522-536 R526C/A529C mal**

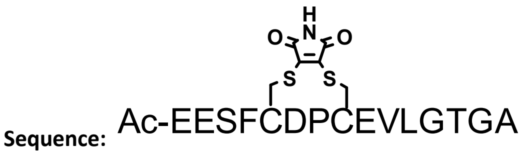

HPLC (λ = 220 nm) tR = 3.355min. HR-QToF (ESI) m/z: [M+2H]2+ Calc. for $C_{70}H_{99}N_{17}O_{28}S_2$: 845.8216; Found: 845.8216.

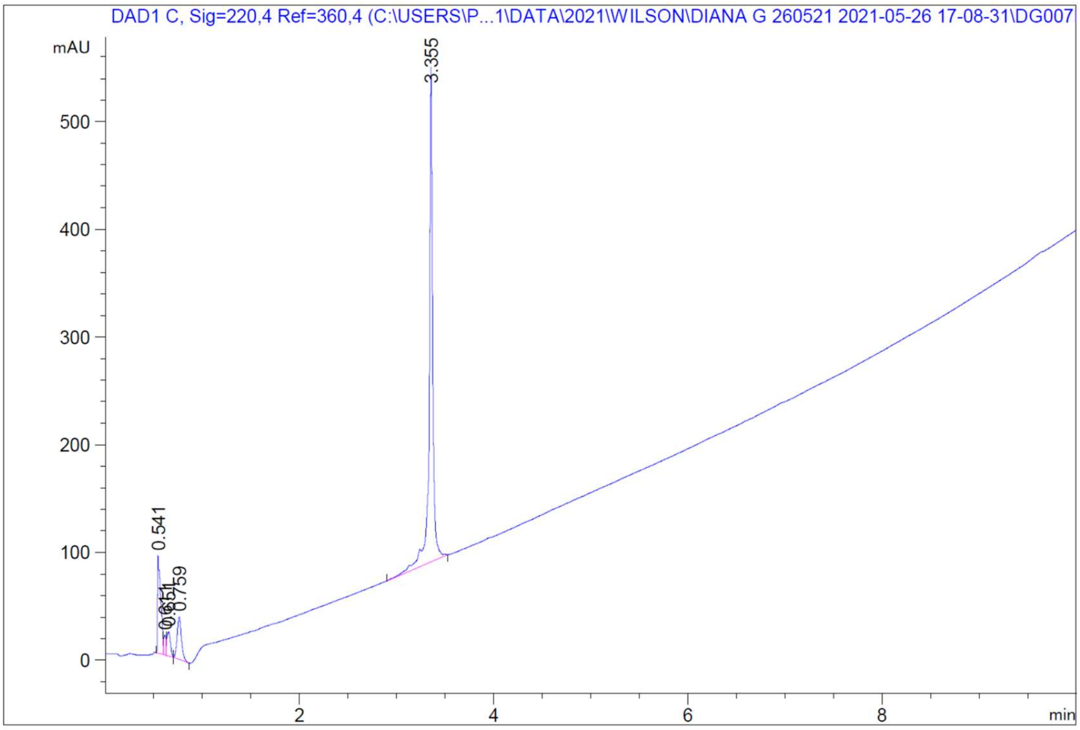

Analytical HPLC trace at λ= 220 nm of purified **TACC3** **522-536 R526C/A529C mal**

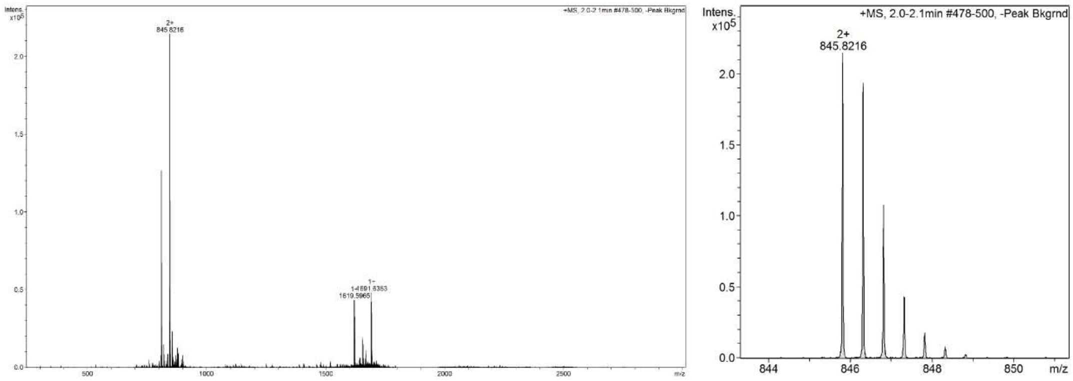

HR-QToF(ESI+)MS analysis of purified **TACC3** **522-536 R526C/A529C mal**

TACC3 $_{522\text{-}536\ D527C/E530C\ mal}$

Sequence: 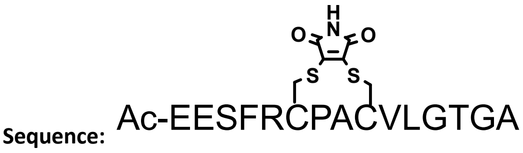

HPLC (λ = 220 nm) tR = 2.442min. HR-QToF (ESI) m/z: [M+H] + Calc. for $C_{70}H_{104}N_{20}O_{24}S_2$: 1073.7046; Found: 1073.7029.

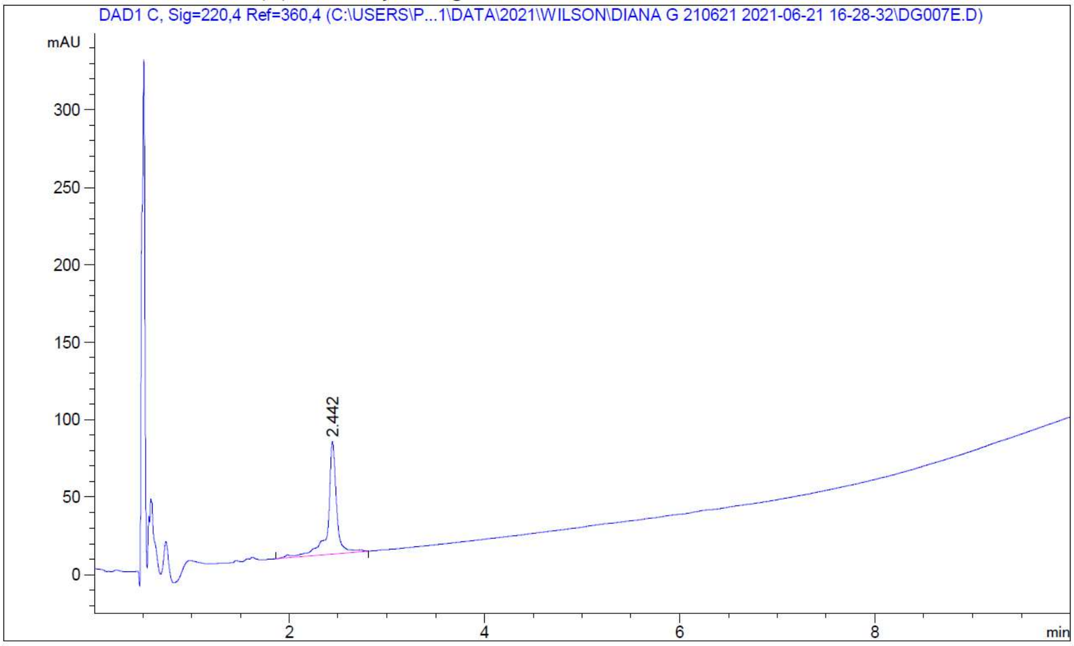

Analytical HPLC trace at λ= 220 nm of purified **TACC3 $_{522\text{-}536\ D527C/E530C\ mal}$**

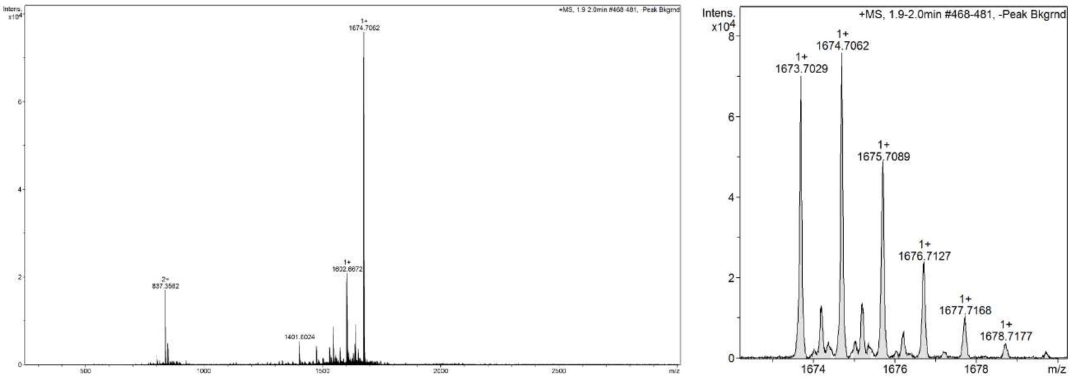

HR-QToF(ESI+)MS analysis of purified **TACC3 $_{522\text{-}536\ D527C/E530C\ mal}$**

TACC3 **522-536 P528C/V531C mal**

Sequence: 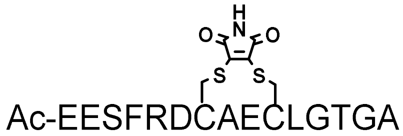

HPLC (λ = 220 nm) tR = 1.824min. HR-QToF (ESI) m/z: [M+2H]2+ Calc. for $C_{69}H_{100}N_{20}O_{28}S_2$: 861.3301; Found: 861.3317.

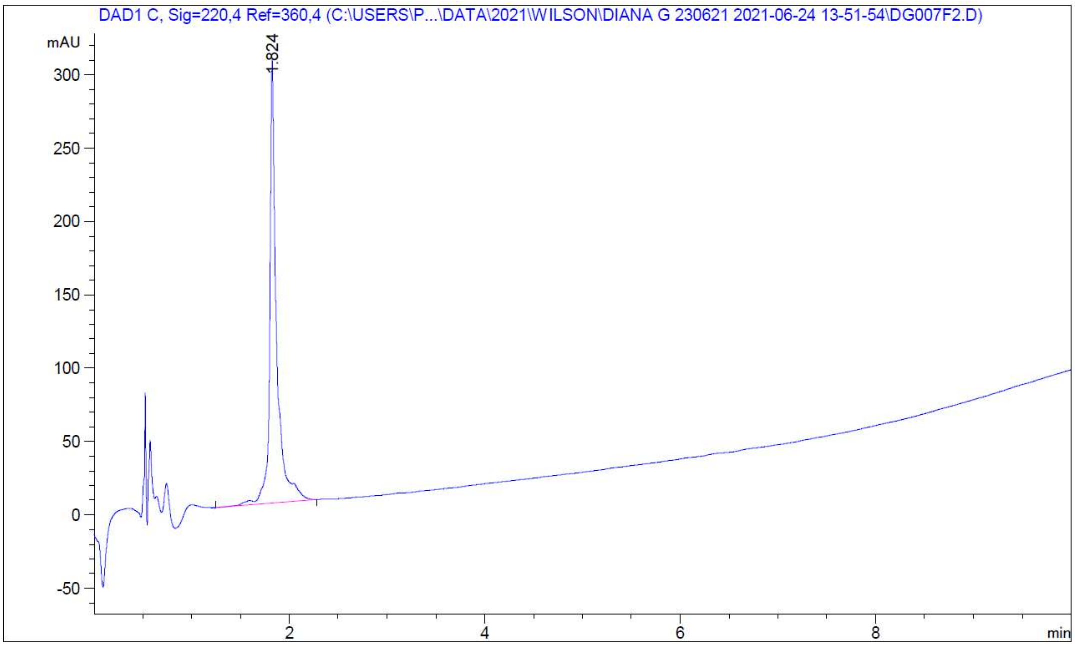

Analytical HPLC trace at λ= 220 nm of purified **TACC3** **522-536 P528C/V531C mal**

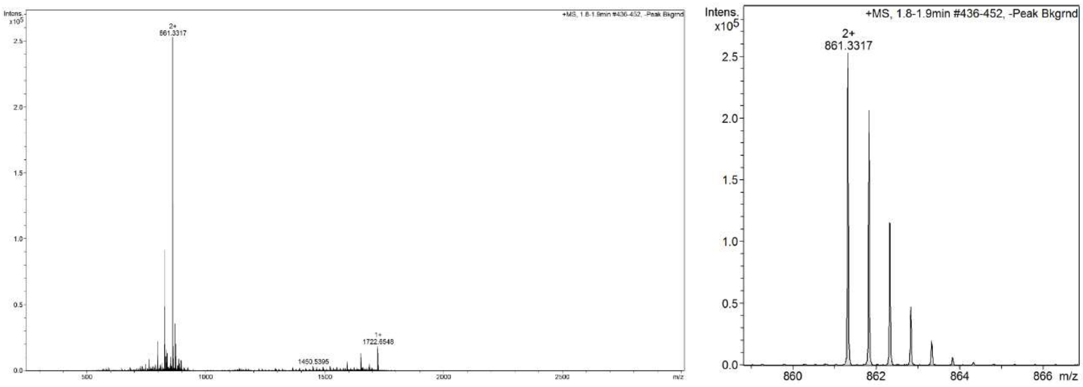

HR-QToF(ESI+)MS analysis of purified **TACC3** **522-536 P528C/V531C mal**

TACC3 **522-536 A529C/L532C mal**

Sequence: 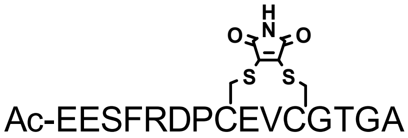

HPLC (λ = 220 nm) tR = 1.893min. HR-QToF (ESI) m/z: [M+2H]2+ Calc. for $C_{70}H_{100}N_{20}O_{28}S_2$: 1733.6530; Found: 1733.6510.

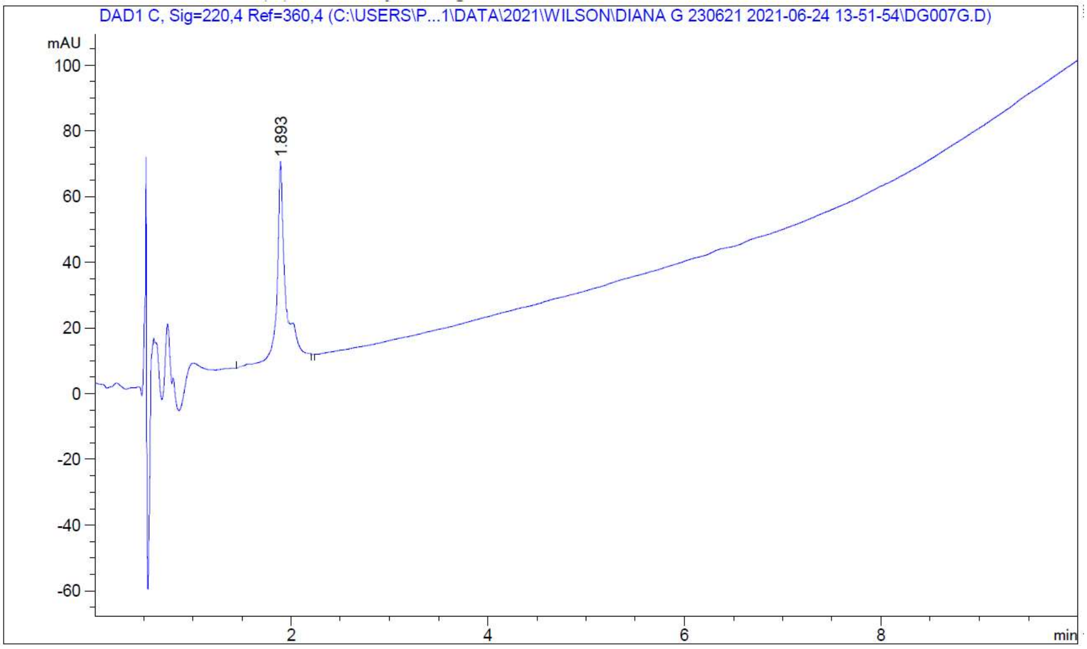

Analytical HPLC trace at λ= 220 nm of purified **TACC3** **522-536 A529C/L532C mal**

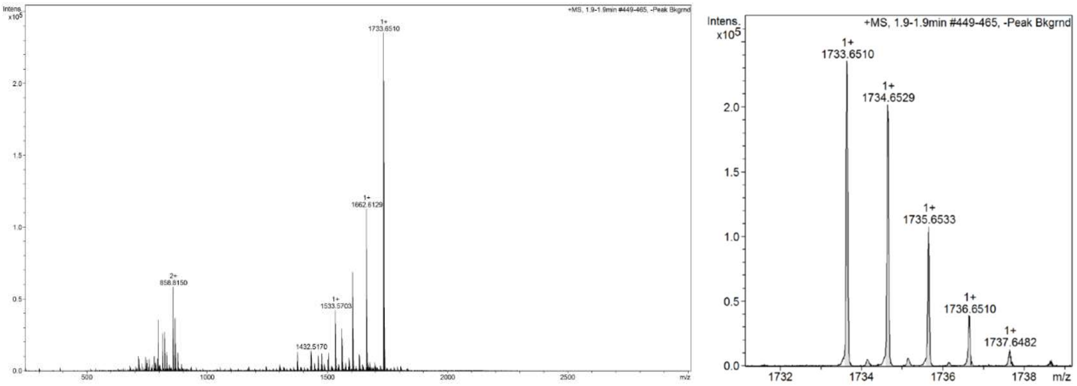

HR-QToF(ESI+)MS analysis of purified **TACC3** **522-536 A529C/L532C mal**

TACC3 **522-536 E530C/G533C mal**

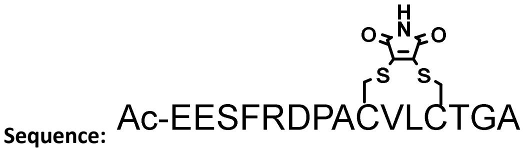

HPLC (λ = 220 nm) tR = 3.566min. HR-QToF (ESI) m/z: [M+2H]2+ Calc. for $C_{72}H_{106}N_{20}O_{26}S_2$: 866.3587; Found: 866.3596.

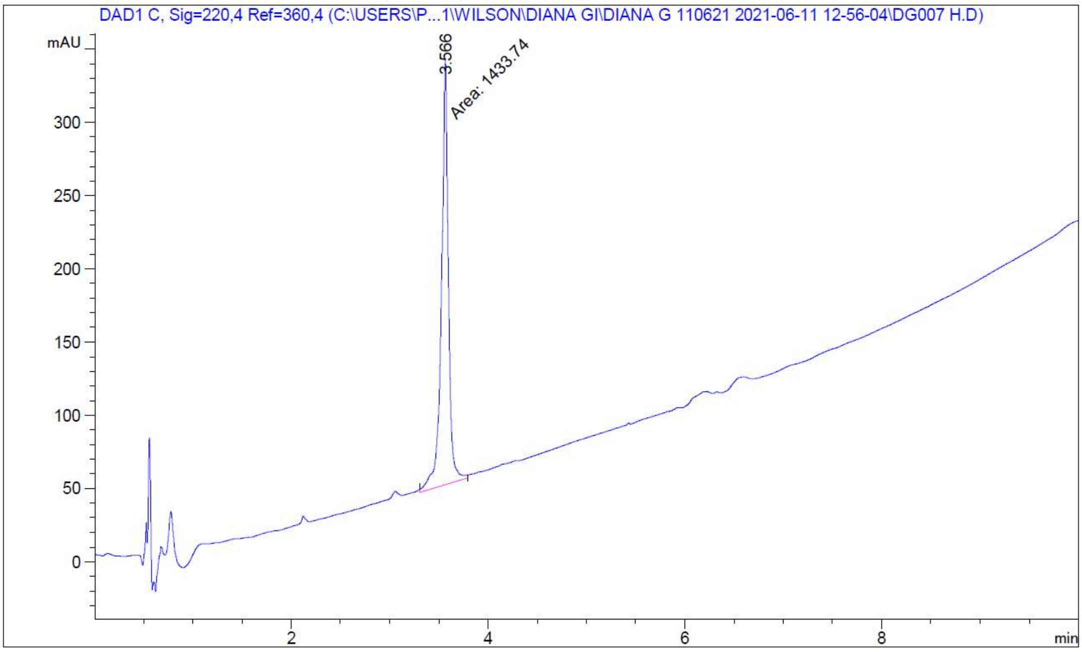

Analytical HPLC trace at λ= 220 nm of purified **TACC3** **522-536 E530C/G533C mal**

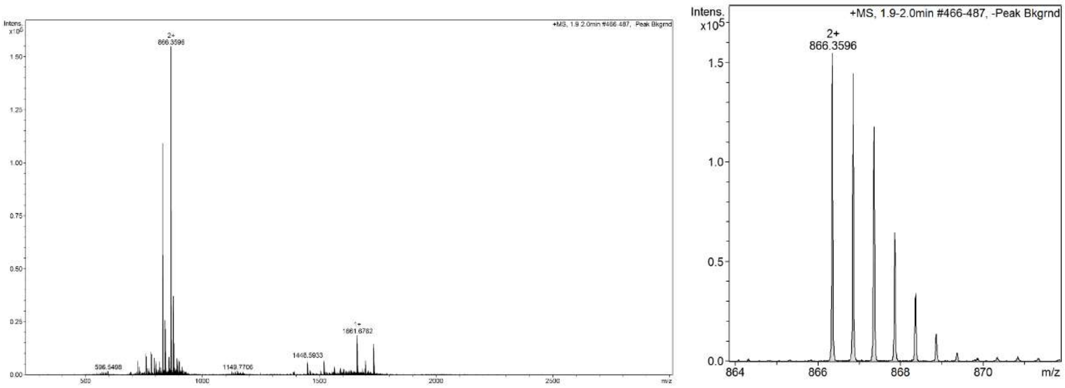

HR-QToF(ESI+)MS analysis of purified **TACC3** **522-536 E530C/G533C mal**

TACC3 **522-536 V531C/T534C mal**

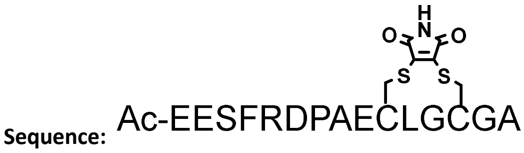

HPLC (λ = 220 nm) tR = 1.851min. HR-QToF (ESI) m/z: [M+2H]2+ Calc. for $C_{70}H_{100}N_{20}O_{27}S_2$: 859.3327; Found: 859.3341.

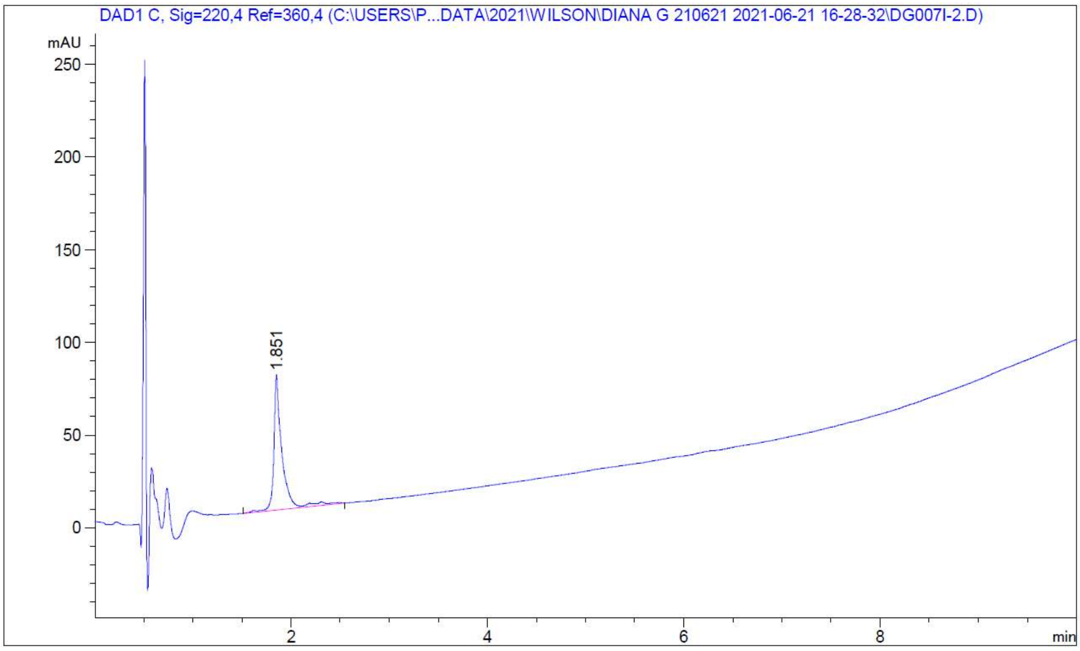

Analytical HPLC trace at λ= 220 nm of purified **TACC3** **522-536 V531C/T534C mal**

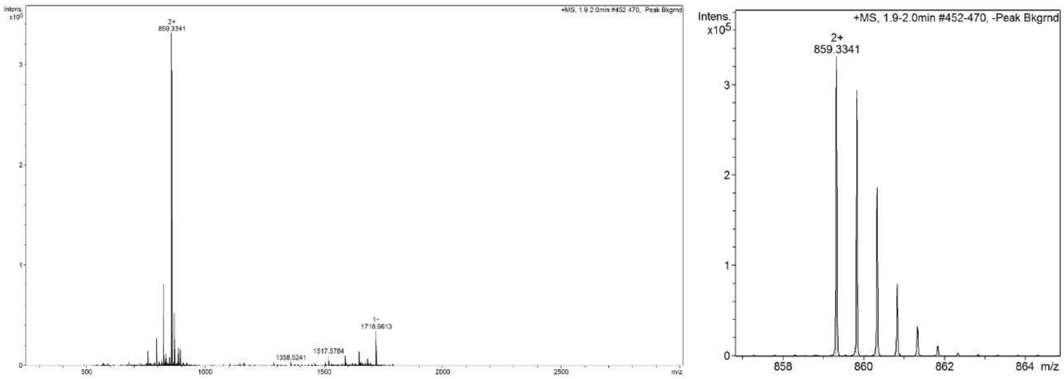

HR-QToF(ESI+)MS analysis of purified **TACC3** **522-536 V531C/T534C mal**

TACC3 $_{522\text{-}536\ L532C/G535C\ mal}$

Sequence: 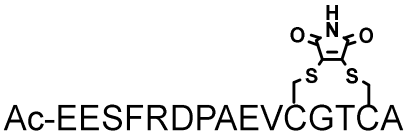

HPLC (λ = 220 nm) tR = 1.843min. HR-QToF (ESI) m/z: [M+2H]2+ Calc. for $C_{71}H_{102}N_{20}O_{28}S_2$: 874.3380; Found: 874.3404.

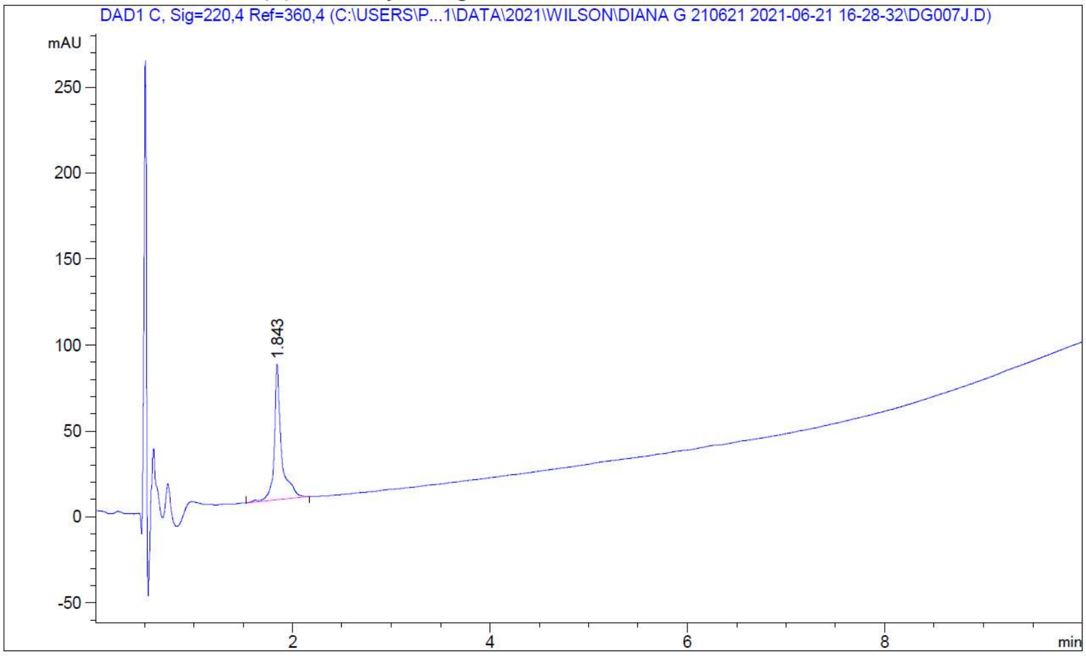

Analytical HPLC trace at λ= 220 nm of purified **TACC3 $_{522\text{-}536\ L532C/G535C\ mal}$**

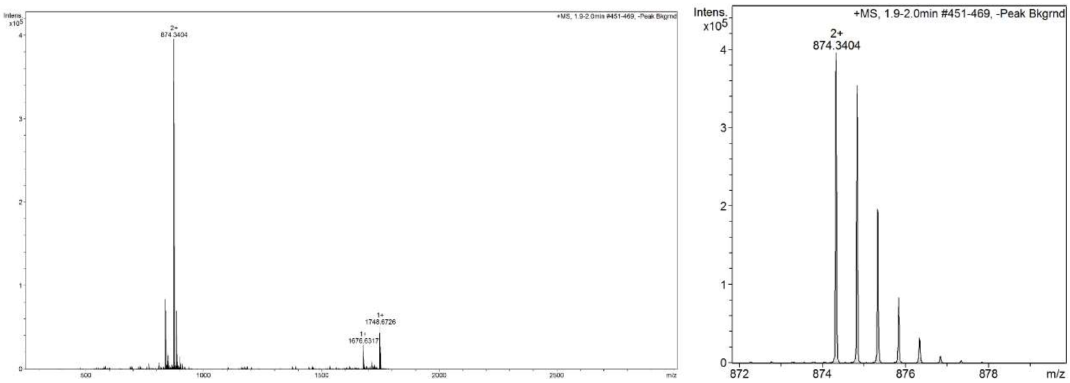

HR-QToF(ESI+)MS analysis of purified **TACC3 $_{522\text{-}536\ L532C/G535C\ mal}$**

TACC3 **522-536 G533C/A536C mal**

Sequence: 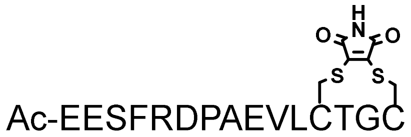

HPLC (λ = 220 nm) tR = 2.361min. HR-QToF (ESI) m/z: [M+2H]2+ Calc. for $C_{74}H_{108}N_{20}O_{28}S_2$: 895.3614; Found: 895.3630.

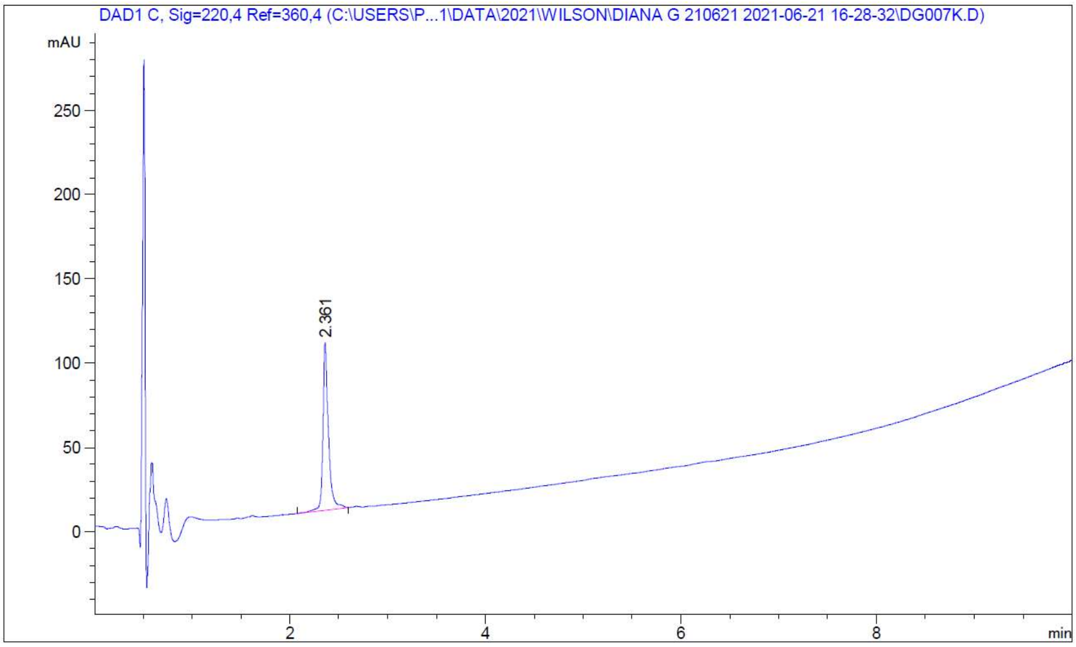

Analytical HPLC trace at λ= 220 nm of purified **TACC3** **522-536 G533C/A536C mal**

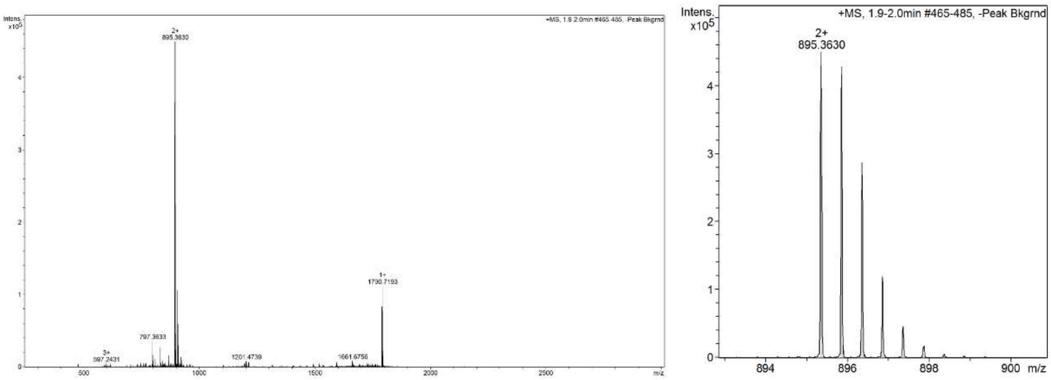

HR-QToF(ESI+)MS analysis of purified **TACC3** **522-536 G533C/A536C mal**

TACC3 $_{522\text{-}536\ R526C/V531C\ Bph}$

Sequence:

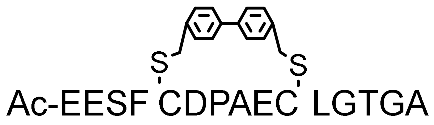

HPLC (λ = 220 nm) tR = 3.682min. HR-QToF (ESI) m/z: [M+2H]2+ Calc. for $C_{78}H_{106}N_{16}O_{26}S_2$: 874.3126; Found: 874.3145.

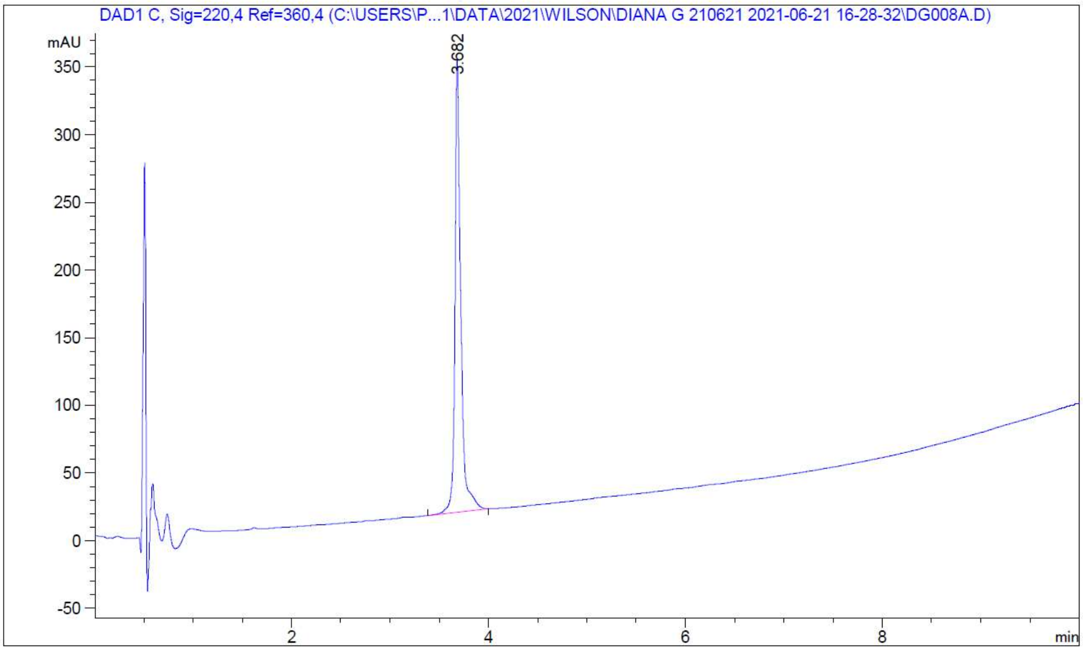

Analytical HPLC trace at λ= 220 nm of purified **TACC3 $_{522\text{-}536\ R526C/V531C\ Bph}$**

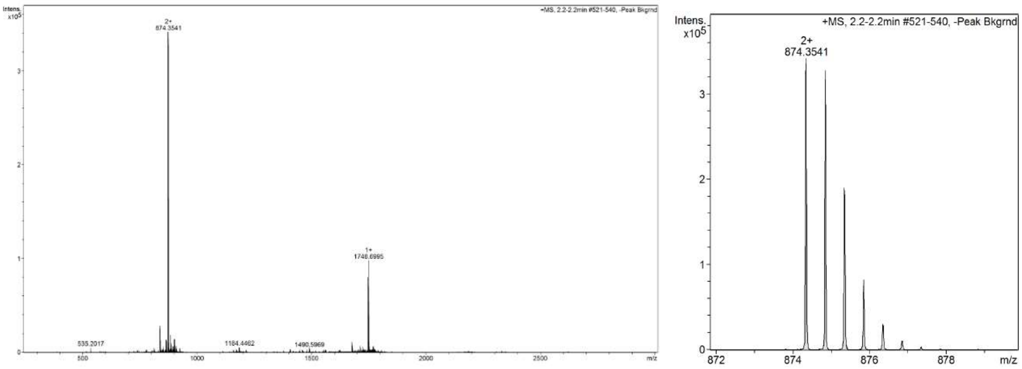

HR-QToF(ESI+)MS analysis of purified **TACC3 $_{522\text{-}536\ R526C/V531C\ Bph}$**

TACC3 522-536 E522C/P528C Bph

Sequence:

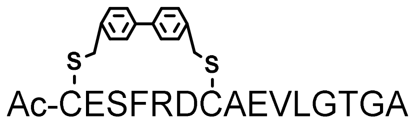

HPLC (λ = 220 nm) tR = 4.211min. HR-QToF (ESI) m/z: [M+2H]2+ Calc. for $C_{79}H_{113}N_{19}O_{24}S_2$: 888.8896; Found: 888.8909.

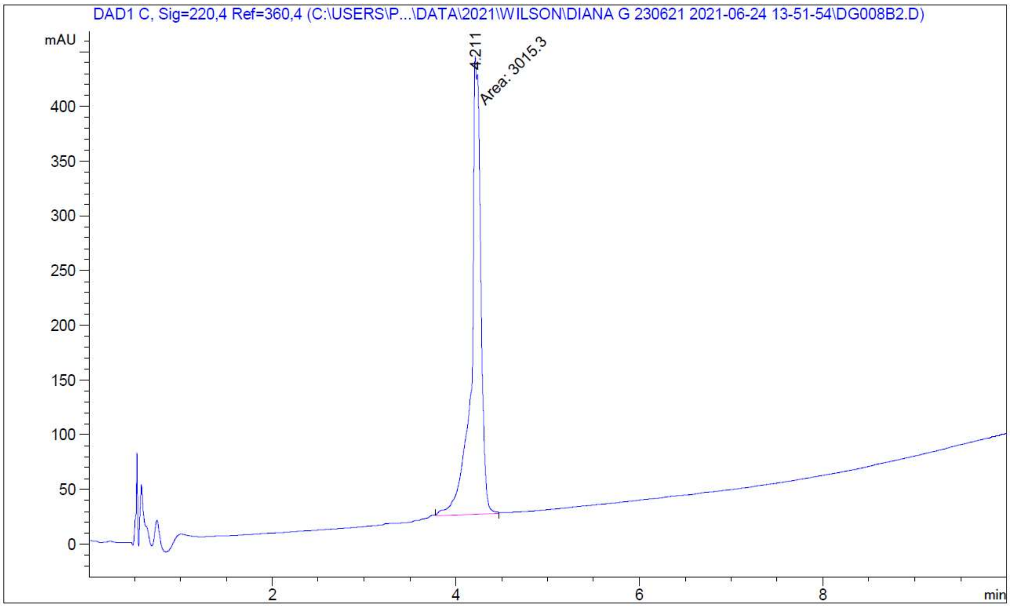

Analytical HPLC trace at λ= 220 nm of purified **TACC3 522-536 E522C/P528C Bph**

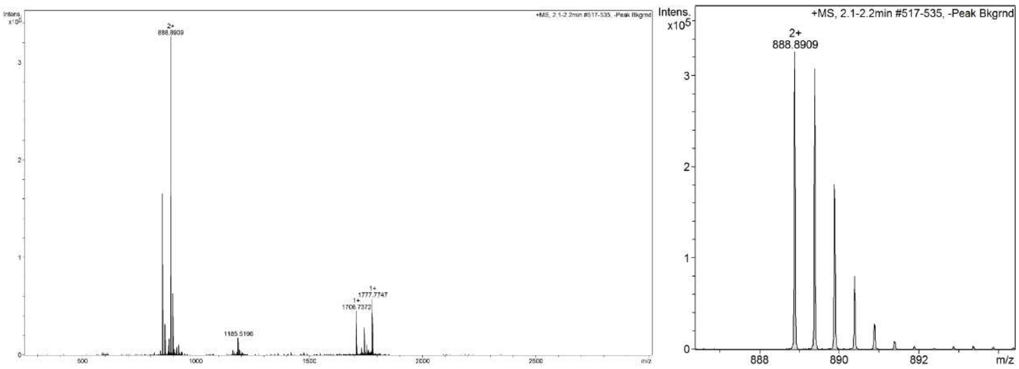

HR-QToF(ESI+)MS analysis of purified **TACC3 522-536 E522C/P528C Bph**

TACC3 **522-536 A529C/G535C Bph**

Sequence:

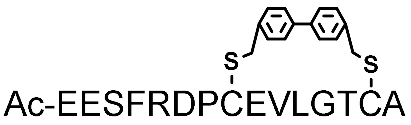

HPLC (λ = 220 nm) tR = 3.648min. HR-QToF (ESI) m/z: [M+2H]2+ Calc. for $C_{84}H_{119}N_{19}O_{26}S_2$: 938.4097; Found: 938.4107.

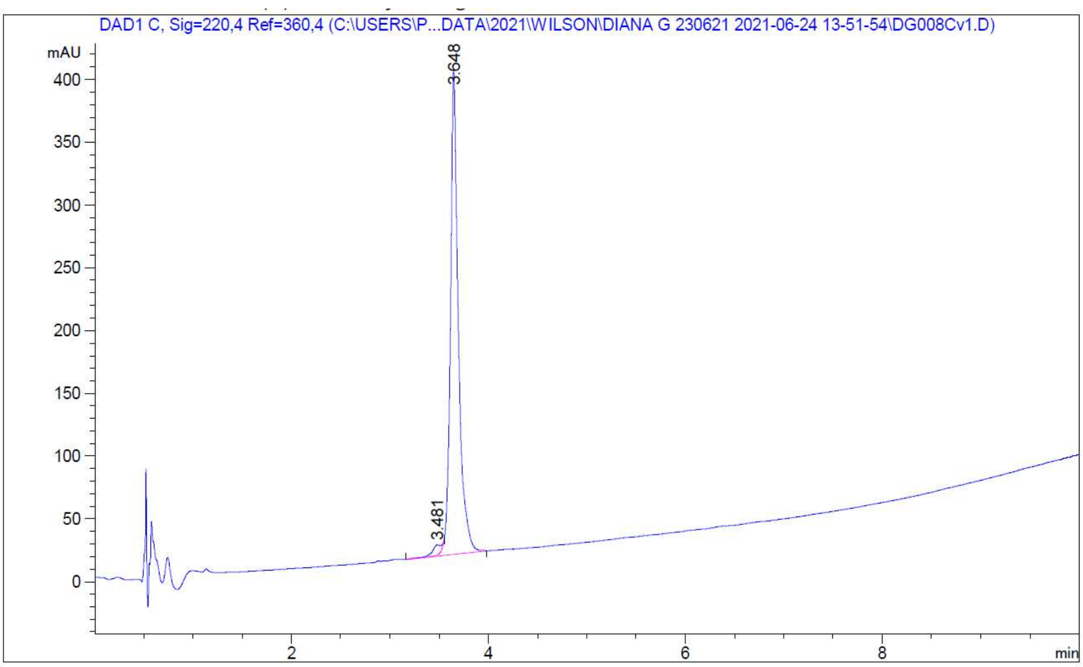

Analytical HPLC trace at λ= 220 nm of purified **TACC3** **522-536 A529C/G535C Bph**

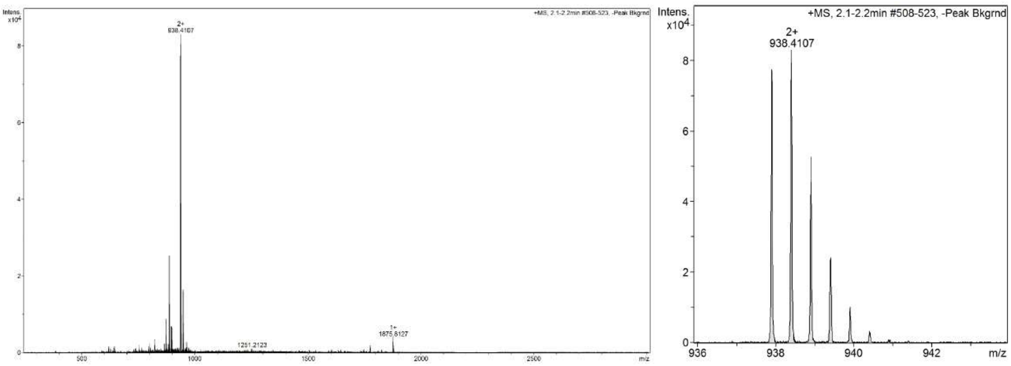

HR-QToF(ESI+)MS analysis of purified **TACC3** **522-536 A529C/G535C Bph**

TACC3 $_{518\text{-}532}$

Sequence: Ac- LELKEESFRDPAEVL

HPLC (λ = 220 nm) tR = 3.657min. HR-QToF (ESI) m/z: [M+2H]2+ Calc. for $C_{81}H_{130}N_{20}O_{27}$: 908.4780; Found: 908.4679.

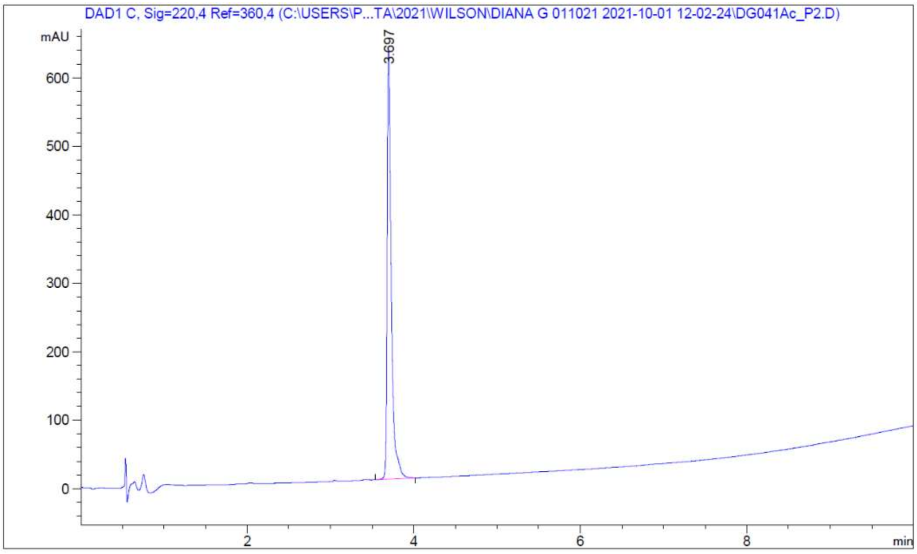

Analytical HPLC trace at λ= 220 nm of purified peptide **TACC3 $_{518\text{-}532}$**

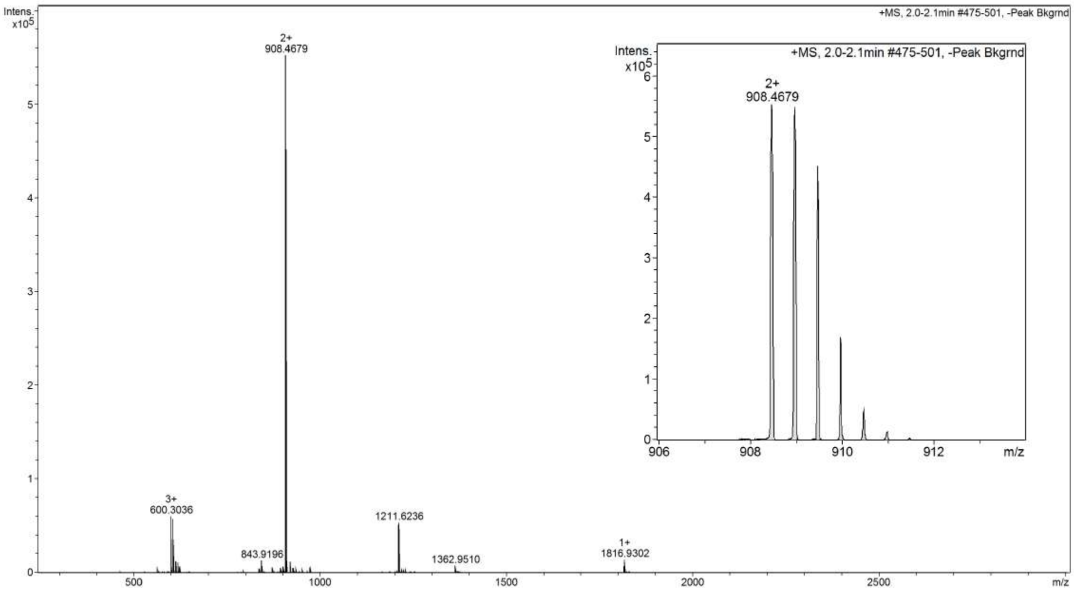

HR-QToF(ESI+)MS analysis of purified peptide **TACC3 $_{518\text{-}532}$**

TACC3 $_{518\text{-}532\ S524C/E530C}$

Sequence: Ac- LELKEECFRDPACVL

HPLC (λ = 220 nm) tR = 4.999 min. HR-QToF (ESI) m/z: [M+2H]2+ Calc. for $\mathbf{C_{79}H_{128}N_{20}O_{24}S_2}$: 903.4499; Found: 903.4446.

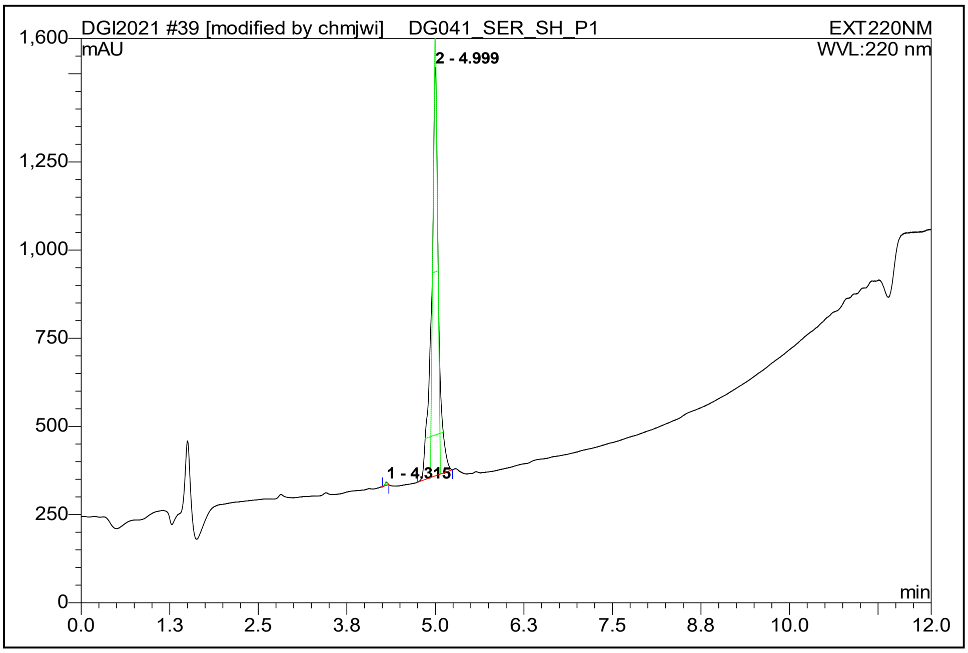

Analytical HPLC trace at λ= 220 nm of purified peptide **TACC3 $_{518\text{-}532\ S524C/E530C}$**

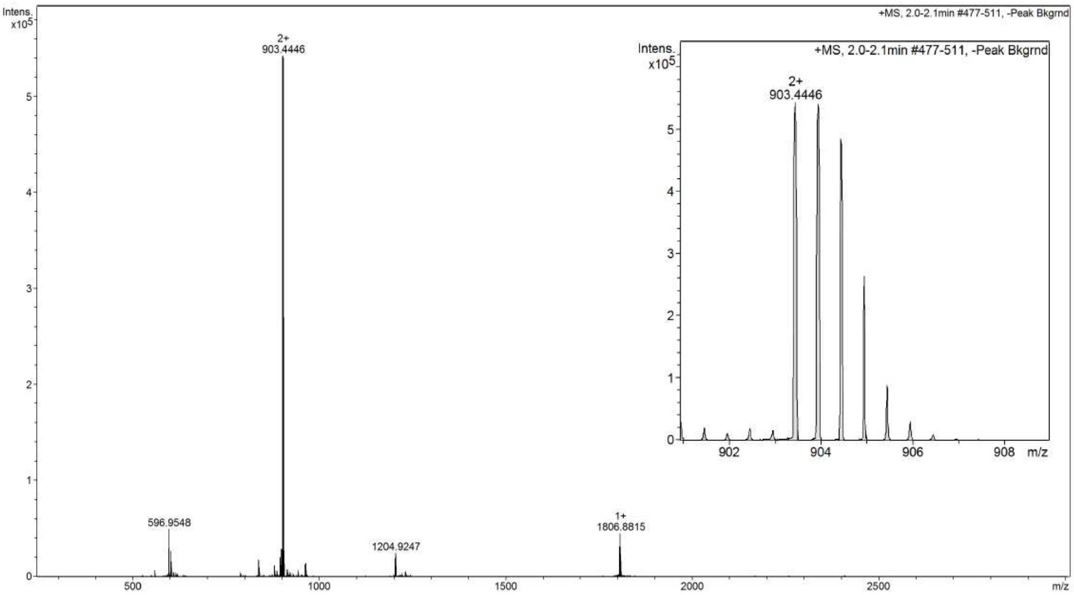

HR-QToF(ESI+)MS analysis of purified peptide **TACC3 $_{518\text{-}532\ S524C/E530C}$**

TACC3 $_{518\text{-}532\ K521C/D527C}$

Sequence: Ac- LELCEESFRCPAEVL

HPLC (λ = 220 nm) tR = 5.436 min. HR-QToF (ESI) m/z: [M+2H]2+ Calc. for $\mathbf{C_{77}H_{123}N_{19}O_{25}S_2}$: 889.9262; Found: 889.9194.

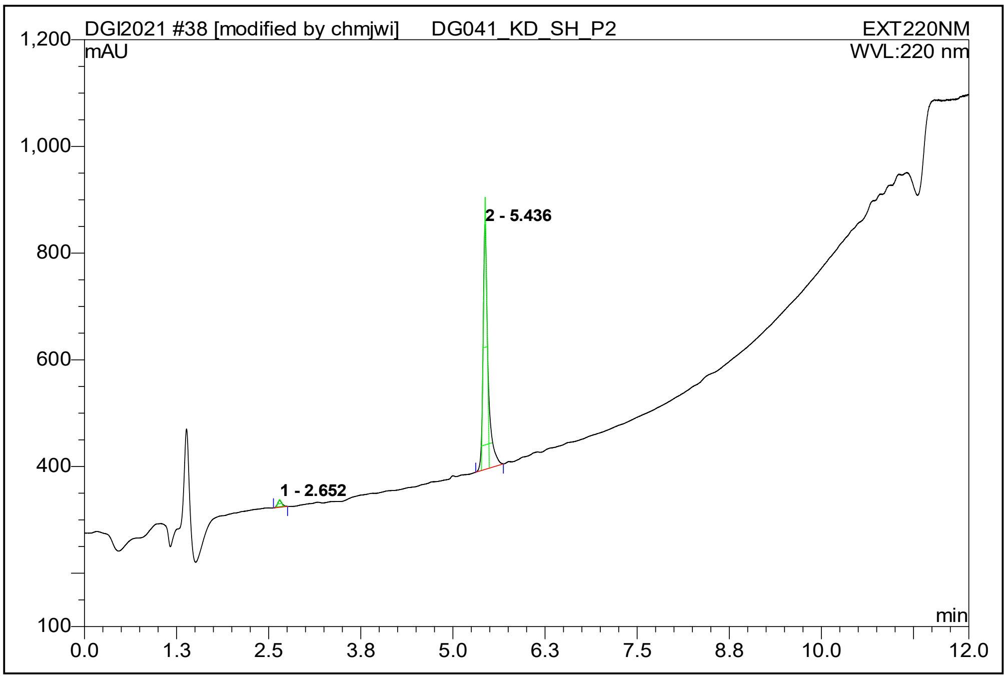

Analytical HPLC trace at λ= 220 nm of purified peptide **TACC3** $_{\mathbf{518\text{-}532\ K521C/D527C}}$

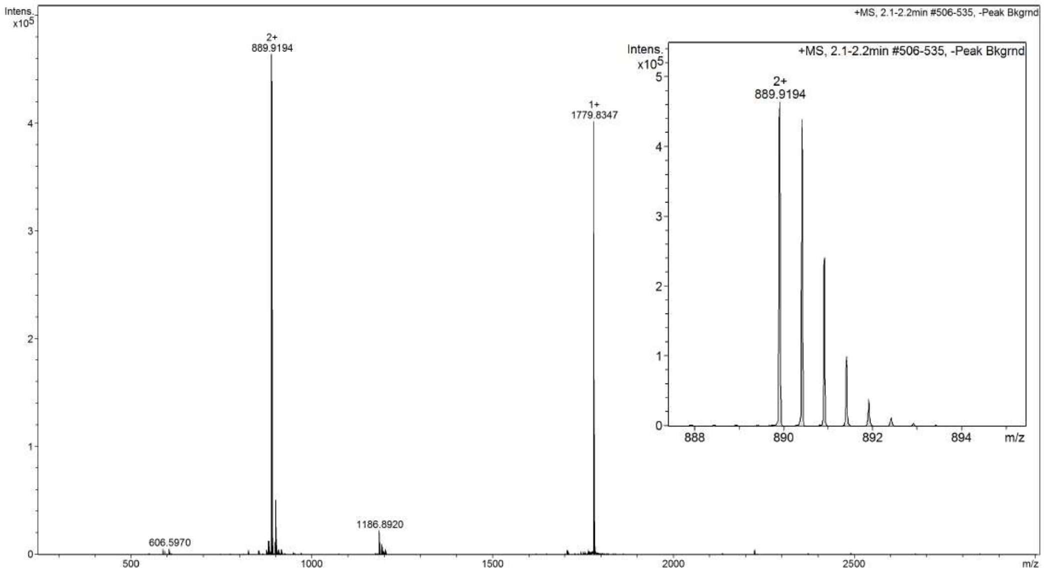

HR-QToF(ESI+)MS analysis of purified peptide **TACC3** $_{\mathbf{518\text{-}532\ K521C/D527C}}$

TACC3 $_{518\text{-}532\ L520C/R526C}$

Sequence: Ac- LECKEESFCDPAEVL

HPLC (λ = 220 nm) tR = 4.640 min. HR-QToF (ESI) m/z: [M+H]+ Calc. for $\mathbf{C_{75}H_{117}N_{17}O_{27}S_2}$: 1753.7844; Found: 1753.7757.

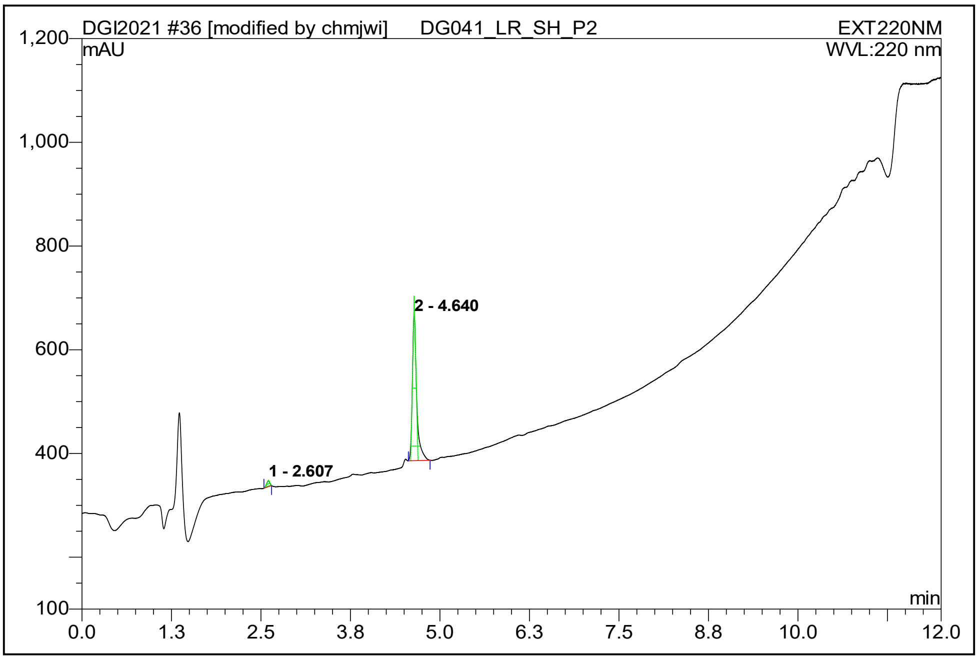

Analytical HPLC trace at λ= 220 nm of purified peptide **TACC3 $_{518\text{-}532\ L520C/R526C}$**

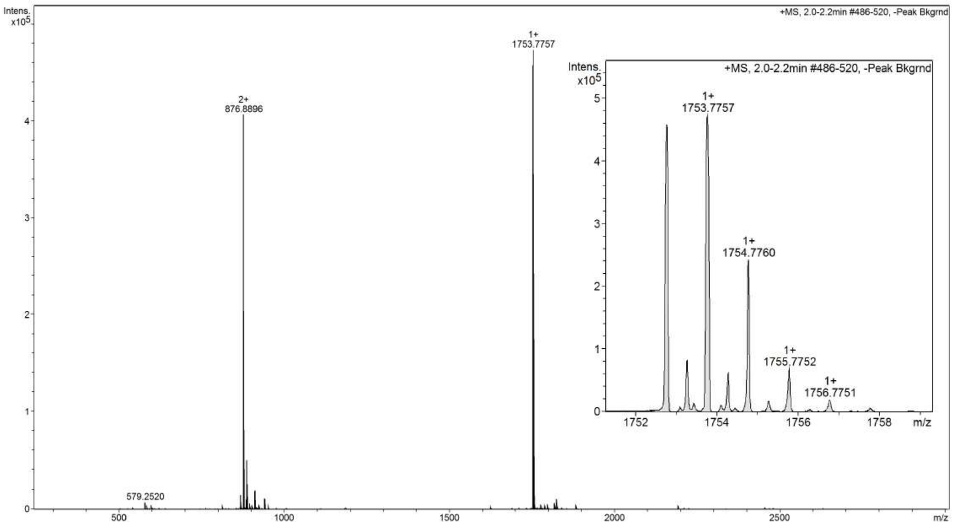

HR-QToF(ESI+)MS analysis of purified peptide **TACC3 $_{518\text{-}532\ L520C/R526C}$**

TACC3 518-532 S524C/E530C Bph

Sequence: 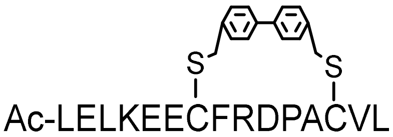

HPLC (λ = 220 nm) tR = 5.383 min. HR-QToF (ESI) m/z: [M+2H]2+ Calc. for $\mathbf{C_{93}H_{138}N_{20}O_{24}S_{2}}$: 992.9902; Found: 992.9850.

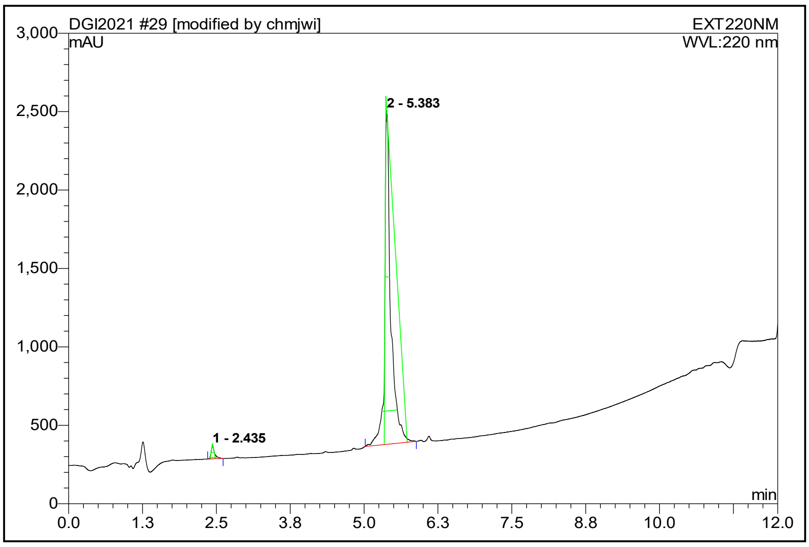

Analytical HPLC trace at λ= 220 nm of purified peptide **TACC3 518-532 S524C/E530C Bph**

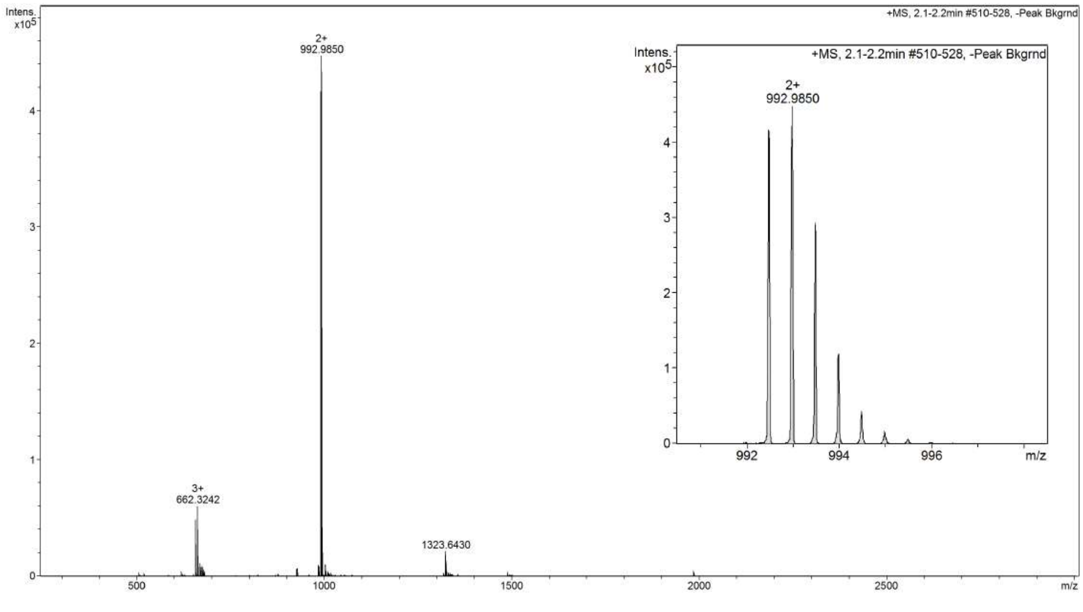

HR-QToF(ESI+)MS analysis of purified peptide **TACC3 518-532 S524C/E530C Bph**

TACC3 **518-532 K521C/D527C Bph**

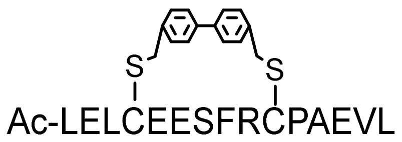

Sequence: Ac-LELCEESFRCPAEVL

HPLC (λ = 220 nm) tR = 5.436 min. HR-QToF (ESI) m/z: [M+2H]2+ Calc. for $\mathbf{C_{91}H_{133}N_{19}O_{25}S_2}$: 979.4665; Found: 979.4629.

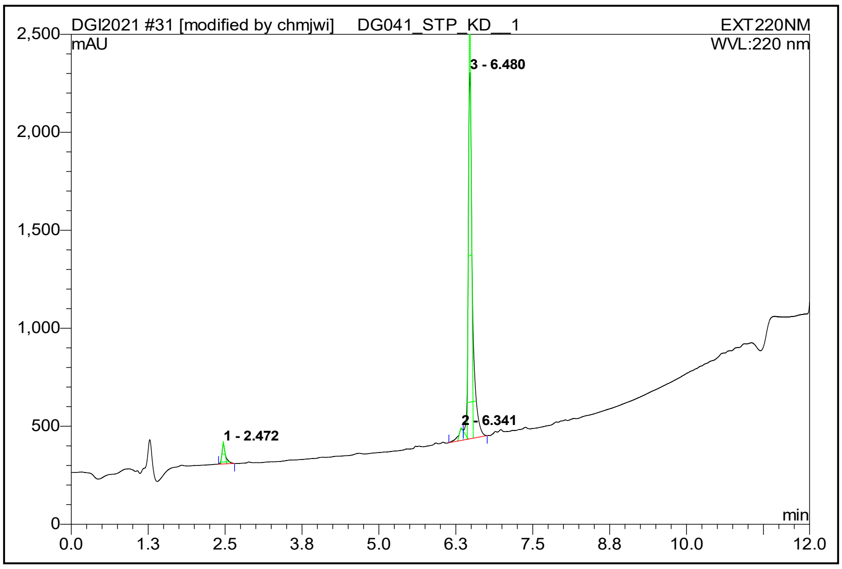

Analytical HPLC trace at λ= 220 nm of purified peptide **TACC3** **518-532 K521C/D527C Bph**

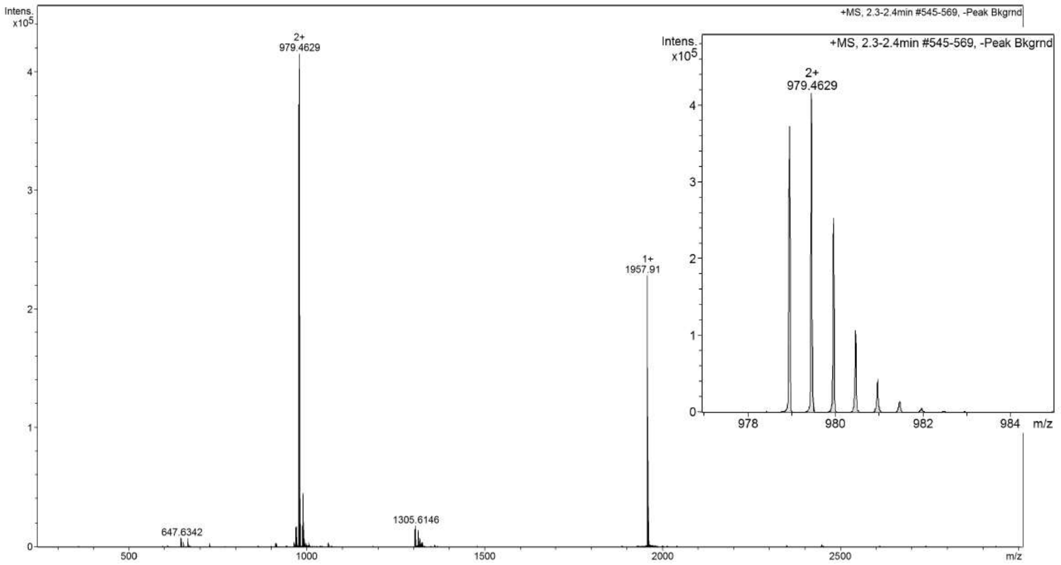

HR-QToF(ESI+)MS analysis of purified peptide **TACC3** **518-532 K521C/D527C Bph**

TACC3 **518-532 L520C/R526C Bph**

Sequence: 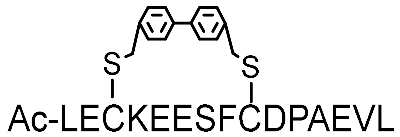

HPLC (λ = 220 nm) tR = 4.791 min. HR-QToF (ESI) m/z: [M+2H]2+ Calc. for $\mathbf{C_{89}H_{127}N_{17}O_{27}S_2}$: 966.4347; Found: 966.4299.

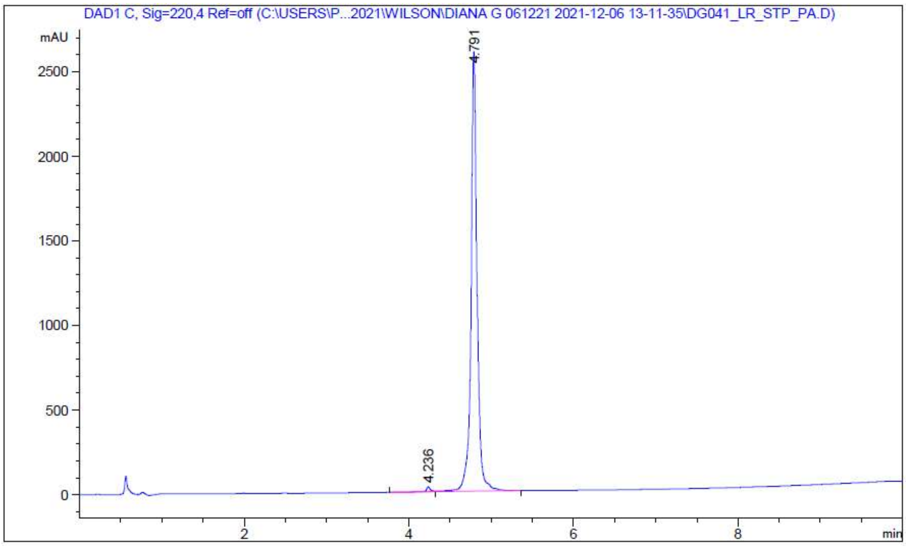

Analytical HPLC trace at λ= 220 nm of purified peptide **TACC3** **518-532 L520C/R526C Bph**

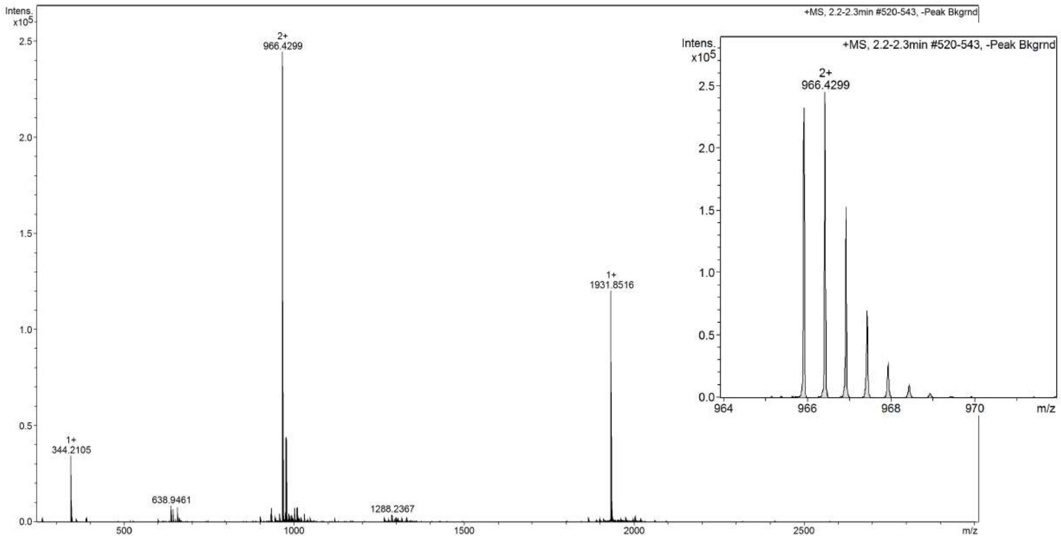

HR-QToF(ESI+)MS analysis of purified peptide **TACC3** **518-532 L520C/R526C Bph**

TACCC3

TACC3 $_{518\text{-}532\ S524C/E530C\ Oct}$

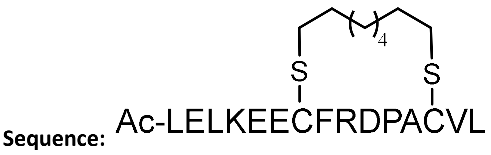

HPLC (λ = 220 nm) tR = 4.714 min. HR-QToF (ESI) m/z: [M+2H]2+ Calc. for $\mathbf{C_{87}H_{142}N_{20}O_{24}S_2}$: 959.0056; Found: 959.0075.

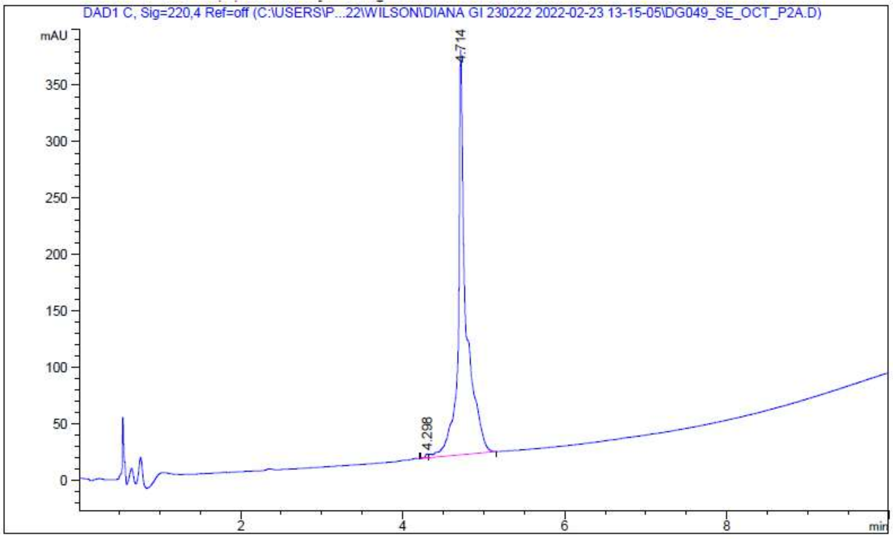

Analytical HPLC trace at λ= 220 nm of purified peptide **TACC3** $_{518\text{-}532\ S524C/E530C\ Oct}$

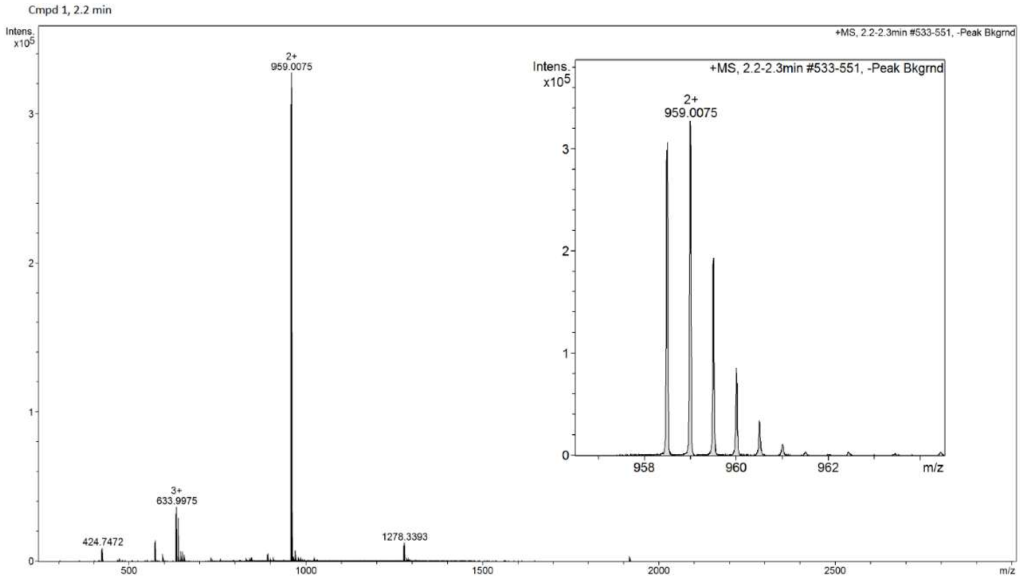

HR-QToF(ESI+)MS analysis of purified peptide **TACC3** $_{518\text{-}532\ S524C/E530C\ Oct}$

TACC3 $_{518\text{-}532\ L520C/R526C\ Oct}$

Sequence:

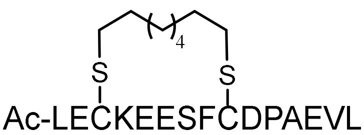

HPLC (λ = 220 nm) tR = 4.086 min. HR-QToF (ESI) m/z: [M+2H]2+ Calc. for $\mathbf{C_{83}H_{131}N_{17}O_{27}S_2}$: 932.4643; Found: 932.4523.

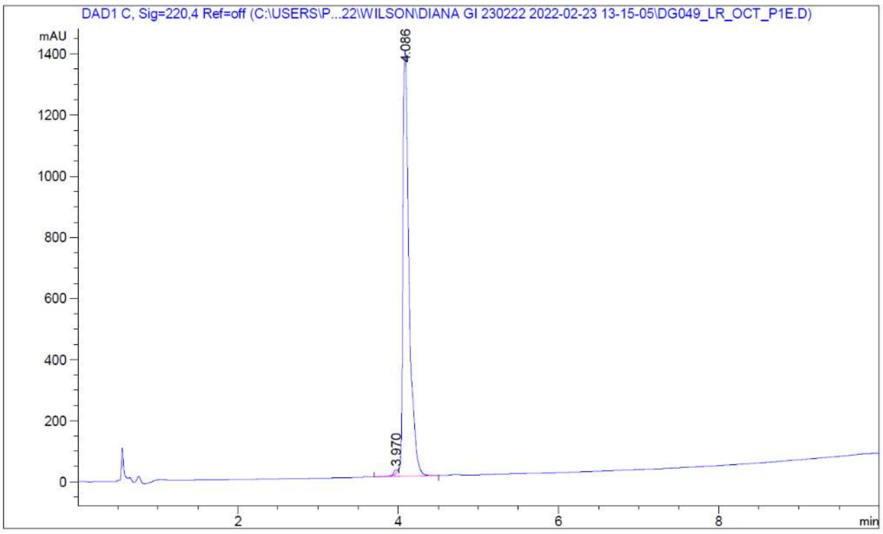

Analytical HPLC trace at λ= 220 nm of purified peptide **TACC3 $_{518\text{-}532\ L520C/R526C\ Oct}$**

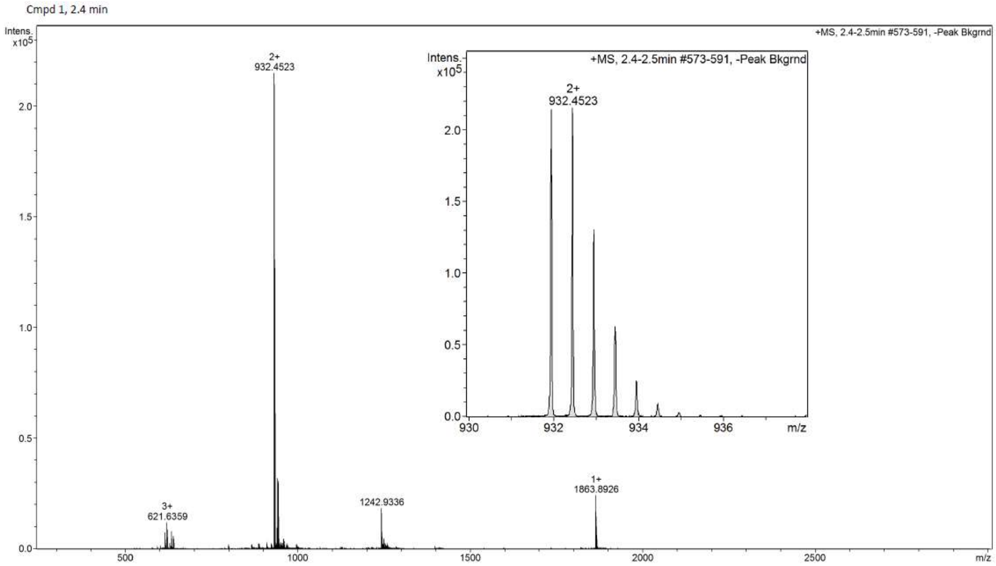

HR-QToF(ESI+)MS analysis of purified peptide **TACC3 $_{518\text{-}532\ L520C/R526C\ Oct}$**

TACC3 $_{518\text{-}532\ S524C/E530C\ PEG}$

Sequence:

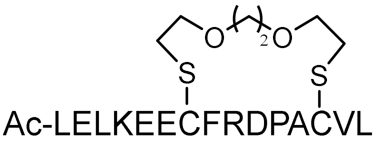

HPLC (λ = 220 nm) tR = 4.136 min. HR-QToF (ESI) m/z: [M+2H]2+ Calc. for **$C_{85}H_{138}N_{20}O_{26}S_{2}$**: 960.9840; Found: 960.9884.

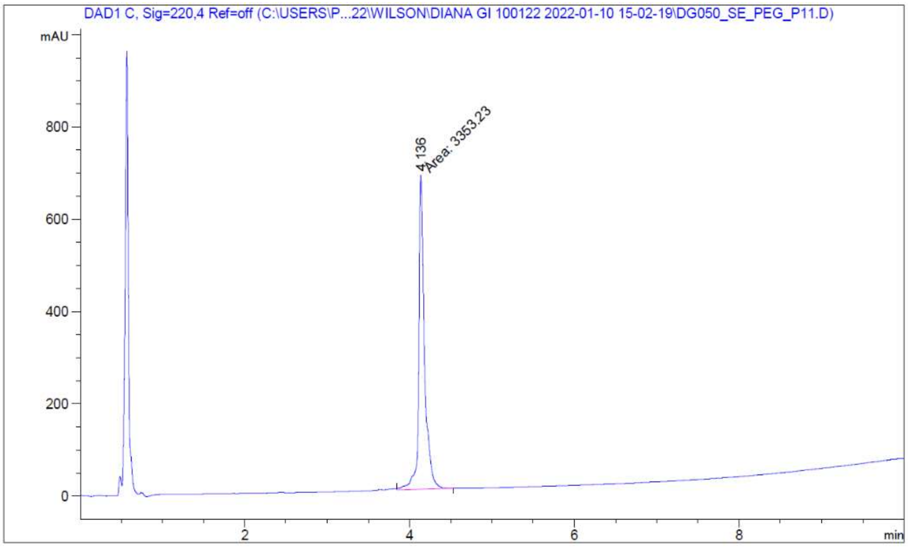

Analytical HPLC trace at λ= 220 nm of purified peptide **TACC3 $_{518\text{-}532\ S524C/E530C\ PEG}$**

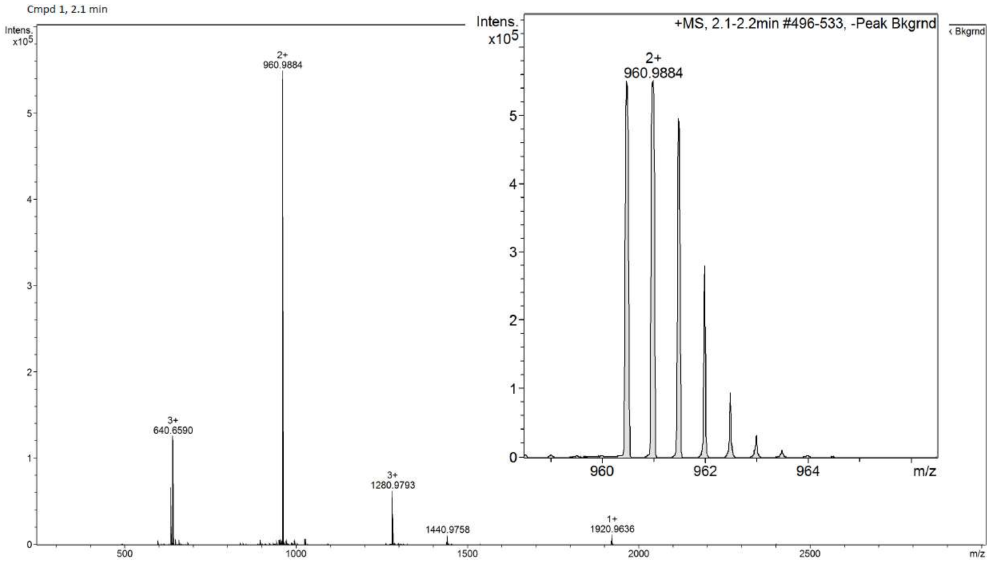

HR-QToF(ESI+)MS analysis of purified **TACC3 $_{518\text{-}532\ S524C/E530C\ PEG}$**

TACC3 $_{518\text{-}532\ L520C/R526C\ PEG}$

Sequence: 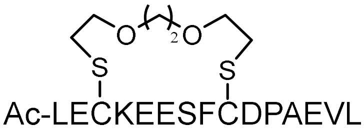

HPLC (λ = 220 nm) tR = 3.937 min. HR-QToF (ESI) m/z: [M+2H]2+ Calc. for $\mathbf{C_{81}H_{127}N_{17}O_{29}S_2}$: 933.9296; Found: 933.9295.

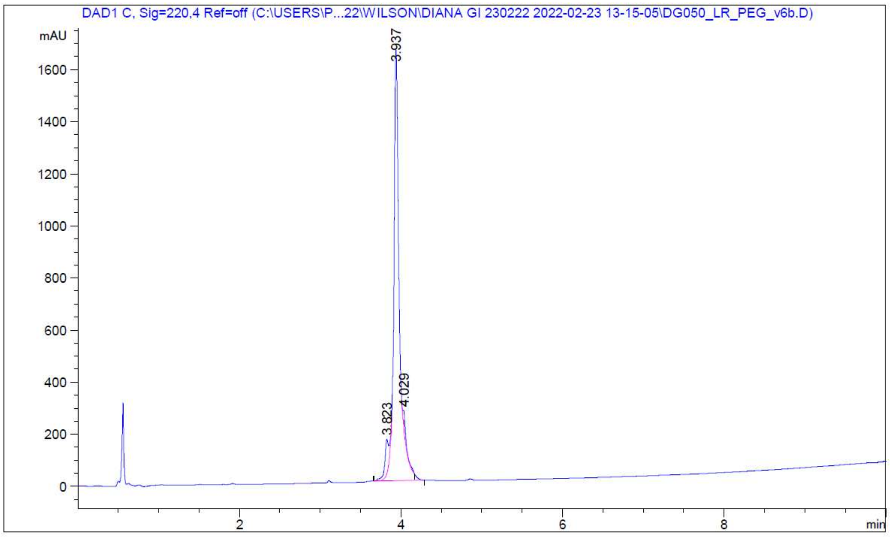

Analytical HPLC trace at λ= 220 nm of purified peptide **TACC3 $_{518\text{-}532\ L520C/R526C\ PEG}$**

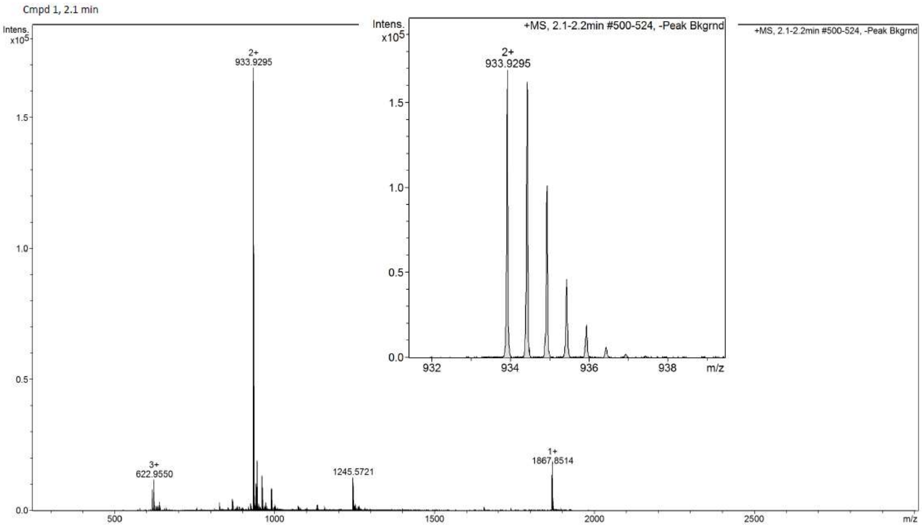

HR-QToF(ESI+)MS analysis of purified peptide **TACC3 $_{518\text{-}532\ L520C/R526C\ PEG}$**

TACC3 **518-532 S524C/E530C Bph (4-I)525F**

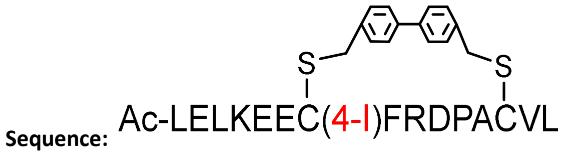

HPLC (λ = 220 nm) tR = 4.931 min. HR-QToF (ESI) m/z: [M+2H]2+ Calc. for $\mathbf{C_{93}H_{137}IN_{20}O_{24}S_2}$: 960.9840; Found: 960.9884.

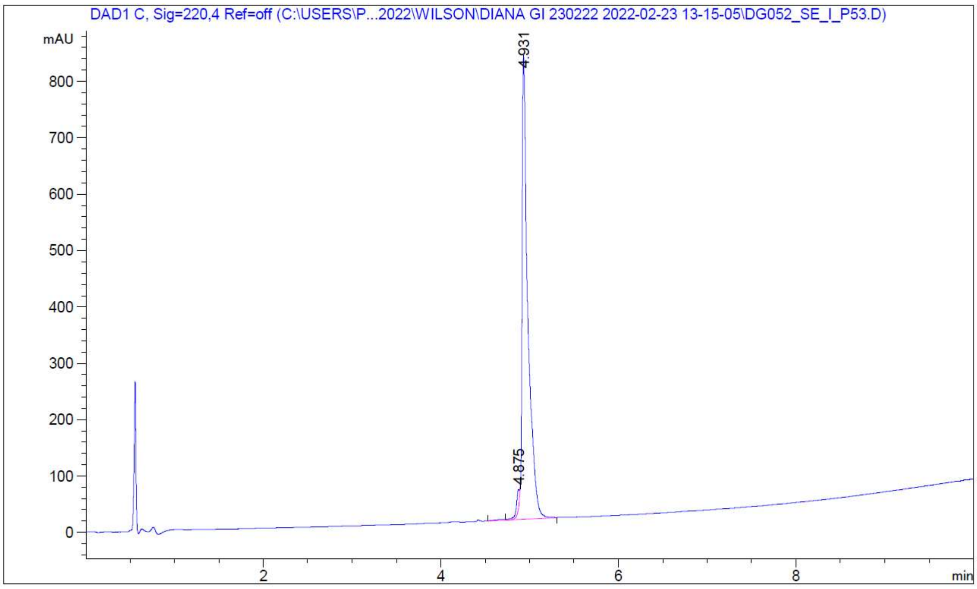

Analytical HPLC trace at λ= 220 nm of purified **TACC3** **518-532 S524C/E530C Bph (4-I)525F**

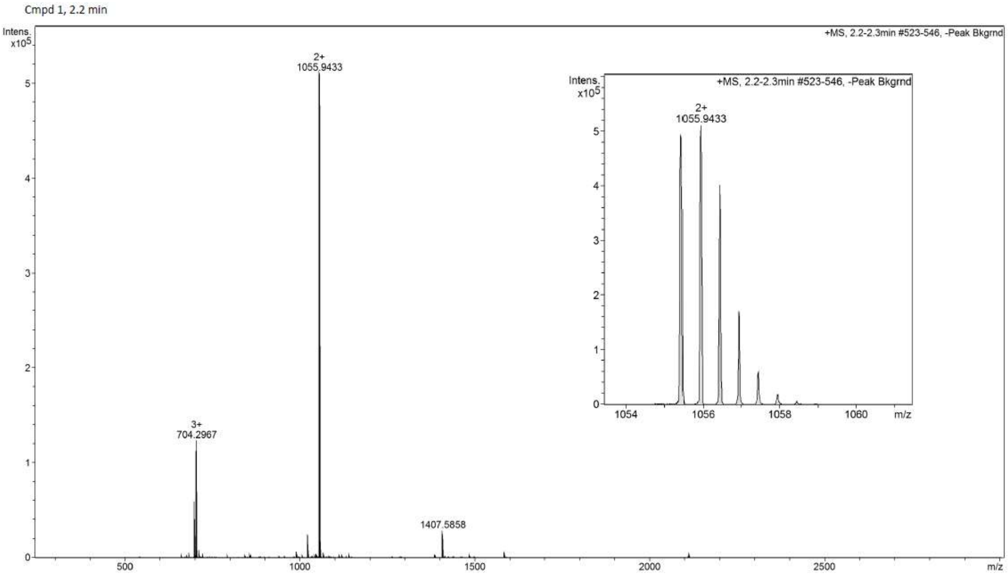

HR-QToF(ESI+)MS analysis of purified **TACC3** **518-532 S524C/E530C Bph (4-I)525F**

TACC3 518-532 L520C/R526C Bph (4-I)525F

Sequence:

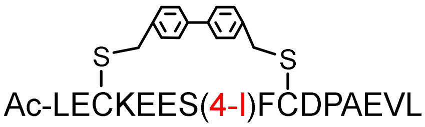

HPLC (λ = 220 nm) tR = 5.024 min. HR-QToF (ESI) m/z: [M+2H]2+ Calc. for $\mathbf{C_{89}H_{126}IN_{17}O_{27}S_2}$: 1029.3829; Found: 1029.3854.

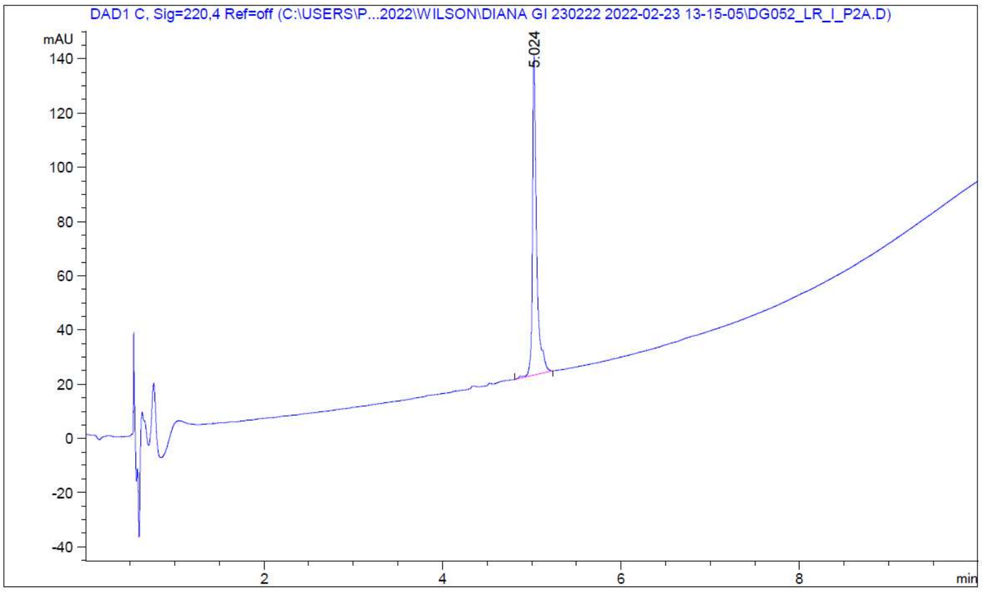

Analytical HPLC trace at λ= 220 nm of purified **TACC3 518-532 L520C/R526C Bph (4-I)525F**

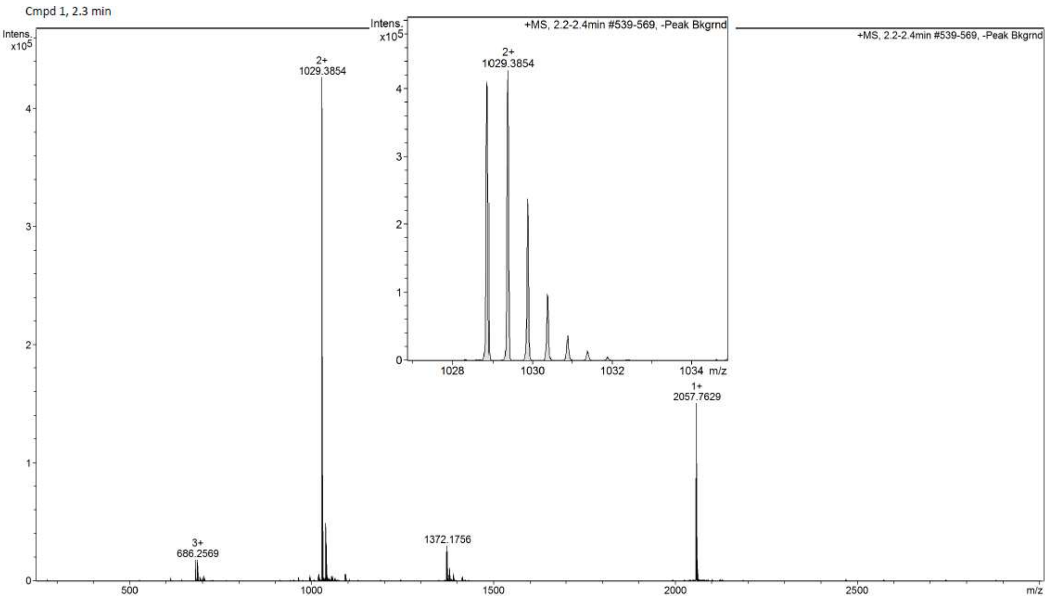

HR-QToF(ESI+)MS analysis of purified peptide **TACC3 518-532 L520C/R526C Bph (4-I)525F**

TACC3 **518-532 L520C/R526C Bph (4-Br)525F**

Sequence: 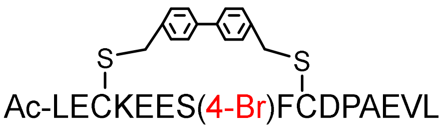

HPLC (λ = 220 nm) tR = 4.951 min. HR-QToF (ESI) m/z: [M+2H]2+ Calc. for $\mathbf{C_{89}H_{126}BrN_{17}O_{27}S_2}$: 1005.8893; Found: 1005.8922.

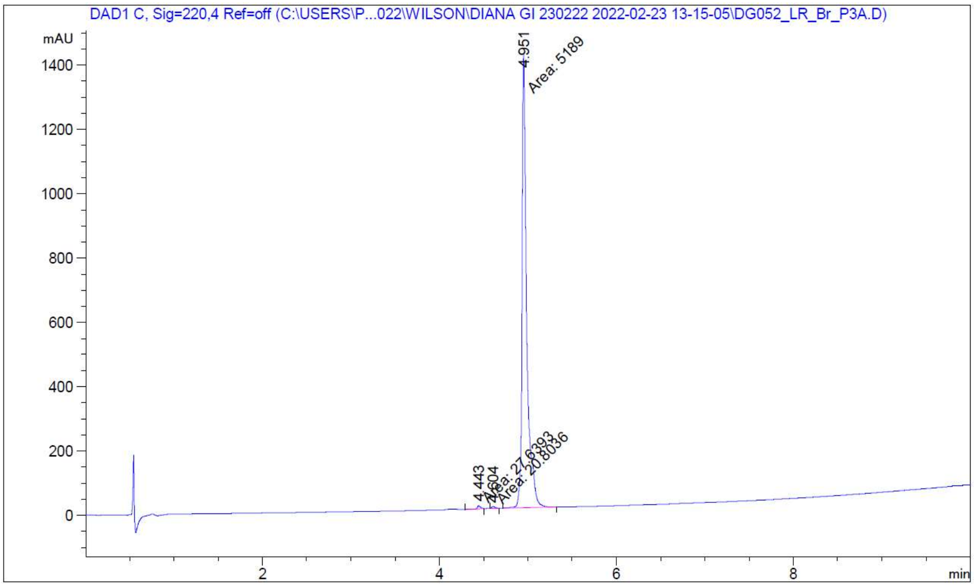

Analytical HPLC trace at λ= 220 nm of purified peptide **TACC3** **518-532 L520C/R526C Bph (4-Br)525F**

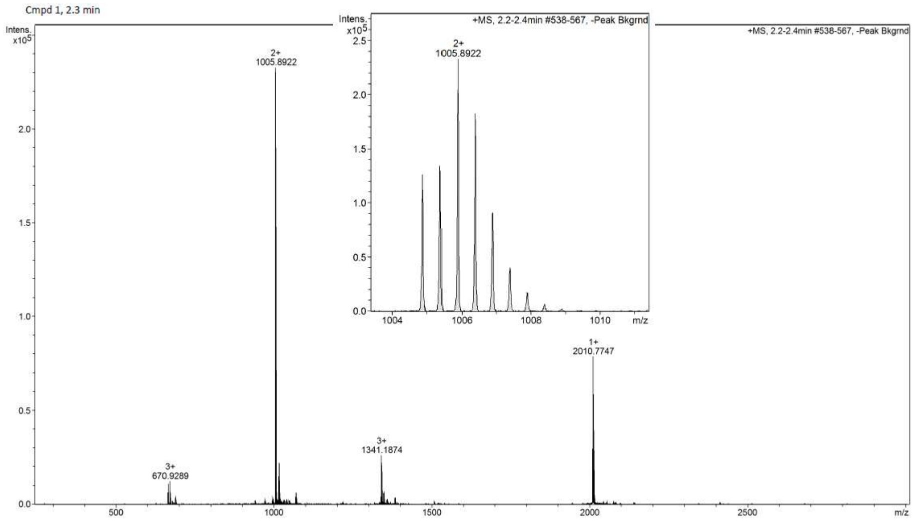

HR-QToF(ESI+)MS analysis of purified peptide **TACC3** **518-532 L520C/R526C Bph (4-Br)525F**

TACC3 **518-532 S524C/E530C Bph (4-Br)525F**

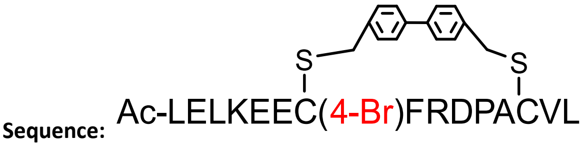

Sequence:

HPLC (λ = 220 nm) tR = 7.751 min. HR-QToF (ESI) m/z: [M+2H]2+ Calc. for **$C_{93}H_{137}BrN_{20}O_{24}S_2$**: 1032.4445; Found: 1032.4981.

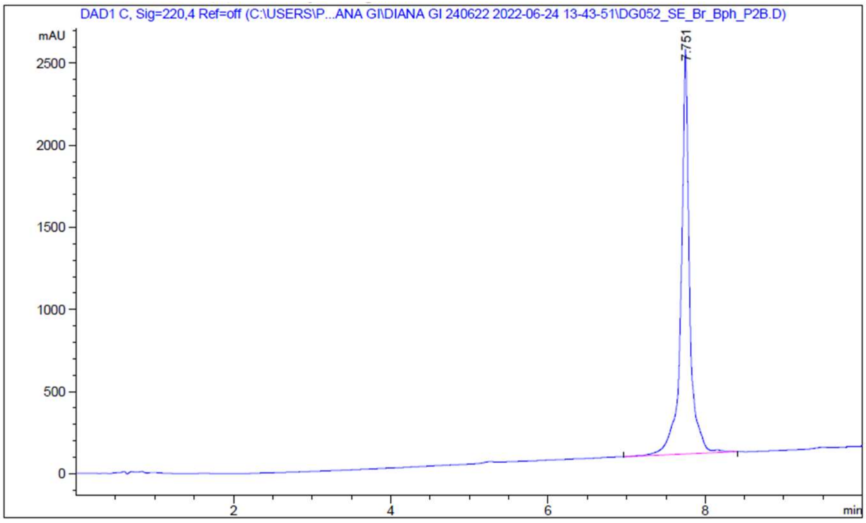

Analytical HPLC trace at λ= 220 nm of purified **TACC3** **518-532 S524C/E530C Bph (4-Br)525F**

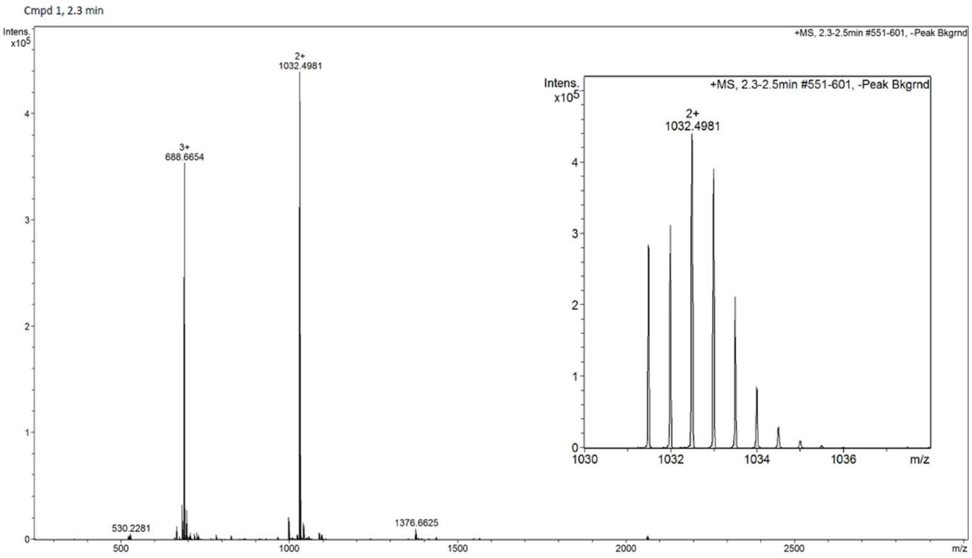

HR-QToF(ESI+)MS analysis of purified **TACC3** **518-532 S524C/E530C Bph (4-Br)525F**

TACC3 **518-532 S524C/E530C Bph (4-I)525F/(4-F)528P**

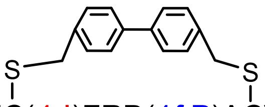

Sequence: Ac-LELKEEC(4-I)FRD(4*f*-P)ACVL

HPLC (λ = 220 nm) tR = 4.882 min. HR-QToF (ESI) m/z: [M+2H]2+ Calc. for $\mathbf{C_{93}H_{136}FIN_{20}O_{24}S_2}$: 1064.9338; Found: 1064.9368.

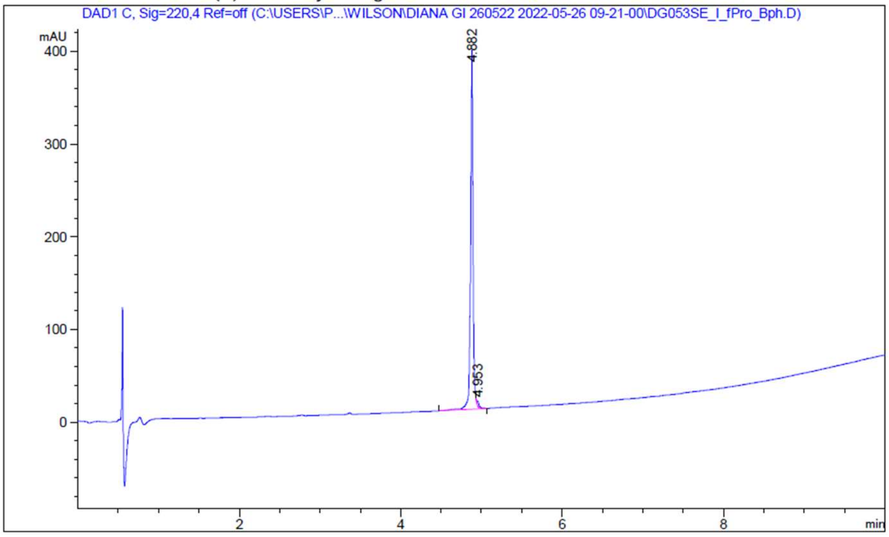

Analytical HPLC trace at λ= 220 nm of purified **TACC3** **518-532 S524C/E530C Bph (4-I)525F/(4-F)528P**

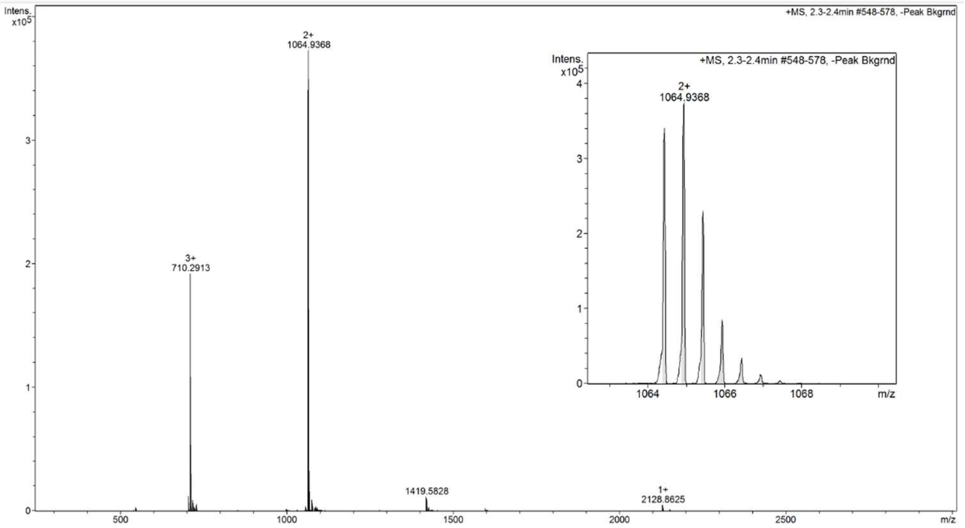

HR-QToF(ESI+)MS analysis of purified **TACC3** **518-532 S524C/E530C Bph (4-I)525F/(4-F)528P**

TACC3 **518-532 S524C/E530C Bph (4-Br)525F/(4-F)528P**

Sequence:

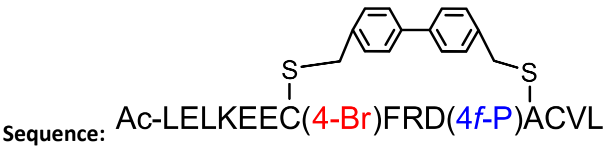

HPLC (λ = 220 nm) tR = 4.882 min. HR-QToF (ESI) m/z: [M+2H]2+ Calc. for $\mathbf{C_{93}H_{136}FBrN_{20}O_{24}S_{2}}$: 1041.4398; Found: 1041.4427.

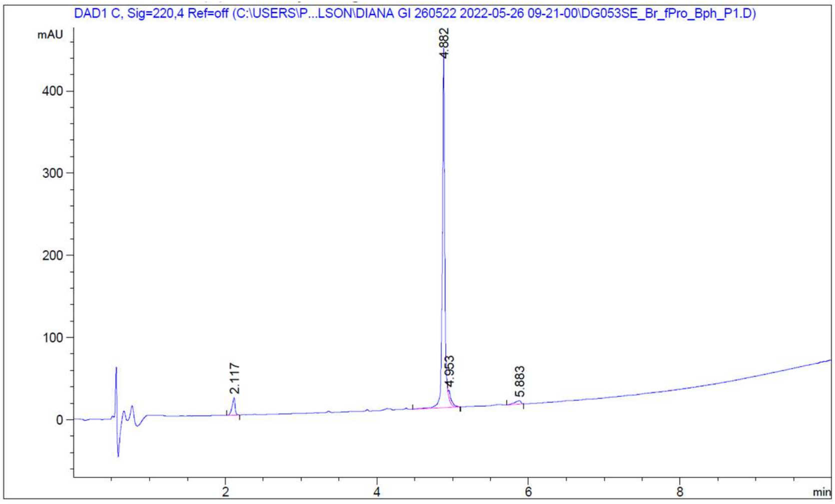

Analytical HPLC trace at λ= 220 nm of purified **TACC3** **518-532 S524C/E530C Bph (4-Br)525F/(4-F)528P**

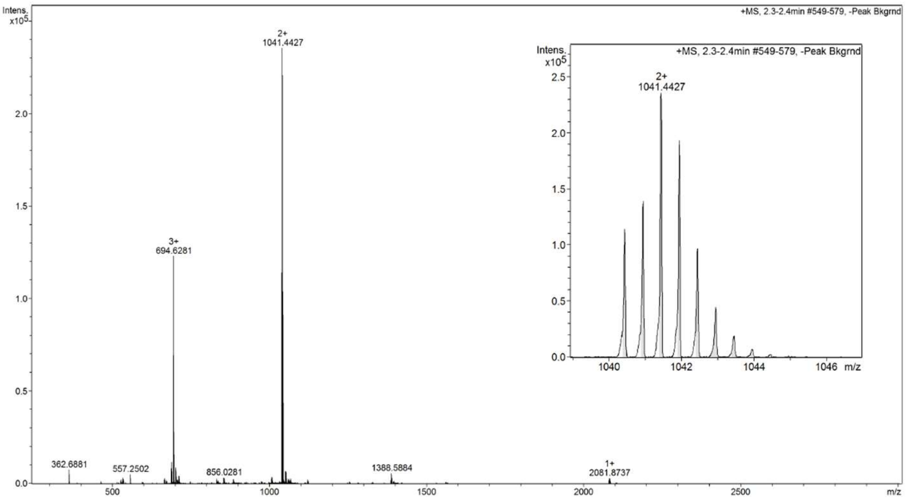

HR-QToF(ESI+)MS analysis of purified **TACC3** **518-532 S524C/E530C Bph (4-Br)525F/(4-F)528P**

TACC3 **518-532 L520C/R526C Bph (4-I)525F/(4-F)528P**

Sequence:

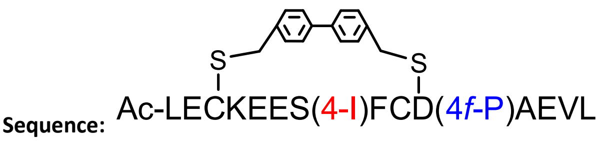

HPLC (λ = 220 nm) tR = 4.979 min. HR-QToF (ESI) m/z: [M+2H]2+ Calc. for $\mathbf{C_{89}H_{125}FIN_{17}O_{27}S_2}$: 1038.3812; Found: 1038.3786.

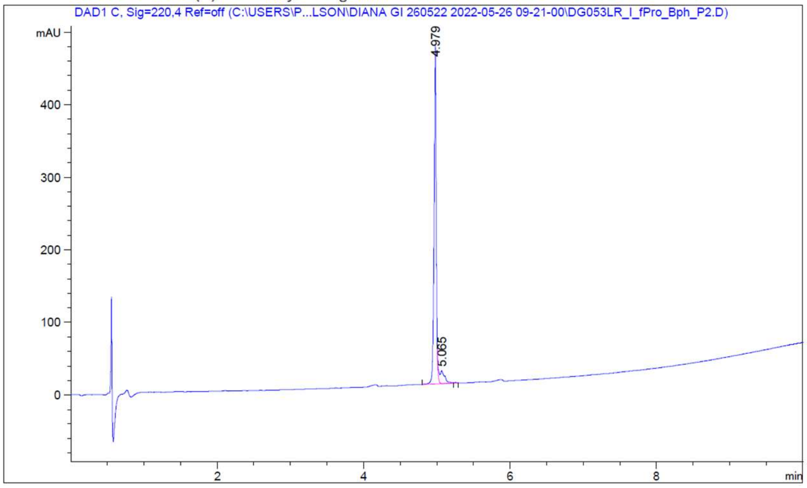

Analytical HPLC trace at λ= 220 nm of purified **TACC3** **518-532 L520C/R526C Bph (4-I)525F/(4-F)528P**

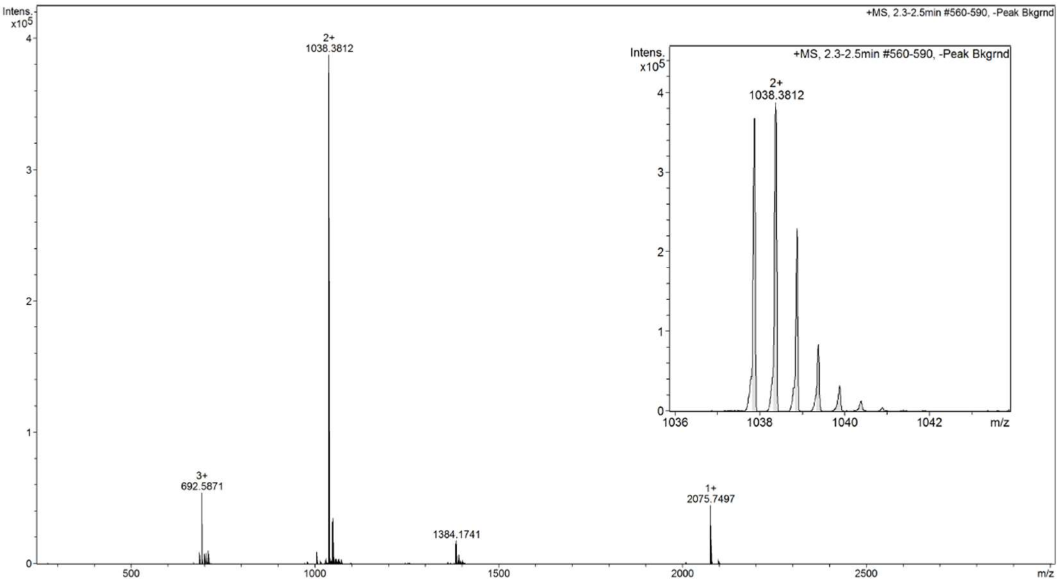

HR-QToF(ESI+)MS analysis of purified **TACC3** **518-532 L520C/R526C Bph (4-I)525F/(4-F)528P**

TACC3 **518-532 L520C/R526C Bph (4-Br)525F/(4-F)528P**

Sequence:

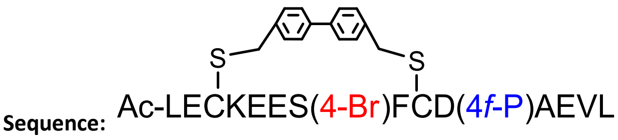

HPLC (λ = 220 nm) tR = 4.882 min. HR-QToF (ESI) m/z: [M+2H]2+ Calc. for $\mathbf{C_{89}H_{125}BrFN_{17}O_{27}S_2}$: 1014.8841; Found: 1014.8872.

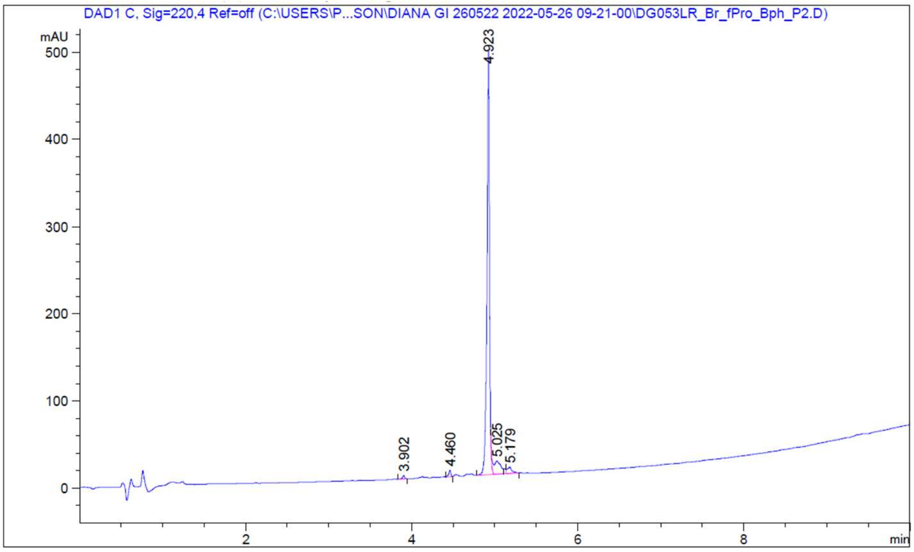

Analytical HPLC trace at λ= 220 nm of purified **TACC3** **518-532 L520C/R526C Bph (4-Br)525F/(4-F)528P**

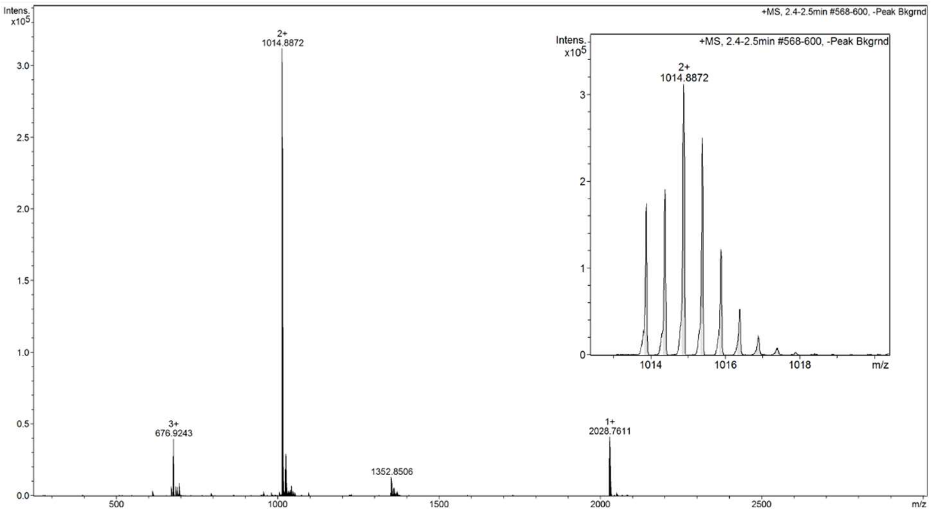

HR-QToF(ESI+)MS analysis of purified **TACC3** **518-532 L520C/R526C Bph (4-Br)525F/(4-F)528P**

TACC3 $_{518\text{-}532\ S524C/E530C\ Bph\ (4\text{-}I)525F/(4\text{-}F)528P}$ Ahx-FAM

Sequence: 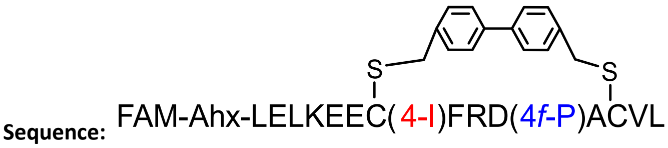

HPLC (λ = 220 nm) tR = 7.955 min. HR-QToF (ESI) m/z: [M+3H]3+ Calc. for **$C_{118}H_{155}FIN_{21}O_{30}S_2$**: 853.3320; Found: 853.3339.

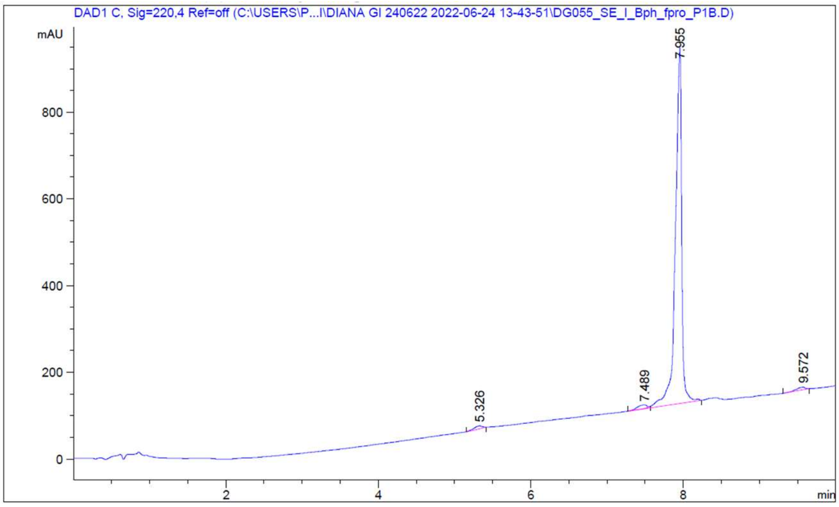

Analytical HPLC trace at λ= 220 nm of purified **TACC3 $_{518\text{-}532\ S524C/E530C\ Bph\ (4\text{-}I)525F/(4\text{-}F)528P}$ Ahx-FAM**

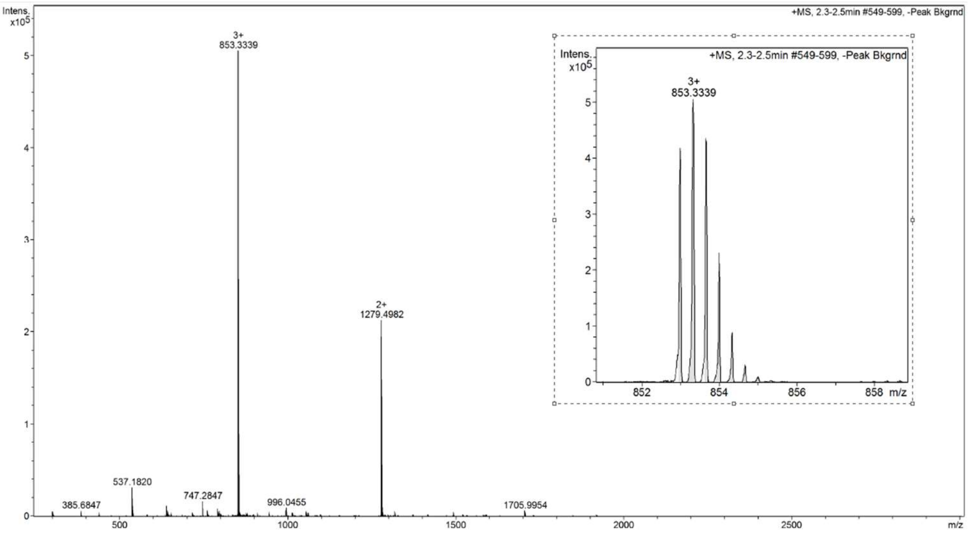

HR-QToF(ESI+)MS analysis of purified **TACC3 $_{518\text{-}532\ S524C/E530C\ Bph\ (4\text{-}I)525F/(4\text{-}F)528P}$ Ahx-FAM**

TACC3 $_{\text{518-532 S524C/E530C Bph (4-Br)525F/(4-F)528P}}$ Ahx-FAM

Sequence:

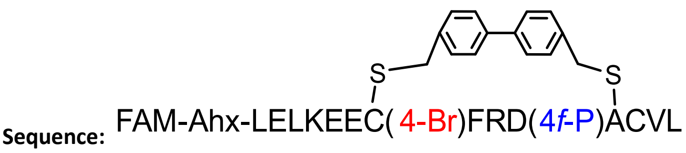

HPLC (λ = 220 nm) tR = 7.906 min. HR-QToF (ESI) m/z: [M+3H]3+ Calc. for **$C_{118}H_{155}BrFN_{21}O_{30}S_{2}$**: 837.6697; Found: 837.6709.

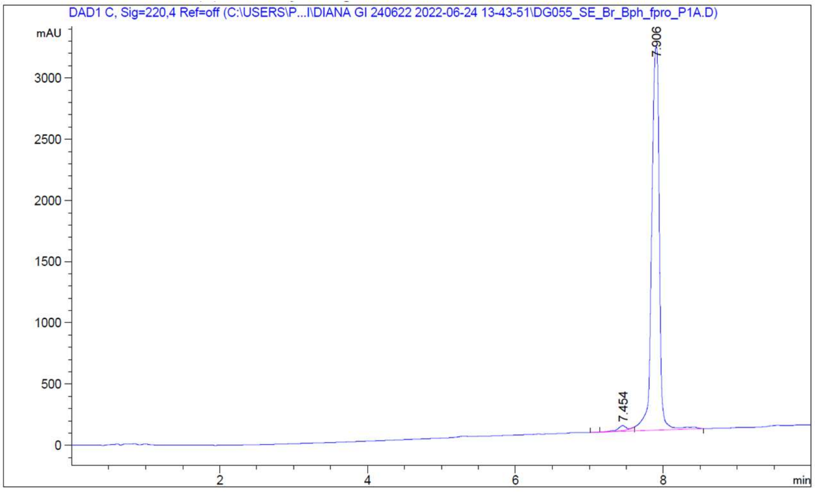

Analytical HPLC trace at λ= 220 nm of purified **TACC3 $_{\text{518-532 S524C/E530C Bph (4-Br)525F/(4-F)528P}}$ Ahx-FAM**

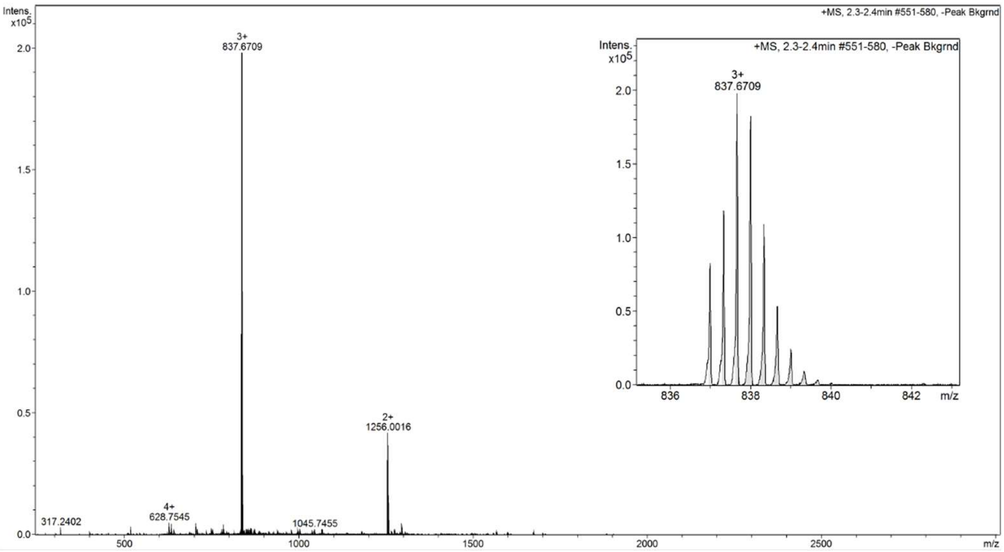

HR-QToF(ESI+)MS analysis of **TACC3 $_{\text{518-532 S524C/E530C Bph (4-Br)525F/(4-F)528P}}$ Ahx-FAM**

TACC3 518-532 S524C/E530C Bph (4-I)525F/(4-F)528P Ahx-FAM

Sequence:

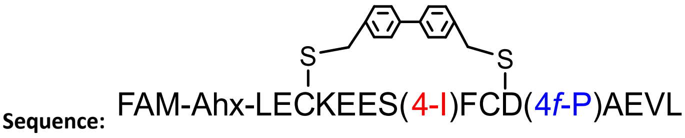

HPLC (λ = 220 nm) tR = 5.671 min. HR-QToF (ESI) m/z: [M+3H]3+ Calc. for **$C_{114}H_{144}FIN_{18}O_{33}S_2$**: 835.6287; Found: 835.6284.

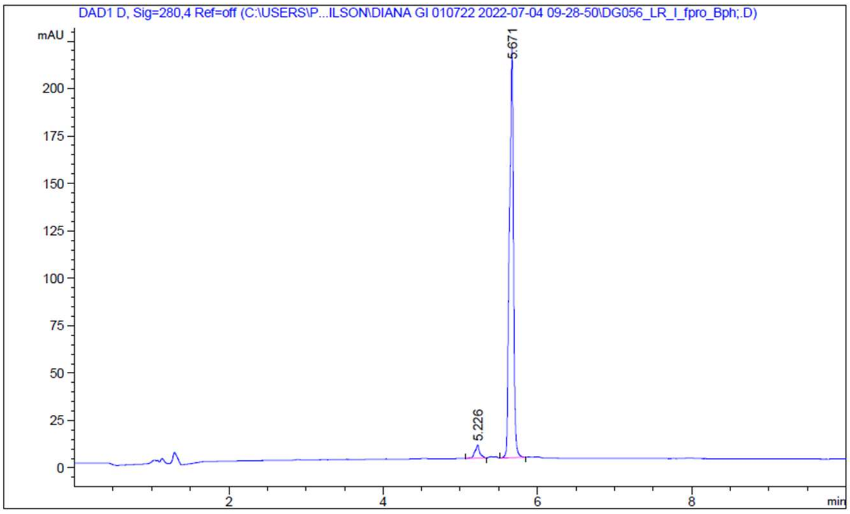

Analytical HPLC trace at λ= 220 nm of purified **TACC3 518-532 S524C/E530C Bph (4-I)525F/(4-F)528P Ahx-FAM**

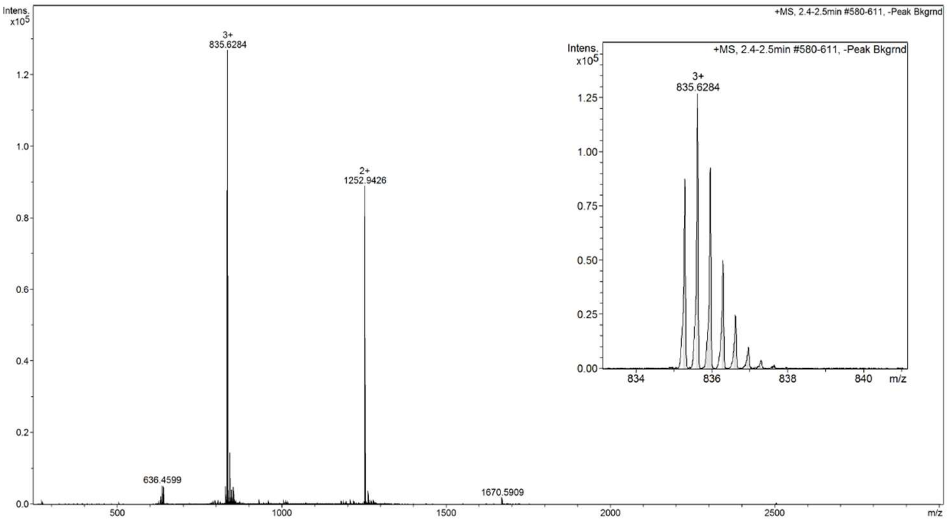

HR-QToF(ESI+)MS analysis of purified **TACC3 518-532 S524C/E530C Bph (4-I)525F/(4-F)528P Ahx-FAM**

TACC3 $_{518\text{-}532\ L520C/R526C\ Bph\ (4\text{-}Br)525F/(4\text{-}F)528P}$ Ahx-FAM

Sequence:

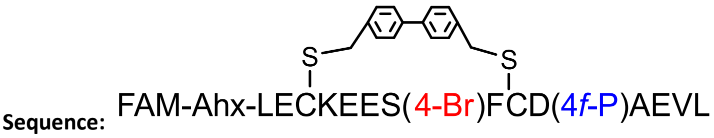

HPLC (λ = 220 nm) tR = 5.958 min. HR-QToF (ESI) m/z: [M+3H]3+ Calc. for **$C_{114}H_{144}BrFN_{18}O_{33}S_2$:** 819.9663; Found: 819.9660.

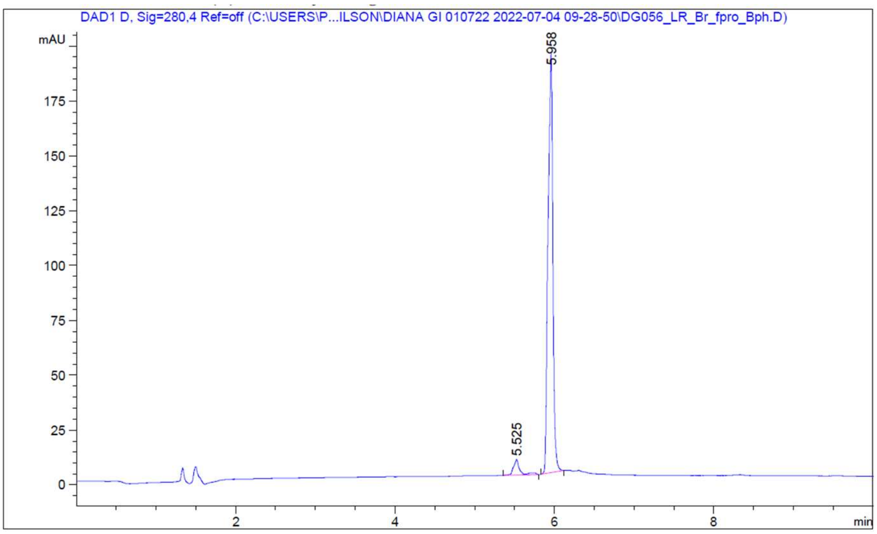

Analytical HPLC trace at λ= 220 nm of purified **TACC3 $_{518\text{-}532\ L520C/R526C\ Bph\ (4\text{-}Br)525F/(4\text{-}F)528P}$ Ahx-FAM**

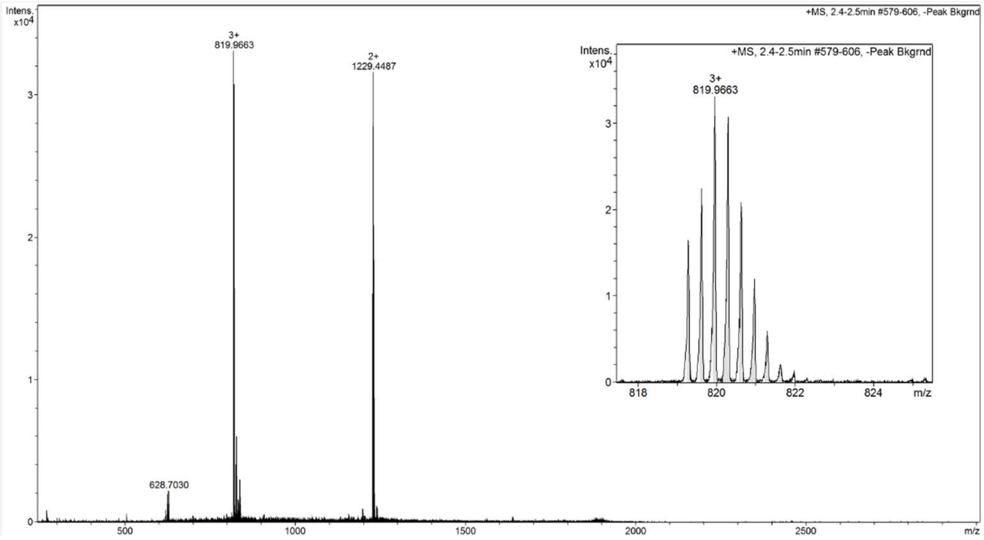

HR-QToF(ESI+)MS analysis of purified **TACC3 $_{518\text{-}532\ L520C/R526C\ Bph\ (4\text{-}Br)525F/(4\text{-}F)528P}$ Ahx-FAM**

N-Myc $_{61\text{-}89}$

Sequence: Ac- LSPSRGFAEHSSEPPSWVTEMLLENELWG

HPLC (λ = 214 nm) tR = 12.06 min. HR-QToF (ESI) m/z: [M+3H]3+ Calc. for $C_{149}H_{220}N_{38}O_{47}S$: 1109.5311; Found: 1109.5300.

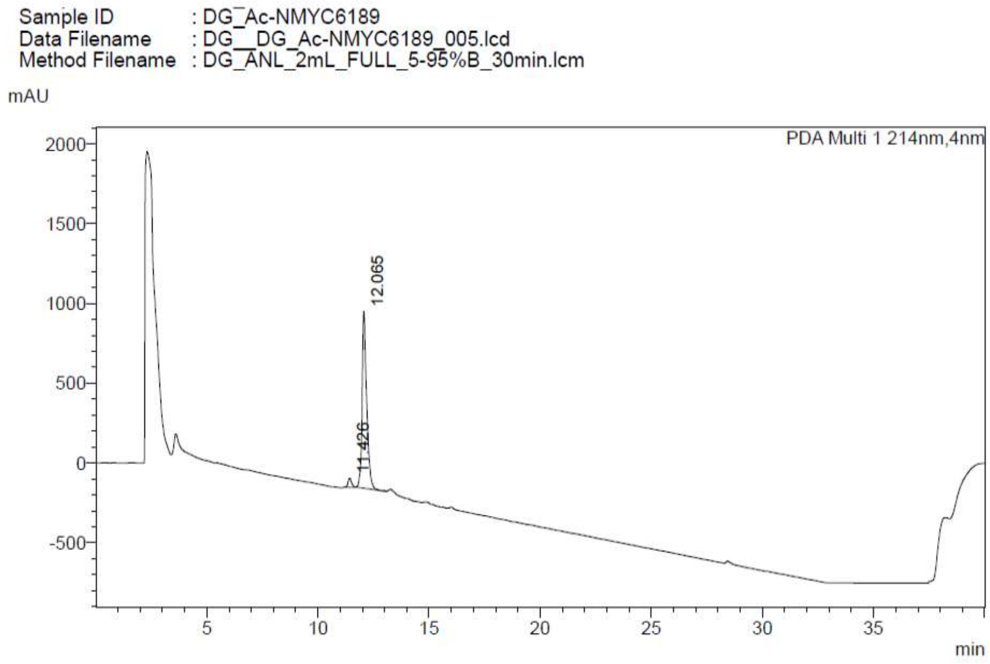

Analytical HPLC trace at λ= 214 nm of purified peptide **N-Myc $_{61\text{-}89}$ Ahx-FAM**

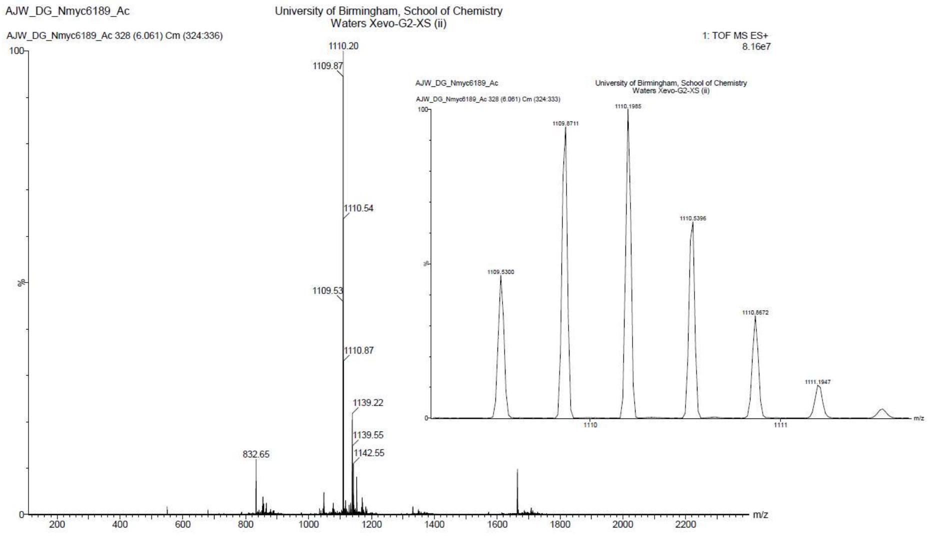

HR-QToF(ESI+)MS analysis of purified peptide **N-Myc $_{61\text{-}89}$ Ahx-FAM**

N-Myc $_{61\text{-}89}$ Ahx-FAM

Sequence: FAM-Ahx- LSPSRGFAEHSSEPPSWVTEMLLENELWG

HPLC (λ = 214 nm) tR = 12.12 min. HR-QToF (ESI) m/z: [M+2H]2+ Calc. for $C_{174}H_{239}N_{39}O_{53}S$: 1878.3536; Found: 1878.3467.

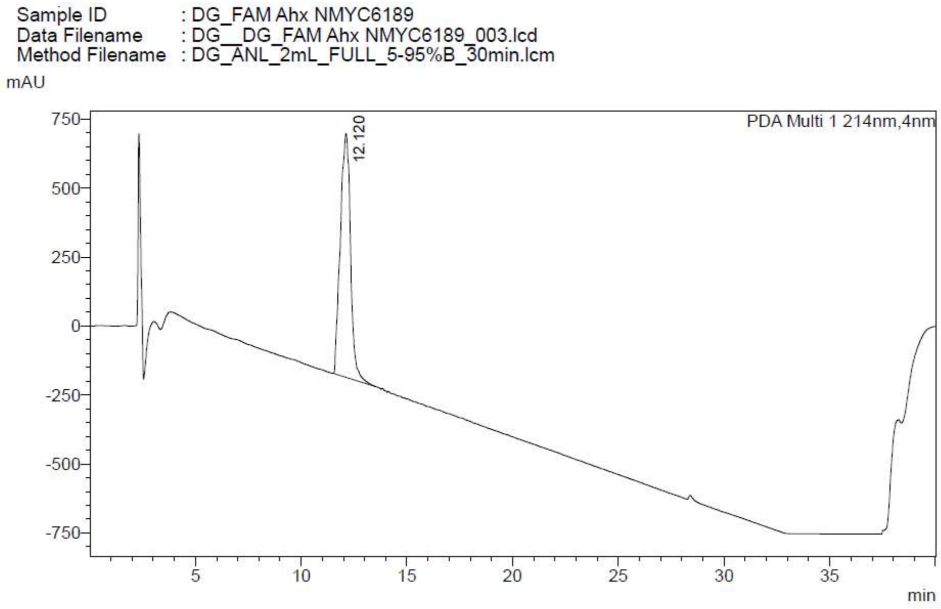

Analytical HPLC trace at λ= 214 nm of purified peptide **N-Myc $_{61\text{-}89}$ Ahx-FAM**

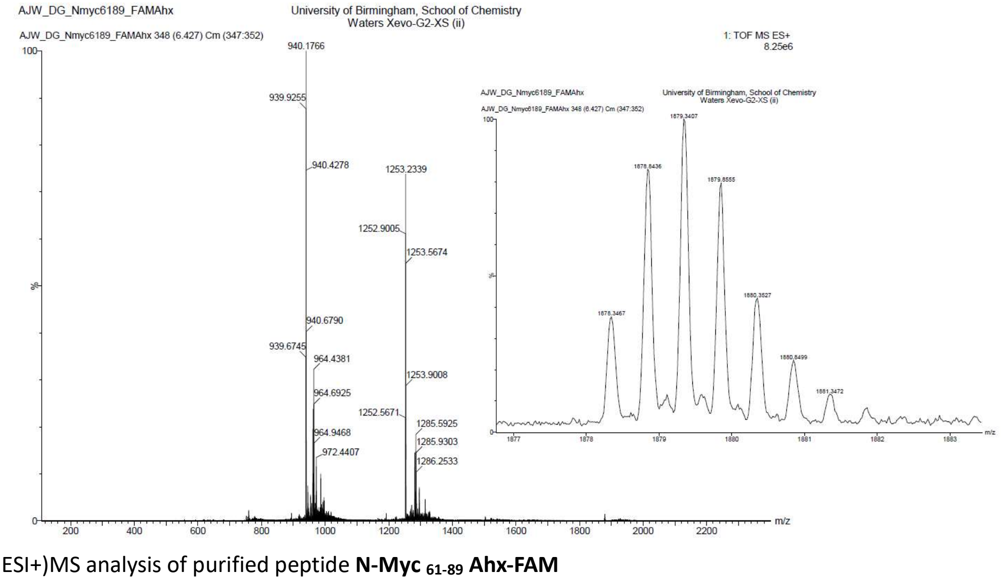

HR-QToF(ESI+)MS analysis of purified peptide **N-Myc $_{61\text{-}89}$ Ahx-FAM**
